# Quantitative and causal analysis for inflammatory genes and the risk of Parkinson’s disease

**DOI:** 10.3389/fimmu.2023.1119315

**Published:** 2023-02-28

**Authors:** Minhan Yi, Jiaxin Li, Shijie Jian, Binbin Li, Zini Huang, Li Shu, Yuan Zhang

**Affiliations:** ^1^ Department of Respiratory Medicine, Xiangya Hospital, Central South University, Changsha, Hunan, China; ^2^ School of Life Sciences, Central South University, Changsha, Hunan, China; ^3^ National Clinical Research Center for Geriatric Disorders, Xiangya Hospital, Central South University, Changsha, Hunan, China; ^4^ Xiangya School of Medicine, Central South University, Changsha, Hunan, China; ^5^ Bangor College, Central South University of Forestry and Technology, Changsha, Hunan, China; ^6^ National Health Commission Key Laboratory for Birth Defect Research and Prevention, Hunan Provincial Maternal and Child Health Care Hospital, Changsha, Hunan, China

**Keywords:** inflammation, Parkinson’s disease, genetics, polymorphism, causal analysis

## Abstract

**Background:**

The dysfunction of immune system and inflammation contribute to the Parkinson’s disease (PD) pathogenesis. Cytokines, oxidative stress, neurotoxin and metabolism associated enzymes participate in neuroinflammation in PD and the genes involved in them have been reported to be associated with the risk of PD. In our study, we performed a quantitative and causal analysis of the relationship between inflammatory genes and PD risk.

**Methods:**

Standard process was performed for quantitative analysis. Allele model (AM) was used as primary outcome analysis and dominant model (DM) and recessive model (RM) were applied to do the secondary analysis. Then, for those genes significantly associated with the risk of PD, we used the published GWAS summary statistics for Mendelian Randomization (MR) to test the causal analysis between them.

**Results:**

We included 36 variants in 18 genes for final pooled analysis. As a result, *IL-6* rs1800795, *TNF-α* rs1799964, *PON1* rs854560, *CYP2D6* rs3892097, *HLA-DRB* rs660895, *BST1* rs11931532, *CCDC62* rs12817488 polymorphisms were associated with the risk of PD statistically with the ORs ranged from 0.66 to 3.19 while variants in *IL-1α, IL-1β, IL-10, MnSOD, NFE2L2, CYP2E1, NOS1, NAT2, ABCB1, HFE* and *MTHFR* were not related to the risk of PD. Besides, we observed that increasing ADP-ribosyl cyclase (coded by *BST1*) had causal effect on higher PD risk (OR[95%CI] =1.16[1.10-1.22]) while PON1(coded by *PON1*) shown probably protective effect on PD risk (OR[95%CI] =0.81[0.66-0.99]).

**Conclusion:**

Several polymorphisms from inflammatory genes of *IL-6, TNF-α, PON1, CYP2D6, HLA-DRB, BST1, CCDC62* were statistically associated with the susceptibility of PD, and with evidence of causal relationships for ADP-ribosyl cyclase and PON1 on PD risk, which may help understand the mechanisms and pathways underlying PD pathogenesis.

## 1 Introduction

Parkinson’s disease (PD) is one of the most common neurodegenerative diseases, the main risk factors for which are genetic background, environmental variables, aging, and their interactions (1). Its typical pathological changes include the formation of α-synuclein (α-syn) positive inclusion bodies in neurons and axons (Lewy bodies and Lewy neurites) and the loss of dopaminergic neurons (1). Resting tremor, stiffness, bradykinesia, and other clinical symptoms of PD are brought on by the increasing weakening of dopaminergic neurons in the substantia nigra (2). Currently, a great amount of clinical and genetic evidences has revealed that inflammation and immune system malfunction are related to the development of PD (3, 4).

According to some theories, both central and peripheral inflammation begin to manifest in the prodromal stage of PD and remain as the condition worsens (4). The origin of inflammation arises from the central nervous system (CNS), where resting microglia are activated by α-syn, triggering an inflammatory cascade response that leads to the death of dopaminergic neurons (3, 5). Particularly, the activated microglia can release pro-inflammatory cytokines such as interleukins (ILs) and tumor necrosis factor-α (TNF-α), which eventually produce damage to dopaminergic neurons (6, 7). To make matters worse, immune cells from the peripheral circulation infiltrate the brain parenchyma through the compromised blood-brain barrier (BBB) and trigger immune responses *via* several pathways (8–10). Meanwhile, higher levels of inflammatory factors released by immune cells, such as IL-6, IL-1β, and TNF-α are also found in peripheral blood of PD patients, indicating the occurrence of peripheral inflammation (11, 12). However, it is important to note that the activation of peripheral inflammatory is nonspecific and can be evaluated using some generalized markers like neutrophil-to-lymphocyte ratio (NLR) and platelet-to-lymphocyte ratio (PLR) (13). The discordant central inflammatory response is enhanced concurrently with peripheral immune system activation, which may be a factor exacerbating the neurodegeneration (4).

Besides, autoimmunity and the impairment in resolving inflammation also participate in the PD-related inflammation response and promote the development of PD (10, 14, 15). There are a high number of infiltrating T cells in the ventral midbrain of PD patients, which are autoreactive and can recognize disease-altered self-proteins (e.g., α-syn) as foreign antigens through histocompatibility complex (MHC) molecules and drive helper and cytotoxic T cell responses (10, 15). The alleles and haplotypes of MHC class II genes, like *HLA-DRB*, has been extensively studied in its association with the risk of PD (8, 16, 17). Physiologically, a carefully regulated immune network is involved in mitigating the progression of inflammation to reduce the tissue damage it causes (14, 18). The balance between effector T cells and regulatory T cells in circulation and some specialized pro-resolving lipid mediators in CNS contribute to the resolution of neuroinflammation and the maintenance of immune homeostasis (18, 19). Accelerating the resolution of early neuroinflammation induced by α-syn could prevent the damage of dopaminergic neurons and the onset of PD (20).

Furthermore, due to mitochondrial dysfunction, inflammation can also be triggered by oxidative stress, which is a significant factor in neurodegeneration (21). Oxidative stress and inflammation interact to produce excitotoxicity, neuronal degeneration, and axonal damage, all of which are eventually significant contributors to the development of PD (22). Studies have shown that Nrf2, nitric oxide synthase (NOS), manganese superoxide dismutase (MnSOD), cytochrome P450s (CYPs), hemochromatosis (HFE) and methylenetetrahydrofolate reductase (MTHFR) participate in the development and progress of PD through pathways related to the oxidative stress, including mitochondrial dysfunction, DNA damage, nerve cell apoptosis, and neuroinflammation (23–27).

Overall, the innate and adaptive immune systems play critical roles in the neuroinflammatory process in PD, including oxidative stress, activation and infiltration of immune cells, and the production of inflammatory mediators (3, 21, 28). Single nucleotide polymorphisms (SNPs) of immunological and inflammatory genes can influence the risk of PD by influencing the immune system and inflammatory response since PD is directly tied to genetics. Genetic factors in PD converge on immune function and inflammation through the activation of immune cells and the release of inflammatory mediators (29). Changes in the inflammatory genes may make a person more vulnerable to the formation of oxidative stress and the activation of the neuroimmune system, both of which can result in the death of dopaminergic neurons (30, 31). Researches on inflammatory polymorphic locus identified by genome-wide association studies (GWAS) study have also exemplified the significance of neuroinflammation in the pathogenesis of PD (32, 33).

Although previous studies have shown a close relationship between inflammation and PD, few studies have investigated the causality between them. Mendelian Randomization (MR) is a reliable genetic epidemiology method, which uses genetic variants as instrumental variable (IV) to assure whether causality exists between exposure and outcome, maybe a powerful tool to explore the causality between inflammation and PD (34). Bottigliengo D et al. investigated the causal role of inflammation on PD by conducting MR analysis (35). They included C-reactive protein (CRP), IL-6, IL-1 receptor antagonist and TNF-α in a two-sample MR analysis and suggested the pro-inflammatory activity of IL-6 could be a determinant of prodromal PD. Nevertheless, other than this study, no other articles have been reported on the causal relationship between inflammation and PD.

**Figure 1:**
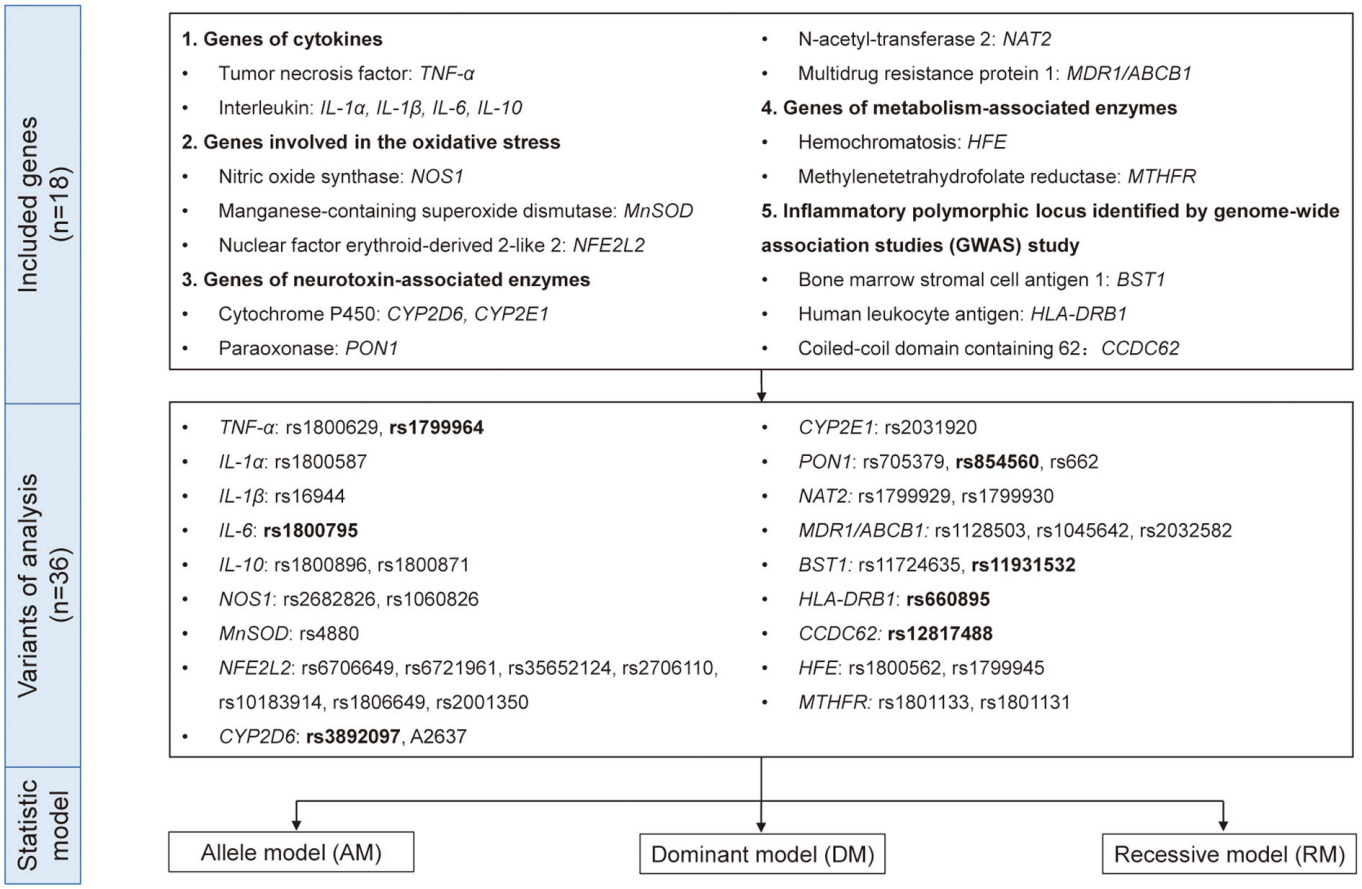
The genes, variants, and data analysis models included in the quantitative analysis. Eighteen genes with 36 variants from five different functional types were included in this study. We used allele model (AM), dominant model (DM), and recessive model (RM) for quantitative analysis and the variants in bold indicated p<0.05 in either model.

Therefore, to reach a comprehensive and updated conclusion, we performed a quantitative and causal analysis to explore the role of inflammatory genes in PD risk in order to bring new understanding of the mechanisms and pathways underlying PD pathogenesis and may provide the theoretical basis for finding the potential biomarkers and implementing anti-inflammatory and immunological treatment in PD. In addition to the genes included in the existing studies, we collected the original researches related to inflammation-related genes and PD as much as possible. Based on the function of genes, we divided them into five groups: genes of cytokines, genes involved in the oxidative stress, genes of neurotoxin-associated enzymes, genes of metabolism-associated enzymes and inflammatory polymorphic locus identified by GWAS study (**Figure 1**). Our study would be an important supplement on the topic of PD’s genetic susceptibility and also provide idea related to its mechanism and treatment.

## 2 Methods

### 2.1 Quantitative analysis of associations between inflammatory genes and PD

#### 2.1.1 Literature searching

Researchers independently retrieved and screened literature, and the inconsistent views were discussed with the third party. Key words were “Parkinson’s disease”, “Parkinso*”, “variants”, “genetic”, “specific genes” (*TNF-α, IL-6, IL-1α, IL-1β, IL-10, NOS1, MnSOD, NFE2L2, CYP2D6, PON1, CYP2E1, NAT2, ABCB1, BST1, HLA-DRB, CCDC62, HFE, MTHFR* involved in five different inflammation-related group (genes of cytokines, genes involved in the oxidative stress, genes of neurotoxin-associated enzymes, genes of metabolism-associated enzymes and inflammatory polymorphic locus identified by GWAS study) in PD). The detailed searching strategy was listed in **Supplementary Table 1**.

#### 2.1.2 Inclusion and exclusion criteria

Inclusion criteria using PICOS (participants, interventions, comparators, outcomes, and studies approach) were applied to screen articles:

Participants: the PD diagnosis from each researched cohort was according to widely accepted criteria (36, 37).

Interventions: genetic sequencing of variants in inflammation-related genes of interest were performed by PCR-based methods or other accepted methods;

Controls: controls were neither having PD nor other neurological diseases.

Outcomes: available data to calculate the number of carriers and non-carriers of the gene variants.

Studies approach: original studies provided sufficient data to do pooled analysis.

Exclusion criteria including: 1) neurological diseases not PD or without control groups; 2) not original studies including editorial, review, systematic review etc.; 3) functional studies using animal or cell models; 4) studies not having sufficient data to calculate odd ratio (OR) and 95% confidence interval (CI) in all models.

#### 2.1.3 Data extraction and quality control

Then, authors independently extracted the detailed information from the included studies. The data extraction table were as follows: first author, publication year, ethnics, number of allele carriers in cases or controls, number of cases, number of controls, number of genotype carriers in cases, number of genotypes in controls. The Newcastle-Ottawa Scale (NOS) scores were used to evaluate the quality of the included articles. If there was any disagreement on data extraction, a third researcher was asked to make a decision.

#### 2.1.4 Statistics analysis for quantitative analysis

Revman 5.3 software was used to calculate pooled OR and 95%CI. Three models were applied to do the association analyses: allele model (AM, indicated “a” distribution between case group and control group), dominant model (DM, indicated “aa + Aa” distribution between case group and control group), and recessive model (RM, indicated “aa” distribution between case group and control group). “A” represented wild type allele, “a” represented mutated allele. P <0.05 was considered statistically significant.

The I^2^ and Q test were performed to analyze the heterogeneity. If I^2^> 50, the random-effect model was used, otherwise if I^2^ ≤ 50, the fix-effect model was applied. The publication bias was measured by the symmetry of funnel plot. If the plot was in a symmetrical shape, no publication bias was shown. Otherwise, publication bias was observed. Sensitivity analysis was performed by sequentially removing one article at a time.

### 2.2 Mendelian randomization analysis investigating causal relationship

#### 2.2.1 Study design for causal analysis

**Figure 2:**
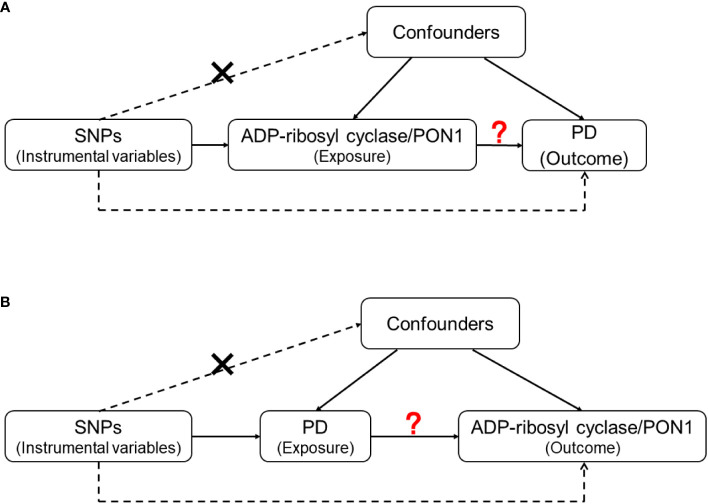
The design of Mendelian Randomization (MR) analysis to assess causality between PD and proteins coded by inflammatory genes. **(A)** SNPs independently associated with ADP-ribosyl cyclase (coded by *BST1*) and PON1(coded by *PON1*) from GWAS summary statistic were used as instrumental variables to explore the causal effect of ADP-ribosyl cyclase and PON1 on PD. **(B)** SNPs independently associated with PD from GWAS summary statistic were used as instrumental variables to explore the causal effect of PD on ADP-ribosyl cyclase and PON1 respectively. In addition to the association assumption, another two assumptions of MR include: (1) SNPs are not associated with the confounders of exposure and outcome; (2) there is no feasible pathway between the genetic variations and outcome other than through exposure.

For the genes with statistically significant results in the quantitative analysis, we further explored the causality between proteins they encode and the risk of PD by conducting a two-sample MR analysis. After searching the GWAS data of the included genes, we evaluated the causal associations between corresponding proteins [ADP-ribosyl cyclase (coded by *BST1*) and PON1 (coded by *PON1*)] and PD in two directions (**Figure 2**).

#### 2.2.2 GWAS data sources

We used a published GWAS summary statistics from International Parkinson Disease Genomics Consortium (IPDGC) Study that contained 482,730 individuals with 37,688 PD from Europe (38). ADP-ribosyl cyclase GWAS summary statistics were obtained from a German cohort included 997 European (39). In this study, the plasma ADP-ribosyl cyclase levels of participants were quantified by proteomics measurements using the SOMAscan platform. Besides, we also include the PON1 GWAS summary statistics from the Milieu Intérieur cohort which contained 400 participants from Europe (40). The level of PON1 in plasm were quantified by protein immunoassay. The specific information was summarized in **Table 1**.

**Table 1:** Summary of genome-wide association study (GWAS) datasets for MR analysis.

Protein/Disease	Gene	Population	Sources	Ref	Sample size
ADP-ribosyl cyclase	*BST1*	European	a German cohort	(39)	997
PON1	*PON1*	European	the Milieu Intérieur cohort	(40)	400
Parkinson’s disease	/	European	IPDGC	(38)	482730

The International Parkinson Disease Genomics Consortium, IPDGC.

#### 2.2.3 Selection of instrument variable

There are three assumptions for instrumental variable (IV) selection in two-sample MR analysis: (1) the selected genetic variants are associated with the exposure; (2) the used IV variants are not associated with the confounders of exposure and outcome; (3) there is no feasible pathway between the genetic variations and outcome other than through exposure (41). In detail, when using ADP-ribosyl cyclase, PON1 and PD as exposure, we selected associated variants with *p *< 5 × 10^−8^ (38–40). Then, the independently associated variants were included as IV with the criteria of r^2^ < 0.1 within distance of 1000kb.

#### 2.2.4 Mendelian Randomization analysis

We used the method of inverse-variance weighting (IVW) (42) and Mendelian randomization-pleiotropy residual sum and outlier (MR-PRESSO) (43) as the primary outcomes that assumed that all SNPs are valid instrument variables. In sensitivity analyses, we used MR Egger (44) and Weighted median (45) to correct for any potential violations of the assumptions. These methods are performed as they operate in different ways and rely on different assumptions for valid inferences to assess the reliability of MR analysis. Besides, heterogeneity was analyzed by Cochran’s Q-test of IVW and MR Egger, and pleiotropy was tested by the intercept of MR Egger analysis. When heterogeneity was detected for associated relationships, we used the RadialMR package to remove outliers and applied above analysis again (46).

## 3 Results

### 3.1 Quantitative analysis of polymorphisms in inflammation related genes and PD risk

As can be seen from the flowchart (**Figure 1** and **Supplementary Figure 1**), articles were retrieved for each research gene separately using three databases (PubMed, Embase and Web of Science database). By removing overlapping articles, reading title/abstract and full-text screening. Final original articles were included for pooled analysis by different genes separately. The detailed information of included original articles and genotypes distributions were presented in **Table 2** and **Supplementary Tables 2, 3**. Thirty-six variants in 18 genes associated with inflammatory mechanisms in PD were involved. The results of quantitative analysis were presented in **Table 3**. The functions of these genes were classified by five groups: genes of cytokines, genes involved in the oxidative stress, genes of neurotoxin-associated enzymes, genes of metabolism-associated enzymes and inflammatory polymorphic locus identified by GWAS study.

**Table 2:** The characteristics of all included publications for quantitative analysis.

Year	First author	Ref	Region/ Country	Number of cases/controls	Included genes	NOS
East Asian						
2020	Chang, K. H.	(16)	China	486/473	*HLA-DRB1*	8
2016	Gui, Y.	(47)	China	765*/489*	*NFE2L2*	7
2016	Liu, Z.	(48)	China	460/473	*IL-10*	7
2015	Chang, K. H.	(49)	China	596/597	*BST1*	7
2015	Guo, J. F.	(50)	China	1061*/1066*	*BST1*	9
2015	Yu, R. L.	(51)	China	507/518	*CCDC62*	9
2014	Chen, M. L.	(52)	China	468/487	*BST1*	7
2014	Liao, Q.	(53)	China	765/717	*MTHFR*	9
2014	Liu, R. R.	(54)	China	341/423	*CCDC62*	8
2013	Chen, Y. C.	(55)	China	480/526	*NFE2L2*	6
2013	Kiyohara, C.	(56)	Japan	238/368	*MDR1/ABCB1*	7
2013	Li, N. N.	(57)	China	783*/725*	*CCDC62*	8
2013	Nie, K.	(58)	China	302/294	*IL-10*	7
2012	Li, D.	(59)	China	355/200	*IL-10*	6
2012	Miyake, Y.	(60)	Japan	229/357	*BST1*	9
2011	Fong, C. S.	(61)	China	211/218	*MTHFR*	9
2011	Chang, X. L.	(62)	China	636*/510*	*BST1*	8
2010	Wang, V. C.	(63)	China	295/111	*MnSOD*	9
2009	Yuan, R. Y.	(64)	China	76/110	*MTHFR*	8
2008	Zhou, Y. T.	(65)	China	533/530	*IL-1α*	6
2007	Wu, Y. R (1).	(66)	China	493/388	*IL-1α, IL-1β*	8
2007	Wu, Y. R (2).	(67)	China	369/326	*TNF-α*	6
2005	Fong, C. S.	(68)	China	125/162	*PON1*	6
2005	Nishimura, M.	(69)	Japan	361/257	*IL-1β*	5
2005	Tan, E. K.	(70)	China	185/206	*MDR1/ABCB1*	8
2002	Wu, R. M.	(71)	China	234/251	*CYP2E1*	9
2001	Nishimura, M.	(72)	Japan	172/157	*TNF-α*	6
2001	Woo, S. I.	(73)	Korea	93/122	*CYP2D6*	9
2000	Nishimura, M.	(74)	Japan	122*/112	*IL-1α, IL-1β*	6
2000	Wang, J (1).	(75)	China	180/180	*PON1*	7
2000	Wang, J (2).	(76)	China	150/150	*CYP2E1*	7
2000	Yasui, K.	(77)	Japan	90/50*	*MTHFR*	7
1998	Kondo, I.	(78)	Japan	166*/252	*PON1*	6
European Caucasian/West Asian						
2019	Mota, A.	(32)	Iran	70/75	*PON1*	6
2017	Chuang, Y. H. (i)	(79)	Denmark	1547/1595	*HLA-DRB1*	7
2017	Ran, C.	(80)	Sweden	501*/509*	*NFE2L2*	8
2016	Gupta, S. P.	(81)	India	89/332	*NOS1*	9
2016	Paul, K. C.	(82)	America	357*/495*	*NOS1*	8
2016	Zahra, C.	(83)	Malta	178*/402*	*MTHFR*	7
2015	Todorovic, M.	(84)	Australia	1338*/1379*	*NFE2L2*	8
2014	Kumudini, N.	(85)	India	151/416	*MTHFR*	8
2013	Lee, P. C.	(86)	America	287*/440*	*PON1*	8
2012	Belin, A. C.	(87)	Sweden	512*/550*	*PON1*	6
2012	San Luciano, M.	(88)	America	381/521*	*IL-6*	7
2011	Punia, S.	(89)	India	487/474	*PON1*	6
2010	Manthripragada, A. D. (i)	(90)	America	282/290	*PON1*	8
2010	Singh, M.	(91)	India	77/125	*CYP2D6, NAT2*	8
2010	von Otter, M. (i)	(92)	Sweden	165*/190*	*NFE2L2*	6
2010	von Otter, M. (ii)	(92)	Poland	192*/192*	*NFE2L2*	6
2009	Camicioli, R. M.	(93)	Canada	51*/50*	*MTHFR*	8
2009	Funke, C.	(94)	Germany	300/302*	*MDR1/ABCB1*	6
2009	Westerlund, M.	(95)	Sweden	288*/313*	*MDR1/ABCB1*	8
2009	Zschiedrich, K. (i)	(96)	Germany	265/123	*MDR1/ABCB1*	8
2009	Zschiedrich, K. (ii)	(96)	Serbia	42/61	*MDR1/ABCB1*	8
2008	Bialecka, M.	(97)	Poland	316/300	*IL-10*	8
2008	Halling, J.	(98)	Denmark	79/153	*HFE, CYP2D6*	7
2008	Singh, M.	(99)	India	70/100	*MnSOD, CYP2E1*	8
2007	Aamodt, A. H.	(100)	Norway	388/505	*HFE*	6
2007	Bialecka, M.	(101)	Poland	341/315	*IL-10*	9
2007	Wahner, A. D.	(102)	America	289/269	*IL-1β, TNF-α*	8
2006	Guerreiro, R. J.	(103)	Portugal	132/115	*HFE*	7
2006	Religa, D.	(104)	Poland	114/100	*MTHFR*	9
2006	Todorovic, Z.	(105)	Serbia and Montenegro	113/53	*MTHFR*	9
2005	Hakansson, A. (1)	(106)	Sweden	265*/308*	*IL-10*	6
2005	Hakansson, A. (2)	(107)	Sweden	265*/308	*IL-6*	6
2005	Wullner, U.	(108)	UK	342/342	*MTHFR*	8
2004	Clarimon, J.	(109)	Finland	144*/135*	*PON1*	7
2004	Hague, S.	(110)	Finland	147*/137*	*NOS1*	6
2004	Moller, J. C.	(111)	Germany	176/170	*IL-1α*	6
2004	Ross, O. A.	(112)	Ireland	90/93	*IL-6, TNF-α*	6
2004	Tan, E. K.	(113)	Poland	158/139	*MDR1/ABCB1*	7
2003	Dekker, M. C.	(114)	Netherlands	197/2914	*HFE*	6
2003	Drozdzik, M.	(115)	Poland	107/103	*MDR1/ABCB1*	8
2003	Kelada, S. N.	(116)	America	150*/244*	*PON1*	6
2002	Buchanan, D. D.	(117)	Australia	438/485	*HFE*	9
2002	Carmine, A.	(118)	Sweden	114*/127*	*PON1*	6
2002	Mattila, K. M.	(119)	Finland	52/73	*IL-1α, IL-1β*	8
2002	McGeer, P. L.	(120)	Canada	100/100	*IL-1α, IL-1β*	6
2002	Schulte, T.	(121)	Germany	295*/270*	*IL-1α, IL-1β*	6
2001	Akhmedova, S. N.	(122)	Russia	117/207	*PON1*	7
2001	Dodel, R. C.	(123)	Germany	201/197	*IL-1α*	7
2001	Payami, H.	(124)	America	576/247	*CYP2D6*	8
2000	Kruger, R.	(125)	Germany	264*/183*	*TNF-α*	7
2000	Taylor, M. C.	(126)	Australia	92/122	*PON1*	7
1999	Akhmedova, S.	(127)	Russia	121/117	*PON1*	8
1999	Atkinson, A.	(128)	UK	33/75	*CYP2D6*	8
1999	Grasbon-Frodl, E. M.	(129)	Germany	44/42	*MnSOD*	6
1999	Nicholl, D. J.	(130)	UK	206*/206*	*CYP2D6, NAT2*	9
1996	Diederich, N.	(131)	Germany	80/108*	*CYP2D6*	9
1994	Plante-Bordeneuve, V.	(132)	UK and Ireland	48/88	*CYP2D6*	8
1993	Kurth, M. C.	(133)	America	50/110	*CYP2D6*	7
Latino						
2018	Agliardi, C.	(134)	Italy	354/443	*TNF-α*	8
2017	Chuang, Y. H. (ii)	(79)	France	509/1128	*HLA-DRB1*	7
2016	Mariani, S.	(135)	Italy	92*/112*	*HFE*	7
2015	Narayan, S.	(136)	France	286*/580*	*MDR1/ABCB1*	6
2013	Mariani, S.	(137)	Italy	78*/139	*HFE*	9
2012	Ahmed, I.	(138)	France	499/1122	*HLA-DRB1*	8
2012	Gorgone, G.	(139)	Italy	60/82	*MTHFR*	8
2011	Greco, V.	(140)	Italy	181/180	*HFE*	7
2011	Pascale, E.	(141)	Italy	146/156	*IL-1β, IL-10, TNF-α*	7
2010	Dutheil, F.	(142)	France	207/482	*MDR1/ABCB1*	6
2009	Rodriguez-Oroz, M. C.	(143)	Spain	89*/30*	*MTHFR*	7
2008	Infante, J.	(144)	Spain	197*/173*	*IL-1α, IL-6, IL-10, TNF-α*	8
2007	Caccamo, D.	(145)	Italy	49/86	*MTHFR*	8
2007	Huerta, C.	(146)	Spain	450/200	*NOS1*	7
2006	Borlak, J.	(147)	Italy	124/243	*NAT2*	9
2004	Elbaz, A.	(148)	France	190/419	*CYP2D6*	7
2003	Levecque, C.	(149)	France	209/488	*NOS1*	7
2002	Borie, C.	(150)	France	216*/193*	*HFE*	6
2002	Furuno, T.	(151)	Italy	95/106	*MDR1/ABCB1*	9
1996	Bordet, R.	(152)	France	105/105	*CYP2D6*	7
1996	Lucotte, G.	(153)	France	47/47	*CYP2D6*	8
Mixed (exclude Caucasion)						
2010	Manthripragada, A. D. (ii)	(90)	America	351/363	*PON1*	8
1996	Gasser, T.	(154)	America	115/73	*CYP2D6*	7
1995	Chen, X.	(155)	America	28*/212*	*CYP2D6*	7

* represents the number of case/control in pool analysis is different from which is written in the original article due to censoring or data unavailable. (1), (2) represent different articles with the same publication year and first authors. (i), (ii), (iii) represent different cohorts from the same paper. The classification of ethnicity depends on the original description in each article primarily. If race description lacking, the classification would depend on its region. “East Asian” refers to residents from China, Japan, Korea or Singapore. “European Caucasian/West Asian” refers to residents from Europe, America, India, north and west part of Africa and other Caucasus region. “Latino” refers to Latino, Portuguese, Spanish, Italian, French and Spanish-or- Portuguese -spoken residents from Latin.

**Table 3:** The results of quantitative analysis for the association between included variants and the risk of PD in different models.

Gene	Variant	Sample size#	Allele	OR [95%CI]		
			Ref/Alt	Allele model	Dominant model	Recessive model
Genes of cytokines						
*TNF-α*	rs1800629	6/1485/1464	G/A	1.12 [0.96, 1.30]	1.11 [0.94, 1.31]	1.33 [0.83, 2.13]
	rs1799964	3/735/653	T/C	1.24 [0.85, 1.79]	1.10 [0.70,1.72]	3.19 [1.66,6.13]***
*IL-1α*	rs1800587	9/2129/2000	C/T	1.03[0.91,1.16]	1.01 [0.88, 1.16]	1.17 [0.84, 1.64]
*IL-1β*	rs16944	8/1857/1622	C/T	1.05 [0.85, 1.31]	1.06 [0.84, 1.33]	1.16 [0.77, 1.74]
*IL-6*	rs1800795	4/924/1093	G/C	0.82 [0.63, 1.06]	0.66 [0.55, 0.79]****	0.85 [0.55, 1.30]
*IL-10*	rs1800896	6/1557/1540	A/G	1.00 [0.90, 1.11]	1.04 [0.88, 1.23]	0.94 [0.78, 1.14]
	rs1800871	4/1472/1288	C/A	1.10 [0.98, 1.24]	1.07 [0.89, 1.29]	1.18 [0.98, 1.42]
Genes involved in the oxidative stress						
*NOS1*	rs2682826	5/1246/1606	C/T	1.11 [0.89,1.38]	1.11 [0.86,1.45]	1.23 [0.92,1.66]
	rs1060826	3/949/764	G/A	1.07 [0.86,1.34]	1.14 [0.94,1.40]	1.02 [0.53, 1.96]
*MnSOD*	rs4880	3/409/253	T/C	1.14 [0.86, 1.53]	1.08 [0.71, 1.64]	1.52 [0.82, 2.82]
*NFE2L2*	rs6706649	4/2048/1869	G/A	1.02 [0.88, 1.19]	0.99 [0.84, 1.17]	1.44 [0.82, 2.52]
	rs6721961	4/2076/1873	C/A	1.02[0.91,1.14]	1.01 [0.88,1.16]	1.04 [0.79,1.35]
	rs35652124	4/2076/1868	A/G	1.03 [0.93, 1.13]	0.99 [0.81,1.21]	1.10 [0.92,1.31]
	rs2706110	3/2399/2191	G/A	1.06 [0.87, 1.30]	1.08 [0.83, 1.41]	0.99 [0.76, 1.30]
	rs10183914	3/2405/2210	G/A	0.95 [0.87, 1.04]	0.95 [0.85, 1.07]	0.91 [0.76, 1.08]
	rs1806649	3/2412/2199	G/A	0.92 [0.77, 1.11]	0.94 [0.83, 1.07]	0.90 [0.56, 1.46]
	rs2001350	3/2141/2207	A/G	1.06[0.92,1.23]	1.09[0.83,1.43]	1.27[0.67,2.42]
Genes of neurotoxin-associated enzymes						
*CYP2D6*	rs3892097	14/1727/2087	G/A	1.14 [1.00, 1.29]*	1.29 [1.02, 1.63]*	1.06 [0.78, 1.44]
	A2637	5/485/598	A/-	1.12 [0.58, 2.16]	1.12 [0.58, 2.18]	NA
*PON1*	rs705379	3/908/1158	C/T	0.96 [0.85,1.08]	0.97 [0.80,1.18]	0.92 [0.75,1.12]
	rs854560	11/2781/3176	T/A	1.20 [1.10, 1.30]***	1.21 [1.08, 1.35]***	1.37 [1.15, 1.62]***
	rs662	10/2205/2538	A/G	1.01 [0.92, 1.10]	0.99 [0.88,1.12]	1.05 [0.88,1.24]
*CYP2E1*	rs2031920	3/454/501	C/T	1.13 [0.89, 1.44]	1.14 [0.86, 1.52]	1.31 [0.62, 2.76]
*NAT2*	rs1799929	3/406/573	C/T	0.96 [0.80, 1.16]	1.03 [0.79, 1.36]	0.84 [0.59, 1.18]
	rs1799930	3/405/573	G/A	1.02 [0.80, 1.30]	1.02 [0.75, 1.37]	1.06 [0.61, 1.83]
*MDR1/* *ABCB1*	rs1128503	4/918/918	C/T	0.98 [0.86, 1.12]	1.11 [0.91, 1.36]	0.84 [0.59, 1.19]
	rs1045642	10/2159/2753	C/T	1.06 [0.97, 1.15]	1.04 [0.91, 1.19]	1.12 [0.98, 1.28]
	rs2032582	7/1499/2091	T/G(A)	0.96 [0.87, 1.06]	0.99 [0.83, 1.17]	0.93 [0.80, 1.07]
Genes of metabolism-associated enzymes						
*HFE*	rs1800562	9/1644/4654	G/A	0.89 [0.73, 1.08]	0.88[0.72, 1.08]	0.93 [0.35, 2.51]
	rs1799945	8/1217/4151	C/G	1.03 [0.89, 1.19]	1.02 [0.87, 1.21]	1.18 [0.70, 1.99]
*MTHFR*	rs1801133	13/2250/2565	C/T	1.11 [0.93, 1.34]	1.15 [0.90, 1.47]	1.02 [0.85, 1.23]
	rs1801131	4/550/527	A/C	1.05 [0.75, 1.47]	0.97 [0.76, 1.23]	0.77 [0.51, 1.14]
Inflammatory polymorphic locus identified by genome-wide association studies (GWAS) study						
*BST1*	rs11724635	3/1293/1441	C/A	1.07 [0.96, 1.20]	1.08 [0.92, 1.27]	1.12 [0.92, 1.37]
	rs11931532	3/1868/3782	T/C	0.90 [0.82, 0.99]*	0.91 [0.78, 1.06]	0.82 [0.70, 0.96]*
*HLA-DRB1*	rs660895	3/3041/4318	A/G	0.80 [0.74, 0.87]****	0.79 [0.71, 0.87]****	0.67 [0.52, 0.86]**
*CCDC62*	rs12817488	3/1608/1649	A/G	0.80 [0.73, 0.89]***	0.77 [0.66, 0.89]***	0.74 [0.62, 0.87]***

# Number of articles/patients/controls included for quantitative analysis. The Allele (Ref/Alt) represents refer allele and alter allele, respectively. OR[95%CI] represents the odd ratios with 95% confidence interval. The values of OR [95%CI] in bold indicate statistically significant. The */**/***/**** represents the significant variants with p< 0.05, p<0.01, p<0.001, p<0.00001, respectively.

#### 3.1.1 Genes of cytokines

Seven variants in five genes (*TNF-α, IL-6, IL-1α, IL-1β, IL-10*) were included in the pooled analysis. In the results of DM and RM models, rs1799964 of *TNF-a* (RM: OR[95%CI] = 3.19[1.66,6.13], p=0.0005) polymorphism was positively associated with PD risk. In contrary, rs1800795 of *IL-6* (DM: OR[95%CI] = 0.66 [0.55, 0.79], p<0.00001) polymorphism was negatively associated with PD risk. About variants in *IL-1α* (rs1800587), *IL-1β*(rs16944), *IL-10* (rs1800871, rs1800896), all three models (AM, DM, RM) showed these variants were not associated with the risk of PD.

#### 3.1.2 Genes involved in the oxidative stress

Ten variants in three genes (*NOS1, MnSOD, NFE2L2*) were included for quantitative analysis. We failed to identify the association between *NFE2L2* rs6706649, rs6721961, rs35652124, rs2706110, rs10183914, rs1806649, rs2001350, *NOS1* rs2682826, rs1060826, *MnSOD* rs4880 and PD risk in all three models (AM, DM, RM).

#### 3.1.3 Genes of neurotoxin-associated enzymes

Eleven variants in five genes (*CYP2D6, PON1, CYP2E1, NAT2, ABCB1/MDR*) were included in the AM for quantitative analysis. *CYP2D6* rs3892097 (OR[95%CI] =1.14[1.00-1.29], p=0.04) variant was positively associated with PD risk on 1727 PD cases and 2087 controls. *PON1* rs854560 (OR[95%CI] =1.20 [1.10, 1.30], p<0.0001) variant was also positively associated with PD risk in AM on 2781 PD cases and 3176 controls.

In the secondary analysis, by including 2781 PD cases and 3176 controls in DM and RM, *PON1* rs854560 variant was positively associated with PD risks (DM: OR[95%CI] =1.21 [1.08-1.35], p=0.0007; RM: OR[95%CI] =1.37[1.15-1.62], p= 0.0003). The significant result was also replicated by DM in *CYP2D6* variant rs3892097 (OR[95%CI] =1.29[1.02-1.63], p=0.04). Variant in *CYP2E1* (rs2031920) was not associated with PD risk. Variants in *NAT2* (rs1799929, rs1799930) or *ABCB1* (rs1128503, rs1045642, rs2032582) were not associated with PD risk either.

#### 3.1.4 Genes of metabolism-associated enzymes

Four variants in two genes (*HFE, MTHFR*) were included for quantitative analysis. We failed to identify the association between *HFE* rs1800562, rs1799945, *MTHFR* rs1801133, rs1801131 and PD risk in all three models (AM, DM, RM).

#### 3.1.5 Inflammatory polymorphic locus identified by GWAS study

We included four variants in three genes (*BST1, HLA-DRB, CCDC62*) in the pooled analysis. *BST1* rs11931532 was negatively related to PD risk in pooled analysis on 1868 PD patients and 3782 controls (AM: OR[95%CI] =0.90[0.82-0.99], p=0.02). In the further analysis, the significant results were also presented in RM about *BST1* rs11931532 (OR[95%CI] =0.82[0.70-0.96], p=0.01). By including 3041 PD cases and 4318 controls, we found that *HLA-DRB* rs660895 was associated with PD risk negatively in all models (AM: OR[95%CI] = 0.80 [0.74, 0.87], p <0.00001; DM: OR[95%CI]= 0.79 [0.71, 0.87], p<0.00001; RM: OR[95%CI]=0.67[0.52-0.86], p=0.002). *CCDC62* rs12817488 was also associated with decreased PD risk in quantitative analysis on 1608 PD cases and 1649 controls in all models (AM: OR[95%CI] = 0.80 [0.73, 0.89], p <0.0001; DM : OR[95%CI]=0.77[0.66-0.89], p=0.0005; RM OR[95%CI]=0.74[0.62-0.87], p=0.0003).

#### 3.1.6 Statistical sensitivity and bias analysis

Funnel plots of almost all quantitative analysis were symmetric, indicating that there was no publication bias (**Supplementary Figures 2, 3**). We conducted the sensitivity analysis by comparing the changes in pooled p value, OR and 95%CI after deleting each article at a time in turn (**Supplementary Table 4**). After removing Ross, O. A. et al. (112) in *IL-6* rs1800795, Hague, S. et al. (110) in *NOS1* rs1060826, Todorovic, M. et al. (84) in *NFE2L2* rs2001350 or Funke, C. et al. (94) in *MDR1* rs1128503, the pooled p value or OR changed significantly. This could be due to large or small sample sizes, or differences in genotype distribution on account of ethnicity and region compared with other studies.

### 3.2 Mendelian randomization analysis for causal analysis

#### 3.2.1 Causal association between ADP-ribosyl cyclase (*BST1*) and PD

**Figure 3:**
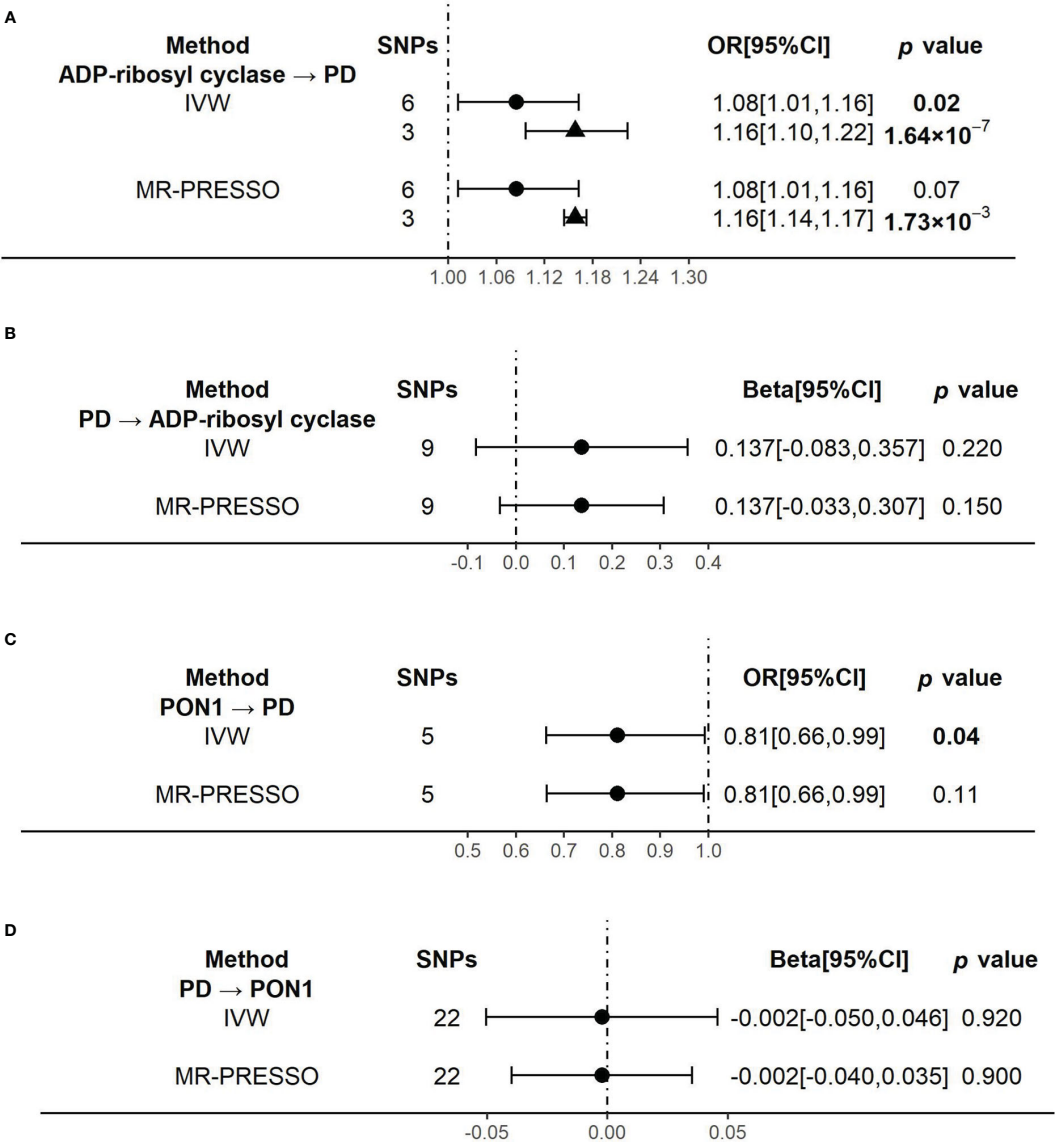
The results of the causal effects between ADP-ribosyl cyclase, PON1 and Parkinson’s disease by Mendelian Randomization analysis **(A)** The causal effect of ADP-ribosyl cyclase on PD. **(B)** The causal effect of PD on ADP-ribosyl cyclase. **(C)** The causal effect of PON1 on PD. **(D)** The causal effect of PD on PON1.. 95% confidence interval, 95% CI; inverse-variance weighted, IVW; Mendelian randomization-pleiotropy residual sum and outlier, MR-PRESSO; Parkinson’s disease, PD. Circulars illustrate the results from primary instrumental variables (IVs) and triangles mean the results from outliers removed IVs.

When using ADP-ribosyl cyclase (coded by *BST1*) as exposure and PD as outcome, the primary outcome of IVW model showed that an increased level of ADP-ribosyl cyclase was causally associated with the higher risk of PD (OR[95%CI] = 1.08 [1.01, 1.16], *p* =0.02) (**Figure 3A**). The result was almost significant in MR-PRESSO model (OR[95%CI] = 1.08 [1.01, 1.16], *p* =0.07) (**Figure 3A**). The results of MR Egger and Weighted median were shown in **Supplementary Table 5**. Since there were high heterogeneity for above analysis (**Supplementary Table 6**), we identified and removed those outliers of SNPs. We confirmed that there was no obvious heterogeneity in all models (**Supplementary Table 6**). And the causality become stronger in both IVW (OR[95%CI] = 1.16 [1.10, 1.22], *p* =1.64×10^-7^] and MR-PRESSO (OR[95%CI] = 1.16 [1.14, 1.17], *p* =1.73×10^-3^] models (**Figure 3A**).

Reversely, when using PD as exposure and ADP-ribosyl cyclase as outcome, the results of all analysis models showed no significant association between them (**Figure 3B** and **Supplementary Table 5**). And there was no evidence of heterogeneity in all models (**Supplementary Table 6**).

#### 3.2.2 Causal association between PON1(*PON1*) and PD

When using PON1 (coded by *PON1*) as exposure and PD as outcome, the primary outcome of IVW model showed that the higher risk of PD was significantly associated with a decreased level of PON1, which indicated PON1 probably had protective effect on PD risk (OR[95%CI] = 0.81 [0.66, 0.99], *p* =0.04) (**Figure 3C**). But the causality was weaker in MR-PRESSO model (OR[95%CI] = 0.81 [0.66, 0.99], *p* =0.11) (**Figure 3C**). There was no evidence of heterogeneity in the IVW and MR-PRESSO analysis (**Supplementary Table 6**). The results of MR Egger and Weighted median were shown in **Supplementary Table 5**.

When we did the reverse analysis, we did not find any significant causality in all the models (**Figure 3D** and **Supplementary Table 5**). None of the models showed evidence of heterogeneity (**Supplementary Table 6**).

## 4 Discussion

The immune system’s dysregulation and inflammatory reactions are now more clearly linked to PD (30). However, due to their reliance on a small number of genes, restricted areas, or territories, and potential for analytical bias, the existing association studies are not sufficiently thorough. After a combination of quantitative analysis and two-sample MR analysis, we found that *TNF-α* rs1799964, *PON1* rs854560 and *CYP2D6* rs3892097 were associated with the higher risk of PD while *IL-6* rs1800795, *HLA-DRB* rs660895, *BST1* rs11931532 and *CCDC62* rs12817488 were related to the lower risk of PD. Besides, we observed that increased plasma level of ADP-ribosyl cyclase (coded by *BST1*) had causal effect on higher PD risk while PON1(coded by *PON1*) shown probably protective effect on PD risk. This study may help us to have a deeper understanding of the relationship between the inflammatory variations and PD, and potentially identify biomarkers and create anti-inflammatory and immunological therapy options for PD.

Our study is the most thorough one to date when compared to quantitative analyses on inflammatory genetic variations associated to PD. ZS Ulhaq conducted a meta-analysis in 2020 to clarify the relationship between inflammatory genes and PD. It discovered that while variations in *IL-1β, TNF-α* were not connected with PD risk, variations in *IL-1α, IL-6, IL-8, IL-10, and IL-18* were associated (156). However, we came to the conclusion that *TNF-α* was linked to an elevated risk of PD from our research. Although these findings were different from what ZS Ulhaq had previously stated, our study included the most recent original publications. Besides, genes of oxidative stress, neurotoxic and metabolism-related enzymes, and inflammatory polymorphism loci discovered by GWAS studies have also been extensively studied in the past and have been shown to impact the risk of PD. These genes were also included in our study. Further, for the genes with statistically significant results in the quantitative analysis, we explore the causality between proteins they encode and the risk of PD by conducting a two-sample MR analysis.

Regarding genes of cytokine specifically, several studies have noted higher levels of TNF-*α*, ILs, and other pro-inflammatory cytokines are presented in the peripheral blood, cerebrospinal fluid (CSF) of patients with PD and in the striatum of post-mortem brains from patients with PD (157–159).The inflammatory genes encoding these molecules have also been widely studies, though the results might be inconsistent. The gene set-association analysis did not reveal the association between *TNF-α, IL-6, IL-8* etc. and PD (144). But Chu et al. considered *TNF-α* rs1799964, *IL-6* rs1800795 and *IL-1*RA VNTR were shown to be associated with PD risk (160). Our study is a thorough update and addition to the prior studies because it used bigger cohorts. We discovered a favorable correlation between PD and a *TNF- α* rs1799964. The variation may alter the expression of TNF-*α* or have an impact on other genes associated with inflammation, contributing to the pathophysiology of PD (161, 162). We also found *IL-6* rs1800795 G>C decreased the PD risk, which may lower the level of IL-6 in serum (163). A MR analysis study also demonstrated that the pro-inflammatory activity of the IL-6 could be a determinant of prodromal PD (35).

In addition, PD pathogenesis is highly related to oxidative stress, which can promote the dysfunction of immune system and inflammatory response in turn (23). By altering the detoxification of neurotoxins in PD, metabolizing enzymes such CYPs (CYP2D6, CYP2E1) and paraoxonase (PON1) may impact the likelihood of developing PD. These enzymes’ activity and sensitivity to oxidative damage are strongly related (32, 164). In our quantitative analysis, *PON1* rs854560 was positively associated with PD risks in all three models (AM, DM and RM), which was inconsistent with the conclusion of previous meta-analysis conducted by Liu Y. et al. (165). Compared with Liu Y. et al., we included more studies to reach a more reliable conclusion. Besides, we also found *CYP2D6* rs3892097 significantly increased the risk of PD, which was consistent with previous meta-analysis conducted by Lu Y. et al. (166). CYP2D6 can catalyze the metabolism of MPTP to toxic 1-methyl-4-phenylpyridinium ion (MPP(+)), which lead to oxidative damage of dopaminergic neurons (167, 168). *CYP2D6* rs3892097 may affect the occurrence of PD by changing the metabolic activity of CYP2D6. GWAS has shown that genes including *BST1, HLA-DQB1* etc. involved in the “regulation of leucocyte/lymphocyte activity” and “cytokine-mediated signaling” are associated with PD risk (33). *HLA-DRA* and *HLA-DRB* alleles encode HLA-DR antigen, acting as regulatory molecule involved in autoimmunity (169). *HLA-DRB* variants differ in affinity with α-syn antigen epitopes, which influence antigen recognition and subsequent immune response (10). Patients with PD also have a higher expression of MHC class II molecules in peripheral blood mononuclear cells, which is consistent with the inflammatory pattern of PD (170). Therefore, variants in *HLA-DRB* could alter the risk of PD by regulating the expression of HLA-DRB or its response to α-syn (10, 15). *CCDC62/HIP1R* loci were identified by the first large-scale meta-analysis of published GWAS in PD (57). These researches are consistent with our research results that the variants in *BST1, HLA-DRB, CCDC62* were correlated with PD risk.

Further, we conducted a two-sample MR analysis to investigate the causality between proteins coded by inflammatory genes and the risk of PD and we found increased plasma level of ADP-ribosyl cyclase had causal effect on higher PD risk while PON1 shown probably protective effect on PD risk. Cyclic ADP-ribose (cADPR) is a signal transduction molecule downstream of the dopamine receptors, which is synthesized from β-NAD+ by both cytosolic and membrane-bound forms of ADP-ribosyl cyclase and/or CD38 (171, 172). Higashida, H. et al. indicated that cADPR, as an endogenous inhibitor of mTOR signaling pathway, reduced downstream protein synthesis and thus affected synaptic plasticity of neurons (173). The dysregulation of dopaminergic system is associated with a variety of neurological and psychiatric disorders, including PD (174). Increased ADP-ribosyl cyclase may lead to the disturbance of dopaminergic system by inhibiting mTOR signaling pathway, thus promoting the occurrence of PD. PON1 is an esterase carried by high-density lipoproteins and has antioxidant and anti-inflammatory effects (175). Its detoxification activity is considered to be an important link between environmental exposure to pesticides or pollutants and the risk of neurodegenerative diseases since it is able to hydrolyze active metabolites of organophosphate insecticides (176). The mutation at *PON1* rs854560 reduced the scavenging activity of lipoprotein free radicals, which may lead to neuronal damage (176). Thus, a decrease in plasma PON1 level would reduce the capacity of antioxidant and anti-inflammatory and increase the risk of PD. Reversely, our results suggested that the development of PD did not lead to the changes in plasma ADP-ribosyl cyclase and PON1 levels.

Our findings indicated that the onset and progression of PD is closely related to inflammatory response and the disorder of immune system. It would be worth mentioning that PD and atypical parkinsonian syndromes (APS), including multiple system atrophy (MSA) and progressive supranuclear palsy (PSP), have overlapping symptoms that are difficult to make an early diagnose. Nevertheless, PD and APS have different inflammatory patterns, which would be helpful in the differential diagnosis and understanding of the pathogenesis of diseases. Although the occurrence of PSP is also related to the activation of microglia and neuroinflammation, the process may be associated with the accumulation of phosphorylated tau protein, but not α-syn (177). Compared with patients with PD patients, patients with PSP have higher NLR in peripheral blood and significantly increased expression of CRP and microglia-derived cytokines in CSF, including IL-1β, IL-6 and TNF-α (177, 178). MSA is a rapidly progressing neurodegenerative disease characterized by the accumulation of oligodendrocyte inclusions composed of α‐syn (179). Animal model of MSA shows a stronger inflammatory response than PD model (180). Compared with the healthy controls, the PLR in the peripheral blood of MSA patients is significantly increased (13). The NLR and PLR in peripheral blood show no significant difference between patients with MSA and PD (13). However, patients with MSA have higher levels of inflammatory markers in the CSF than patients with PD, including CRP, serum amyloid A, IL-1β, IL-6 and TNF-α, but lower levels of neuroprotective molecules, such as beta nerve growth factor (β-NGF) and Delta and Notch like epidermal growth factor-related receptor (DNER) (178, 181, 182).

Excitingly, the existence of these inflammatory patterns and immune system alterations provides new insights into anti-inflammatory and immunological treatment strategies for neurodegenerative diseases, including PD. Firstly, inhibiting or activating the function of inflammatory genes might delay or halt disease progression during prodrome or prevent disease progression. For example, activation of *Nrf2* (the transcription factor of *NFE2L2*) could alleviate the progression of neurodegenerative diseases by counteracting oxidative stress and inflammation (183). Besides, treatments that have anti-inflammatory factors or enhances anti-inflammatory ability could reduce the occurrence and progression of neurodegenerative diseases. A retrospective cohort study indicated that anti-TNF therapy could effectively reduce the incidence of PD (184).Currently, several clinical trials of anti-inflammatory and immunological therapy for PD are underway, though no effective outcomes are available yet (5). Since the inflammatory cascade has an important impact on the development and progression of neurodegeneration in PD, the initiation of more clinical trials on PD inflammation is rational (5). Furthermore, targeting α-syn with antibodies to slow the transmission and reverse the effects of α-syn pathology is another direction for PD immunotherapy since α-syn plays a key role in the pathogenesis of PD. Monoclonal antibodies against α-syn could inhibit the spread of α-syn, reduce the loss of dopaminergic neurons, and alleviate motor deficits in PD mouse models (185, 186). Clinical trials have shown that PRX002/RG7935, the monoclonal antibody against α-syn, could penetrate BBB and efficiently reduce serum α-syn levels to alleviate the progression of PD (187, 188). So far, the safety and tolerability of the PRX002/RG7935 treatment have been preliminarily verified, and the next phase of clinical trials is needed to explore its effectiveness in the treatment of PD (188).

Nevertheless, it must be admitted that our study has several inescapable limitations. Because we combined all of the reported patients and controls for our quantitative analysis, these cases and controls may not be age or sex matched, which might lead to selection bias. Differences in race might also cause confusion. We were unable to run a subgroup analysis on the variables because of the dearth of data. Furthermore, barely fewer than 5 publications were included in some of our quantitative analysis. To reach a reliable conclusion, further unique investigations are required. Due to the insufficient GWAS data resources, we did not conduct causal analysis for all the proteins encoded by statistically significant genes, only ADP-ribosyl cyclase and PON1 were analyzed.

In conclusion, we included 18 inflammatory genes, including genes encoding cytokines, genes implicated in oxidative stress, genes for neurotoxins and metabolism-related enzymes, and inflammatory polymorphic loci discovered by GWAS analysis that are strongly connected with PD pathogenesis. While variations in *IL-1α, IL-1β, IL-10, MnSOD, NFE2L2, CYP2E1, NOS1, NAT2*, *ABCB1*, *HFE* or *MTHFR* were not connected to PD risk, we discovered that multiple polymorphisms from *IL-6, TNF-α, PON1, CYP2D6, HLA-DRB, BST1*, and *CCDC62* were statistically correlated with PD risk. Additionally, we indicated the changes in plasm ADP-ribosyl cyclase and PON1 level have causal effects on the risk of PD. Further researches are needed to confirm these findings.

## Data availability statement

The original contributions presented in the study are included in the article/**Supplementary Material**. Further inquiries can be directed to the corresponding author.

## Author contributions

Study design: MY and YZ. Data collection: MY, JL, SJ, BL, LS, ZH, and YZ. Data analysis: MY, JL, SJ, BL, LS, ZH, and YZ. Writing: MY, JL, SJ, BL, ZH, LS, and YZ. Funding: MY and YZ. Administration: MY and YZ. All authors contributed to the article and approved the submitted version.
