# Supplementary material for: Quantitative and causal analysis for inflammatory genes and the risk of Parkinson’s disease
Source: Front Immunol. 2023 Feb 28;14:1119315. doi: 10.3389/fimmu.2023.1119315 (PMC10011457; doi:10.3389/fimmu.2023.1119315)
Supplement: Supplementary file 1 [file DataSheet_1.pdf]

Supplementary material 1: Supplementary tables

Supplementary Table1: The search strategies for included genes in quantitative analysis.

gene	Search strategies
**1. Genes of cytokines**	
TNF-α	((TNF OR Tumor necrosis factor)) AND ((((((((SNP) OR polymorphism) OR variant) OR mutation) OR genetic) OR gene)) AND parkinson*)
IL-6, IL-1α, IL-1β, IL10	(((Interleukin) OR IL)) AND ((((((((SNP) OR polymorphism) OR variant) OR mutation) OR genetic) OR gene)) AND parkinson*)
**2. Genes involved in the oxidative stress**	
NOS1	((((NOS1) OR nitric oxide synthase 1) OR nNOS)) AND ((((((((SNP) OR polymorphism) OR variant) OR mutation) OR genetic) OR gene)) AND parkinson*)
MnSOD	((MnSOD or manganese-containing superoxide dismutase)) AND ((((((((SNP) OR polymorphism) OR variant) OR mutation) OR genetic) OR gene)) AND parkinson*)
NFE2L2	((NFE2L2 OR nuclear factor erythroid-derived 2-like 2)) AND ((((((((SNP) OR polymorphism) OR variant) OR mutation) OR genetic) OR gene)) AND parkinson*)
**3. Genes of neurotoxin-associated enzymes**	
CYP2D6	(CYP2D6 or cytochrome P450 2D6) AND ((((((((SNP) OR polymorphism) OR variant) OR mutation) OR genetic) OR gene)) AND parkinson*)
PON1	(((PON) OR paraoxonase)) AND ((((((((SNP) OR polymorphism) OR variant) OR mutation) OR genetic) OR gene)) AND parkinson*)
CYP2E1	(CYP2E1 or cytochrome P450 2E1) AND ((((((((SNP) OR polymorphism) OR variant) OR mutation) OR genetic) OR gene)) AND parkinson*)
NAT2	((NAT2 OR N-acetyl-transferase 2)) AND ((((((((SNP) OR polymorphism) OR variant) OR mutation) OR genetic) OR gene)) AND parkinson*)
ABCB1/MDR	((ABCB1 OR MDR1 or multidrug resistance protein 1)) AND ((((((((SNP) OR polymorphism) OR variant) OR mutation) OR genetic) OR gene)) AND parkinson*)
**4. Genes of metabolism-associated enzymes**	
HFE	(HFE or hemochromatosis) AND ((((((((SNP) OR polymorphism) OR variant) OR mutation) OR genetic) OR gene)) AND parkinson*)
MTHFR	((MTHFR OR methylenetetrahydrofolate reductase)) AND ((((((((SNP) OR polymorphism) OR variant) OR mutation) OR genetic) OR gene)) AND parkinson*)
**5. Inflammatory polymorphic locus identified by genome-wide association studies (GWAS) study**	

BST1	(((BST1) OR bone marrow stromal cell antigen 1)) AND ((((((((((((SNP) OR polymorphism) OR variant) OR mutation) OR genetic) OR gene)) AND parkinson*)))))
HLA-DRB	(((human leukocyte antigen) OR HLA)) AND ((((((((SNP) OR polymorphism) OR variant) OR mutation) OR genetic) OR gene)) AND parkinson*)
CCDC62	(((CCDC62) OR HIP1R)) AND (((((((SNP OR polymorphism OR variant OR mutation OR genetic OR gene)) AND parkinson*))))

Supplementary Table2. The distributions of genotypes and alleles for variants included in quantitative analysis

Gene	Variants	A/a	Year	First Author	AA/Aa/aa		Allele(A/a)	
					cases	controls	cases	controls
HFE	rs1800562	G/A	2016	Mariani, S.	82/3/0	101/5/0	167/3	207/5
			2013	Mariani, S.	70/3/0	137/2/0	143/3	276/2
			2011	Greco, V.	178/3/0	177/3/0	359/3	357/3
			2008	Halling, J.	67/12/0	125/26/2	146/12	276/30
			2007	Aamodt, A. H.	330/56/2	428/75/2	716/60	931/79
			2006	Guerreiro, R. J.	114/18/0	110/5/0	246/18	225/5
			2003	Dekker, M. C.	176/19/2	2567/339/8	371/23	5473/355
			2002	Borie, C.	66/5/0	52/5/0	137/5	109/5
			2002	Buchanan, D. D.	391/46/1	405/76/4	828/48	886/84
HFE	rs1799945	C/G	2016	Mariani, S.	64/32/1	56/30/0	160/34	142/30
			2013	Mariani, S.	53/24/0	99/38/2	130/24	236/42
			2011	Greco, V.	125/49/7	141/36/3	299/63	318/42
			2008	Halling, J.	54/22/3	110/38/5	130/28	258/48
			2007	Aamodt, A. H.	315/66/7	396/102/7	696/80	894/116
			2006	Guerreiro, R. J.	89/38/5	74/39/2	216/48	187/43
			2003	Dekker, M. C.	145/50/2	2136/710/68	340/54	4982/846
			2002	Borie, C.	42/23/1	39/20/0	107/25	98/20
HLA-DRB1	rs660895	A/G	2020	Chang, K. H.	271/185/30	251/182/40	727/245	684/262
			2017	Chuang, Y. H. (i)	974/510/63	929/589/77	2458/636	2447/743
			2017	Chuang, Y. H. (ii)	377/126/6	753/339/36	880/138	1845/411
			2012	Ahmed, I.	370/123/6	751/334/37	863/135	1836/408
MTHFR	rs1801133	C/T	2016	Zahra, C.	66/68/17	136/134/41	200/102	406/216
			2014	Kumudini, N.	122/27/2	355/60/1	271/31	770/62
			2014	Liao, Q.	321/347/97	231/390/96	989/541	852/582
			2012	Gorgone, G.	15/23/22	31/36/15	53/67	98/66
			2011	Fong, C. S.	116/78/17	126/77/15	310/112	329/107
			2009	Camicioli, R. M.	20/26/5	17/23/9	66/36	57/41
			2009	Rodriguez-Oroz, M. C.	33/41/3	9/13/6	107/47	31/25
			2009	Yuan, R. Y.	31/37/8	65/38/7	99/53	168/52
			2007	Caccamo, D.	12/21/16	34/37/15	45/53	105/67
			2006	Religa, D.	48/56/10	55/38/7	152/76	148/52
			2006	Todorovic, Z.	47/51/15	16/28/9	145/81	60/46
			2005	Wullner, U.	150/154/38	168/130/44	454/230	466/218
			2000	Yasui, K.	29/40/21	21/21/11	98/82	63/43
MTHFR	rs1801131	A/C	2009	Camicioli, R. M.	24/20/6	28/18/3	68/32	74/24
			2009	Rodriguez-Oroz, M. C.	33/39/10	15/9/2	105/59	39/13
			2009	Yuan, R. Y.	50/21/5	70/32/8	121/31	172/48
			2005	Wullner, U.	171/140/31	157/137/48	482/202	451/233
MDR1/ABCB1	rs1128503	C/T	2009	Funke, C.	92/153/55	110/147/45	337/263	367/237
			2009	Westerlund, M.	87/148/40	89/119/63	322/228	297/245
			2005	Tan, E. K.	29/86/70	32/85/89	144/226	149/263
			2004	Tan, E. K.	56/77/25	49/65/25	189/127	163/115
MDR1/ABCB1	rs1045642	C/T	2015	Narayan, S.	62/128/95	122/308/141	252/318	552/590
			2013	Kiyohara, C.	75/114/49	138/166/64	264/212	442/294
			2010	Dutheil, F.	45/112/50	120/231/131	202/212	471/493
			2009	Funke, C.	67/147/86	76/147/79	281/319	299/305

			2009	Westerlund, M.	53/141/83	56/140/96	247/307	252/332
			2009	Zschiedrich, K. (i)	69/126/70	21/73/29	264/266	115/131
			2009	Zschiedrich, K. (ii)	12/18/12	15/28/18	42/42	58/64
			2005	Tan, E. K.	81/77/27	87/95/24	239/131	269/143
			2004	Tan, E. K.	35/78/45	30/73/36	148/168	133/145
			2003	Drozdzik, M.	26/66/15	24/58/21	118/96	106/100
			2002	Furuno, T.	20/50/25	28/58/20	90/100	114/98
MDR1/ABCB1	rs2032582	T/G(A)	2015	Narayan, S.	68/118/95	104/278/186	254/308	486/650
			2010	Dutheil, F.	39/100/68	88/236/158	178/236	412/552
			2009	Funke, C.	57/150/93	60/127/112	264/336	247/351
			2009	Westerlund, M.	40/143/90	62/132/97	223/323	256/326
			2005	Tan, E. K.	25/93/67	27/96/83	143/227	150/262
			2004	Tan, E. K.	26/77/55	24/67/48	129/187	115/163
			2002	Furuno, T.	21/43/31	21/45/40	85/105	87/125
CYP2D6	rs3892097	G/A	2010	Singh, M.	54/22/1	103/19/3	130/24	225/25
			2007	Halling, J.	35/31/13	58/71/24	101/57	187/119
			2004	Elbaz, A.	122/58/10	280/123/16	302/78	683/155
			2000	Woo, S. I.	89/4/0	121/1/0	182/4	243/1
			1999	Atkinson, A.	17/14/2	58/14/3	48/18	130/20
			1999	Nicholl, D. J.	130/64/12	137/56/12	324/88	330/80
			1999	Payami, H.	374/173/29	160/71/16	921/231	391/103
			1996	Bordet, R.	72/31/2	66/37/2	175/35	169/41
			1996	Diederich, N.	52/25/3	71/28/7	129/31	170/42
			1996	Gasser, T.	74/34/7	47/23/3	182/48	117/29
			1996	Lucotte, G.	29/13/5	38/8/1	71/23	84/10
			1995	Chen, X.	27/0/1	185/24/3	54/2	394/30
			1994	Plante-Bordeneuve, V.	26/20/2	62/20/6	72/24	144/32
			1993	Kurth, M. C.	22/23/5	72/32/6	67/33	176/44
CYP2D6	A2637	A/-	2007	Halling, J.	78/1/0	153/0/0	157/1	306/0
			1999	Nicholl, D. J.	199/7/0	197/8/0	405/7	402/8
			1996	Bordet, R.	100/5/0	98/7/0	205/5	203/7
			1996	Lucotte, G.	46/1/0	47/0/0	93/1	94/0
			1994	Plante-Bordeneuve, V.	45/3/0	86/2/0	93/3	174/2
NAT2	rs1799929	C/T	2010	Singh, M.	38/35/4	71/47/7	111/43	189/61
			2006	Borlak, J.	44/55/25	69/120/54	143/105	258/228
			1999	Nicholl, D. J.	59/107/39	67/91/47	225/185	225/185
NAT2	rs1799930	G/A	2010	Singh, M.	33/36/8	58/55/12	102/52	171/79
			2006	Borlak, J.	115/8/1	225/18/0	238/10	468/18
			1999	Nicholl, D. J.	111/76/17	110/77/18	298/110	297/113
BST1	rs11724635	C/A	2015	Chang, K. H.	183/296/117	197/296/104	662/530	690/504
			2014	Chen, M. L.	174/217/77	178/226/83	565/371	582/392
			2012	Miyake, Y.	84/102/43	148/158/51	270/188	454/260
BST1	rs11931532	T/C	2015	Guo, J. F.	242/576/201	243/550/237	1060/978	1036/1024
			2012	Miyake, Y.	54/101/74	72/158/127	209/249	302/412
			2010	Chang, X. L.	158/336/126	110/271/123	652/588	491/517
CCDC62	rs12817488	A/G	2015	Yu, R. L.	164/249/94	133/247/138	577/437	513/523
			2014	Liu, R. R.	121/154/66	108/216/99	396/286	432/414
			2013	Li, N. N.	234/366/160	200/330/178	834/686	730/686
IL-1α	rs1800587	C/T	2008	Infante, J.	102/78/15	97/61/12	282/108	255/85

			2008	Zhou, Y. T.	451/79/3	422/103/5	981/85	947/113
			2007	Wu, Y. R. (1)	417/73/3	334/52/2	907/79	720/56
			2004	Moller, J. C.	83/84/9	79/86/5	250/102	244/96
			2002	Mattila, K. M.	28/20/4	33/29/11	76/28	95/51
			2002	McGeer, P. L.	36/51/13	37/60/3	123/77	134/66
			2002	Schulte, T.	125/107/25	141/93/26	357/157	375/145
			2001	Dodel, R. C.	108/86/7	115/77/5	302/100	307/87
			2000	Nishimura, M.	97/24/1	87/25/0	218/26	199/25
IL-1β	rs 16944	C/T	2011	Pascale, E.	65/55/26	56/70/30	185/107	182/130
			2007	Wahner, A. D.	106/135/48	118/126/25	347/231	362/176
			2007	Wu, Y. R. (1)	146/257/90	105/195/88	549/437	405/371
			2005	Nishimura, M.	91/181/89	69/128/60	363/359	266/248
			2002	Mattila, K. M.	25/25/2	24/30/19	75/29	78/68
			2002	McGeer, P. L.	33/45/22	44/53/3	111/89	141/59
			2002	Schulte, T.	110/141/44	127/105/35	361/229	359/175
			2000	Nishimura, M.	35/55/31	31/58/23	125/117	120/104
IL-6	rs1800795	G/C	2012	San Luciano, M.	205/144/31	208/245/69	554/206	661/383
			2008	Infante, J.	88/81/27	62/81/27	257/135	205/135
			2005	Hakansson, A. (2)	78/129/51	68/162/78	285/231	298/318
			2004	Ross, O. A.	26/44/20	32/50/11	96/84	114/72
IL-10	rs1800896	A/G	2013	Nie, K.	266/34/2	268/26/0	566/38	562/26
			2011	Pascale, E.	51/76/19	59/70/27	178/114	188/124
			2008	Bialecka, M.	105/144/67	101/137/62	354/278	339/261
			2008	Infante, J.	32/95/66	32/81/55	159/227	145/191
			2007	Bialecka, M.	112/168/61	99/153/63	392/290	351/279
			2005	Hakansson, A. (1)	76/124/59	87/144/76	276/242	318/296
IL-10	rs1800871	C/A	2016	Liu, Z.	51/169/240	47/211/215	271/649	305/641
			2012	Li, D.	40/153/162	28/79/93	233/477	135/265
			2008	Bialecka, M.	185/104/27	170/108/22	474/158	448/152
			2007	Bialecka, M.	185/124/32	190/100/25	494/188	480/150
PON1	rs705379	C/T	2013	Lee, P. C.	77/121/71	104/187/96	275/263	395/379
			2012	Belin, A. C.	130/247/126	141/253/149	507/499	535/551
			2003	Kelada, S. N.	43/67/26	71/102/55	153/119	244/212
PON1	rs854560	T/A	2019	Mota, A.	24/21/25	36/31/8	69/71	103/47
			2013	Lee, P. C.	120/125/42	195/196/49	365/209	586/294
			2012	Belin, A. C.	178/238/78	199/241/72	594/394	639/385
			2011	Punia, S.	317/144/26	319/137/18	778/196	775/173
			2010	Manthripragada, A. D.(i)	118/122/42	133/128/29	358/206	394/186
			2010	Manthripragada, A. D.(ii)	159/144/48	180/148/35	462/240	508/218
			2005	Fong, C. S.	120/4/1	151/10/1	244/6	312/12
			2004	Clarimon, J.	58/68/12	58/59/13	184/92	175/85
			2003	Kelada, S. N.	60/70/20	105/104/34	190/110	314/172
			2002	Carmine, A.	31/52/17	49/39/12	114/86	137/63
			2001	Akhmedova, S. N.	36/61/20	102/80/25	133/101	284/130
			2000	Wang, J. (1)	165/14/1	166/13/1	344/16	345/15
PON1	rs662	A/G	2019	Mota, A.	33/32/5	35/32/8	98/42	102/48
			2013	Lee, P. C.	144/110/27	180/163/54	398/164	523/271

			2012	Belin, A. C.	282/197/32	308/209/27	761/261	825/263
			2011	Punia, S.	188/226/73	176/225/73	602/372	577/371
			2004	Clarimon, J.	67/59/18	65/55/15	193/95	185/85
			2003	Kelada, S. N.	81/57/12	121/97/24	219/81	339/145
			2000	Taylor, M. C.	42/45/5	63/46/13	129/55	172/72
			2000	Wang, J. (1)	25/85/70	26/88/66	135/225	140/220
			1999	Akhmedova, S.	65/51/5	63/48/6	181/61	174/60
			1998	Kondo, I.	13/70/86	34/124/94	96/242	192/312
TNF-α	rs1800629	G/A	2018	Agliardi, C.	269/80/5	350/88/5	618/90	788/98
			2011	Pascale, E.	115/25/6	109/45/2	255/37	263/49
			2007	Wahner, A. D.	205/71/13	201/62/6	481/97	464/74
			2007	Wu, Y. R. (2)	282/84/3	262/61/3	648/90	585/67
			2004	Ross, O. A.	60/20/10	54/31/8	140/40	139/47
			2000	Kruger, R.	153/79/5	127/43/7	385/89	297/57
TNF-α	rs1799964	T/C	2008	Infante, J.	174/17/3	147/22/1	365/23	316/24
			2007	Wu, Y. R. (2)	260/82/27	228/90/8	602/136	546/106
			2001	Nishimura, M.	112/49/11	120/34/3	273/71	274/40
NFE2L2	rs6706649	G/A	2017	Ran, C.	385/88/7	387/90/7	858/102	864/104
			2016	Gui, Y.	582/138/15	405/76/7	1302/168	886/90
			2013	Chen, Y. C.	434/45/1	461/64/1	913/47	986/66
			2010	von Otter, M. (i)	125/33/4	136/45/3	283/41	317/51
			2010	von Otter, M. (ii)	150/35/6	143/42/2	335/47	328/46
NFE2L2	rs6721961	C/A	2017	Ran, C.	370/102/6	365/120/3	842/114	850/126
			2016	Gui, Y.	528/147/90	334/99/55	1203/327	767/209
			2013	Chen, Y. C.	243/202/35	274/207/45	688/272	755/297
			2010	von Otter, M. (i)	122/39/1	156/27/1	283/41	339/29
			2010	von Otter, M. (ii)	147/38/6	142/43/2	332/50	327/47
NFE2L2	rs35652124	A/G	2017	Ran, C.	215/217/46	230/210/42	647/309	670/294
			2016	Gui, Y.	365/268/132	235/166/88	998/532	636/342
			2013	Chen, Y. C.	119/234/127	114/285/127	472/488	513/539
			2010	von Otter, M. (i)	89/58/15	82/89/13	236/88	253/115
			2010	von Otter, M. (ii)	88/77/26	103/67/17	253/129	273/101
NFE2L2	rs2706110	G/A	2016	Gui, Y.	466/233/63	320/121/47	1165/359	761/215
			2015	Todorovic, M.	874/372/50	860/427/53	2120/472	2147/533
			2010	von Otter, M. (i)	110/48/6	146/40/4	268/60	332/48
			2010	von Otter, M. (ii)	125/45/7	119/53/1	295/59	291/55
NFE2L2	rs10183914	G/A	2016	Gui, Y.	431/228/105	290/136/63	1090/438	716/262
			2015	Todorovic, M.	556/573/161	547/615/182	1685/895	1709/979
			2010	von Otter, M. (i)	70/81/14	76/89/25	221/109	241/139
			2010	von Otter, M. (ii)	93/73/20	86/73/28	259/113	245/129
NFE2L2	rs1806649	G/A	2016	Gui, Y.	621/117/24	400/77/12	1359/165	877/101
			2015	Todorovic, M.	727/476/96	738/509/92	1930/668	1985/693
			2010	von Otter, M. (i)	86/65/14	95/80/15	237/93	270/110
			2010	von Otter, M. (ii)	117/60/9	97/61/23	294/78	255/107
NFE2L2	rs2001350	A/G	2017	Ran, C.	387/95/5	388/93/1	869/105	869/95
			2015	Todorovic, M.	1077/212/10	1121/212/15	2366/232	2454/242
			2010	von Otter, M. (i)	129/35/1	167/23/0	293/37	357/23
			2010	von Otter, M. (ii)	158/28/4	148/39/0	344/36	335/39
NOS1	rs2682826	C/T	2016	Gupta, S. P.	40/38/11	192/118/22	118/60	502/162

			2016	Paul, K. C.	178/154/24	231/198/26	510/202	660/250
			2007	Huerta, C.	248/170/32	110/74/16	666/234	294/106
			2004	Hague, S.	78/57/7	59/63/9	213/71	181/81
			2003	Levecque, C.	94/90/25	264/185/39	278/140	713/263
NOS1	rs1060826	G/A	2016	Paul, K. C.	129/170/57	179/204/46	428/284	562/296
			2007	Huerta, C.	194/202/54	88/92/20	590/310	268/132
			2004	Hague, S.	51/72/20	50/52/33	174/112	152/118
CYP2E1	rs2031920	C/T	2008	Singh, M.	65/5/0	99/1/0	135/5	199/1
			2002	Wu, R. M.	135/88/11	144/97/10	358/110	385/117
			2000	Wang, J. (2)	101/44/5	109/38/3	246/54	256/44
MnSOD	rs4880	T/C	2010	Wang, V. C.	229/60/6	92/19/0	518/72	203/19
			2008	Singh, M.	15/42/13	16/73/11	72/68	105/95
			1999	Grasbon-Frodl, E. M.	7/26/11	6/25/11	40/48	37/47

Note: (1), (2) represent different articles with the same publication year and first authors. (i), (ii), (iii) represent different cohorts from the same paper.

Supplementary Table3. The distributions of allele frequency (AF) for variants included in quantitative analysis

Gene	Variant	Sample size#	Allele (Ref/Alt)	Allele frequency (AF) AF(P)	AF(C)	AF(G)
**Genes of cytokines**						
TNF-α	rs1800629	6/1485/1464	G/A	0.15	0.13	0.14
TNF-α	rs1799964	3/735/653	T/C	0.16	0.13	0.19
IL-1α	rs1800587	9/2129/2000	C/T	0.18	0.18	0.28
IL-1β	rs16944	8/1857/1622	C/T	0.43	0.41	0.42
IL-6	rs1800795	4/924/1093	G/C	0.35	0.42	0.29
IL-10	rs1800896	6/1557/1540	A/G	0.38	0.38	0.41
IL-10	rs1800871	4/1472/1288	C/A	0.50	0.47	0.43
**Genes involved in the oxidative stress**						
NOS1	rs2682826	5/1246/1606	C/T	0.28	0.27	0.25
NOS1	rs1060826	3/949/764	G/A	0.37	0.36	0.33
MnSOD	rs4880	3/409/253	T/C	0.23	0.32	0.41
NFE2L2	rs6706649	4/2048/1869	G/A	0.10	0.10	0.09
NFE2L2	rs6721961	4/2076/1873	C/A	0.19	0.19	0.15
NFE2L2	rs35652124	4/2076/1868	A/G	0.37	0.37	0.38
NFE2L2	rs2706110	3/2399/2191	G/A	0.20	0.19	0.32
NFE2L2	rs10183914	3/2405/2210	G/A	0.32	0.34	0.33
NFE2L2	rs1806649	3/2412/2199	G/A	0.21	0.23	0.17
NFE2L2	rs2001350	3/2141/2207	A/G	0.10	0.09	0.11
**Genes of neurotoxin-associated enzymes**						
CYP2D6	rs3892097	14/1727/2087	G/A	0.20	0.18	0.14
CYP2D6	A2637	5/485/598	A/-	0.02	0.01	NA
PON1	rs705379	3/908/1158	C/T	0.49	0.49	0.35
PON1	rs854560	11/2781/3176	T/A	0.31	0.28	0.29
PON1	rs662	10/2205/2538	A/G	0.36	0.36	0.42
CYP2E1	rs2031920	3/454/501	C/T	0.19	0.16	0.03
NAT2	rs1799929	3/406/573	C/T	0.41	0.41	0.36
NAT2	rs1799930	3/405/573	G/A	0.21	0.18	0.26
MDR1/ABCB1	rs1128503	4/918/918	C/T	0.46	0.47	0.37
MDR1/ABCB1	rs1045642	10/2159/2753	C/T	0.50	0.49	0.42
MDR1/ABCB1	rs2032582	7/1499/2091	T/G(A)	0.57	0.58	0.33
**Metabolism associated enzymes**						
HFE	rs1800562	9/1644/4654	G/A	0.05	0.06	0.04
HFE	rs1799945	8/1217/4151	C/G	0.15	0.14	0.10
MTHFR	rs1801133	13/2250/2565	C/T	0.33	0.31	0.27
MTHFR	rs1801131	4/550/527	A/C	0.31	0.30	0.26
**Inflammatory polymorphic locus identified by genome-wide association studies (GWAS) study**						
BST1	rs11724635	3/1293/1441	C/A	0.42	0.40	0.41
BST1	rs11931532	3/1868/3782	T/C	0.49	0.52	0.13
HLA-DRB1	rs660895	3/3041/4318	A/G	0.19	0.21	0.20
CCDC62	rs12817488	3/1608/1649	A/G	0.44	0.49	0.61

Note: # Number of articles/patients/controls included for quantitative analysis. The Allele (Ref/Alt) represents refer allele and alter allele, respectively. Allele frequency (AF) included data from patients (P) and controls (C) used for quantitative analysis, as well as data from databases (G) including GnomAD and 1000Genomes

Supplementary Table4. The results of sensitivity analysis of variants included in quantitative analysis

(1) *TNF-α* rs1800629

Reference (remove)	NO. of cases/controls	AM		DM		RM	
		OR	p	OR	p	OR	p
2000Kruger, R.	1248/1287	1.10 [0.94, 1.30]	0.24	1.07 [0.89, 1.28]	0.49	1.61 [0.95, 2.73]	0.08
2004Ross, O. A.	1395/1371	1.15 [0.99, 1.34]	0.08	1.16 [0.97, 1.38]	0.10	1.61 [0.95, 2.73]	0.30
2007Wahner, A. D.	1196/1195	1.08 [0.92, 1.28]	0.34	1.09 [0.90, 1.31]	0.37	1.15 [0.67, 1.98]	0.62
2007Wu, Y. R. (2)	1116/1138	1.10 [0.93, 1.29]	0.27	1.07 [0.89, 1.30]	0.45	1.38 [0.84, 2.26]	0.20
2011Pascale, E.	1339/1308	1.17 [1.00, 1.36]	0.05	1.19 [1.00, 1.42]	0.05	1.20 [0.73, 1.97]	0.48
2018Agliardi, C.	1131/1021	1.10 [0.93, 1.31]	0.26	1.09 [0.90, 1.32]	0.39	1.34 [0.80, 2.23]	0.26
All Combination	1485/1464	1.12 [0.96, 1.30]	0.14	1.11 [0.94, 1.31]	0.21	1.33 [0.83, 2.13]	0.24

(2) *TNF-α* rs1799964

Reference (remove)	NO. of cases/controls	AM		DM		RM	
		OR	p	OR	p	OR	p
2001Nishimura, M.	563/496	1.09 [0.84, 1.42]	0.51	0.92 [0.69, 1.23]	0.57	3.08 [1.44, 6.57]	0.004
2007Wu, Y. R. (2)	366/327	1.25 [0.59, 2.64]	0.56	1.16 [0.50, 2.69]	0.73	3.28 [1.07, 10.11]	0.04
2008Infante, J.	541/483	1.40 [0.92, 2.11]	0.11	1.26 [0.72, 2.22]	0.41	3.24 [1.64, 6.41]	0.0007
All Combination	735/653	1.24 [0.85, 1.79]	0.26	1.10 [0.70, 1.72]	0.69	3.19 [1.66, 6.13]	0.0005

(3) *IL-1α* rs1800587

Reference (remove)	NO. of cases/controls	AM		DM		RM	
		OR	p	OR	p	OR	p
2000Nishimura, M.	2007/1888	1.03 [0.92, 1.16]	0.61	1.02 [0.88, 1.18]	0.80	1.16 [0.83, 1.62]	0.39
2001Dodel, R. C.	1928/1803	1.01 [0.89, 1.14]	0.89	0.99 [0.85, 1.15]	0.86	1.15 [0.81, 1.64]	0.42
2002Mattila, K. M.	2077/1927	1.05 [0.93, 1.18]	0.44	1.03 [0.89, 1.18]	0.72	1.28 [0.90, 1.82]	0.17
2002McGeer, P. L.	2029/1900	1.01 [0.89, 1.14]	0.88	1.01 [0.87, 1.17]	0.89	1.01 [0.71, 1.45]	0.94
2002Schulte, T.	1872/1740	1.00 [0.88, 1.14]	0.95	0.97 [0.83, 1.13]	0.70	1.29 [0.85, 1.94]	0.23
2004Moller, J. C.	1953/1830	1.03 [0.91, 1.16]	0.68	1.02 [0.88, 1.18]	0.82	1.12 [0.79, 1.59]	0.52
2007Wu, Y. R. (1)	1636/1612	1.02 [0.90, 1.15]	0.78	0.99 [0.86, 1.16]	0.95	1.17 [0.83, 1.65]	0.36
2008Infante, J.	1934/1830	1.01 [0.89, 1.15]	0.85	0.99 [0.85, 1.15]	0.88	1.19 [0.82, 1.72]	0.36
2008Zhou, Y. T.	1596/1470	1.10 [0.97, 1.25]	0.15	1.10 [0.94, 1.29]	0.21	0.59 [0.14, 2.50]	0.26
All Combination	2129/2000	1.03 [0.91, 1.16]	0.64	1.01 [0.88, 1.16]	0.87	1.17 [0.84, 1.64]	0.35

(4) *IL-1β* rs16944

Reference (remove)	NO. of cases/controls	AM		DM		RM	
		OR	p	OR	p	OR	p
2000Nishimura, M.	1736/1510	1.05 [0.82, 1.34]	0.71	1.07 [0.83, 1.38]	0.63	1.13 [0.71, 1.80]	0.60
2002Mattila, K. M.	1805/1549	1.13 [0.94, 1.38]	0.20	1.11 [0.89, 1.38]	0.35	1.28 [0.89, 1.85]	0.18
2002McGeer, P. L.	1757/1522	0.99 [0.80, 1.22]	0.91	1.01 [0.79, 1.29]	0.93	1.02 [0.72, 1.44]	0.91
2002Schulte, T.	1562/1355	1.02 [0.80, 1.30]	0.90	0.99 [0.78, 1.25]	0.93	1.16 [0.71, 1.88]	0.56
2005Nishimura, M.	1496/1365	1.05 [0.81, 1.36]	0.73	1.04 [0.79, 1.37]	0.76	1.17 [0.70, 1.95]	0.54
2007Wahner, A. D.	1568/1353	1.01 [0.80, 1.28]	0.94	1.01 [0.78, 1.31]	0.96	1.06 [0.69, 1.63]	0.79
2007Wu, Y. R. (1)	1364/1234	1.09 [0.85, 1.39]	0.50	1.09 [0.84, 1.42]	0.52	1.25 [0.78, 2.01]	0.35
2011Pascale, E.	1711/1466	1.09 [0.86, 1.38]	0.46	1.12 [0.89, 1.41]	0.34	1.20 [0.75, 1.92]	0.45
All Combination	1857/1622	1.05 [0.85, 1.31]	0.64	1.06 [0.84, 1.33]	0.65	1.16 [0.77, 1.74]	0.49

(5) *IL-6* rs1800795

Reference (remove)	NO. of cases/controls	AM		DM		RM	
		OR	p	OR	p	OR	p
2004Ross, O. A.	834/1000	0.71 [0.62, 0.82]	<0.00001	0.62 [0.51, 0.75]	<0.00001	0.69 [0.53, 0.90]	0.007
2005Hakansson, A. (2)	666/785	0.86 [0.58, 1.28]	0.46	0.66 [0.53, 0.81]	0.0001	0.95 [0.48, 1.86]	0.88
2008Infante, J.	728/923	0.84 [0.59, 1.20]	0.34	0.65 [0.53, 0.79]	<0.0001	0.88 [0.49, 1.58]	0.66
2012San Luciano, M.	544/571	0.91 [0.66, 1.25]	0.55	0.75 [0.58, 0.97]	0.03	1.00 [0.58, 1.74]	0.99
All Combination	924/1093	0.82 [0.63, 1.06]	0.13	0.66 [0.55, 0.79]	<0.00001	0.85 [0.55, 1.30]	0.45

(6) *IL-10* rs1800896

Reference (remove)	NO. of cases/controls	AM		DM		RM	
		OR	p	OR	p	OR	p
2005Hakansson, A. (1)	1298/1233	1.02 [0.90, 1.15]	0.81	1.07 [0.89, 1.28]	0.49	0.96 [0.77, 1.19]	0.69
2007Bialecka, M.	1216/1225	1.02 [0.90, 1.16]	0.73	1.08 [0.89, 1.30]	0.43	0.97 [0.78, 1.20]	0.75
2008Bialecka, M.	1241/1240	0.99 [0.88, 1.12]	0.90	1.05 [0.87, 1.27]	0.62	0.92 [0.74, 1.14]	0.43
2008Infante, J.	1364/1372	0.99 [0.88, 1.11]	0.81	1.03 [0.87, 1.22]	0.75	0.92 [0.74, 1.13]	0.41
2011Pascale, E.	1411/1384	1.00 [0.89, 1.13]	0.97	1.03 [0.87, 1.23]	0.74	0.97 [0.79, 1.18]	0.75
2013Nie, K.	1255/1246	0.98 [0.88, 1.10]	0.73	1.01 [0.85, 1.20]	0.91	0.93 [0.77, 1.13]	0.48
All Combination	1557/1540	1.00 [0.90, 1.11]	0.98	1.04 [0.88, 1.23]	0.62	0.94 [0.78, 1.14]	0.54

(7) *IL-10* rs1800871

Reference (remove)	NO. of cases/controls	AM		DM		RM	
		OR	p	OR	p	OR	p
2007Bialecka, M.	1131/973	1.07 [0.94, 1.22]	0.33	0.97 [0.77, 1.22]	0.81	1.18 [0.97, 1.43]	0.10
2008Bialecka, M.	1156/988	1.13 [0.99, 1.30]	0.06	1.15 [0.92, 1.44]	0.22	1.18 [0.97, 1.43]	0.10
2012Li, D.	1117/1088	1.12 [0.98, 1.27]	0.10	1.04 [0.86, 1.27]	0.66	1.27 [1.03, 1.58]	0.03
2016Liu, Z.	1012/815	1.08 [0.93, 1.25]	0.30	1.12 [0.91, 1.38]	0.27	1.06 [0.81, 1.37]	0.68
All Combination	1472/1288	1.10 [0.98, 1.24]	0.11	1.07 [0.89, 1.29]	0.46	1.18 [0.98, 1.42]	0.08

(8) *NOS1* rs2682826

Reference (remove)	NO. of cases/controls	AM		DM		RM	
		OR	p	OR	p	OR	p
2003Levecque, C.	1037/1118	1.05 [0.82, 1.34]	0.73	1.04 [0.77, 1.39]	0.82	1.12 [0.79, 1.59]	0.53
2004Hague, S.	1104/1475	1.19 [0.97, 1.46]	0.09	1.21 [0.96, 1.53]	0.10	1.30 [0.96, 1.77]	0.09
2007Huerta, C.	796/1406	1.15 [0.87, 1.51]	0.33	1.15 [0.82, 1.61]	0.43	1.36 [0.98, 1.89]	0.07
2016Gupta, S. P.	1157/1274	1.04 [0.83, 1.29]	0.75	1.03 [0.79, 1.35]	0.81	1.15 [0.84, 1.58]	0.39
2016Paul, K. C.	890/1151	1.13 [0.84, 1.52]	0.44	1.14 [0.80, 1.63]	0.48	1.25 [0.89, 1.76]	0.20
All Combination	1246/1606	1.11 [0.89, 1.38]	0.36	1.11 [0.86, 1.45]	0.42	1.23 [0.92, 1.66]	0.16

(9) *NOS1* rs1060826

Reference (remove)	NO. of cases/controls	AM		DM		RM	
		OR	p	OR	p	OR	p
2004Hague, S.	806/629	1.18 [1.00, 1.38]	0.05	1.16 [0.93, 1.44]	0.18	1.44 [1.04, 2.01]	0.03
2007Huerta, C.	499/564	1.05 [0.70, 1.57]	0.83	1.21 [0.94, 1.55]	0.14	0.91 [0.30, 2.82]	0.88
2016Paul, K. C.	593/335	0.97 [0.76, 1.23]	0.78	1.04 [0.79, 1.38]	0.76	0.79 [0.33, 1.91]	0.61
All Combination	949/764	1.07 [0.86, 1.34]	0.55	1.14 [0.94, 1.40]	0.19	1.02 [0.53, 1.96]	0.95

(10) *MnSOD* rs4880

Reference (remove)	NO. of cases/controls	AM		DM		RM	
		OR	p	OR	p	OR	p
1999Grasbon-Frodl, E. M.	365/211	1.21 [0.87, 1.69]	0.26	1.11 [0.71, 1.74]	0.65	0.94 [0.36, 2.48]	0.07
2008Singh, M.	339/153	1.23 [0.83, 1.82]	0.30	1.29 [0.77, 2.13]	0.33	1.25 [0.52, 3.01]	0.61
2010Wang, V. C.	114 142	1.01 [0.71, 1.43]	0.96	0.75 [0.39, 1.44]	0.39	1.36 [0.72, 2.59]	0.35
All Combination	409/253	1.14 [0.86, 1.53]	0.37	1.08 [0.71, 1.64]	0.72	1.52 [0.82, 2.82]	0.19

(11) *NFE2L2* rs6706649

Reference (remove)	NO. of cases/controls	AM		DM		RM	
		OR	p	OR	p	OR	p
2010von Otter, M. (i)	1886/1685	1.04 [0.88, 1.22]	0.65	1.01 [0.85, 1.21]	0.88	1.42 [0.78, 2.61]	0.25
2010von Otter, M. (ii)	1857/1682	1.02 [0.87, 1.20]	0.78	1.01 [0.84, 1.20]	0.94	1.42 [0.78, 2.61]	0.43
2013Chen, Y. C.	1568/1343	1.08 [0.91, 1.27]	0.39	1.05 [0.88, 1.26]	0.59	1.45 [0.82, 2.58]	0.20
2016Gui, Y.	1313/1381	0.92 [0.76, 1.11]	0.37	0.88 [0.72, 1.07]	0.21	1.44 [0.70, 2.96]	0.32
2017Ran, C.	1568/1385	1.03 [0.86, 1.23]	0.72	0.99 [0.82, 1.21]	0.96	1.65 [0.84, 3.23]	0.15
All Combination	2048/1869	1.02 [0.88, 1.19]	0.79	0.99 [0.84, 1.17]	0.92	1.44 [0.82, 2.52]	0.21

(12) *NFE2L2* rs6721961

Reference (remove)	NO. of cases/controls	AM		DM		RM	
		OR	p	OR	p	OR	p
2010von Otter, M. (i)	1914/1689	0.99 [0.88, 1.11]	0.83	0.97 [0.84, 1.12]	0.70	1.03 [0.79, 1.36]	0.80
2010von Otter, M. (ii)	1885/1686	1.01 [0.90, 1.14]	0.82	1.02 [0.88, 1.18]	0.78	1.00 [0.76, 1.31]	0.99
2013Chen, Y. C.	1596/1347	1.02 [0.89, 1.18]	0.76	0.99 [0.84, 1.17]	0.94	1.15 [0.83, 1.61]	0.40
2016Gui, Y.	1311/1385	1.03 [0.89, 1.18]	0.72	1.03 [0.87, 1.22]	0.70	1.02 [0.68, 1.53]	0.93
2017Ran, C.	1598/1385	1.04 [0.92, 1.18]	0.54	1.06 [0.91, 1.24]	0.47	1.01 [0.77, 1.32]	0.96
All Combination	2076/1873	1.02 [0.91, 1.14]	0.78	1.01 [0.88, 1.16]	0.84	1.04 [0.79, 1.35]	0.80

(13) *NFE2L2* rs35652124

Reference (remove)	NO. of cases/controls	AM		DM		RM	
		OR	p	OR	p	OR	p
2010von Otter, M. (i)	1914/1684	1.05 [0.95, 1.15]	0.36	1.05 [0.88, 1.26]	0.59	1.09 [0.91, 1.30]	0.36
2010von Otter, M. (ii)	1885/1681	1.00 [0.90, 1.10]	0.94	0.93 [0.77, 1.13]	0.48	1.07 [0.89, 1.28]	0.48
2013Chen, Y. C.	1596/1342	1.04 [0.93, 1.17]	0.45	1.03 [0.81, 1.31]	0.80	1.08 [0.87, 1.35]	0.49
2016Gui, Y.	1311/1379	1.04 [0.93, 1.17]	0.47	0.98 [0.73, 1.30]	0.87	1.19 [0.96, 1.47]	0.12
2017Ran, C.	1598/1386	1.01 [0.91, 1.12]	0.89	0.95 [0.73, 1.24]	0.70	1.10 [0.91, 1.33]	0.35
All Combination	2076/1868	1.03 [0.93, 1.13]	0.59	0.99 [0.81, 1.21]	0.91	1.10 [0.92, 1.31]	0.29

(14) *NFE2L2* rs2706110

Reference (remove)	NO. of cases/controls	AM		DM		RM	
		OR	p	OR	p	OR	p
2010von Otter, M. (i)	2235/2001	0.98 [0.85, 1.13]	0.78	0.99 [0.78, 1.26]	0.92	0.97 [0.73, 1.27]	0.81
2010von Otter, M. (ii)	2222/2018	1.08 [0.84, 1.38]	0.54	1.13 [0.81, 1.58]	0.46	0.94 [0.71, 1.23]	0.64
2015Todorovic, M.	1103/851	1.16 [0.96, 1.41]	0.13	1.21 [0.94, 1.57]	0.14	1.01 [0.70, 1.46]	0.96
2016Gui, Y.	1637/1703	1.09 [0.79, 1.50]	0.60	1.04 [0.73, 1.50]	0.82	1.13 [0.79, 1.63]	0.50
All Combination	2399/2191	1.06 [0.87, 1.30]	0.53	1.08 [0.83, 1.41]	0.57	0.99 [0.76, 1.30]	0.96

(15) *NFE2L2* rs10183914

Reference (remove)	NO. of cases/controls	AM		DM		RM	
		OR	p	OR	p	OR	p
2010von Otter, M. (i)	2240/2020	0.96 [0.88, 1.05]	0.37	0.96 [0.85, 1.08]	0.49	0.93 [0.78, 1.12]	0.44
2010von Otter, M. (ii)	2219/2023	0.96 [0.88, 1.05]	0.41	0.96 [0.85, 1.09]	0.56	0.93 [0.78, 1.11]	0.43
2015Todorovic, M.	1115/866	0.99 [0.86, 1.13]	0.85	1.02 [0.85, 1.23]	0.80	0.90 [0.69, 1.18]	0.45
2016Gui, Y.	1641/1721	0.91 [0.82, 1.00]	0.06	0.90 [0.78, 1.03]	0.13	0.85 [0.69, 1.04]	0.12
All Combination	2405/2210	0.95 [0.87, 1.04]	0.25	0.95 [0.85, 1.07]	0.43	0.91 [0.76, 1.08]	0.27

(16) *NFE2L2* rs1806649

Reference (remove)	NO. of cases/controls	AM		DM		RM	
		OR	p	OR	p	OR	p
2010von Otter, M. (i)	2247/2009	0.90 [0.70, 1.15]	0.41	0.94 [0.83, 1.07]	0.38	0.84 [0.44, 1.61]	0.60
2010von Otter, M. (ii)	2226/2018	1.00 [0.90, 1.11]	0.98	0.97 [0.85, 1.11]	0.66	1.11 [0.86, 1.43]	0.44
2015Todorovic, M.	1113/860	0.87 [0.65, 1.18]	0.38	0.90 [0.73, 1.11]	0.31	0.80 [0.37, 1.75]	0.58
2016Gui, Y.	1650/1710	0.87 [0.67, 1.13]	0.29	0.93 [0.81, 1.06]	0.27	0.79 [0.41, 1.52]	0.48
All Combination	2412/2199	0.92 [0.77, 1.11]	0.39	0.94 [0.83, 1.07]	0.34	0.90 [0.56, 1.46]	0.67

(17) *NFE2L2* rs2001350

Reference (remove)	NO. of cases/controls	AM		DM		RM	
		OR	p	OR	p	OR	p
2010von Otter, M. (i)	1976/2017	1.01 [0.87, 1.18]	0.87	1.00 [0.85, 1.18]	0.97	1.21 [0.63, 2.34]	0.57
2010von Otter, M. (ii)	1951/2020	1.08 [0.93, 1.26]	0.31	1.18 [0.87, 1.59]	0.29	1.04 [0.52, 2.06]	0.92
2015Todorovic, M.	842/859	1.17 [0.93, 1.46]	0.18	1.16 [0.72, 1.86]	0.54	5.66 [1.25, 25.67]	0.02
2017Ran, C.	1654/1725	1.05 [0.89, 1.24]	0.57	1.13 [0.72, 1.76]	0.59	1.04 [0.52, 2.08]	0.92
All Combination	2141/2207	1.06 [0.92, 1.23]	0.41	1.09 [0.83, 1.43]	0.52	1.27 [0.67, 2.42]	0.46

(18) *CYP2D6* rs3892097

Reference (remove)	NO. of cases/controls	AM		DM		RM	
		OR	p	OR	p	OR	p
1993Kurth, M. C.	1677/1977	1.10 [0.97, 1.25]	0.13	1.22 [0.97, 1.54]	0.09	1.03 [0.75, 1.41]	0.87
1994Plante-Bordeneuve, V.	1679/1999	1.12 [0.99, 1.27]	0.07	1.25 [0.98, 1.59]	0.07	1.09 [0.80, 1.49]	0.59
1995Chen, X.	1699/1875	1.15 [1.01, 1.30]	0.03	1.31 [1.04, 1.65]	0.02	1.05 [0.77, 1.43]	0.75
1996Bordet, R.	1622/1982	1.16 [1.02, 1.32]	0.02	1.35 [1.05, 1.72]	0.02	1.07 [0.78, 1.45]	0.69
1996Diederich, N.	1647/1981	1.15 [1.01, 1.30]	0.03	1.31 [1.02, 1.69]	0.04	1.10 [0.81, 1.51]	0.54
1996Gasser, T.	1612/2014	1.14 [1.01, 1.30]	0.04	1.32 [1.02, 1.70]	0.03	1.04 [0.76, 1.43]	0.79
1996Lucotte, G.	1680/2040	1.11 [0.98, 1.26]	0.10	1.24 [0.98, 1.57]	0.07	1.01 [0.74, 1.38]	0.93
1999Atkinson, A.	1694/2012	1.11 [0.98, 1.26]	0.10	1.21 [0.97, 1.52]	0.09	1.05 [0.77, 1.44]	0.74
1999Nicholl, D. J.	1521/1882	1.14 [1.00, 1.30]	0.05	1.31 [1.01, 1.71]	0.05	1.08 [0.77, 1.49]	0.66
1999Payami, H.	1151/1840	1.20 [1.04, 1.38]	0.01	1.34 [1.03, 1.75]	0.03	1.17 [0.83, 1.66]	0.37
2000Woo, S. I.	1634/1965	1.13 [1.00, 1.28]	0.05	1.26 [1.00, 1.60]	0.05	1.06 [0.78, 1.44]	0.69
2004Elbaz, A.	1537/1668	1.14 [0.99, 1.30]	0.06	1.32 [1.01, 1.73]	0.04	1.02 [0.73, 1.42]	0.91
2007Halling, J.	1648/1934	1.17 [1.03, 1.33]	0.02	1.35 [1.06, 1.72]	0.02	1.07 [0.76, 1.49]	0.71
2010Singh, M.	1650/1962	1.12 [0.99, 1.27]	0.08	1.24 [0.98, 1.58]	0.08	1.08 [0.79, 1.47]	0.63
All Combination	1727/2087	1.14 [1.00, 1.29]	0.04	1.29 [1.02, 1.63]	0.04	1.06 [0.78, 1.44]	0.69

(19) *CYP2D6* A2637

Reference (remove)	NO. of cases/controls	AM		DM		RM	
		OR	p	OR	p	OR	p
1994Plante-Bordeneuve, V.	437/510	0.97 [0.48, 1.98]	0.94	0.97 [0.48, 1.99]	0.94	NA	NA
1996Bordet, R.	380/493	1.40 [0.63, 3.13]	0.41	1.41 [0.63, 3.17]	0.40	NA	NA
1996Lucotte, G.	438/551	1.07 [0.54, 2.09]	0.85	1.07 [0.54, 2.10]	0.85	NA	NA
1999Nicholl, D. J.	279/393	1.34 [0.57, 3.16]	0.50	1.35 [0.57, 3.21]	0.49	NA	NA
2007Halling, J.	406/445	1.03 [0.52, 2.02]	0.94	1.03 [0.52, 2.03]	0.94	NA	NA
All Combination	485/598	1.12 [0.58, 2.16]	0.73	1.12 [0.58, 2.18]	0.73	NA	NA

(20) *CYP2E1* rs2031920

Reference (remove)	NO. of cases/controls	AM		DM		RM	
		OR	p	OR	p	OR	p
2000Wang, J. (2)	304/351	1.07 [0.80, 1.43]	0.65	1.07 [0.75, 1.52]	0.70	1.19 [0.50, 2.85]	0.70
2002Wu, R. M.	220/250	1.41 [0.92, 2.14]	0.11	1.46 [0.91, 2.35]	0.12	1.69 [0.40, 7.20]	0.48
2008Singh, M.	384/401	1.09 [0.85, 1.39]	0.49	1.08 [0.81, 1.45]	0.59	1.31 [0.62, 2.76]	0.48
All Combination	454/501	1.13 [0.89, 1.44]	0.32	1.14 [0.86, 1.52]	0.37	1.31 [0.62, 2.76]	0.48

(21) *PON1* rs705379

Reference (remove)	NO. of cases/controls	AM		DM		RM	
		OR	p	OR	p	OR	p
2003Kelada, S. N.	772/930	0.97 [0.85, 1.11]	0.67	0.97 [0.78, 1.20]	0.79	0.95 [0.77, 1.19]	0.68
2012Belin, A. C.	405/615	0.96 [0.80, 1.15]	0.65	0.94 [0.71, 1.24]	0.65	0.96 [0.72, 1.29]	0.79
2013Lee, P. C.	639/771	0.94 [0.81, 1.09]	0.42	1.00 [0.79, 1.27]	0.99	0.85 [0.67, 1.09]	0.19
All Combination	908/1158	0.96 [0.85, 1.08]	0.49	0.97 [0.80, 1.18]	0.77	0.92 [0.75, 1.12]	0.41

(22) *PON1* rs854560

Reference (remove)	NO. of cases/controls	AM		DM		RM	
		OR	p	OR	p	OR	p
2000Wang, J. (1)	2601/2996	1.20 [1.10, 1.30]	<0.0001	1.21 [1.08, 1.35]	0.0007	1.37 [1.16, 1.62]	0.0003
2001Akhmedova, S. N.	2664/2969	1.17 [1.08, 1.28]	0.0002	1.17 [1.04, 1.31]	0.007	1.36 [1.14, 1.62]	0.0006
2002Carmine, A.	2681/3076	1.18 [1.09, 1.28]	<0.0001	1.18 [1.06, 1.32]	0.003	1.36 [1.15, 1.62]	0.0005
2003Kelada, S. N.	2631/2933	1.21 [1.11, 1.32]	<0.0001	1.21 [1.08, 1.36]	0.0008	1.41 [1.18, 1.69]	0.0001
2004Clarimon, J.	2643/3046	1.21 [1.11, 1.31]	<0.0001	1.21 [1.09, 1.36]	0.0007	1.40 [1.17, 1.66]	0.0002
2005Fong, C. S.	2656/3014	1.20 [1.11, 1.31]	<0.0001	1.22 [1.09, 1.36]	0.0004	1.37 [1.15, 1.62]	0.0003
2010Manthripragada, A. D.(i)	2499/2886	1.19 [1.10, 1.30]	<0.0001	1.21 [1.08, 1.36]	0.001	1.34 [1.12, 1.61]	0.001
2010Manthripragada, A. D.(ii)	2430/2813	1.20 [1.09, 1.30]	<0.0001	1.21 [1.08, 1.36]	0.001	1.35 [1.13, 1.62]	0.001
2011Punia, S.	2294/2702	1.21 [1.11, 1.32]	<0.0001	1.23 [1.09, 1.39]	0.0007	1.36 [1.14, 1.62]	0.0006
2012Belin, A. C.	2287/2664	1.22 [1.12, 1.34]	<0.0001	1.23 [1.09, 1.38]	0.0009	1.44 [1.19, 1.75]	0.0002
2013Lee, P. C.	2494/2736	1.21 [1.10, 1.32]	<0.0001	1.22 [1.09, 1.38]	0.0007	1.37 [1.14, 1.64]	0.0008
2019Mota, A.	2711/3101	1.17 [1.08, 1.28]	0.0002	1.20 [1.07, 1.34]	0.002	1.29 [1.09, 1.54]	0.003
All Combination	2781/3176	1.20 [1.10, 1.30]	<0.0001	1.21 [1.08, 1.35]	0.0007	1.37 [1.15, 1.62]	0.0003

(23) *PON1* rs662

Reference (remove)	NO. of cases/controls	AM		DM		RM	
		OR	p	OR	p	OR	p
1998Kondo, I.	2036/2286	0.97 [0.88, 1.06]	0.45	0.97 [0.85, 1.09]	0.59	0.93 [0.77, 1.13]	0.47
1999Akhmedova, S.	2084/2421	1.01 [0.92, 1.10]	0.85	0.99 [0.87, 1.12]	0.86	1.05 [0.89, 1.25]	0.55
2000Taylor, M. C.	2113/2416	1.01 [0.92, 1.10]	0.89	0.98 [0.86, 1.11]	0.71	1.07 [0.90, 1.27]	0.43
2000Wang, J. (1)	2025/2358	1.00 [0.91, 1.10]	0.96	0.99 [0.87, 1.12]	0.84	1.04 [0.86, 1.25]	0.69
2003Kelada, S. N.	2055/2296	1.02 [0.93, 1.12]	0.67	1.00 [0.88, 1.14]	0.95	1.07 [0.89, 1.27]	0.48
2004Clarimon, J.	2061/2403	1.00 [0.92, 1.10]	0.95	0.98 [0.87, 1.12]	0.81	1.04 [0.88, 1.24]	0.64
2011Punia, S.	1718/2064	1.02 [0.92, 1.13]	0.68	1.00 [0.88, 1.15]	0.95	1.07 [0.88, 1.30]	0.48
2012Belin, A. C.	1694/1994	0.99 [0.90, 1.09]	0.86	0.97 [0.84, 1.11]	0.65	1.02 [0.86, 1.23]	0.80
2013Lee, P. C.	1924/2141	1.05 [0.95, 1.15]	0.34	1.03 [0.90, 1.18]	0.63	1.12 [0.93, 1.34]	0.24
2019Mota, A.	2135/2463	1.01 [0.92, 1.10]	0.82	0.99 [0.88, 1.12]	0.88	1.06 [0.89, 1.26]	0.51
All Combination	2205/2538	1.01 [0.92, 1.10]	0.87	0.99 [0.88, 1.12]	0.87	1.05 [0.88, 1.24]	0.59

(24) *NAT2* rs1799929

Reference (remove)	NO. of cases/controls	AM		DM		RM	
		OR	p	OR	p	OR	p
1999Nicholl, D. J.	201/368	0.93 [0.72, 1.20]	0.59	0.93 [0.65, 1.33]	0.68	0.89 [0.54, 1.45]	0.64
2006Borlak, J.	282/330	1.05 [0.83, 1.33]	0.69	1.25 [0.89, 1.76]	0.19	0.81 [0.52, 1.26]	0.34
2010Singh, M.	329/448	0.92 [0.75, 1.13]	0.43	0.96 [0.70, 1.30]	0.77	0.83 [0.58, 1.19]	0.31
All Combination	406/573	0.96 [0.80, 1.16]	0.69	1.03 [0.79, 1.36]	0.81	0.84 [0.59, 1.18]	0.31

(25) *NAT2* rs1799930

Reference (remove)	NO. of cases/controls	AM		DM		RM	
		OR	p	OR	p	OR	p
1999Nicholl, D. J.	201/368	1.10 [0.76, 1.60]	0.62	1.09 [0.68, 1.75]	0.71	1.28 [0.53, 3.10]	0.58
2006Borlak, J.	281/330	1.01 [0.79, 1.30]	0.92	1.02 [0.74, 1.41]	0.88	0.99 [0.57, 1.74]	0.98
2010Singh, M.	328/448	0.99 [0.74, 1.31]	0.92	0.97 [0.68, 1.38]	0.87	1.04 [0.53, 2.04]	0.90
All Combination	405/573	1.02 [0.80, 1.30]	0.87	1.02 [0.75, 1.37]	0.90	1.06 [0.61, 1.83]	0.84

(26) *MDR1* rs1128503

Reference (remove)	NO. of cases/controls	AM		DM		RM	
		OR	p	OR	p	OR	p
2004Tan, E. K.	760/779	0.99 [0.86, 1.14]	0.90	1.11 [0.91, 1.36]	0.24	0.83 [0.53, 1.32]	0.43
2005Tan, E. K.	733/712	1.01 [0.87, 1.17]	0.89	1.14 [0.91, 1.41]	0.26	0.85 [0.51, 1.44]	0.55
2009Funke, C.	618/616	0.89 [0.76, 1.05]	0.15	1.02 [0.79, 1.32]	0.86	0.71 [0.54, 0.93]	0.01
2009Westerlund, M.	643/647	1.04 [0.89, 1.22]	0.59	1.14 [0.89, 1.46]	0.29	0.97 [0.71, 1.32]	0.84
All Combination	918/918	0.98 [0.86, 1.12]	0.81	1.11 [0.91, 1.36]	0.30	0.84 [0.59, 1.19]	0.32

(27) *MDR1* rs1045642

Reference (remove)	NO. of cases/controls	AM		DM		RM	
		OR	p	OR	p	OR	p
2002Furuno, T.	2064/2647	1.05 [0.96, 1.14]	0.27	1.03 [0.90, 1.18]	0.68	1.10 [0.96, 1.26]	0.16
2003Dekker, M. C.	2052/2650	1.07 [0.98, 1.16]	0.12	1.05 [0.91, 1.20]	0.53	1.14 [0.99, 1.31]	0.06
2004Tan, E. K.	2001/2614	1.06 [0.97, 1.15]	0.18	1.05 [0.91, 1.20]	0.53	1.12 [0.97, 1.28]	0.12
2005Tan, E. K.	1974/2547	1.06 [0.97, 1.15]	0.18	1.05 [0.91, 1.21]	0.47	1.11 [0.97, 1.27]	0.14
2009Funke, C.	1859/2451	1.05 [0.96, 1.15]	0.28	1.02 [0.89, 1.18]	0.76	1.11 [0.96, 1.29]	0.14
2009Westerlund, M.	1882/2461	1.08 [0.98, 1.17]	0.11	1.04 [0.91, 1.20]	0.54	1.16 [1.01, 1.35]	0.04
2009Zschiedrich, K. (i)	1894/2630	1.07 [0.99, 1.17]	0.10	1.08 [0.94, 1.24]	0.26	1.11 [0.97, 1.28]	0.13
2009Zschiedrich, K. (ii)	2117/2692	1.06 [0.98, 1.15]	0.16	1.05 [0.91, 1.20]	0.51	1.12 [0.98, 1.28]	0.10
2010Dutheil, F.	1952/2271	1.06 [0.98, 1.15]	0.15	1.02 [0.89, 1.18]	0.77	1.16 [1.01, 1.34]	0.04
2013Kiyohara, C.	1921/2385	1.04 [0.95, 1.13]	0.40	1.00 [0.86, 1.16]	0.99	1.10 [0.96, 1.27]	0.17
2015Narayan, S.	1874/2182	1.04 [0.95, 1.13]	0.45	1.05 [0.91, 1.22]	0.49	1.04 [0.90, 1.21]	0.58
All Combination	2159/2753	1.06 [0.97, 1.15]	0.18	1.04 [0.91, 1.19]	0.56	1.12 [0.98, 1.28]	0.11

(28) *MDR1* rs2032582

Reference (remove)	NO. of cases/controls	AM		DM		RM	
		OR	p	OR	p	OR	p
2002Furuno, T.	1404/1985	0.97 (0.88, 1.07]	0.52	0.99 [0.83, 1.19]	0.95	0.94 [0.81, 1.08]	0.38
2004Tan, E. K.	1341/1952	0.96 [0.86, 1.06]	0.38	0.98 [0.82, 1.17]	0.82	0.92 [0.79, 1.07]	0.27
2005Tan, E. K.	1314/1885	0.97 [0.87, 1.07]	0.53	0.99 [0.82, 1.18]	0.89	0.94 [0.81, 1.09]	0.42
2009Funke, C.	1199/1792	0.98 [0.88, 1.09]	0.65	0.97 [0.80, 1.17]	0.73	0.97 [0.83, 1.13]	0.70
2009Westerlund, M.	1226/1800	0.93 [0.84, 1.03]	0.17	0.90 [0.74, 1.09]	0.27	0.92 [0.78, 1.07]	0.27
2010Dutheil, F.	1292/1609	0.96 [0.86, 1.06]	0.40	0.99 [0.82, 1.20]	0.92	0.91 [0.78, 1.07]	0.25
2015Narayan, S.	1218/1523	0.98 [0.88, 1.09]	0.69	1.10 [0.90, 1.34]	0.35	0.90 [0.76, 1.05]	0.18
All Combination	1499/2091	0.96 [0.87, 1.06]	0.42	0.99 [0.83, 1.17]	0.87	0.93 [0.80, 1.07]	0.30

(29) *BST1* rs11724635

Reference (remove)	NO. of cases/controls	AM		DM		RM	
		OR	p	OR	p	OR	p
2012Miyake, Y.	1064/1084	1.04 [0.92, 1.18]	0.51	1.05 [0.87, 1.25]	0.63	1.07 [0.86, 1.33]	0.56
2014Chen, M. L.	825/954	1.13 [0.99, 1.29]	0.07	1.15 [0.94, 1.40]	0.17	1.22 [0.96, 1.56]	0.11
2015Chang, K. H.	697/844	1.06 [0.91, 1.22]	0.46	1.06 [0.86, 1.30]	0.58	1.10 [0.84, 1.44]	0.50
All Combination	1293/1441	1.07 [0.96, 1.20]	0.19	1.08 [0.92, 1.27]	0.33	1.12 [0.92, 1.37]	0.25

(30) *BST1* rs11931532

Reference (remove)	NO. of cases/controls	AM		DM		RM	
		OR	p	OR	p	OR	p
2010Chang, X. L.	1248/1387	0.92 [0.83, 1.03]	0.13	0.95 [0.80, 1.14]	0.61	0.83 [0.70, 1.00]	0.05
2012Miyake, Y.	1639/1534	0.91 [0.82, 1.00]	0.05	0.93 [0.79, 1.09]	0.36	0.81 [0.68, 0.96]	0.02
2015Guo, J. F.	849/861	0.86 [0.75, 0.99]	0.03	0.82 [0.65, 1.03]	0.08	0.82 [0.66, 1.02]	0.07
All Combination	1868/1891	0.90 [0.82, 0.99]	0.02	0.91 [0.78, 1.06]	0.22	0.82 [0.70, 0.96]	0.01

(31) *HLA-DRB1* rs660895

Reference (remove)	NO. of cases/controls	AM		DM		RM	
		OR	p	OR	p	OR	p
2012Ahmed, I.	2542/3196	0.82 [0.75, 0.90]	<0.0001	0.81 [0.72, 0.90]	0.0001	0.72 [0.55, 0.94]	0.02

2017Chuang, Y. H. (i)	1494/2723	0.76 [0.67, 0.85]	<0.00001	0.76 [0.66, 0.87]	<0.0001	0.52 [0.36, 0.76]	0.001
2017Chuang, Y. H. (ii)	2532/3190	0.83 [0.75, 0.91]	<0.0001	0.81 [0.72, 0.90]	0.0001	0.72 [0.55, 0.94]	0.01
2020Chang, K. H.	2555/3845	0.79 [0.72, 0.87]	<0.00001	0.77 [0.69, 0.86]	<0.00001	0.66 [0.49, 0.88]	0.004
All Combination	3041/4318	0.80 [0.74, 0.87]	<0.00001	0.79 [0.71, 0.87]	<0.00001	0.67 [0.52, 0.86]	0.002

(32) *CCDC62* rs12817488

Reference (remove)	NO. of cases/controls	AM		DM		RM	
		OR	p	OR	p	OR	p
2013Li, N. N.	848/941	0.75 [0.65, 0.85]	<0.0001	0.68 [0.55, 0.83]	0.0002	0.69 [0.55, 0.86]	0.001
2014Liu, R. R.	1267/1226	0.82 [0.73, 0.91]	0.0004	0.81 [0.69, 0.97]	0.02	0.72 [0.60, 0.87]	0.0007
2015Yu, R. L.	1101/1131	0.83 [0.74, 0.94]	0.002	0.79 [0.65, 0.94]	0.009	0.79 [0.65, 0.97]	0.02
All Combination	1608/1649	0.80 [0.73, 0.89]	<0.0001	0.77 [0.66, 0.89]	0.0005	0.74 [0.62, 0.87]	0.0003

(33) *HFE* rs1800562

Reference (remove)	NO. of cases/controls	AM		DM		RM	
		OR	p	OR	p	OR	p
2002Borie, C.	1573/4597	0.89 [0.73, 1.08]	0.24	0.88 [0.72, 1.08]	0.23	0.93 [0.35, 2.51]	0.89
2002Buchanan, D. D.	1206/4169	1.03 [0.82, 1.30]	0.79	1.02 [0.80, 1.30]	0.87	1.50 [0.48, 4.71]	0.49
2003Dekker, M. C.	1447/1740	0.87 [0.70, 1.08]	0.21	0.88 [0.70, 1.10]	0.26	0.55 [0.16, 1.91]	0.34
2006Guerreiro, R. J.	1512/4539	0.83 [0.68, 1.02]	0.07	0.82 [0.66, 1.01]	0.06	0.93 [0.35, 2.51]	0.89
2007Aamodt, A. H.	1256/4149	0.85 [0.67, 1.07]	0.16	0.84 [0.66, 1.07]	0.17	0.84 [0.26, 2.67]	0.76
2008Halling, J.	1565/4501	0.90 [0.73, 1.10]	0.30	0.89 [0.72, 1.10]	0.27	1.08 [0.37, 3.11]	0.89
2011Greco, V.	1463/4474	0.89 [0.73, 1.08]	0.22	0.88 [0.71, 1.08]	0.21	0.93 [0.35, 2.51]	0.89
2013Mariani, S.	1571/4515	0.87 [0.72, 1.06]	0.18	0.87 [0.70, 1.06]	0.17	0.93 [0.35, 2.51]	0.89
2016Mariani, S.	1559/4548	0.89 [0.73, 1.08]	0.24	0.88 [0.72, 1.08]	0.24	0.93 [0.35, 2.51]	0.89
All Combination	1644/4654	0.89 [0.73, 1.08]	0.23	0.88 [0.72, 1.08]	0.22	0.93 [0.35, 2.51]	0.89

(34) *HFE* rs1799945

Reference (remove)	NO. of cases/controls	AM		DM		RM	
		OR	p	OR	p	OR	p
2002Borie, C.	1151/4092	1.03 [0.88, 1.19]	0.74	1.02 [0.86, 1.21]	0.83	1.15 [0.68, 1.95]	0.61
2003Dekker, M. C.	1020/1237	1.07 [0.90, 1.26]	0.46	1.04 [0.86, 1.25]	0.71	1.56 [0.85, 2.85]	0.15
2006Guerreiro, R. J.	1085/4036	1.04 [0.89, 1.21]	0.63	1.04 [0.88, 1.24]	0.65	1.09 [0.62, 1.90]	0.76
2007Aamodt, A. H.	829/3646	1.08 [0.91, 1.28]	0.36	1.09 [0.90, 1.32]	0.36	1.14 [0.63, 2.08]	0.66
2008Halling, J.	1138/3998	1.02 [0.88, 1.19]	0.79	1.01 [0.85, 1.20]	0.90	1.18 [0.68, 2.07]	0.55
2011Greco, V.	1036/3971	0.97 [0.83, 1.13]	0.70	0.96 [0.80, 1.14]	0.65	1.03 [0.58, 1.83]	0.92
2013Mariani, S.	1140/4012	1.03 [0.89, 1.20]	0.70	1.02 [0.86, 1.21]	0.86	1.24 [0.73, 2.12]	0.42
2016Mariani, S.	1120/4065	1.03 [0.89, 1.20]	0.67	1.03 [0.87, 1.22]	0.75	1.15 [0.68, 1.96]	0.61
All Combination	1217/4151	1.03 [0.89, 1.19]	0.68	1.02 [0.87, 1.21]	0.78	1.18 [0.70, 1.99]	0.53

(35) *MTHFR* rs1801133

Reference (remove)	NO. of cases/controls	AM		DM		RM	
		OR	p	OR	p	OR	p
2000Yasui, K.	2160/2512	1.11 [0.91, 1.34]	0.31	1.14 [0.88, 1.47]	0.33	1.02 [0.84, 1.22]	0.87
2005Wullner, U.	1908/2223	1.12 [0.91, 1.38]	0.30	1.14 [0.87, 1.50]	0.34	1.06 [0.87, 1.29]	0.57
2006Religa, D.	2136/2465	1.09 [0.90, 1.32]	0.38	1.11 [0.86, 1.43]	0.41	1.01 [0.84, 1.22]	0.88
2006Todorovic, Z.	2137/2512	1.15 [0.95, 1.39]	0.16	1.20 [0.93, 1.55]	0.16	1.04 [0.86, 1.25]	0.71
2007Caccamo, D.	2201/2479	1.07 [0.90, 1.29]	0.45	1.11 [0.87, 1.42]	0.40	0.98 [0.81, 1.18]	0.84

2009Camicioli, R. M.	2199/2516	1.14 [0.94, 1.38]	0.18	1.17 [0.91, 1.52]	0.22	1.04 [0.87, 1.25]	0.66
2009Rodriguez-Oroz, M. C.	2173/2537	1.15 [0.96, 1.39]	0.13	1.18 [0.92, 1.53]	0.19	1.06 [0.88, 1.27]	0.56
2009Yuan, R. Y.	2174/2455	1.07 [0.90, 1.29]	0.44	1.09 [0.86, 1.39]	0.47	1.01 [0.84, 1.21]	0.95
2011Fong, C. S.	2039/2347	1.11 [0.91, 1.36]	0.29	1.16 [0.88, 1.52]	0.29	1.01 [0.84, 1.22]	0.90
2012Gorgone, G.	2190/2483	1.07 [0.89, 1.28]	0.46	1.12 [0.87, 1.43]	0.39	0.97 [0.80, 1.17]	0.72
2014Kumudini, N.	2099/2149	1.09 [0.90, 1.32]	0.37	1.13 [0.87, 1.47]	0.35	1.01 [0.84, 1.22]	0.90
2014Liao, Q.	1485/1848	1.16 [0.97, 1.39]	0.10	1.24 [1.03, 1.50]	0.03	1.07 [0.85, 1.35]	0.54
2016Zahra, C.	2099/2254	1.13 [0.92, 1.39]	0.23	1.17 [0.89, 1.54]	0.26	1.04 [0.86, 1.26]	0.66
All Combination	2250/2565	1.11 [0.93, 1.34]	0.26	1.15 [0.90, 1.47]	0.26	1.02 [0.85, 1.23]	0.81

(36) *MTHFR* rs1801131

Reference (remove)	NO. of cases/controls	AM		DM		RM	
		OR	p	OR	p	OR	p
2005Wullner, U.	208/185	1.23 [0.85, 1.79]	0.27	1.25 [0.82, 1.90]	0.31	1.36 [0.63, 2.91]	0.44
2009Camicioli, R. M.	500/478	0.96 [0.67, 1.37]	0.83	0.93 [0.72, 1.20]	0.55	0.70 [0.46, 1.06]	0.09
2009Rodriguez-Oroz, M. C.	468/501	0.93 [0.69, 1.24]	0.61	0.91 [0.70, 1.17]	0.46	0.72 [0.47, 1.09]	0.12
2009Yuan, R. Y.	474/417	1.16 [0.69, 1.93]	0.58	0.98 [0.75, 1.27]	0.86	0.75 [0.49, 1.15]	0.18
All Combination	550/527	1.05 [0.75, 1.47]	0.79	0.97 [0.76, 1.23]	0.78	0.77 [0.51, 1.14]	0.19

Note: allele model, AM; dominant model, DM; recessive model, RM.

Supplementary Table5: The results of the Mendelian Randomization analysis investigating the cause relationships between ADP-ribosyl cyclase, PON1 and Parkinson's disease.

Exposure	Outcome	Method	IV condition	SNPs	Beta	SE	*p* value
**ADP-ribosyl cyclase (*BST1*)**	**PD**	**IVW**	raw	6	0.08	0.04	**0.02**
			outliers removed	3	0.15	0.03	**1.64×10^{-7}**
		**MR PRESSO**	raw	6	0.08	0.04	0.07
			outliers removed	3	0.15	6.13×10^{-3}	**1.73×10^{-3}**
		**MR Egger**	raw	6	-3.23×10^{-3}	0.06	0.96
			outliers removed	3	0.15	0.06	0.24
		**Weighted median**	raw	6	0.08	0.02	**1.83×10^{-4}**
			outliers removed	3	0.15	0.03	**9.13×10^{-6}**
**PON1 (*PON1*)**	**PD**	**IVW**	/	5	-0.21	0.10	**0.04**
		**MR PRESSO**	/	5	-0.21	0.10	0.11
		**MR Egger**	/	5	1.10	0.76	0.24
		**Weighted median**	/	5	-0.22	0.14	0.12
**PD**	**ADP-ribosyl cyclase (*BST1*)**	**IVW**	/	9	0.14	0.11	0.22
		**MR PRESSO**	/	9	0.14	0.11	0.22
		**MR Egger**	/	9	-0.13	0.34	0.73
		**Weighted median**	/	9	0.19	0.14	0.18
**PD**	**PON1 (*PON1*)**	**IVW**	/	22	-2.32×10^{-3}	0.02	0.92
		**MR PRESSO**	/	22	-2.32×10^{-3}	0.02	0.90
		**MR Egger**	/	22	0.03	0.08	0.67
		**Weighted median**	/	22	-4.56×10^{-3}	0.03	0.89

Note: instrumental variable, IV; standard error, SE; inverse-variance weighting, IVW; Mendelian randomization-pleiotropy residual sum and outlier, MR-PRESSO.

Supplementary Table 6: The heterogeneity and sensitivity results of ADP-ribosyl cyclase, PON1 and Parkinson's disease in Mendelian Randomization analysis.

Exposure	Outcome		SNPs	MR Egger intercept		Cochran's heterogeneity test			
				Intercept value	*p* value	IVW		Egger	
						Qvalue	*p* value	Qvalue	*p* value
**ADP-ribosyl cyclase (*BST1*)**	**PD**	raw	6	0.07	0.14	32.46	**4.82×10^{-6}**	17.82	**1.34×10^{-3}**
		outliers removed	3	-1.38×10^{-3}	0.97	0.1	0.95	0.1	0.76
**PON1 (*PON1*)**	**PD**	/	5	-0.14	0.18	3.92	0.42	0.85	0.84
**PD**	**ADP-ribosyl cyclase (*BST1*)**	/	9	0.04	0.44	4.78	0.78	4.12	0.77
**PD**	**PON1 (*PON1*)**	/	22	-5.61×10^{-3}	0.64	12.8	0.92	12.57	0.9

Note: inverse-variance weighting, IVW
