# Supplementary material for: Quantitative and causal analysis for inflammatory genes and the risk of Parkinson’s disease
Source: Front Immunol. 2023 Feb 28;14:1119315. doi: 10.3389/fimmu.2023.1119315 (PMC10011457; doi:10.3389/fimmu.2023.1119315)

Supplementary material 2: Supplementary Figures

Supplementary Figure1. The workflow of publications screening process for quantitative analysis.

(A) The gene of TNF-α

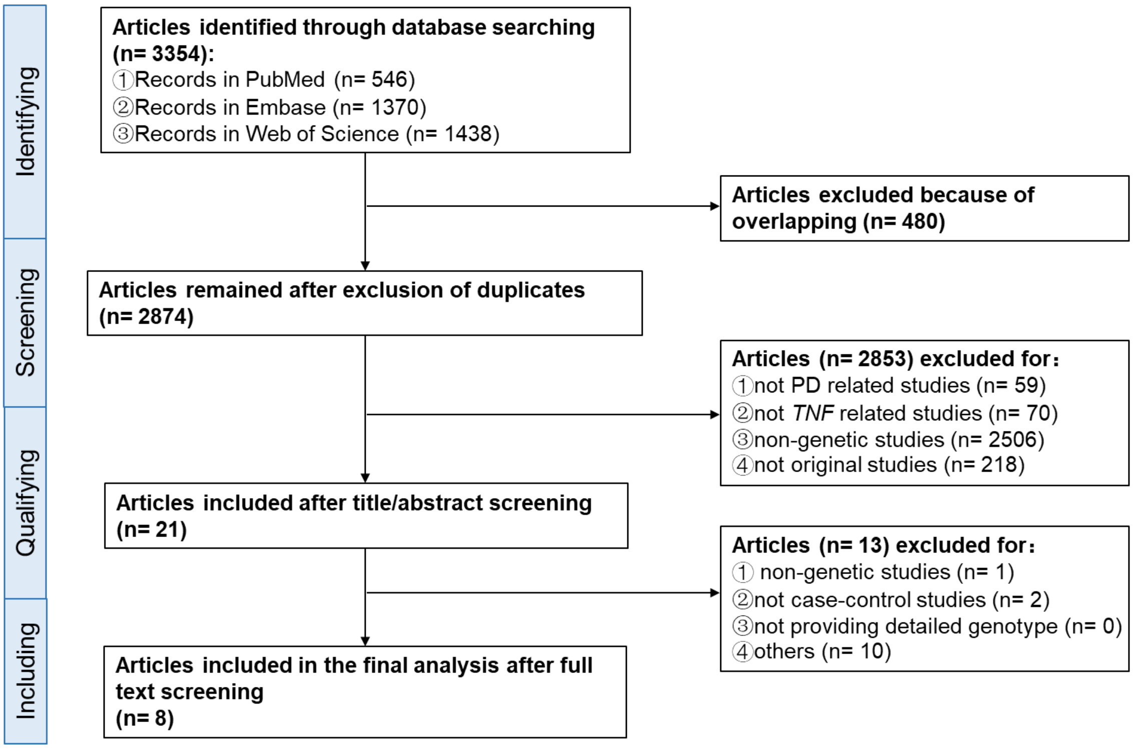

(B) The gene of IL

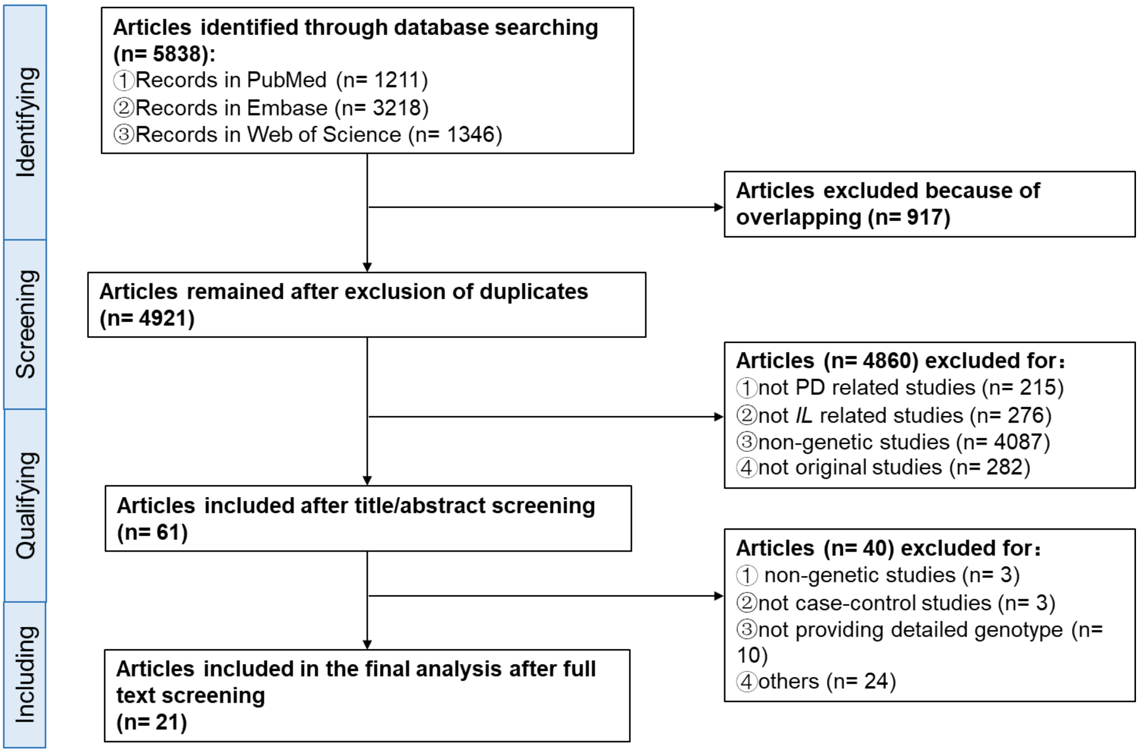

(C) The gene of NOS

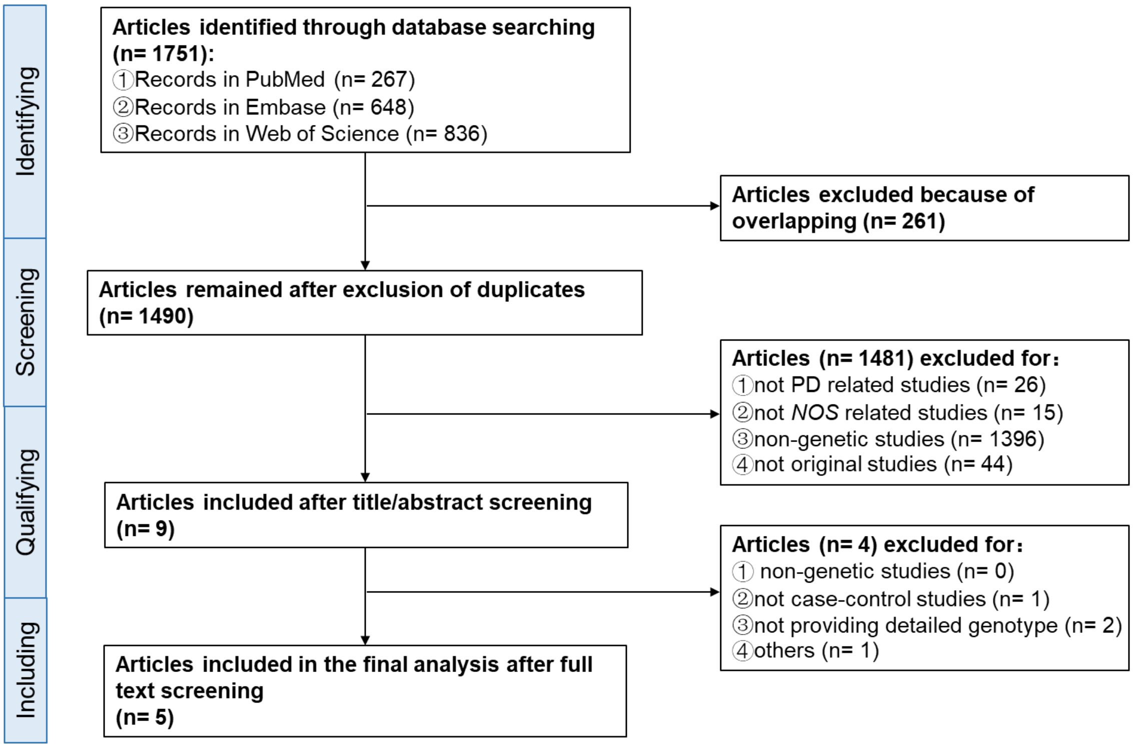

(D) The gene of MnSOD

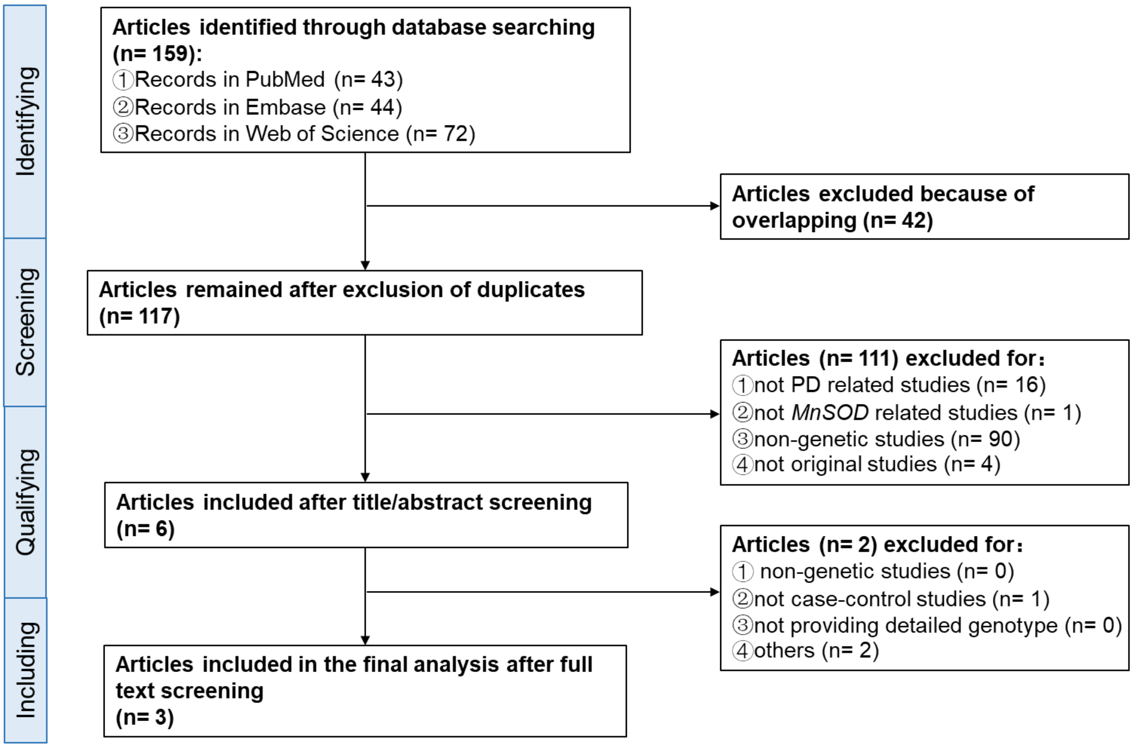

(E) The gene of NFE2L2

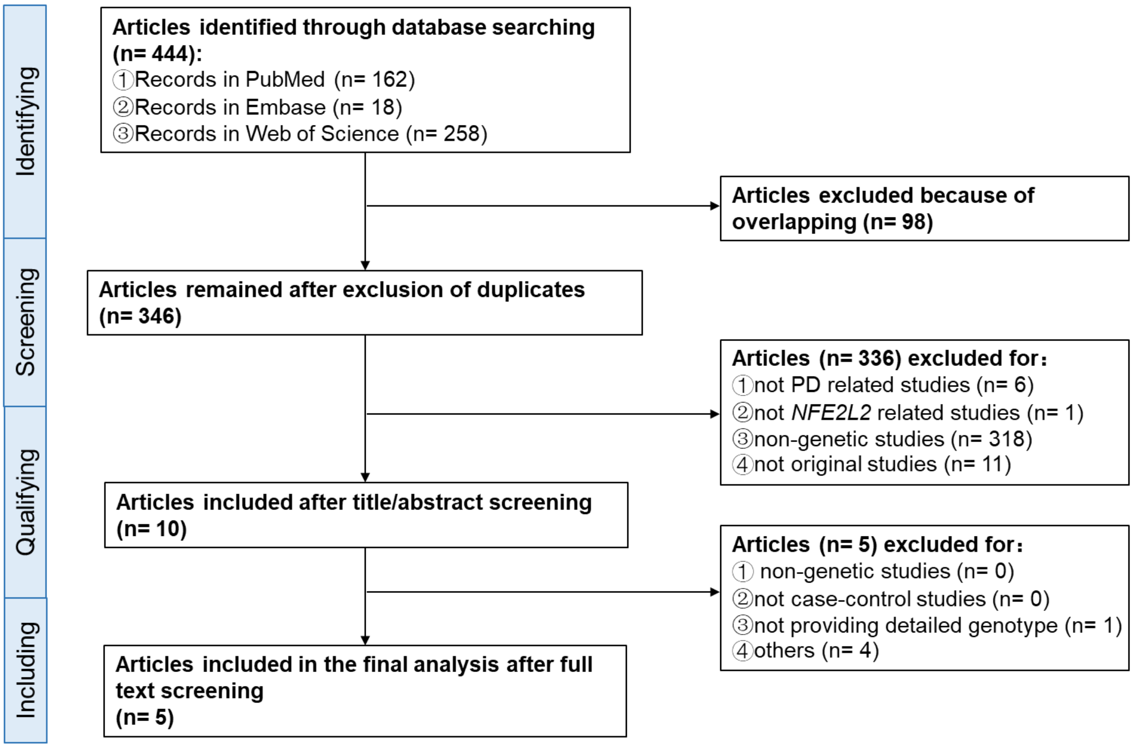

(F) The gene of CYP2D6

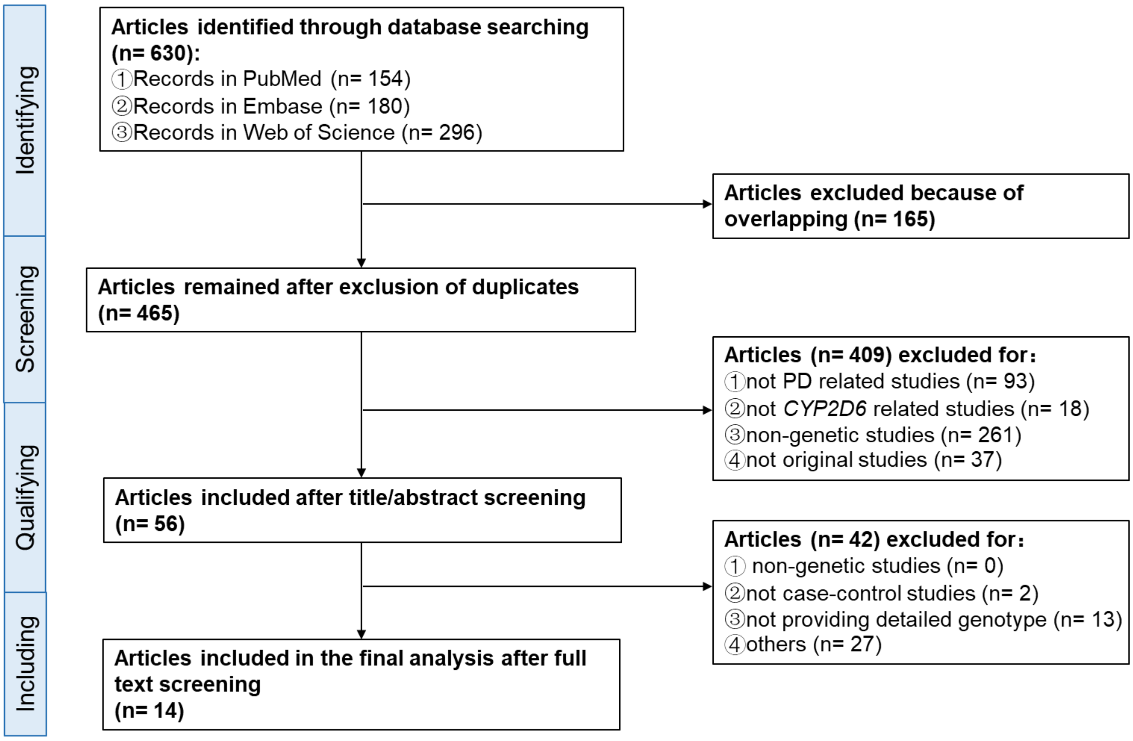

(G) The gene of PON

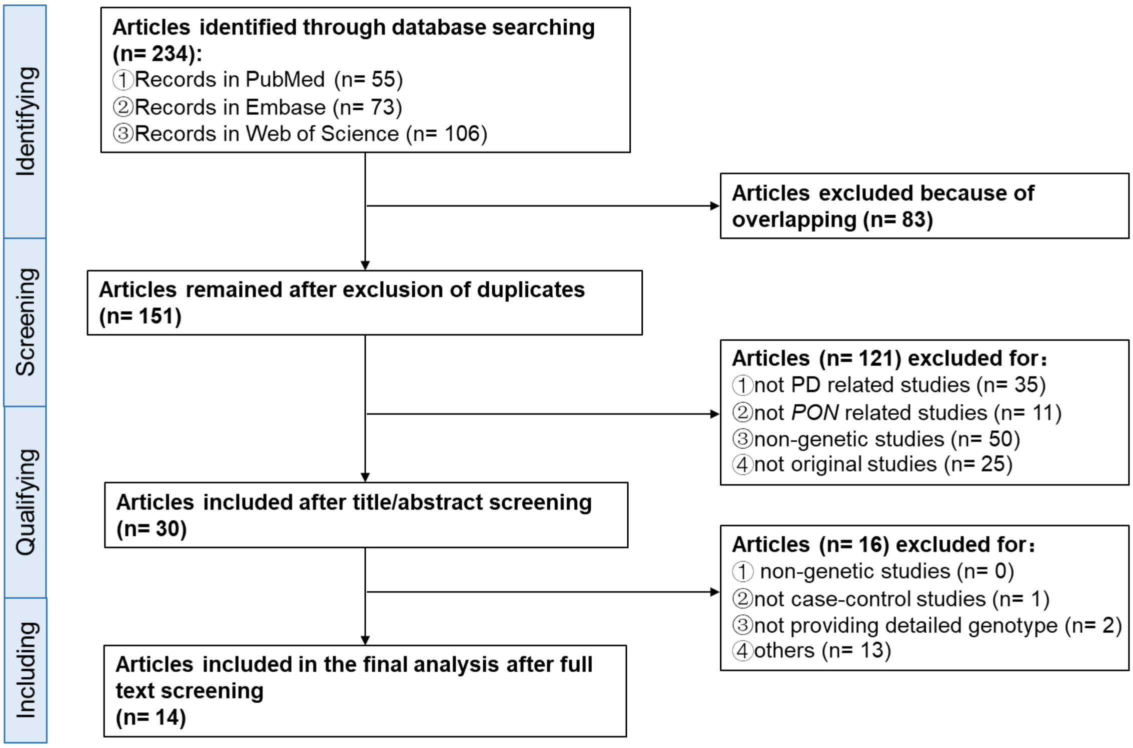

(H) The gene of CYP2E1

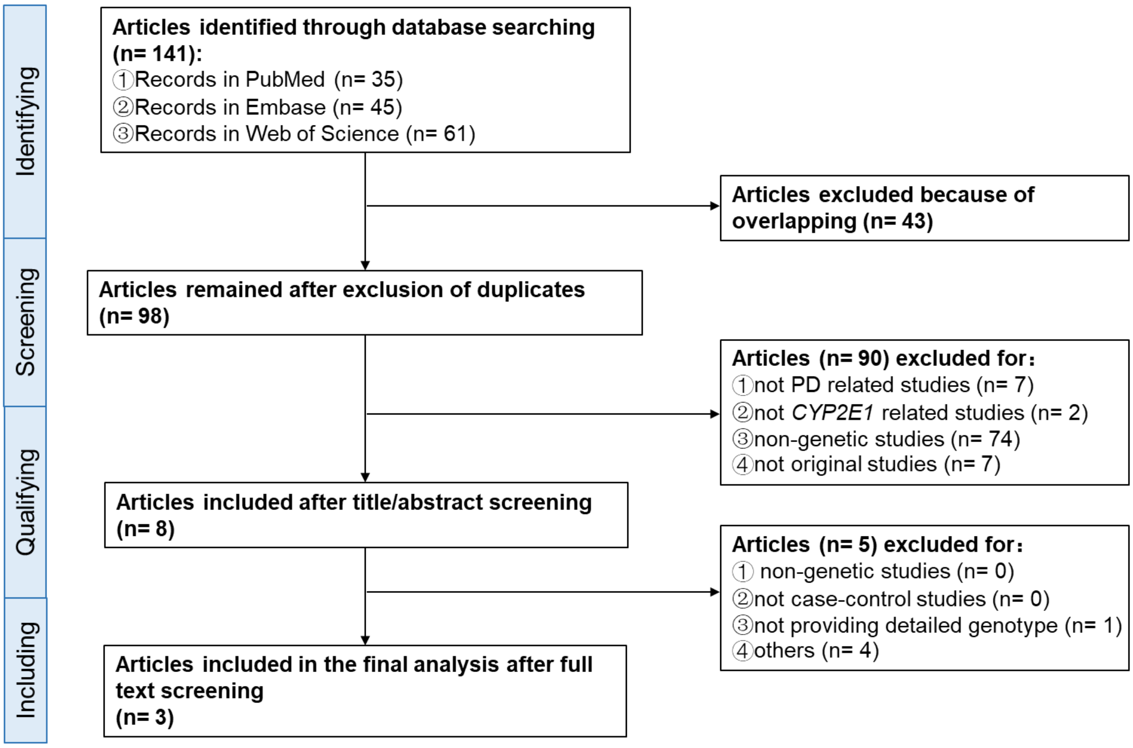

(I) The gene of NAT2

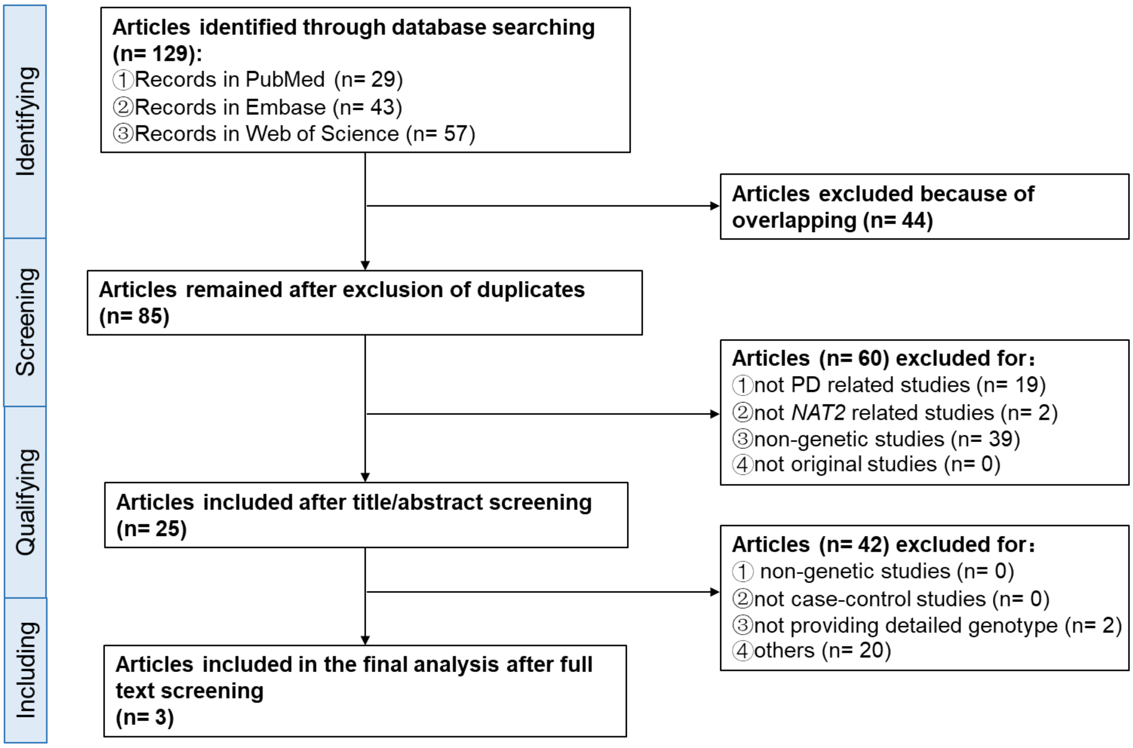

(J) The gene of MDR1/ABCB1

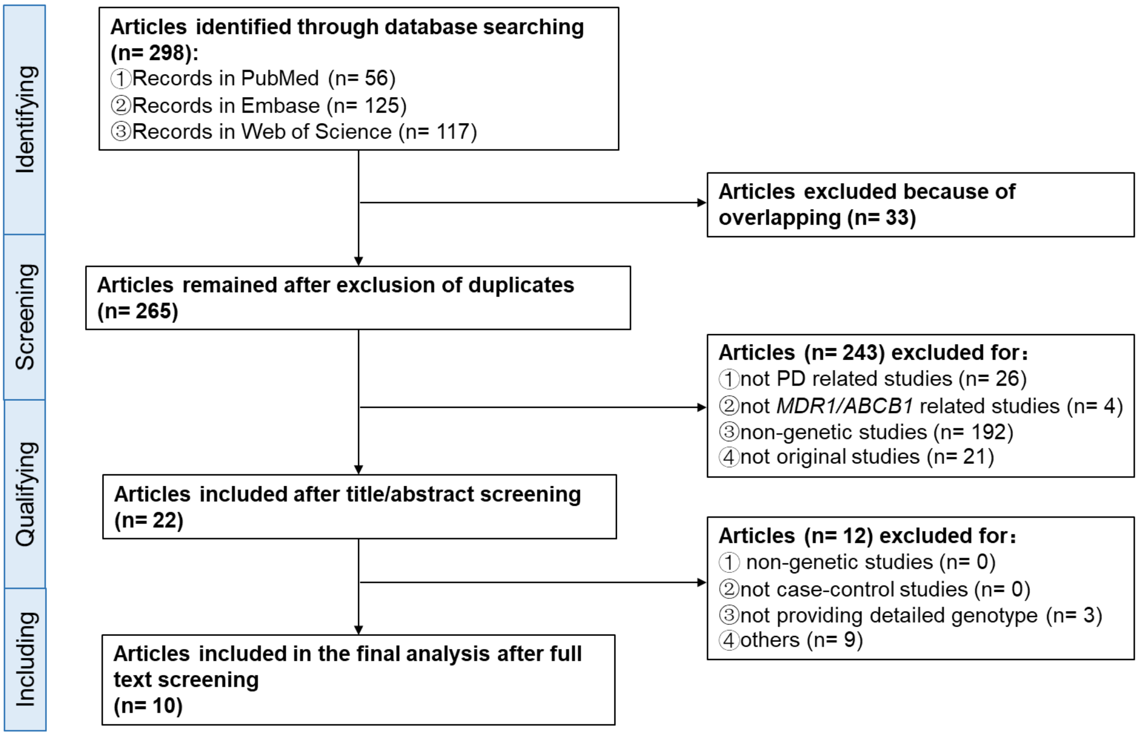

(K) The gene of BST1

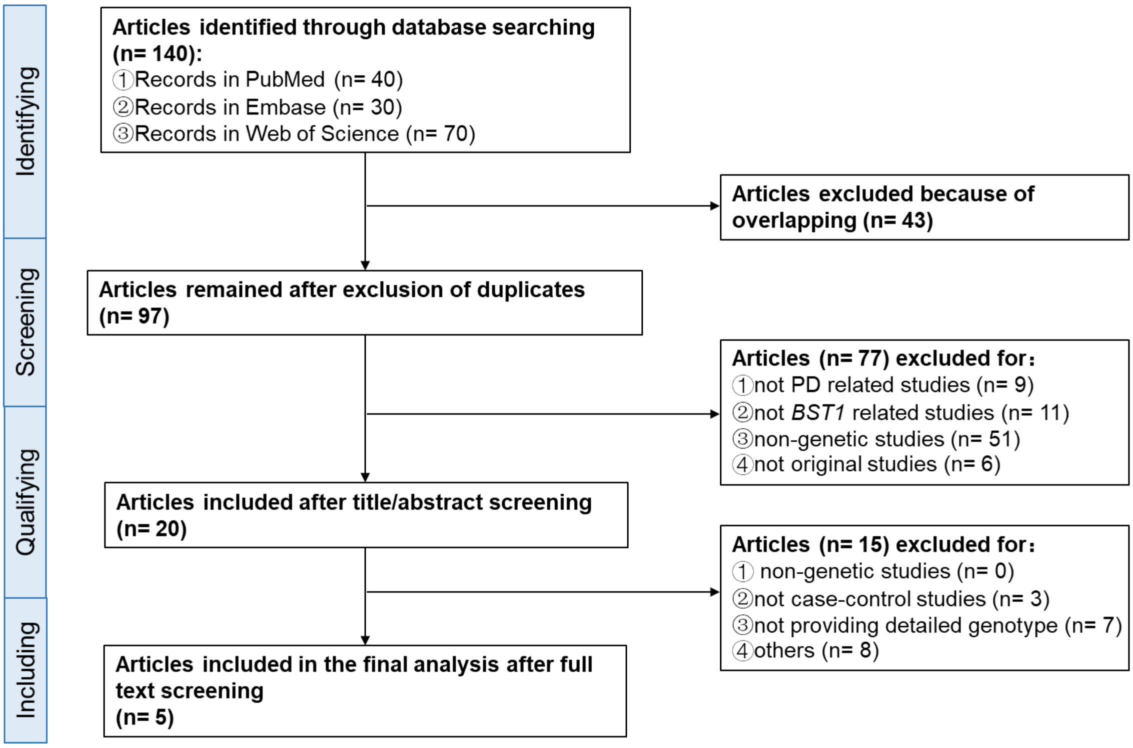

(L) The gene of HLA-DRB1

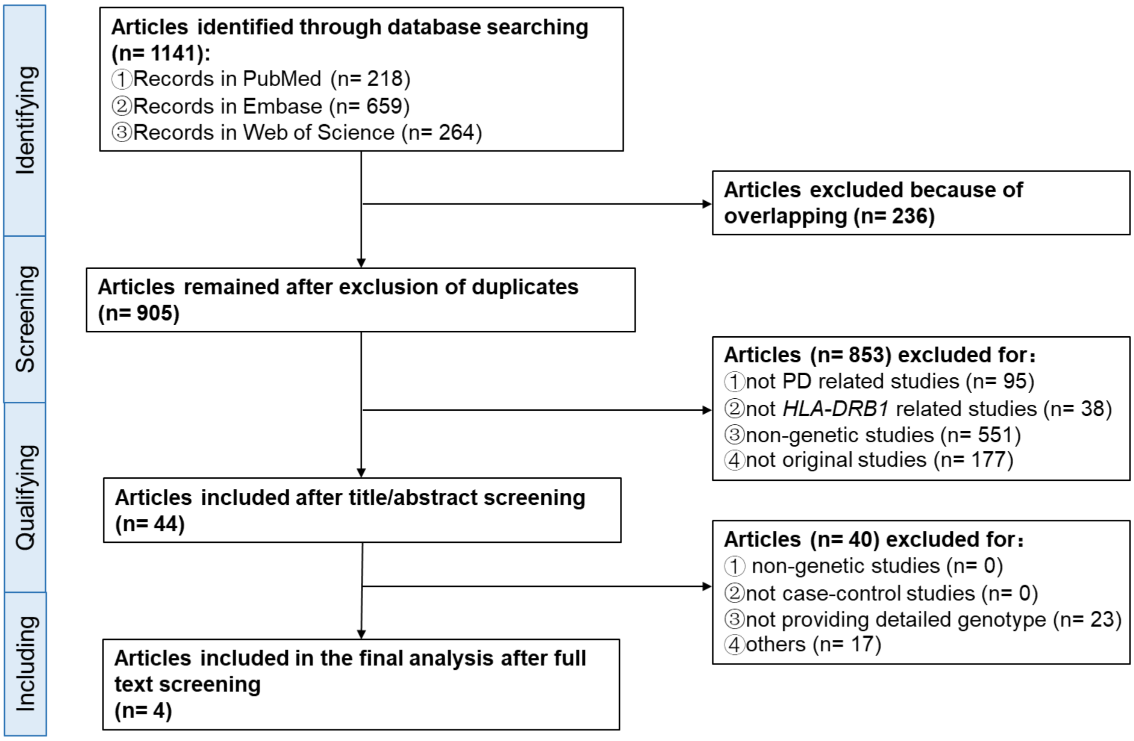

(M) The gene of CCDC62

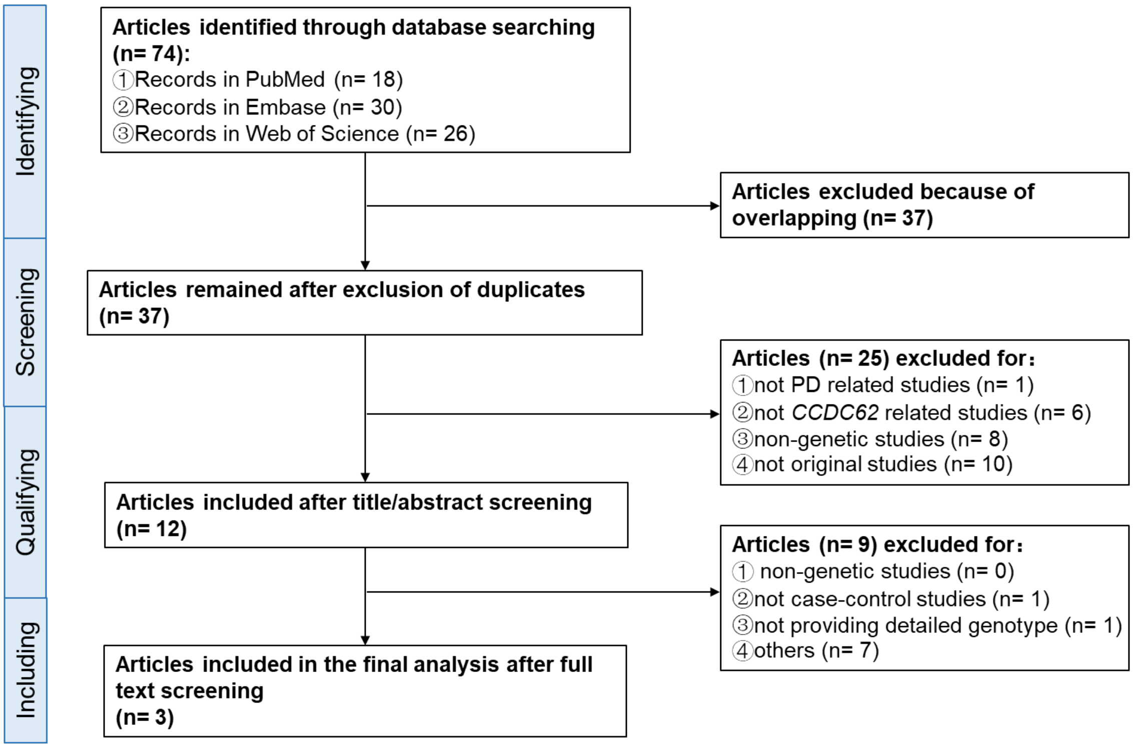

(N) The gene of HFE

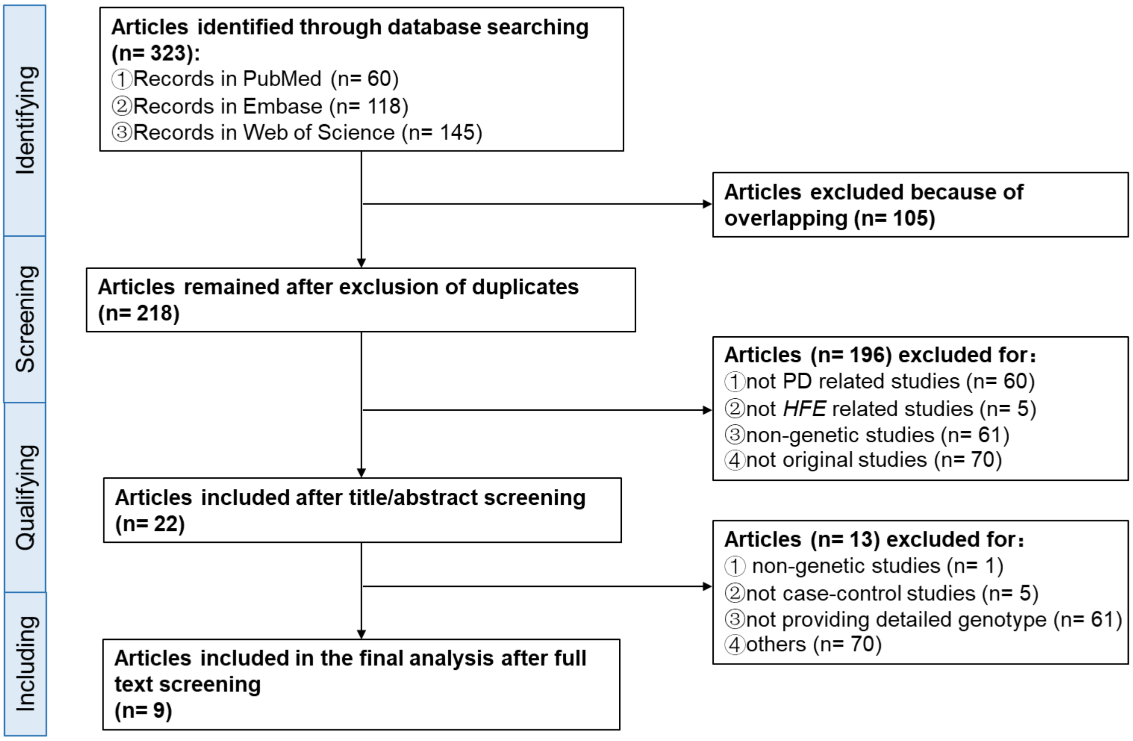

(O) The gene of MTHFR

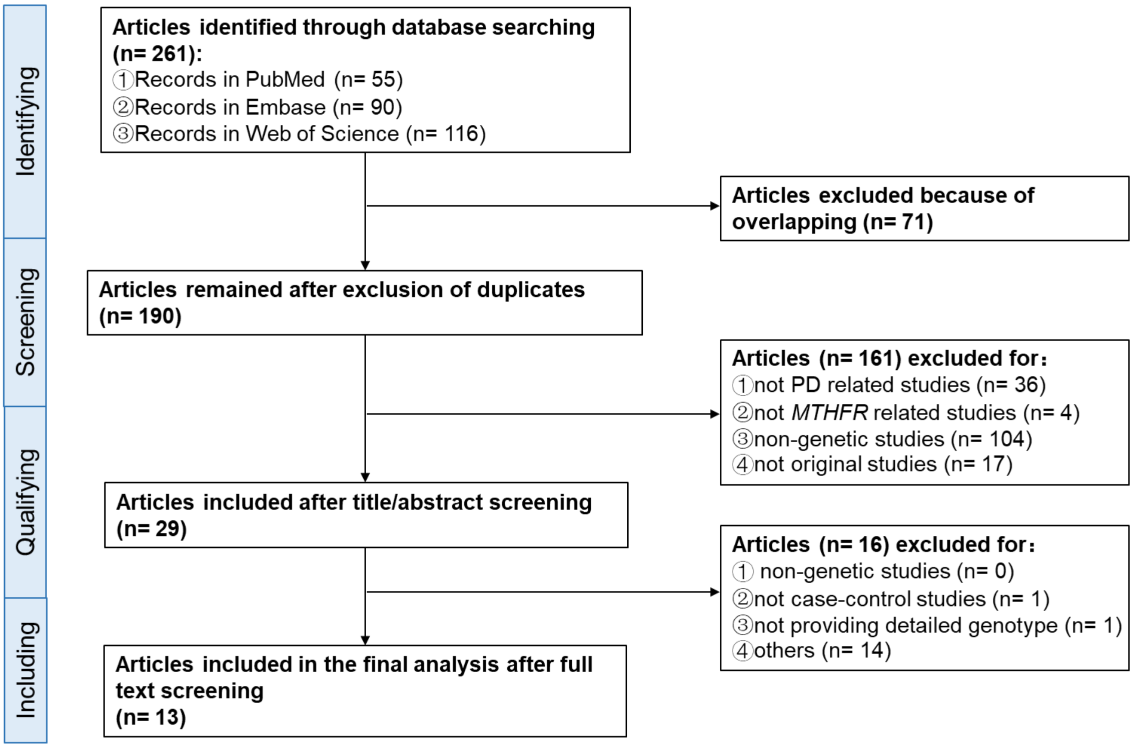

Supplementary Figure 2. Forest plots of the association between each candidate variant included in quantitative analysis and risk of PD.

（1）rs1800629, G-308A(MAF-A)

Study or Subgroup	Experimental Events	Experimental Total	Control Events	Control Total	Weight	Odds Ratio M-H, Fixed, 95% CI
2000Kruger, R.	89	474	57	354	15.9%	1.20 [0.84, 1.74]
2004Ross, O. A.	40	180	47	186	10.8%	0.84 [0.52, 1.37]
2007Wahner, A. D.	97	578	74	538	19.2%	1.26 [0.91, 1.76]
2007Wu, Y. R. (2)	90	738	67	652	18.8%	1.21 [0.87, 1.70]
2011Pascale, E.	37	292	49	312	12.4%	0.78 [0.49, 1.23]
2018Agliardi, C.	90	708	98	886	22.8%	1.17 [0.86, 1.59]
**Total (95% CI)**		**2970**		**2928**	**100.0%**	**1.12 [0.96, 1.30]**
Total events	443		392			

Heterogeneity: Chi² = 4.68, df = 5 (P = 0.46); I² = 0%
Test for overall effect: Z = 1.48 (P = 0.14)

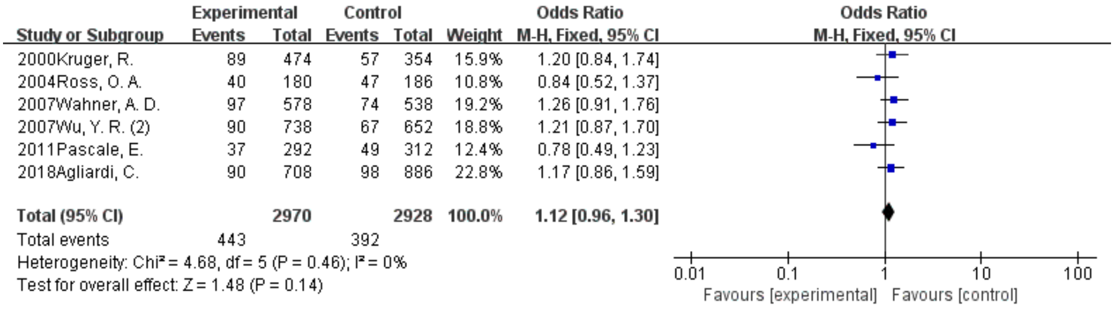

（2）rs1800629, G-308A(GA+AA)

Study or Subgroup	Experimental Events	Experimental Total	Control Events	Control Total	Weight	Odds Ratio M-H, Fixed, 95% CI
2000Kruger, R.	84	237	50	177	14.1%	1.39 [0.91, 2.13]
2004Ross, O. A.	30	90	39	93	9.7%	0.69 [0.38, 1.26]
2007Wahner, A. D.	84	289	68	269	19.0%	1.21 [0.83, 1.76]
2007Wu, Y. R. (2)	87	369	64	326	19.7%	1.26 [0.88, 1.82]
2011Pascale, E.	31	146	47	156	13.6%	0.63 [0.37, 1.06]
2018Agliardi, C.	85	354	93	443	23.9%	1.19 [0.85, 1.66]
**Total (95% CI)**		**1485**		**1464**	**100.0%**	**1.11 [0.94, 1.31]**
Total events	401		361			

Heterogeneity: Chi² = 8.96, df = 5 (P = 0.11); I² = 44%
Test for overall effect: Z = 1.25 (P = 0.21)

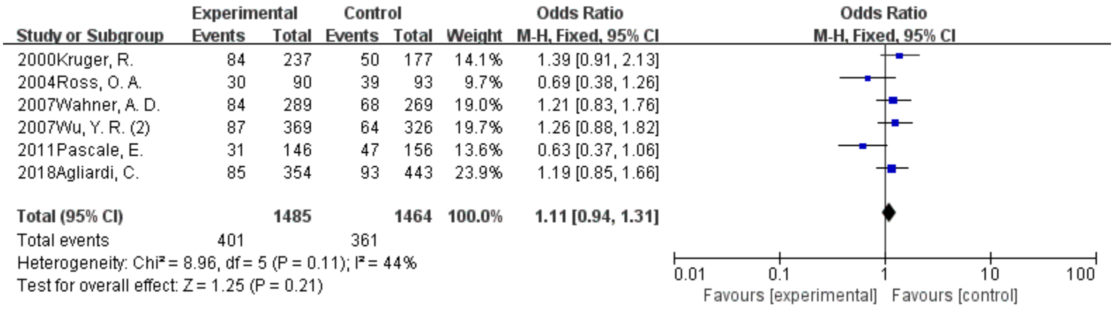

（3）rs1800629, G-308A(AA)

Study or Subgroup	Experimental Events	Experimental Total	Control Events	Control Total	Weight	Odds Ratio M-H, Fixed, 95% CI
2000Kruger, R.	5	237	7	177	26.0%	0.52 [0.16, 1.68]
2004Ross, O. A.	10	90	8	93	23.2%	1.33 [0.50, 3.53]
2007Wahner, A. D.	13	289	6	269	19.7%	2.06 [0.77, 5.51]
2007Wu, Y. R. (2)	3	369	3	326	10.5%	0.88 [0.18, 4.40]
2011Pascale, E.	6	146	2	156	6.1%	3.30 [0.66, 16.62]
2018Agliardi, C.	5	354	5	443	14.5%	1.26 [0.36, 4.37]
**Total (95% CI)**		**1485**		**1464**	**100.0%**	**1.33 [0.83, 2.13]**
Total events	42		31			

Heterogeneity: Chi² = 4.71, df = 5 (P = 0.45); I² = 0%
Test for overall effect: Z = 1.18 (P = 0.24)

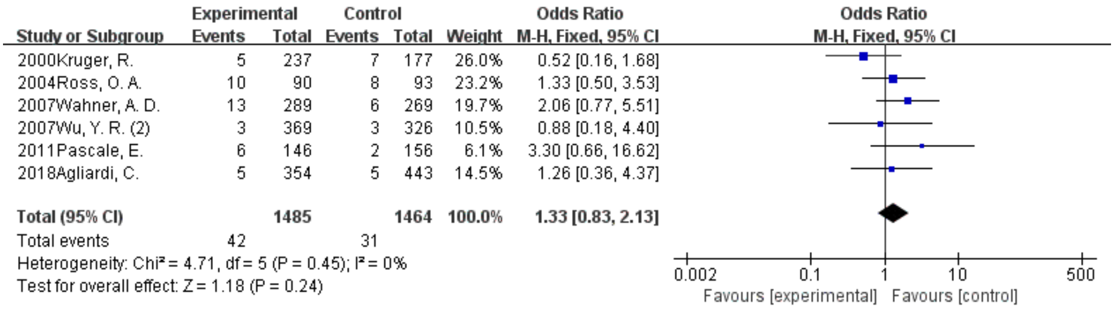

（4）rs1799964, T-1031C(MAF-C)

Study or Subgroup	Experimental Events	Experimental Total	Control Events	Control Total	Weight	Odds Ratio M-H, Random, 95% CI
2001Nishimura, M.	71	344	40	314	33.1%	1.78 [1.17, 2.72]
2007Wu, Y. R. (2)	136	738	106	652	43.5%	1.16 [0.88, 1.54]
2008Infante, J.	23	388	24	340	23.4%	0.83 [0.46, 1.50]
**Total (95% CI)**		**1470**		**1306**	**100.0%**	**1.24 [0.85, 1.79]**
Total events	230		170			

Heterogeneity: Tau² = 0.06; Chi² = 4.80, df = 2 (P = 0.09); I² = 58%
Test for overall effect: Z = 1.12 (P = 0.26)

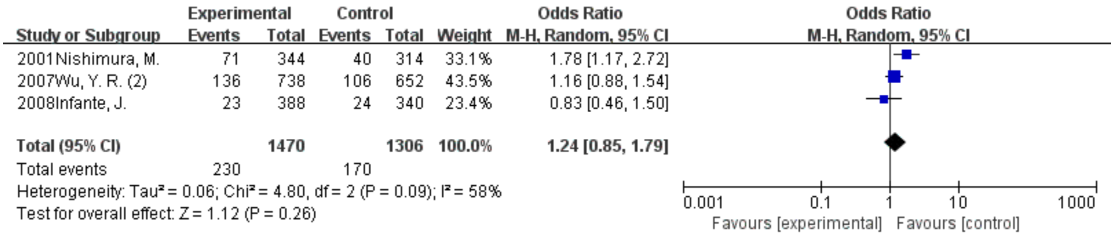

（5）rs1799964, T-1031C(CC)

Study or Subgroup	Experimental Events	Experimental Total	Control Events	Control Total	Weight	Odds Ratio M-H, Fixed, 95% CI
2001Nishimura, M.	11	172	3	157	24.8%	3.51 [0.96, 12.81]
2007Wu, Y. R. (2)	27	369	8	326	66.4%	3.14 [1.40, 7.01]
2008Infante, J.	3	194	1	170	8.8%	2.65 [0.27, 25.76]
**Total (95% CI)**		**735**		**653**	**100.0%**	**3.19 [1.66, 6.13]**
Total events	41		12			

Heterogeneity: Chi² = 0.05, df = 2 (P = 0.98); I² = 0%
Test for overall effect: Z = 3.47 (P = 0.0005)

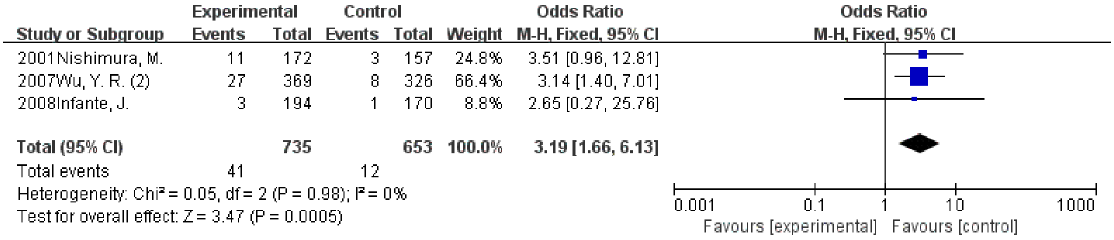

（6）rs1799964, T-1031C(TC+CC)

Study or Subgroup	Experimental Events	Experimental Total	Control Events	Control Total	Weight	Odds Ratio M-H, Random, 95% CI
2001Nishimura, M.	60	172	37	157	32.9%	1.74 [1.07, 2.82]
2007Wu, Y. R. (2)	109	369	98	326	41.5%	0.98 [0.70, 1.35]
2008Infante, J.	20	194	23	170	25.6%	0.73 [0.39, 1.39]
**Total (95% CI)**		**735**		**653**	**100.0%**	**1.10 [0.70, 1.72]**
Total events	189		158			

Heterogeneity: $Tau^2 = 0.10$; $Chi^2 = 5.48$, df = 2 (P = 0.06); $I^2 = 64\%$
Test for overall effect: Z = 0.40 (P = 0.69)

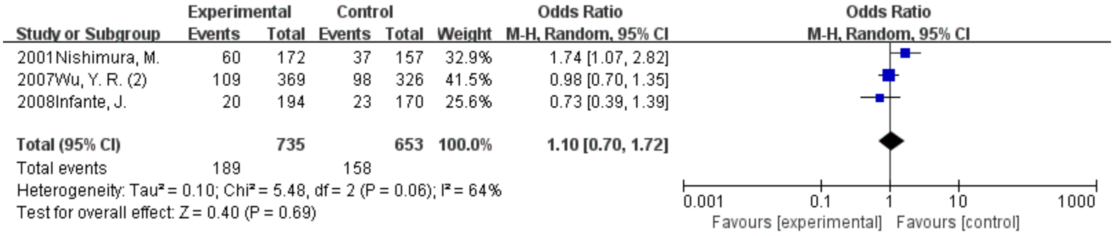

（7）rs1800587, C-889T（MAF-T）

Study or Subgroup	Experimental Events	Experimental Total	Control Events	Control Total	Weight	Odds Ratio M-H, Fixed, 95% CI
2000Nishimura, M.	26	244	25	224	4.2%	0.95 [0.53, 1.70]
2001Dodel, R. C.	100	402	87	394	11.8%	1.17 [0.84, 1.62]
2002Mattila, K. M.	28	104	51	146	5.6%	0.69 [0.40, 1.19]
2002McGeer, P. L.	77	200	66	200	7.3%	1.27 [0.84, 1.91]
2002Schulte, T.	157	514	145	520	17.9%	1.14 [0.87, 1.49]
2004Moller, J. C.	102	352	96	340	12.4%	1.04 [0.75, 1.44]
2007Wu, Y. R. (1)	79	986	56	776	10.3%	1.12 [0.78, 1.60]
2008Infante, J.	108	390	85	340	11.8%	1.15 [0.83, 1.60]
2008Zhou, Y. T.	85	1066	113	1060	18.7%	0.73 [0.54, 0.98]
**Total (95% CI)**		**4258**		**4000**	**100.0%**	**1.03 [0.91, 1.16]**
Total events	762		724			

Heterogeneity: $Chi^2 = 10.28$, df = 8 (P = 0.25); $I^2 = 22\%$
Test for overall effect: Z = 0.47 (P = 0.64)

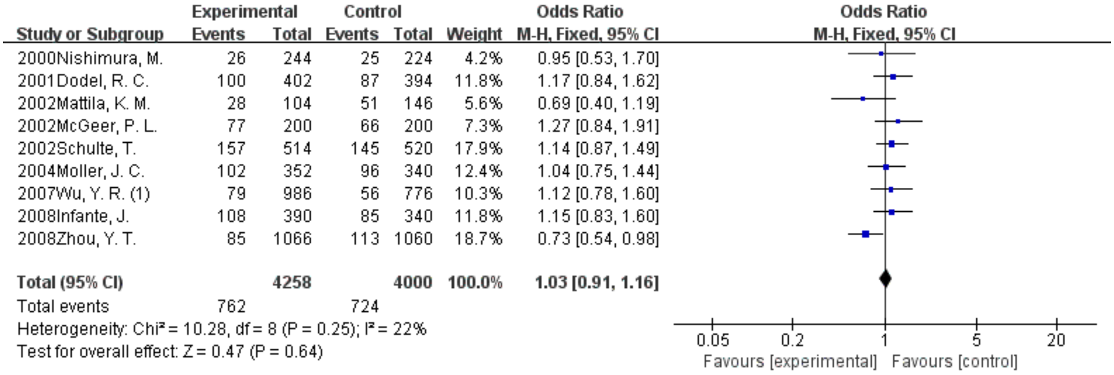

（8）rs1800587, C-889T（CT+TT）

Study or Subgroup	Experimental Events	Experimental Total	Control Events	Control Total	Weight	Odds Ratio M-H, Fixed, 95% CI
2000Nishimura, M.	25	122	25	112	5.3%	0.90 [0.48, 1.68]
2001Dodel, R. C.	93	201	82	197	11.4%	1.21 [0.81, 1.80]
2002Mattila, K. M.	24	52	40	73	4.6%	0.71 [0.35, 1.44]
2002McGeer, P. L.	64	100	63	100	5.8%	1.04 [0.59, 1.86]
2002Schulte, T.	132	257	119	260	14.7%	1.25 [0.89, 1.77]
2004Moller, J. C.	93	176	91	170	11.2%	0.97 [0.64, 1.48]
2007Wu, Y. R. (1)	76	493	54	388	13.1%	1.13 [0.77, 1.64]
2008Infante, J.	93	195	73	170	10.4%	1.21 [0.80, 1.83]
2008Zhou, Y. T.	82	533	108	530	23.5%	0.71 [0.52, 0.97]
**Total (95% CI)**		**2129**		**2000**	**100.0%**	**1.01 [0.88, 1.16]**
Total events	682		655			

Heterogeneity: $Chi^2 = 9.22$, df = 8 (P = 0.32); $I^2 = 13\%$
Test for overall effect: Z = 0.17 (P = 0.87)

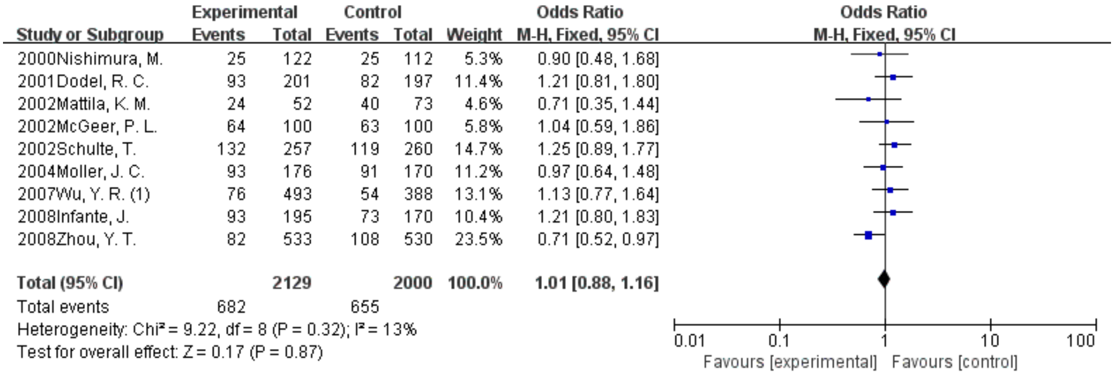

（9）rs1800587, C-889T（TT）

Study or Subgroup	Experimental Events	Experimental Total	Control Events	Control Total	Weight	Odds Ratio M-H, Fixed, 95% CI
2000Nishimura, M.	1	122	0	112	0.8%	2.78 [0.11, 68.89]
2001Dodel, R. C.	7	201	5	197	7.7%	1.39 [0.43, 4.44]
2002Mattila, K. M.	4	52	11	73	13.3%	0.47 [0.14, 1.57]
2002McGeer, P. L.	13	100	3	100	4.1%	4.83 [1.33, 17.52]
2002Schulte, T.	25	257	26	260	36.7%	0.97 [0.54, 1.73]
2004Moller, J. C.	9	176	5	170	7.6%	1.78 [0.58, 5.42]
2007Wu, Y. R. (1)	3	493	2	388	3.5%	1.18 [0.20, 7.11]
2008Infante, J.	15	195	12	170	18.6%	1.10 [0.50, 2.41]
2008Zhou, Y. T.	3	533	5	530	7.8%	0.59 [0.14, 2.50]
**Total (95% CI)**		**2129**		**2000**	**100.0%**	**1.17 [0.84, 1.64]**
Total events	80		69			

Heterogeneity: $Chi^2 = 9.05$, df = 8 (P = 0.34); $I^2 = 12\%$
Test for overall effect: Z = 0.93 (P = 0.35)

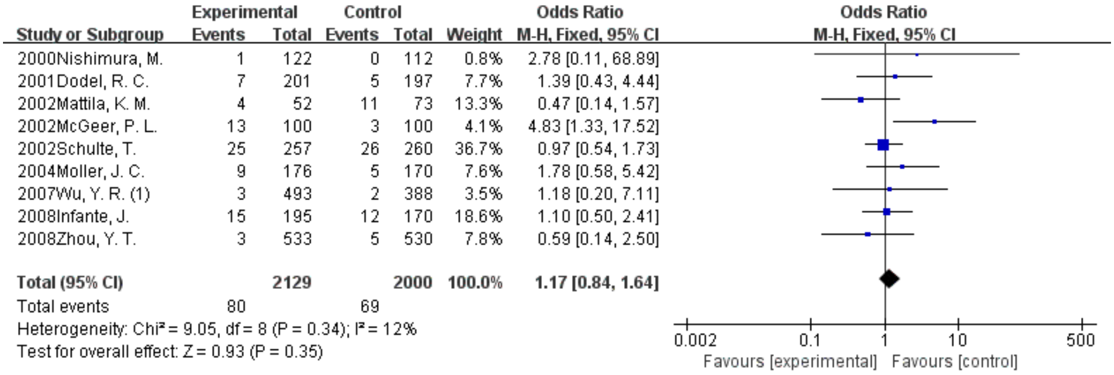

（10）rs16944, C-511T(MAF-T)

Study or Subgroup	Experimental Events	Experimental Total	Control Events	Control Total	Weight	Odds Ratio M-H, Random, 95% CI
2000Nishimura, M.	117	242	104	224	11.5%	1.08 [0.75, 1.55]
2002Mattila, K. M.	29	104	68	146	8.3%	0.44 [0.26, 0.76]
2002McGeer, P. L.	89	200	59	200	10.5%	1.92 [1.27, 2.89]
2002Schulte, T.	229	590	175	534	14.0%	1.30 [1.02, 1.66]
2005Nishimura, M.	359	722	248	514	14.4%	1.06 [0.85, 1.33]
2007Wahner, A. D.	231	578	176	538	14.0%	1.37 [1.07, 1.75]
2007Wu, Y. R. (1)	437	986	371	776	15.1%	0.87 [0.72, 1.05]
2011Pascale, E.	107	292	130	312	12.3%	0.81 [0.58, 1.12]
**Total (95% CI)**		**3714**		**3244**	**100.0%**	**1.05 [0.85, 1.31]**
Total events	1598		1331			

Heterogeneity: Tau² = 0.07; Chi² = 31.73, df = 7 (P < 0.0001); I² = 78%
Test for overall effect: Z = 0.47 (P = 0.64)

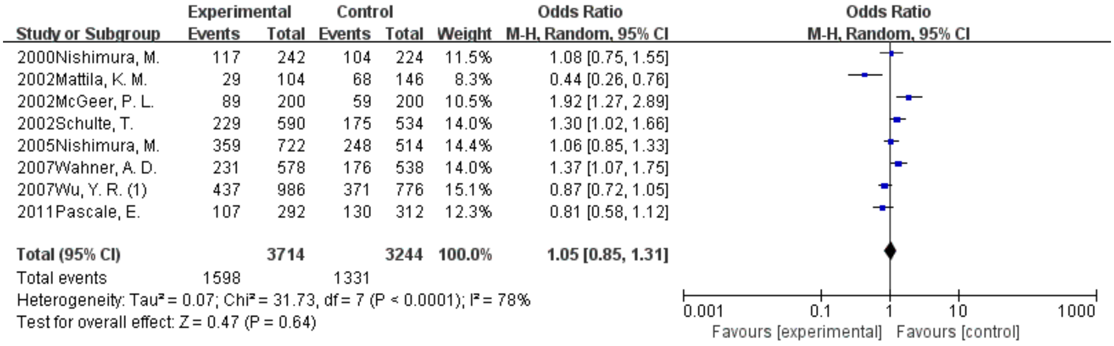

（11）rs16944, C-511T(CT+TT)

Study or Subgroup	Experimental Events	Experimental Total	Control Events	Control Total	Weight	Odds Ratio M-H, Fixed, 95% CI
1999Grasbon-Frodl, E. M.	48	88	47	84	25.3%	0.94 [0.52, 1.72]
2008Singh, M.	68	140	95	200	46.6%	1.04 [0.68, 1.61]
2010Wang, V. C.	72	590	19	222	28.1%	1.49 [0.87, 2.53]
**Total (95% CI)**		**818**		**506**	**100.0%**	**1.14 [0.86, 1.53]**
Total events	188		161			

Heterogeneity: Chi² = 1.49, df = 2 (P = 0.48); I² = 0%
Test for overall effect: Z = 0.90 (P = 0.37)

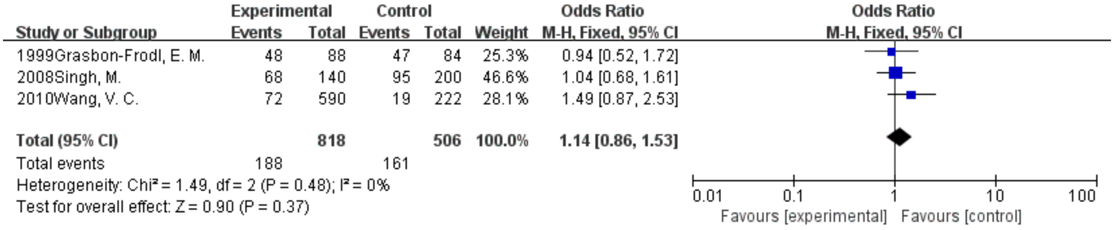

（12）rs16944, C-511T(TT)

Study or Subgroup	Experimental Events	Experimental Total	Control Events	Control Total	Weight	Odds Ratio M-H, Random, 95% CI
2000Nishimura, M.	31	121	23	112	13.0%	1.33 [0.72, 2.46]
2002Mattila, K. M.	2	52	19	73	5.3%	0.11 [0.03, 0.51]
2002McGeer, P. L.	22	100	3	100	6.9%	9.12 [2.63, 31.59]
2002Schulte, T.	44	295	35	267	14.7%	1.16 [0.72, 1.87]
2005Nishimura, M.	89	361	60	257	15.9%	1.07 [0.74, 1.56]
2007Wahner, A. D.	48	289	25	269	14.3%	1.94 [1.16, 3.25]
2007Wu, Y. R. (1)	90	493	88	388	16.4%	0.76 [0.55, 1.06]
2011Pascale, E.	26	146	30	156	13.4%	0.91 [0.51, 1.63]
**Total (95% CI)**		**1857**		**1622**	**100.0%**	**1.16 [0.77, 1.74]**
Total events	352		283			

Heterogeneity: Tau² = 0.24; Chi² = 30.09, df = 7 (P < 0.0001); I² = 77%
Test for overall effect: Z = 0.69 (P = 0.49)

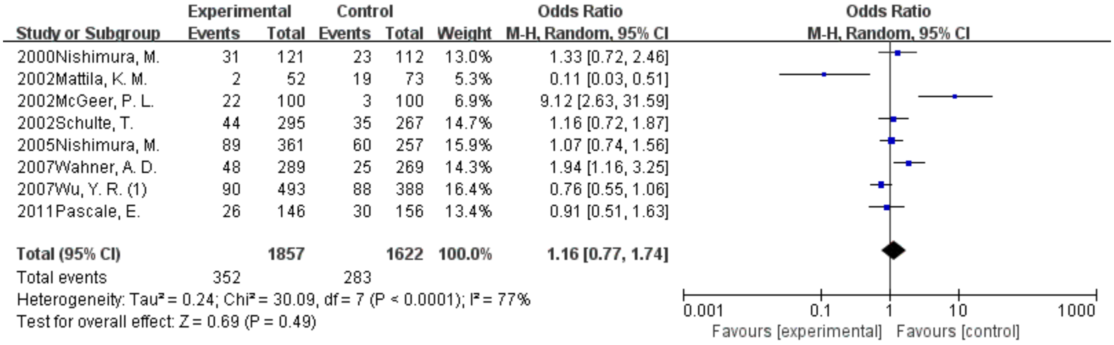

（13）rs1800795, G-174C(MAF-C)

Study or Subgroup	Experimental Events	Experimental Total	Control Events	Control Total	Weight	Odds Ratio M-H, Random, 95% CI
2004Ross, O. A.	84	180	72	186	18.7%	1.39 [0.91, 2.10]
2005Hakansson, A. (2)	231	516	318	616	27.7%	0.76 [0.60, 0.96]
2008Infante, J.	135	392	135	340	24.2%	0.80 [0.59, 1.08]
2012San Luciano, M.	206	760	383	1044	29.4%	0.64 [0.52, 0.79]
**Total (95% CI)**		**1848**		**2186**	**100.0%**	**0.82 [0.63, 1.06]**
Total events	656		908			

Heterogeneity: Tau² = 0.05; Chi² = 10.77, df = 3 (P = 0.01); I² = 72%
Test for overall effect: Z = 1.51 (P = 0.13)

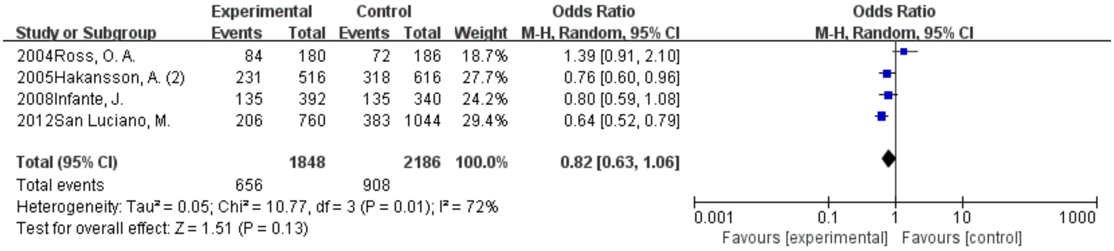

（14）rs1800795, G-174C(GC+CC)

Study or Subgroup	Experimental Events	Experimental Total	Control Events	Control Total	Weight	Odds Ratio M-H, Fixed, 95% CI
2004Ross, O. A.	64	90	61	93	6.2%	1.29 [0.69, 2.41]
2005Hakansson, A. (2)	180	258	240	308	23.8%	0.65 [0.45, 0.95]
2008Infante, J.	108	196	108	170	18.7%	0.70 [0.46, 1.07]
2012San Luciano, M.	175	380	314	522	51.3%	0.57 [0.43, 0.74]
**Total (95% CI)**		**924**		**1093**	**100.0%**	**0.66 [0.55, 0.79]**
Total events	527		723			

Heterogeneity: Chi² = 5.81, df = 3 (P = 0.12); I² = 48%
Test for overall effect: Z = 4.45 (P < 0.00001)

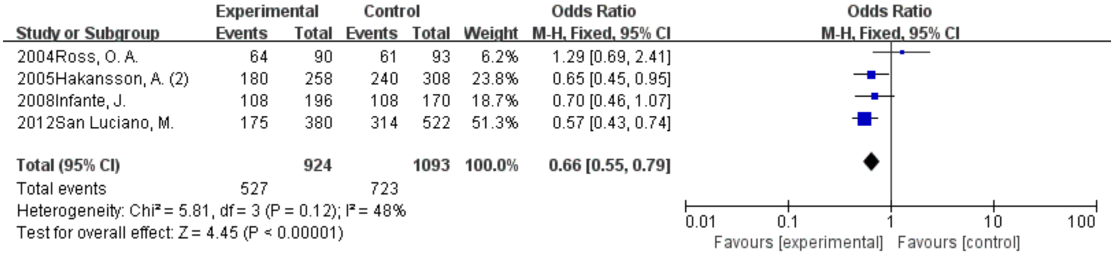

（15）rs1800795, G-174C(CC)

Study or Subgroup	Experimental Events	Experimental Total	Control Events	Control Total	Weight	Odds Ratio M-H, Random, 95% CI
2004Ross, O. A.	20	90	11	93	16.9%	2.13 [0.96, 4.75]
2005Hakansson, A. (2)	51	258	78	308	30.6%	0.73 [0.49, 1.08]
2008Infante, J.	27	196	27	170	23.7%	0.85 [0.47, 1.51]
2012San Luciano, M.	31	380	69	522	28.7%	0.58 [0.37, 0.91]
**Total (95% CI)**		**924**		**1093**	**100.0%**	**0.85 [0.55, 1.30]**
Total events	129		185			

Heterogeneity: Tau² = 0.11; Chi² = 7.86, df = 3 (P = 0.05); I² = 62%
Test for overall effect: Z = 0.75 (P = 0.45)

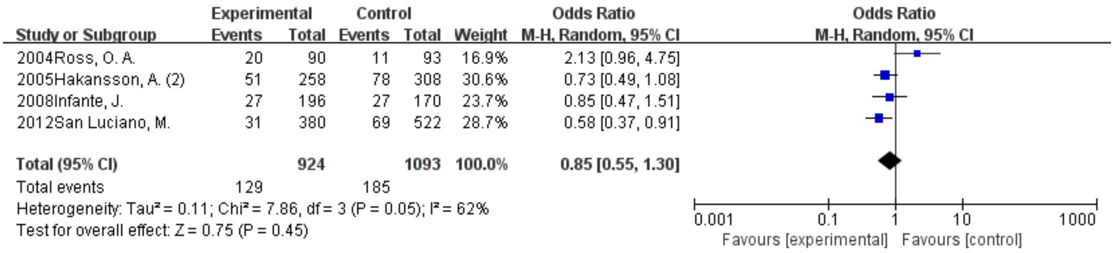

（16）rs1800896, -1082GA（MAF-G）

Study or Subgroup	Experimental Events	Experimental Total	Control Events	Control Total	Weight	Odds Ratio M-H, Fixed, 95% CI
2005Hakansson, A. (1)	242	518	296	614	22.4%	0.94 [0.75, 1.19]
2007Bialecka, M.	290	682	279	630	25.9%	0.93 [0.75, 1.16]
2008Bialecka, M.	278	632	261	600	23.3%	1.02 [0.81, 1.28]
2008Infante, J.	227	386	191	336	13.1%	1.08 [0.81, 1.46]
2011Pascale, E.	114	292	124	312	11.4%	0.97 [0.70, 1.35]
2013Nie, K.	38	604	26	588	3.8%	1.45 [0.87, 2.42]
**Total (95% CI)**		**3114**		**3080**	**100.0%**	**1.00 [0.90, 1.11]**
Total events	1189		1177			

Heterogeneity: Chi² = 3.04, df = 5 (P = 0.69); I² = 0%
Test for overall effect: Z = 0.02 (P = 0.98)

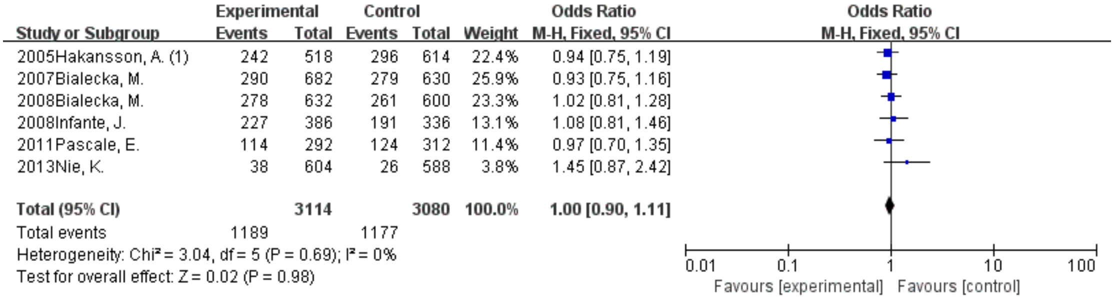

（17）rs1800896, -1082GA（GG）

Study or Subgroup	Experimental Events	Experimental Total	Control Events	Control Total	Weight	Odds Ratio M-H, Fixed, 95% CI
2005Hakansson, A. (1)	59	259	76	307	24.5%	0.90 [0.61, 1.32]
2007Bialecka, M.	61	341	63	315	24.5%	0.87 [0.59, 1.29]
2008Bialecka, M.	67	316	62	300	22.8%	1.03 [0.70, 1.52]
2008Infante, J.	66	193	55	168	17.6%	1.07 [0.69, 1.66]
2011Pascale, E.	19	146	27	156	10.3%	0.71 [0.38, 1.35]
2013Nie, K.	2	302	0	294	0.2%	4.90 [0.23, 102.50]
**Total (95% CI)**		**1557**		**1540**	**100.0%**	**0.94 [0.78, 1.14]**
Total events	274		283			

Heterogeneity: Chi² = 2.60, df = 5 (P = 0.76); I² = 0%
Test for overall effect: Z = 0.62 (P = 0.54)

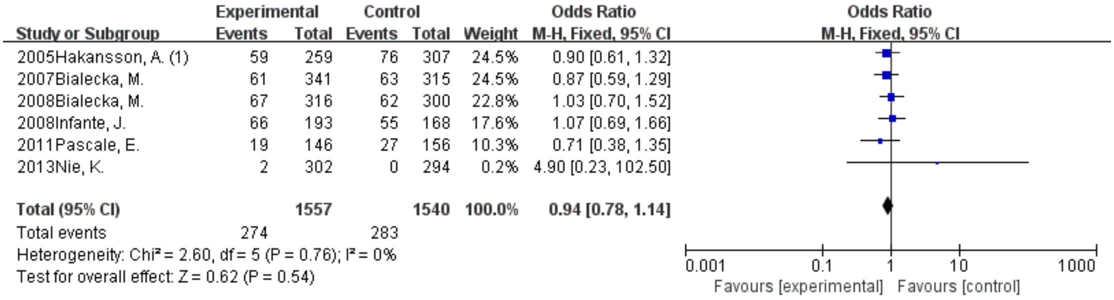

（18）rs1800896, -1082GA（AG+GG）

Study or Subgroup	Experimental Events	Experimental Total	Control Events	Control Total	Weight	Odds Ratio M-H, Fixed, 95% CI
2005Hakansson, A. (1)	183	259	220	307	21.0%	0.95 [0.66, 1.37]
2007Bialecka, M.	229	341	216	315	26.3%	0.94 [0.67, 1.30]
2008Bialecka, M.	211	316	199	300	24.2%	1.02 [0.73, 1.43]
2008Infante, J.	161	193	136	168	8.6%	1.18 [0.69, 2.03]
2011Pascale, E.	95	146	97	156	11.7%	1.13 [0.71, 1.81]
2013Nie, K.	36	302	26	294	8.3%	1.40 [0.82, 2.38]
**Total (95% CI)**		**1557**		**1540**	**100.0%**	**1.04 [0.88, 1.23]**
Total events	915		894			

Heterogeneity: Chi² = 2.14, df = 5 (P = 0.83); I² = 0%
Test for overall effect: Z = 0.49 (P = 0.62)

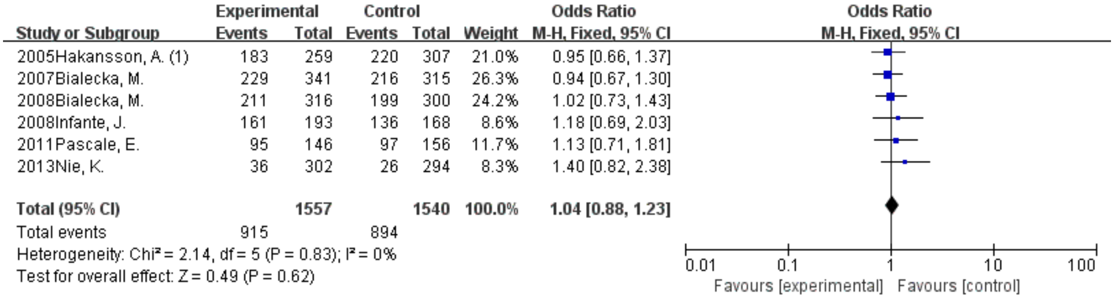

（19）rs1800871, -592CA(MAF-A)

Study or Subgroup	Experimental Events	Experimental Total	Control Events	Control Total	Weight	Odds Ratio M-H, Fixed, 95% CI
2007Bialecka, M.	188	682	150	630	21.4%	1.22 [0.95, 1.56]
2008Bialecka, M.	158	632	152	600	22.2%	0.98 [0.76, 1.27]
2012Li, D.	477	710	265	400	21.1%	1.04 [0.80, 1.35]
2016Liu, Z.	649	920	641	946	35.3%	1.14 [0.94, 1.39]
**Total (95% CI)**		**2944**		**2576**	**100.0%**	**1.10 [0.98, 1.24]**
Total events	1472		1208			

Heterogeneity: Chi² = 1.67, df = 3 (P = 0.64); I² = 0%
Test for overall effect: Z = 1.60 (P = 0.11)

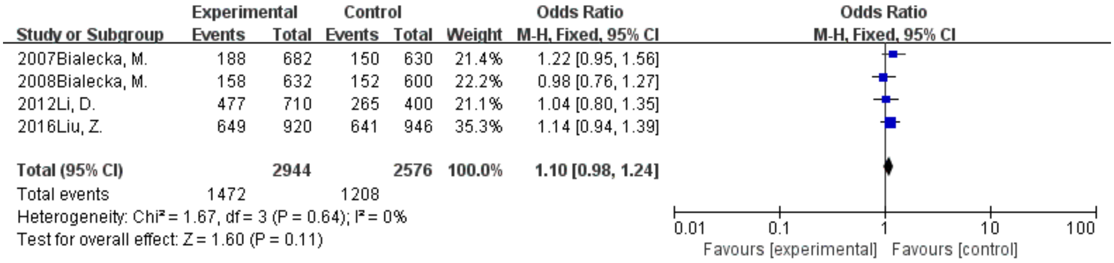

（20）rs1800871, -592CA(CA+AA)

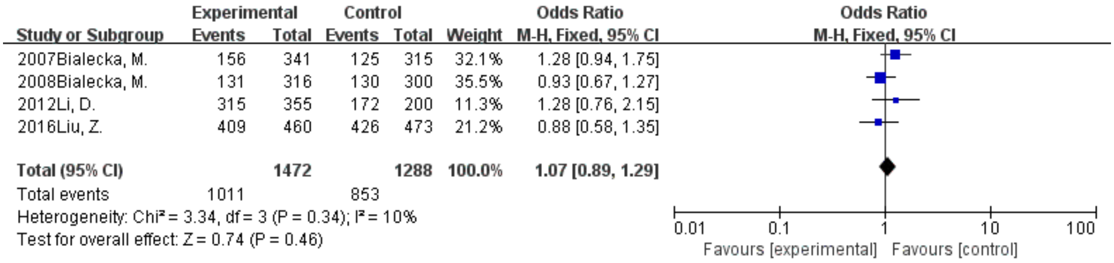

（21）rs1800871, -592CA(AA)

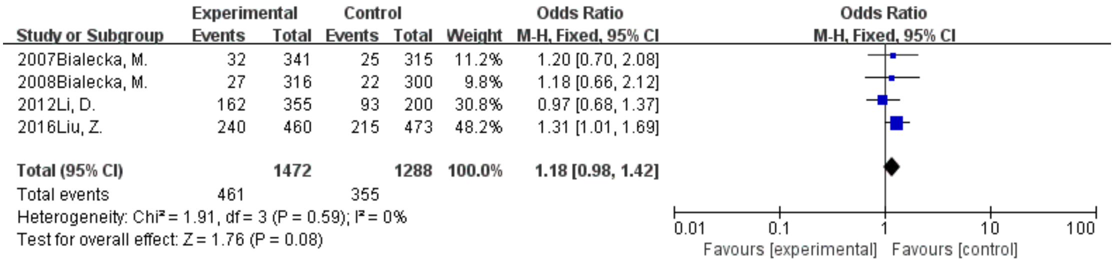

（22）rs2682826(MAF-T)

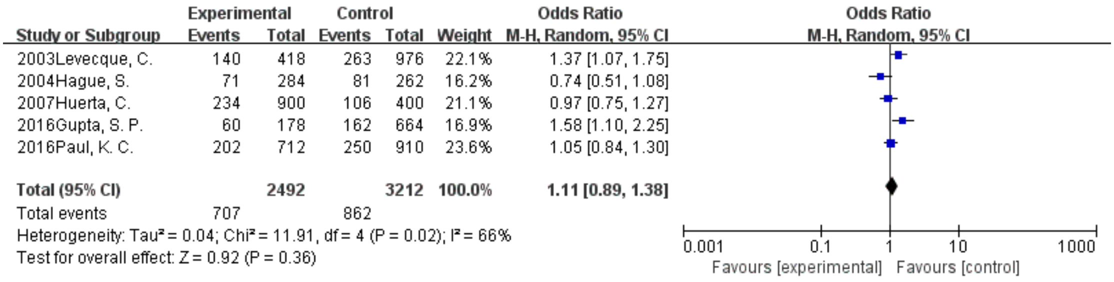

（23）rs2682826(CT+TT)

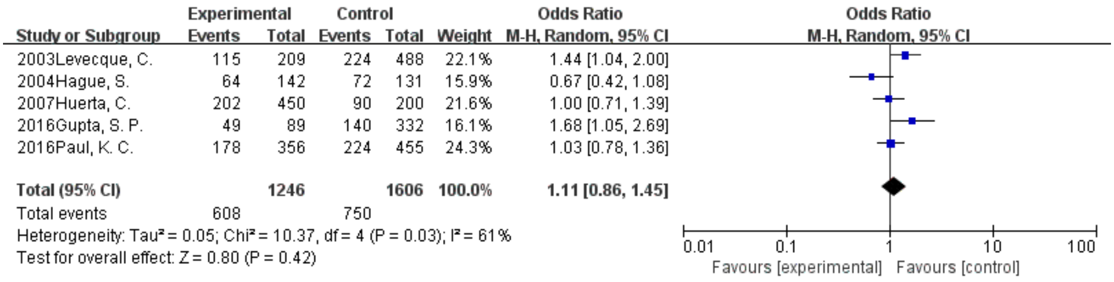

（24）rs2682826(TT)

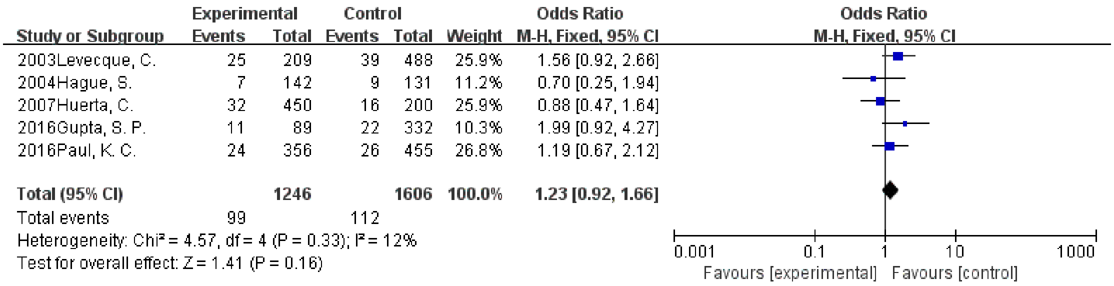

（25）rs1060826(MAF-A)

Study or Subgroup	Experimental Events	Experimental Total	Control Events	Control Total	Weight	Odds Ratio M-H, Random, 95% CI
2004Hague, S.	112	286	118	270	25.3%	0.83 [0.59, 1.16]
2007Huerta, C.	310	900	132	400	34.5%	1.07 [0.83, 1.37]
2016Paul, K. C.	284	712	296	858	40.2%	1.26 [1.03, 1.55]
**Total (95% CI)**		**1898**		**1528**	**100.0%**	**1.07 [0.86, 1.34]**
Total events	706		546			

Heterogeneity: Tau² = 0.02; Chi² = 4.41, df = 2 (P = 0.11); I² = 55%
Test for overall effect: Z = 0.60 (P = 0.55)

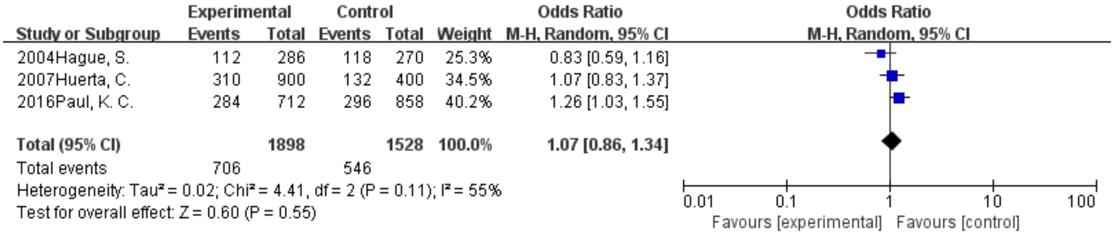

（26）rs1060826(GA+AA)

Study or Subgroup	Experimental Events	Experimental Total	Control Events	Control Total	Weight	Odds Ratio M-H, Fixed, 95% CI
2004Hague, S.	92	143	85	135	17.3%	1.06 [0.65, 1.73]
2007Huerta, C.	256	450	112	200	37.1%	1.04 [0.74, 1.45]
2016Paul, K. C.	227	356	250	429	45.6%	1.26 [0.94, 1.68]
**Total (95% CI)**		**949**		**764**	**100.0%**	**1.14 [0.94, 1.40]**
Total events	575		447			

Heterogeneity: Chi² = 0.85, df = 2 (P = 0.65); I² = 0%
Test for overall effect: Z = 1.31 (P = 0.19)

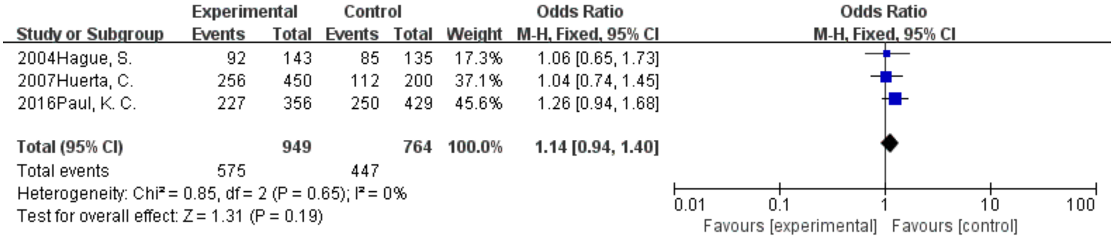

（27）rs1060826(AA)

Study or Subgroup	Experimental Events	Experimental Total	Control Events	Control Total	Weight	Odds Ratio M-H, Random, 95% CI
2004Hague, S.	20	143	33	135	30.9%	0.50 [0.27, 0.93]
2007Huerta, C.	54	450	20	200	32.9%	1.23 [0.71, 2.11]
2016Paul, K. C.	57	356	46	429	36.3%	1.59 [1.05, 2.41]
**Total (95% CI)**		**949**		**764**	**100.0%**	**1.02 [0.53, 1.96]**
Total events	131		99			

Heterogeneity: Tau² = 0.26; Chi² = 9.32, df = 2 (P = 0.009); I² = 79%
Test for overall effect: Z = 0.07 (P = 0.95)

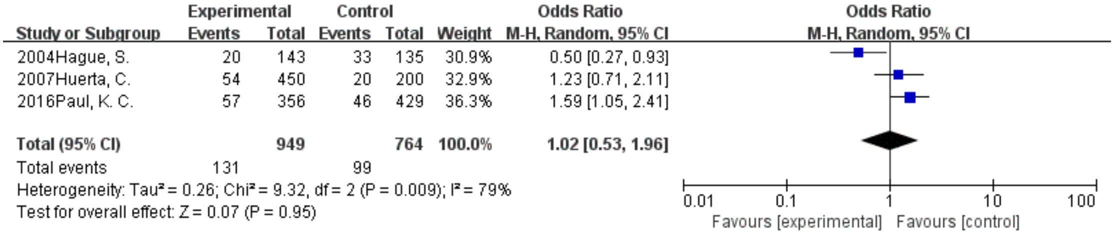

（28）rs4880(MAF-C)

Study or Subgroup	Experimental Events	Experimental Total	Control Events	Control Total	Weight	Odds Ratio M-H, Fixed, 95% CI
1999Grasbon-Frodl, E. M.	48	88	47	84	25.3%	0.94 [0.52, 1.72]
2008Singh, M.	68	140	95	200	46.6%	1.04 [0.68, 1.61]
2010Wang, V. C.	72	590	19	222	28.1%	1.49 [0.87, 2.53]
**Total (95% CI)**		**818**		**506**	**100.0%**	**1.14 [0.86, 1.53]**
Total events	188		161			

Heterogeneity: Chi² = 1.49, df = 2 (P = 0.48); I² = 0%
Test for overall effect: Z = 0.90 (P = 0.37)

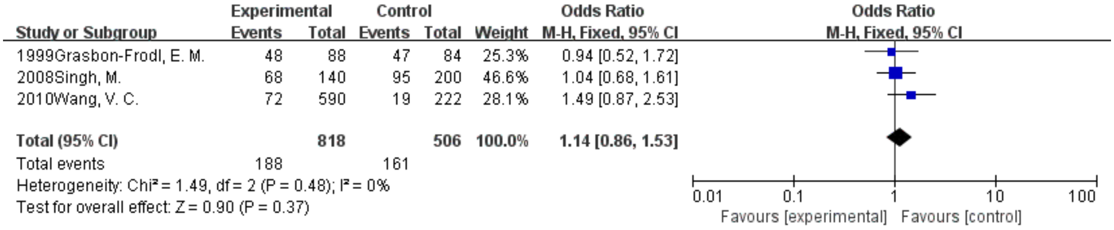

（29）rs4880(TC+CC)

Study or Subgroup	Experimental Events	Experimental Total	Control Events	Control Total	Weight	Odds Ratio M-H, Fixed, 95% CI
1999Grasbon-Frodl, E. M.	37	44	36	42	13.9%	0.88 [0.27, 2.88]
2008Singh, M.	55	70	84	100	35.2%	0.70 [0.32, 1.53]
2010Wang, V. C.	66	295	19	111	50.9%	1.40 [0.79, 2.45]
**Total (95% CI)**		**409**		**253**	**100.0%**	**1.08 [0.71, 1.64]**
Total events	158		139			

Heterogeneity: Chi² = 2.10, df = 2 (P = 0.35); I² = 5%
Test for overall effect: Z = 0.35 (P = 0.72)

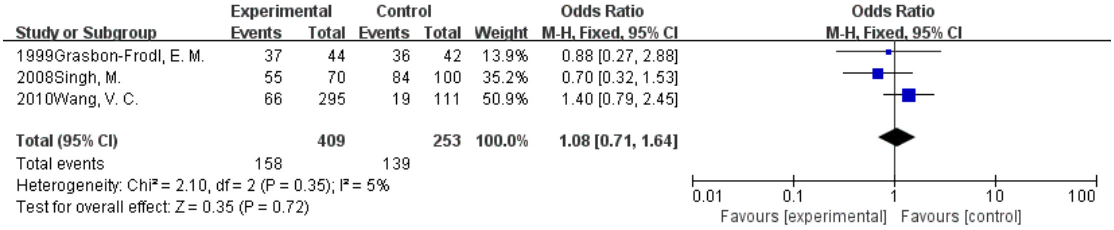

（30）rs4880(CC)

Study or Subgroup	Experimental Events	Experimental Total	Control Events	Control Total	Weight	Odds Ratio M-H, Fixed, 95% CI
1999Grasbon-Frodl, E. M.	11	44	11	42	51.1%	0.94 [0.36, 2.48]
2008Singh, M.	13	70	11	100	44.6%	1.85 [0.77, 4.40]
2010Wang, V. C.	6	295	0	111	4.3%	5.01 [0.28, 89.62]
**Total (95% CI)**		**409**		**253**	**100.0%**	**1.52 [0.82, 2.82]**
Total events	30		22			

Heterogeneity: Chi² = 1.79, df = 2 (P = 0.41); I² = 0%
Test for overall effect: Z = 1.33 (P = 0.19)

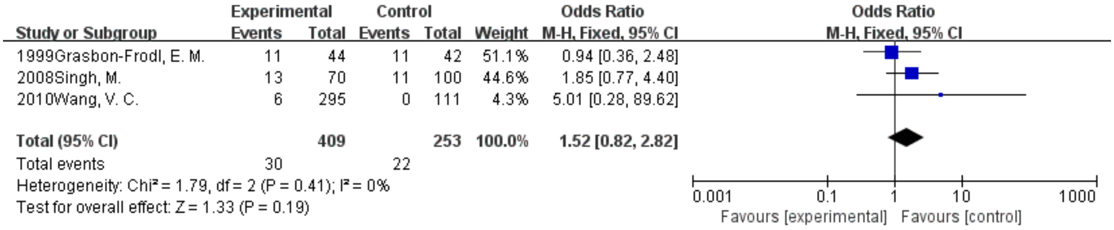

（31）rs6706649（MAF-A）

Study or Subgroup	Experimental Events	Experimental Total	Control Events	Control Total	Weight	Odds Ratio M-H, Fixed, 95% CI
2010von Otter, M. (i)	41	324	51	368	12.6%	0.90 [0.58, 1.40]
2010von Otter, M. (ii)	47	382	46	374	12.3%	1.00 [0.65, 1.54]
2013Chen, Y. C.	47	960	66	1052	18.1%	0.77 [0.52, 1.13]
2016Gui, Y.	168	1470	90	976	29.0%	1.27 [0.97, 1.66]
2017Ran, C.	102	960	104	968	28.0%	0.99 [0.74, 1.32]
**Total (95% CI)**		**4096**		**3738**	**100.0%**	**1.02 [0.88, 1.19]**
Total events	405		357			

Heterogeneity: Chi² = 4.97, df = 4 (P = 0.29); I² = 20%
Test for overall effect: Z = 0.26 (P = 0.79)

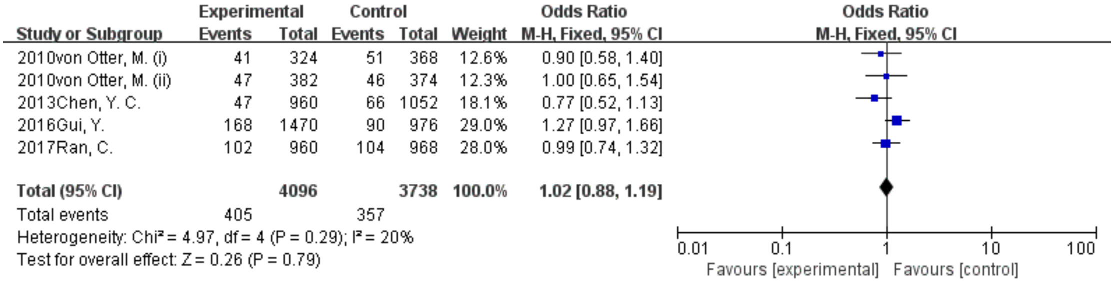

（32）rs6706649（GA+AA）

Study or Subgroup	Experimental Events	Experimental Total	Control Events	Control Total	Weight	Odds Ratio M-H, Fixed, 95% CI
2010von Otter, M. (i)	37	162	48	184	12.3%	0.84 [0.51, 1.37]
2010von Otter, M. (ii)	41	191	44	187	12.4%	0.89 [0.55, 1.44]
2013Chen, Y. C.	46	480	65	526	19.9%	0.75 [0.50, 1.12]
2016Gui, Y.	153	735	83	488	28.0%	1.28 [0.95, 1.72]
2017Ran, C.	95	480	97	484	27.5%	0.98 [0.72, 1.35]
**Total (95% CI)**		**2048**		**1869**	**100.0%**	**0.99 [0.84, 1.17]**
Total events	372		337			

Heterogeneity: Chi² = 5.40, df = 4 (P = 0.25); I² = 26%
Test for overall effect: Z = 0.10 (P = 0.92)

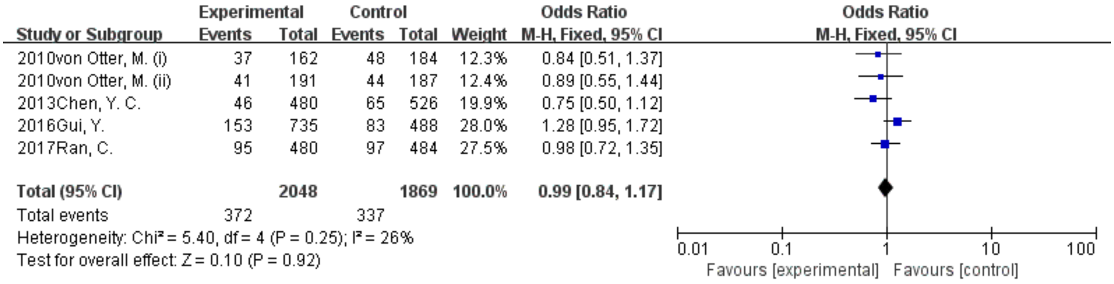

（33）rs6706649（AA）

Study or Subgroup	Experimental Events	Experimental Total	Control Events	Control Total	Weight	Odds Ratio M-H, Fixed, 95% CI
2010von Otter, M. (i)	4	162	3	184	13.2%	1.53 [0.34, 6.93]
2010von Otter, M. (ii)	6	191	2	187	9.4%	3.00 [0.60, 15.06]
2013Chen, Y. C.	1	480	1	526	4.6%	1.10 [0.07, 17.57]
2016Gui, Y.	15	735	7	488	39.7%	1.43 [0.58, 3.54]
2017Ran, C.	7	480	7	484	33.1%	1.01 [0.35, 2.90]
**Total (95% CI)**		**2048**		**1869**	**100.0%**	**1.44 [0.82, 2.52]**
Total events	33		20			

Heterogeneity: Chi² = 1.28, df = 4 (P = 0.87); I² = 0%
Test for overall effect: Z = 1.26 (P = 0.21)

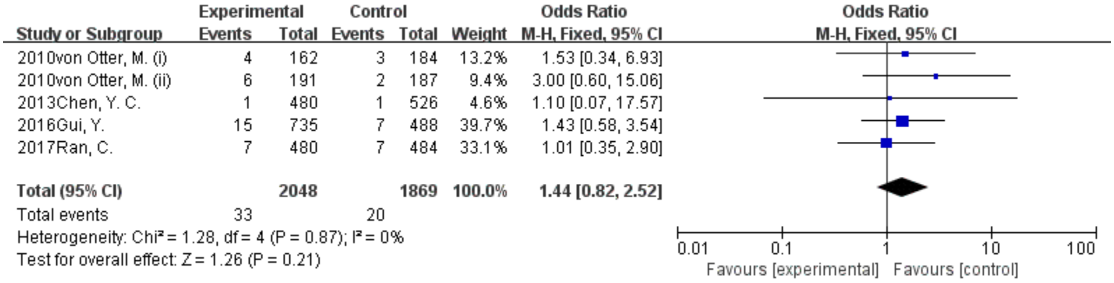

（34）rs6721961(MAF-A)

Study or Subgroup	Experimental Events	Experimental Total	Control Events	Control Total	Weight	Odds Ratio M-H, Fixed, 95% CI
2010von Otter, M. (i)	41	324	29	368	4.1%	1.69 [1.03, 2.80]
2010von Otter, M. (ii)	50	382	47	374	7.1%	1.05 [0.68, 1.61]
2013Chen, Y. C.	272	960	297	1052	35.1%	1.01 [0.83, 1.22]
2016Gui, Y.	327	1530	209	976	34.7%	1.00 [0.82, 1.21]
2017Ran, C.	114	956	126	976	19.0%	0.91 [0.70, 1.20]
**Total (95% CI)**		**4152**		**3746**	**100.0%**	**1.02 [0.91, 1.14]**
Total events	804		708			

Heterogeneity: Chi² = 4.66, df = 4 (P = 0.32); I² = 14%
Test for overall effect: Z = 0.28 (P = 0.78)

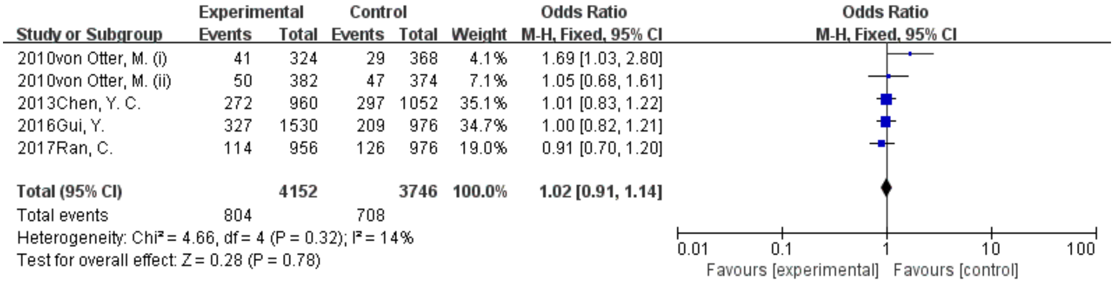

（35）rs6721961(AC+AA)

Study or Subgroup	Experimental Events	Experimental Total	Control Events	Control Total	Weight	Odds Ratio M-H, Fixed, 95% CI
2010von Otter, M. (i)	40	162	28	184	4.9%	1.83 [1.07, 3.13]
2010von Otter, M. (ii)	44	191	45	187	8.7%	0.94 [0.59, 1.52]
2013Chen, Y. C.	237	480	252	526	30.4%	1.06 [0.83, 1.36]
2016Gui, Y.	237	765	154	488	32.4%	0.97 [0.76, 1.24]
2017Ran, C.	108	478	123	488	23.5%	0.87 [0.64, 1.16]
**Total (95% CI)**		**2076**		**1873**	**100.0%**	**1.01 [0.88, 1.16]**
Total events	666		602			

Heterogeneity: Chi² = 6.01, df = 4 (P = 0.20); I² = 33%
Test for overall effect: Z = 0.20 (P = 0.84)

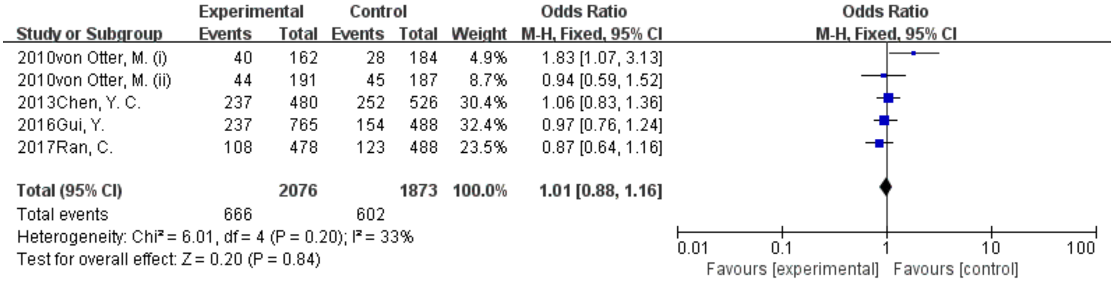

（36）rs6721961(AA)

Study or Subgroup	Experimental Events	Experimental Total	Control Events	Control Total	Weight	Odds Ratio M-H, Fixed, 95% CI
2010von Otter, M. (i)	1	162	1	184	0.9%	1.14 [0.07, 18.32]
2010von Otter, M. (ii)	6	191	2	187	1.9%	3.00 [0.60, 15.06]
2013Chen, Y. C.	35	480	45	526	38.0%	0.84 [0.53, 1.33]
2016Gui, Y.	90	765	55	488	56.5%	1.05 [0.73, 1.50]
2017Ran, C.	6	478	3	488	2.8%	2.06 [0.51, 8.26]
**Total (95% CI)**		**2076**		**1873**	**100.0%**	**1.04 [0.79, 1.35]**
Total events	138		106			

Heterogeneity: Chi² = 3.40, df = 4 (P = 0.49); I² = 0%
Test for overall effect: Z = 0.26 (P = 0.80)

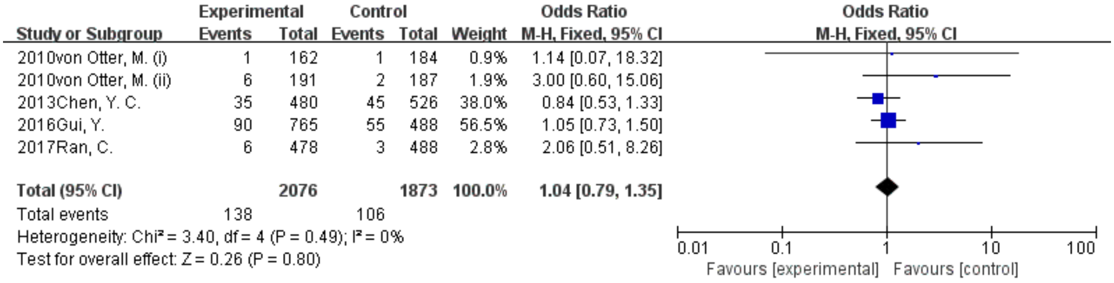

（37）rs35652124 (MAF-G)

Study or Subgroup	Experimental Events	Experimental Total	Control Events	Control Total	Weight	Odds Ratio M-H, Fixed, 95% CI
2010von Otter, M. (i)	88	324	115	368	9.0%	0.82 [0.59, 1.14]
2010von Otter, M. (ii)	129	382	101	374	7.8%	1.38 [1.01, 1.88]
2013Chen, Y. C.	488	960	539	1052	29.1%	0.98 [0.83, 1.17]
2016Gui, Y.	532	1530	342	978	31.3%	0.99 [0.84, 1.17]
2017Ran, C.	309	956	294	964	22.8%	1.09 [0.90, 1.32]
**Total (95% CI)**		**4152**		**3736**	**100.0%**	**1.03 [0.93, 1.13]**
Total events	1546		1391			

Heterogeneity: Chi² = 5.96, df = 4 (P = 0.20); I² = 33%
Test for overall effect: Z = 0.54 (P = 0.59)

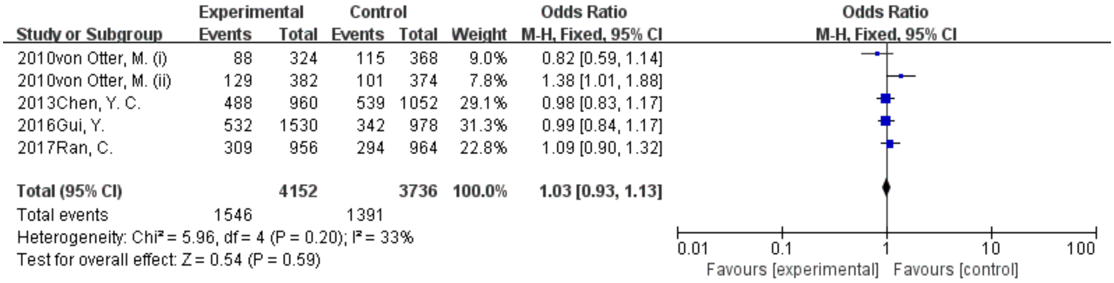

（38）rs35652124 (AG+GG)

Study or Subgroup	Experimental Events	Experimental Total	Control Events	Control Total	Weight	Odds Ratio M-H, Random, 95% CI
2010von Otter, M. (i)	73	162	102	184	14.3%	0.66 [0.43, 1.01]
2010von Otter, M. (ii)	103	191	84	187	15.1%	1.44 [0.96, 2.15]
2013Chen, Y. C.	361	480	412	526	21.2%	0.84 [0.63, 1.13]
2016Gui, Y.	400	765	254	489	25.7%	1.01 [0.81, 1.27]
2017Ran, C.	263	478	252	482	23.8%	1.12 [0.87, 1.44]
**Total (95% CI)**		**2076**		**1868**	**100.0%**	**0.99 [0.81, 1.21]**
Total events	1200		1104			

Heterogeneity: Tau² = 0.03; Chi² = 8.86, df = 4 (P = 0.06); I² = 55%
Test for overall effect: Z = 0.12 (P = 0.91)

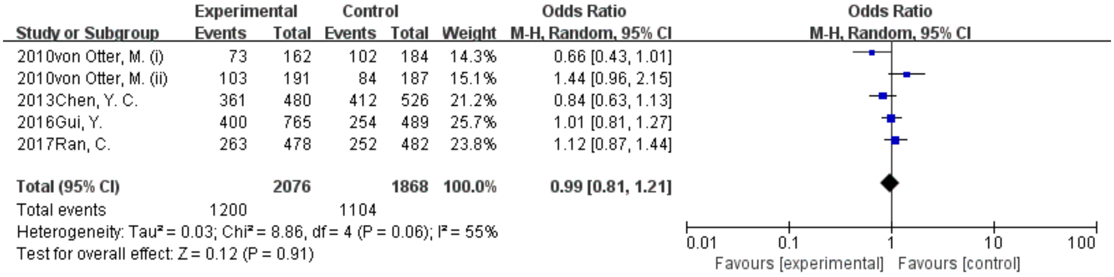

（39）rs35652124 (GG)

Study or Subgroup	Experimental Events	Experimental Total	Control Events	Control Total	Weight	Odds Ratio M-H, Fixed, 95% CI
2010von Otter, M. (i)	15	162	13	184	4.6%	1.34 [0.62, 2.91]
2010von Otter, M. (ii)	26	191	17	187	6.1%	1.58 [0.82, 3.01]
2013Chen, Y. C.	127	480	127	526	36.9%	1.13 [0.85, 1.50]
2016Gui, Y.	132	765	88	489	36.8%	0.95 [0.71, 1.28]
2017Ran, C.	46	478	42	482	15.6%	1.12 [0.72, 1.73]
**Total (95% CI)**		**2076**		**1868**	**100.0%**	**1.10 [0.92, 1.31]**
Total events	346		287			

Heterogeneity: Chi² = 2.41, df = 4 (P = 0.66); I² = 0%
Test for overall effect: Z = 1.06 (P = 0.29)

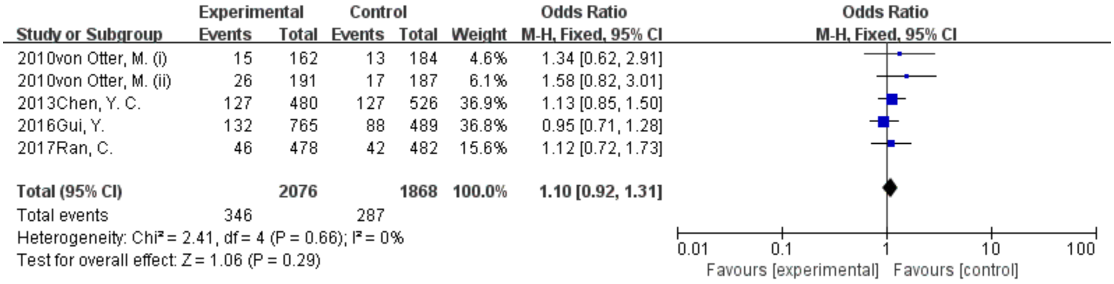

（40）rs2706110(MAF-A)

Study or Subgroup	Experimental Events	Experimental Total	Control Events	Control Total	Weight	Odds Ratio M-H, Random, 95% CI
2010von Otter, M. (i)	60	328	48	380	15.3%	1.55 [1.03, 2.34]
2010von Otter, M. (ii)	59	354	55	346	15.8%	1.06 [0.71, 1.58]
2015Todorovic, M.	472	2592	533	2680	37.2%	0.90 [0.78, 1.03]
2016Gui, Y.	359	1524	215	976	31.7%	1.09 [0.90, 1.32]
**Total (95% CI)**		**4798**		**4382**	**100.0%**	**1.06 [0.87, 1.30]**
Total events	950		851			

Heterogeneity: Tau² = 0.02; Chi² = 7.58, df = 3 (P = 0.06); I² = 60%
Test for overall effect: Z = 0.62 (P = 0.53)

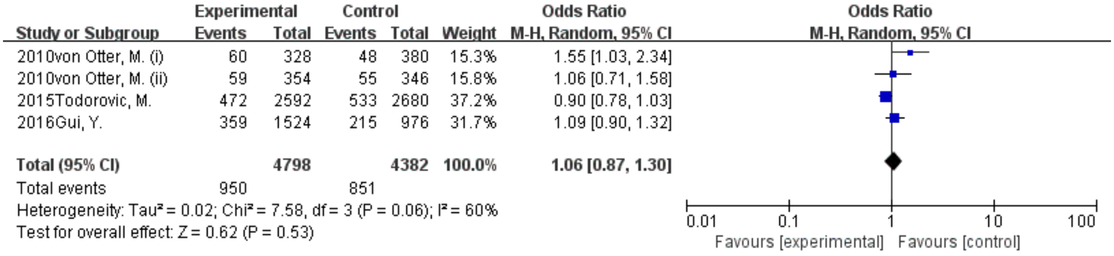

（41）rs2706110(GA+AA)

Study or Subgroup	Experimental Events	Experimental Total	Control Events	Control Total	Weight	Odds Ratio M-H, Random, 95% CI
2010von Otter, M. (i)	54	164	44	190	17.7%	1.63 [1.02, 2.60]
2010von Otter, M. (ii)	52	177	54	173	18.3%	0.92 [0.58, 1.45]
2015Todorovic, M.	422	1296	480	1340	34.2%	0.87 [0.74, 1.02]
2016Gui, Y.	296	762	168	488	29.9%	1.21 [0.95, 1.53]
**Total (95% CI)**		**2399**		**2191**	**100.0%**	**1.08 [0.83, 1.41]**
Total events	824		746			

Heterogeneity: Tau² = 0.05; Chi² = 9.89, df = 3 (P = 0.02); I² = 70%
Test for overall effect: Z = 0.57 (P = 0.57)

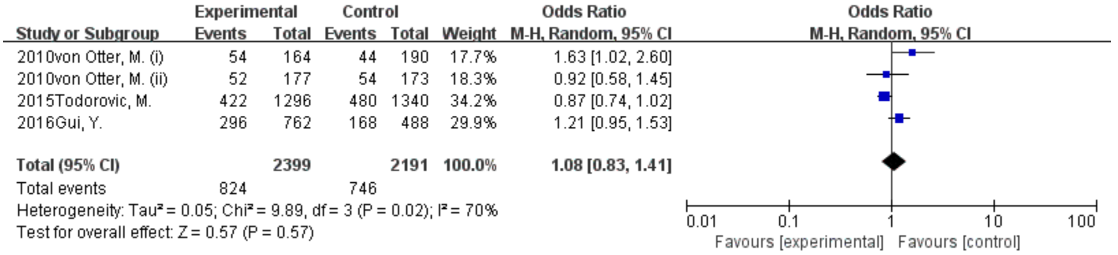

（42）rs2706110(AA)

Study or Subgroup	Experimental Events	Experimental Total	Control Events	Control Total	Weight	Odds Ratio M-H, Fixed, 95% CI
2010von Otter, M. (i)	6	164	4	190	3.3%	1.77 [0.49, 6.37]
2010von Otter, M. (ii)	7	177	1	173	0.9%	7.08 [0.86, 58.18]
2015Todorovic, M.	50	1296	53	1340	46.7%	0.97 [0.66, 1.45]
2016Gui, Y.	63	762	47	488	49.0%	0.85 [0.57, 1.26]
**Total (95% CI)**		**2399**		**2191**	**100.0%**	**0.99 [0.76, 1.30]**
Total events	126		105			

Heterogeneity: Chi² = 4.76, df = 3 (P = 0.19); I² = 37%
Test for overall effect: Z = 0.05 (P = 0.96)

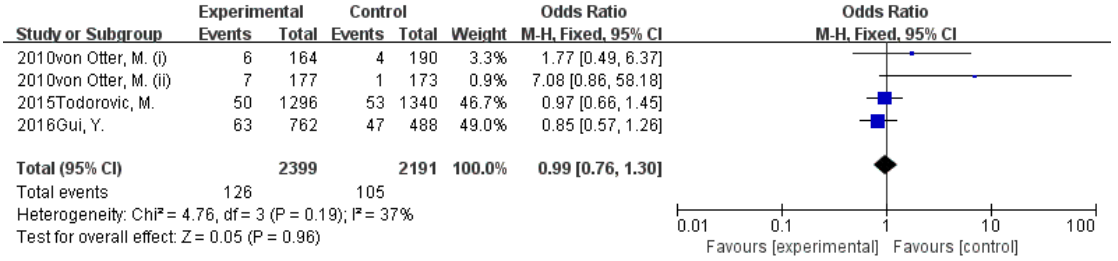

（43）rs10183914 (MAF-A)

Study or Subgroup	Experimental Events	Experimental Total	Control Events	Control Total	Weight	Odds Ratio M-H, Fixed, 95% CI
2010von Otter, M. (i)	109	330	139	380	8.4%	0.86 [0.63, 1.17]
2010von Otter, M. (ii)	113	372	129	374	8.7%	0.83 [0.61, 1.13]
2015Todorovic, M.	895	2580	979	2688	60.8%	0.93 [0.83, 1.04]
2016Gui, Y.	438	1528	262	978	22.1%	1.10 [0.92, 1.31]
**Total (95% CI)**		**4810**		**4420**	**100.0%**	**0.95 [0.87, 1.04]**
Total events	1555		1509			

Heterogeneity: Chi² = 3.88, df = 3 (P = 0.28); I² = 23%
Test for overall effect: Z = 1.14 (P = 0.25)

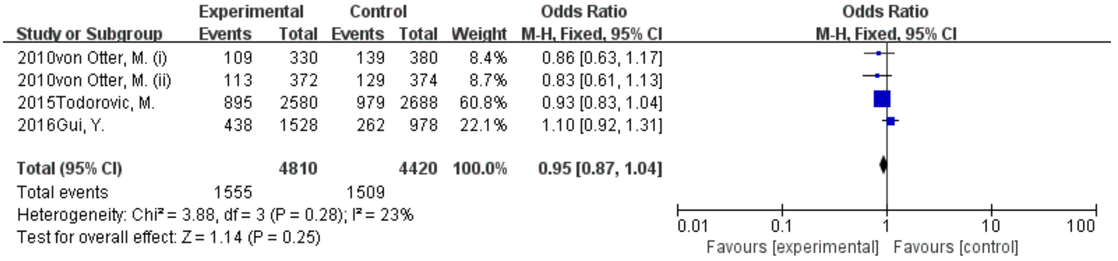

（44）rs10183914 (GA+AA)

Study or Subgroup	Experimental Events	Experimental Total	Control Events	Control Total	Weight	Odds Ratio M-H, Fixed, 95% CI
2010von Otter, M. (i)	95	165	114	190	7.9%	0.90 [0.59, 1.38]
2010von Otter, M. (ii)	93	186	101	187	8.9%	0.85 [0.57, 1.28]
2015Todorovic, M.	734	1290	797	1344	59.2%	0.91 [0.78, 1.06]
2016Gui, Y.	333	764	199	489	24.1%	1.13 [0.89, 1.42]
**Total (95% CI)**		**2405**		**2210**	**100.0%**	**0.95 [0.85, 1.07]**
Total events	1255		1211			

Heterogeneity: Chi² = 2.78, df = 3 (P = 0.43); I² = 0%
Test for overall effect: Z = 0.78 (P = 0.43)

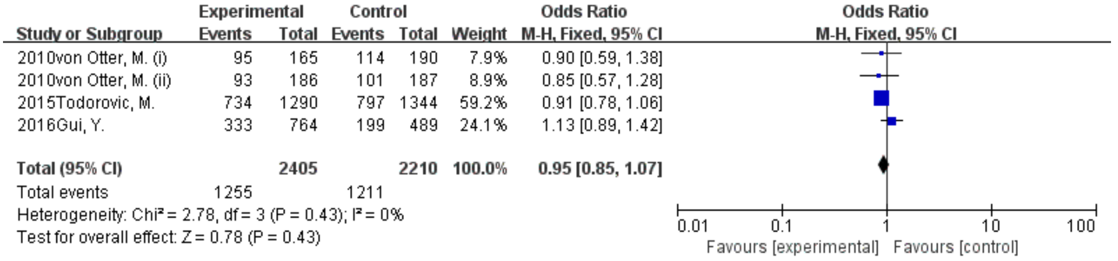

（45）rs10183914 (AA)

Study or Subgroup	Experimental Events	Experimental Total	Control Events	Control Total	Weight	Odds Ratio M-H, Fixed, 95% CI
2010von Otter, M. (i)	14	165	25	190	7.9%	0.61 [0.31, 1.22]
2010von Otter, M. (ii)	20	186	28	187	9.3%	0.68 [0.37, 1.26]
2015Todorovic, M.	161	1290	182	1344	58.1%	0.91 [0.73, 1.14]
2016Gui, Y.	105	764	63	489	24.7%	1.08 [0.77, 1.51]
**Total (95% CI)**		**2405**		**2210**	**100.0%**	**0.91 [0.76, 1.08]**
Total events	300		298			

Heterogeneity: Chi² = 3.07, df = 3 (P = 0.38); I² = 2%
Test for overall effect: Z = 1.10 (P = 0.27)

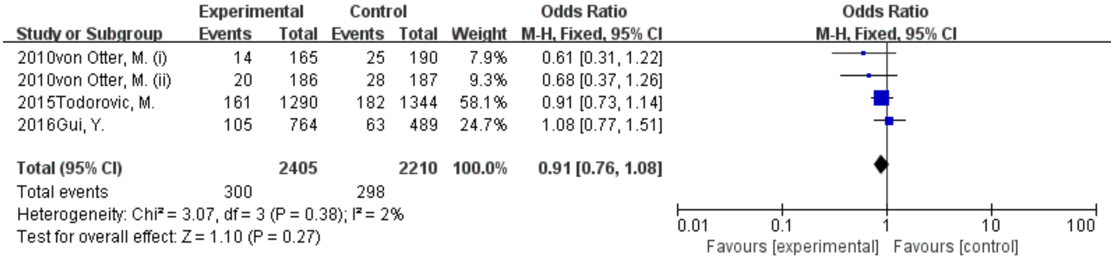

（46）rs1806649 (MAF-A)

Study or Subgroup	Experimental Events	Experimental Total	Control Events	Control Total	Weight	Odds Ratio M-H, Random, 95% CI
2010von Otter, M. (i)	93	330	110	380	18.9%	0.96 [0.69, 1.34]
2010von Otter, M. (ii)	78	372	107	362	18.3%	0.63 [0.45, 0.89]
2015Todorovic, M.	668	2598	693	2678	38.7%	0.99 [0.88, 1.12]
2016Gui, Y.	165	1524	101	978	24.1%	1.05 [0.81, 1.37]
**Total (95% CI)**		**4824**		**4398**	**100.0%**	**0.92 [0.77, 1.11]**
Total events	1004		1011			

Heterogeneity: Tau² = 0.02; Chi² = 6.67, df = 3 (P = 0.08); I² = 55%
Test for overall effect: Z = 0.87 (P = 0.39)

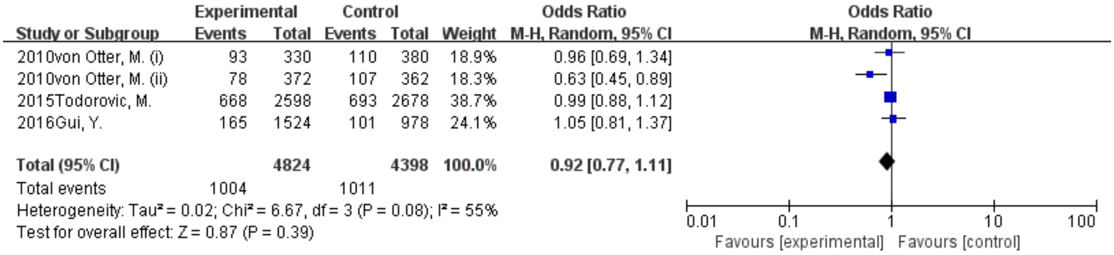

（47）rs1806649 (GA+AA)

Study or Subgroup	Experimental Events	Experimental Total	Control Events	Control Total	Weight	Odds Ratio M-H, Fixed, 95% CI
2010von Otter, M. (i)	79	165	95	190	8.9%	0.92 [0.61, 1.39]
2010von Otter, M. (ii)	69	186	84	181	10.3%	0.68 [0.45, 1.03]
2015Todorovic, M.	572	1299	601	1339	63.8%	0.97 [0.83, 1.13]
2016Gui, Y.	141	762	89	489	17.0%	1.02 [0.76, 1.37]
**Total (95% CI)**		**2412**		**2199**	**100.0%**	**0.94 [0.83, 1.07]**
Total events	861		869			

Heterogeneity: Chi² = 2.73, df = 3 (P = 0.44); I² = 0%
Test for overall effect: Z = 0.95 (P = 0.34)

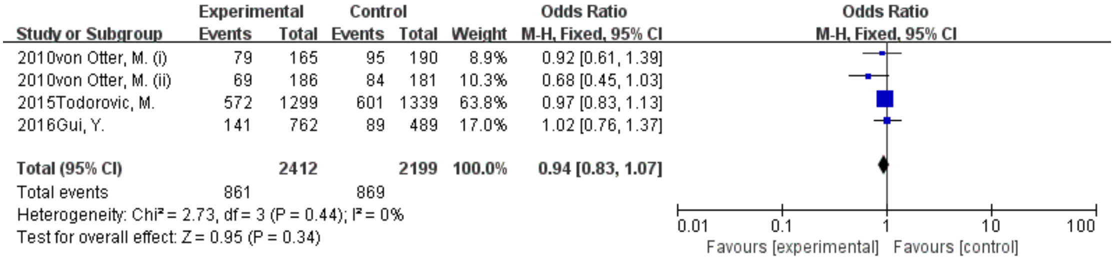

（48）rs1806649 (AA)

Study or Subgroup	Experimental Events	Experimental Total	Control Events	Control Total	Weight	Odds Ratio M-H, Random, 95% CI
2010von Otter, M. (i)	14	165	15	190	20.8%	1.08 [0.51, 2.31]
2010von Otter, M. (ii)	9	186	23	181	19.7%	0.35 [0.16, 0.78]
2015Todorovic, M.	96	1299	92	1339	37.1%	1.08 [0.80, 1.46]
2016Gui, Y.	24	762	12	489	22.5%	1.29 [0.64, 2.61]
**Total (95% CI)**		**2412**		**2199**	**100.0%**	**0.90 [0.56, 1.46]**
Total events	143		142			

Heterogeneity: Tau² = 0.14; Chi² = 7.47, df = 3 (P = 0.06); I² = 60%
Test for overall effect: Z = 0.42 (P = 0.67)

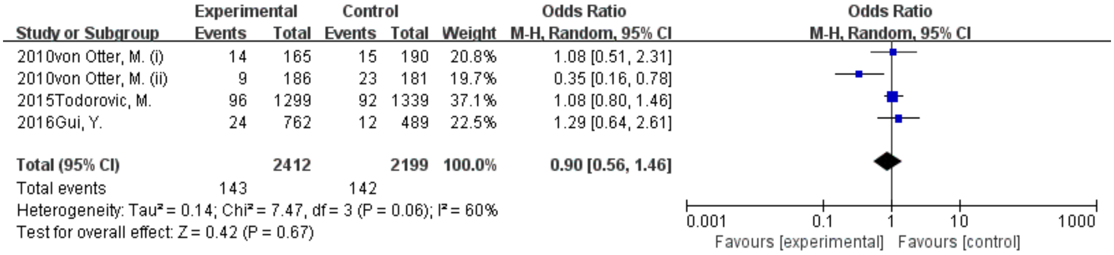

（49）rs2001350(MAF-G)

Study or Subgroup	Experimental Events	Experimental Total	Control Events	Control Total	Weight	Odds Ratio M-H, Fixed, 95% CI
2010von Otter, M. (i)	37	330	23	380	5.3%	1.96 [1.14, 3.37]
2010von Otter, M. (ii)	36	380	39	374	10.0%	0.90 [0.56, 1.45]
2015Todorovic, M.	232	2598	242	2696	60.7%	0.99 [0.82, 1.20]
2017Ran, C.	105	974	95	964	23.9%	1.11 [0.82, 1.48]
**Total (95% CI)**		**4282**		**4414**	**100.0%**	**1.06 [0.92, 1.23]**
Total events	410		399			

Heterogeneity: Chi² = 5.90, df = 3 (P = 0.12); I² = 49%
Test for overall effect: Z = 0.83 (P = 0.41)

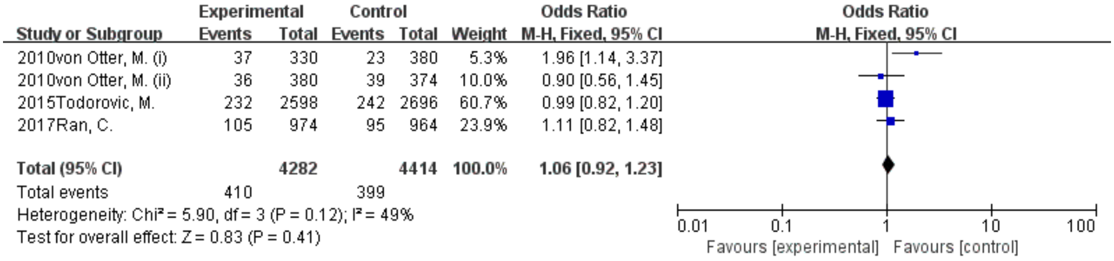

（50）rs2001350(AG+GG)

Study or Subgroup	Experimental Events	Experimental Total	Control Events	Control Total	Weight	Odds Ratio M-H, Random, 95% CI
2010von Otter, M. (i)	36	165	23	190	15.3%	2.03 [1.14, 3.59]
2010von Otter, M. (ii)	32	190	39	187	17.4%	0.77 [0.46, 1.29]
2015Todorovic, M.	222	1299	227	1348	38.1%	1.02 [0.83, 1.25]
2017Ran, C.	100	487	94	482	29.2%	1.07 [0.78, 1.46]
**Total (95% CI)**		**2141**		**2207**	**100.0%**	**1.09 [0.83, 1.43]**
Total events	390		383			

Heterogeneity: Tau² = 0.04; Chi² = 6.57, df = 3 (P = 0.09); I² = 54%
Test for overall effect: Z = 0.64 (P = 0.52)

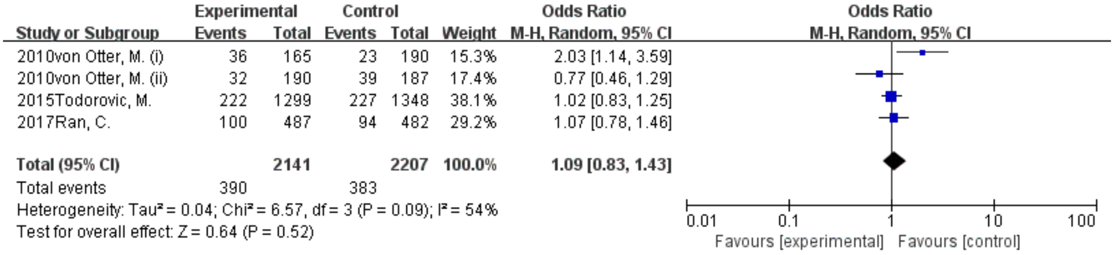

（51）rs2001350(GG)

Study or Subgroup	Experimental Events	Experimental Total	Control Events	Control Total	Weight	Odds Ratio M-H, Fixed, 95% CI
2010von Otter, M. (i)	1	165	0	190	2.8%	3.47 [0.14, 85.86]
2010von Otter, M. (ii)	4	190	0	187	3.0%	9.05 [0.48, 169.24]
2015Todorovic, M.	10	1299	15	1348	88.2%	0.69 [0.31, 1.54]
2017Ran, C.	5	487	1	482	6.0%	4.99 [0.58, 42.87]
**Total (95% CI)**		**2141**		**2207**	**100.0%**	**1.27 [0.67, 2.42]**
Total events	20		16			

Heterogeneity: Chi² = 5.89, df = 3 (P = 0.12); I² = 49%
Test for overall effect: Z = 0.74 (P = 0.46)

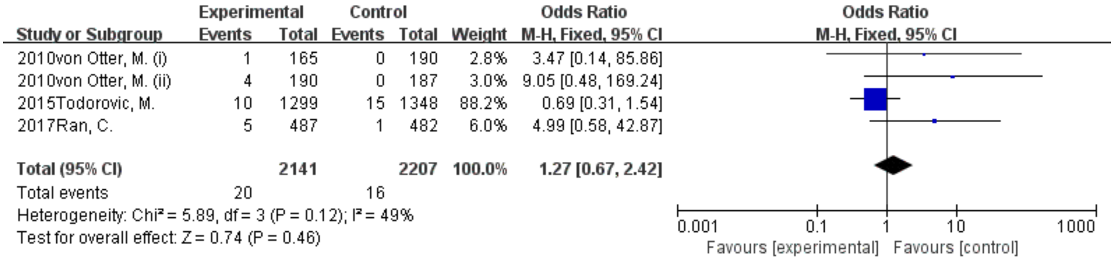

（52）rs3892097 (MAF-A)

Study or Subgroup	Experimental Events	Experimental Total	Control Events	Control Total	Weight	Odds Ratio M-H, Fixed, 95% CI
1993Kurth, M. C.	33	100	44	220	3.9%	1.97 [1.16, 3.35]
1994Plante-Bordeneuve, V.	24	96	32	176	3.6%	1.50 [0.82, 2.73]
1995Chen, X.	2	56	30	424	1.4%	0.49 [0.11, 2.09]
1996Bordet, R.	35	210	41	210	7.2%	0.82 [0.50, 1.36]
1996Diederich, N.	31	160	42	212	6.1%	0.97 [0.58, 1.63]
1996Gasser, T.	48	230	29	146	5.9%	1.06 [0.64, 1.78]
1996Lucotte, G.	23	94	10	94	1.6%	2.72 [1.21, 6.10]
1999Atkinson, A.	18	66	20	150	1.9%	2.44 [1.19, 5.00]
1999Nicholl, D. J.	88	412	80	410	13.3%	1.12 [0.80, 1.57]
1999Payami, H.	231	1152	103	494	24.3%	0.95 [0.73, 1.24]
2000Woo, S. I.	4	186	1	244	0.2%	5.34 [0.59, 48.19]
2004Elbaz, A.	78	380	155	838	16.2%	1.14 [0.84, 1.54]
2007Halling, J.	57	158	119	306	10.9%	0.89 [0.60, 1.32]
2010Singh, M.	24	154	25	250	3.4%	1.66 [0.91, 3.03]
**Total (95% CI)**		**3454**		**4174**	**100.0%**	**1.14 [1.00, 1.29]**
Total events	696		731			

Heterogeneity: Chi² = 23.79, df = 13 (P = 0.03); I² = 45%
Test for overall effect: Z = 2.04 (P = 0.04)

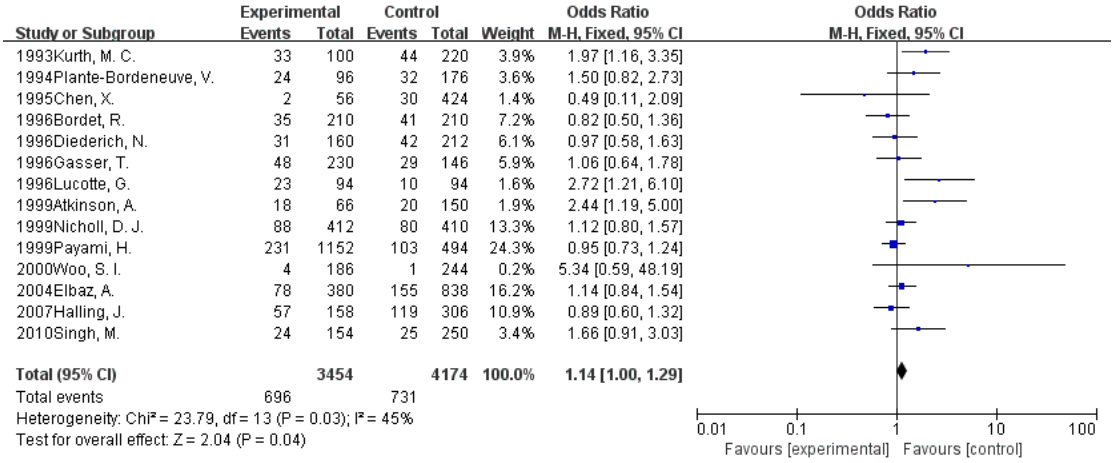

（53）rs3892097(GA+AA)

Study or Subgroup	Experimental Events	Experimental Total	Control Events	Control Total	Weight	Odds Ratio M-H, Random, 95% CI
1993Kurth, M. C.	28	50	38	110	6.9%	2.41 [1.22, 4.77]
1994Plante-Bordeneuve, V.	22	48	26	88	6.4%	2.02 [0.97, 4.18]
1995Chen, X.	1	28	27	212	1.2%	0.25 [0.03, 1.94]
1996Bordet, R.	33	105	39	105	8.3%	0.78 [0.44, 1.37]
1996Diederich, N.	28	80	35	106	7.8%	1.09 [0.59, 2.01]
1996Gasser, T.	41	115	26	73	7.8%	1.00 [0.54, 1.85]
1996Lucotte, G.	18	47	9	47	4.6%	2.62 [1.03, 6.67]
1999Atkinson, A.	16	33	17	75	5.1%	3.21 [1.34, 7.67]
1999Nicholl, D. J.	76	206	68	205	11.0%	1.18 [0.79, 1.77]
1999Payami, H.	202	576	87	247	12.6%	0.99 [0.73, 1.36]
2000Woo, S. I.	4	93	1	122	1.1%	5.44 [0.60, 49.49]
2004Elbaz, A.	68	190	139	419	11.8%	1.12 [0.78, 1.61]
2007Halling, J.	44	79	95	153	8.6%	0.77 [0.44, 1.33]
2010Singh, M.	23	77	22	125	7.0%	1.99 [1.02, 3.90]
**Total (95% CI)**		**1727**		**2087**	**100.0%**	**1.29 [1.02, 1.63]**
Total events	604		629			

Heterogeneity: Tau² = 0.09; Chi² = 26.52, df = 13 (P = 0.01); I² = 51%
Test for overall effect: Z = 2.09 (P = 0.04)

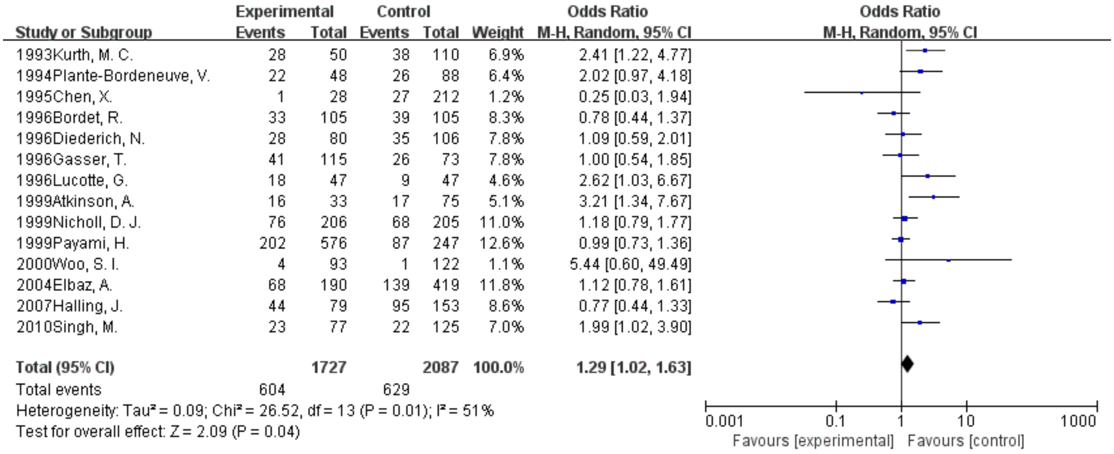

（54）rs3892097(AA)

Study or Subgroup	Experimental Events	Experimental Total	Control Events	Control Total	Weight	Odds Ratio M-H, Fixed, 95% CI
1993Kurth, M. C.	5	50	6	110	4.2%	1.93 [0.56, 6.64]
1994Plante-Bordeneuve, V.	2	48	6	88	5.1%	0.59 [0.12, 3.06]
1995Chen, X.	1	28	3	212	0.8%	2.58 [0.26, 25.69]
1996Bordet, R.	2	105	2	105	2.5%	1.00 [0.14, 7.23]
1996Diederich, N.	3	80	7	106	7.3%	0.55 [0.14, 2.20]
1996Gasser, T.	7	115	3	73	4.3%	1.51 [0.38, 6.04]
1996Lucotte, G.	5	47	1	47	1.1%	5.48 [0.61, 48.80]
1999Atkinson, A.	2	33	3	75	2.2%	1.55 [0.25, 9.73]
1999Nicholl, D. J.	12	206	12	205	14.2%	0.99 [0.44, 2.27]
1999Payami, H.	29	576	16	247	26.6%	0.77 [0.41, 1.44]
2000Woo, S. I.	0	93	0	122		Not estimable
2004Elbaz, A.	10	190	16	419	11.8%	1.40 [0.62, 3.14]
2007Halling, J.	13	79	24	153	17.1%	1.06 [0.51, 2.21]
2010Singh, M.	1	77	3	125	2.8%	0.54 [0.05, 5.24]
**Total (95% CI)**		**1727**		**2087**	**100.0%**	**1.06 [0.78, 1.44]**
Total events	92		102			

Heterogeneity: Chi² = 7.24, df = 12 (P = 0.84); I² = 0%
Test for overall effect: Z = 0.40 (P = 0.69)

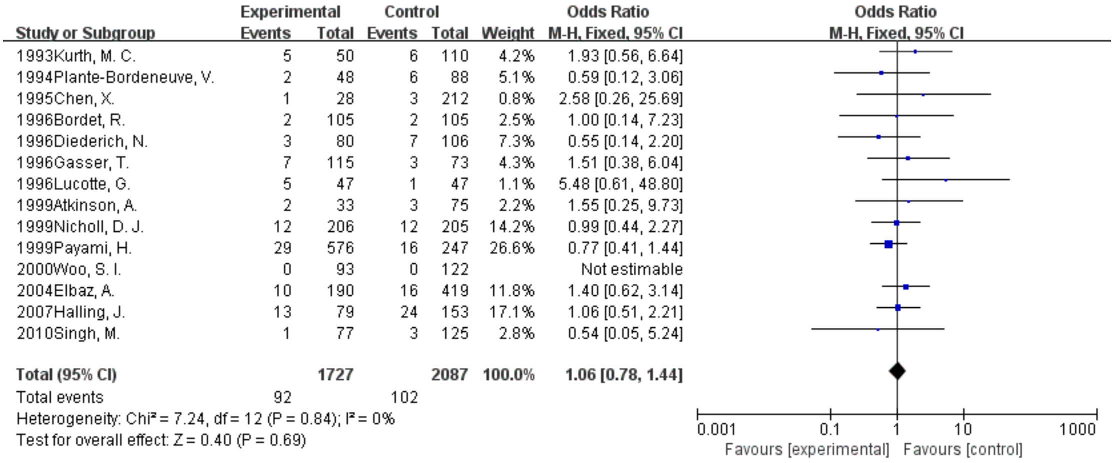

（55）A2637(MAF- -)

Study or Subgroup	Experimental Events	Experimental Total	Control Events	Control Total	Weight	Odds Ratio M-H, Fixed, 95% CI
1994Plante-Bordeneuve, V.	3	96	2	176	8.1%	2.81 [0.46, 17.09]
1996Bordet, R.	5	210	7	210	40.4%	0.71 [0.22, 2.27]
1996Lucotte, G.	1	94	0	94	2.9%	3.03 [0.12, 75.38]
1999Nicholl, D. J.	7	412	8	410	46.6%	0.87 [0.31, 2.42]
2007Halling, J.	1	158	0	306	2.0%	5.84 [0.24, 144.14]
**Total (95% CI)**		**970**		**1196**	**100.0%**	**1.12 [0.58, 2.16]**
Total events	17		17			

Heterogeneity: Chi² = 3.22, df = 4 (P = 0.52); I² = 0%
Test for overall effect: Z = 0.35 (P = 0.73)

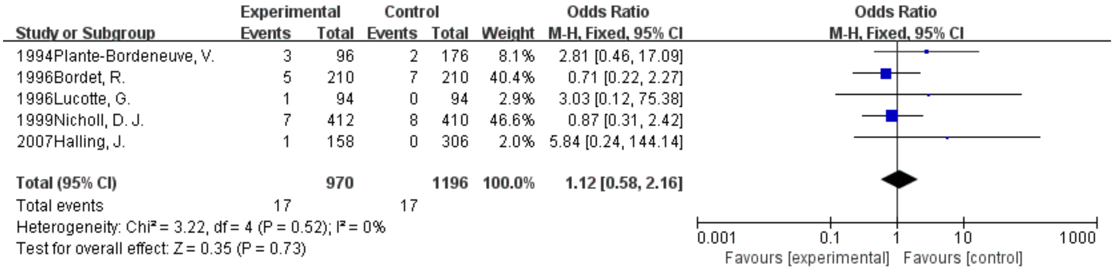

（56）A2637(A-+ --)

Study or Subgroup	Experimental Events	Experimental Total	Control Events	Control Total	Weight	Odds Ratio M-H, Fixed, 95% CI
1994Plante-Bordeneuve, V.	3	48	2	88	8.0%	2.87 [0.46, 17.78]
1996Bordet, R.	5	105	7	105	40.3%	0.70 [0.21, 2.28]
1996Lucotte, G.	1	47	0	47	2.9%	3.06 [0.12, 77.16]
1999Nicholl, D. J.	7	206	8	205	46.8%	0.87 [0.31, 2.43]
2007Halling, J.	1	79	0	153	2.0%	5.87 [0.24, 145.67]
**Total (95% CI)**		**485**		**598**	**100.0%**	**1.12 [0.58, 2.18]**
Total events	17		17			

Heterogeneity: Chi² = 3.26, df = 4 (P = 0.52); I² = 0%
Test for overall effect: Z = 0.35 (P = 0.73)

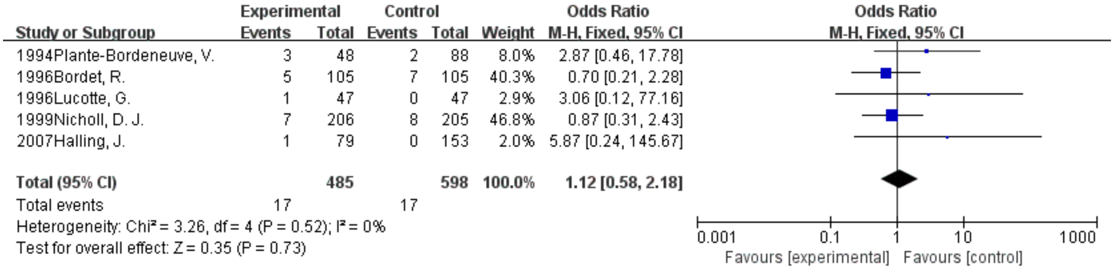

（57）rs2031920 (MAF-T)

Study or Subgroup	Experimental Events	Experimental Total	Control Events	Control Total	Weight	Odds Ratio M-H, Fixed, 95% CI
2000Wang, J. (2)	54	300	44	300	29.3%	1.28 [0.83, 1.97]
2002Wu, R. M.	110	468	117	502	70.1%	1.01 [0.75, 1.36]
2008Singh, M.	5	140	1	200	0.6%	7.37 [0.85, 63.79]
**Total (95% CI)**		**908**		**1002**	**100.0%**	**1.13 [0.89, 1.44]**
Total events	169		162			

Heterogeneity: Chi² = 3.74, df = 2 (P = 0.15); I² = 47%
Test for overall effect: Z = 0.99 (P = 0.32)

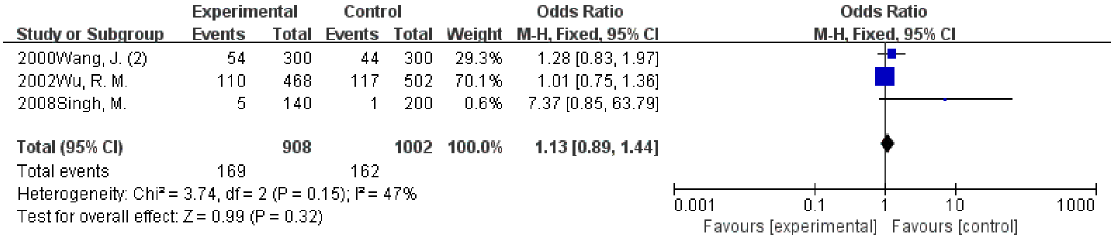

（58）rs2031920 (CT+TT)

Study or Subgroup	Experimental Events	Experimental Total	Control Events	Control Total	Weight	Odds Ratio M-H, Fixed, 95% CI
2000Wang, J. (2)	49	150	41	150	31.4%	1.29 [0.79, 2.12]
2002Wu, R. M.	99	234	107	251	67.7%	0.99 [0.69, 1.42]
2008Singh, M.	5	70	1	100	0.9%	7.62 [0.87, 66.68]
**Total (95% CI)**		**454**		**501**	**100.0%**	**1.14 [0.86, 1.52]**
Total events	153		149			

Heterogeneity: Chi² = 3.80, df = 2 (P = 0.15); I² = 47%
Test for overall effect: Z = 0.90 (P = 0.37)

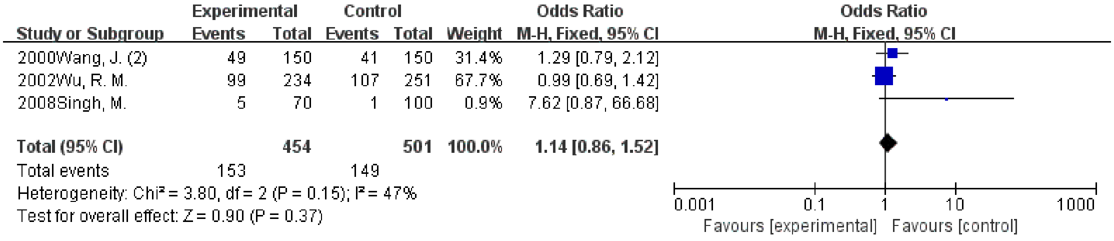

（59）rs2031920 (TT)

Study or Subgroup	Experimental Events	Experimental Total	Control Events	Control Total	Weight	Odds Ratio M-H, Fixed, 95% CI
2000Wang, J. (2)	5	150	3	150	24.0%	1.69 [0.40, 7.20]
2002Wu, R. M.	11	234	10	251	76.0%	1.19 [0.50, 2.85]
2008Singh, M.	0	70	0	100		Not estimable
**Total (95% CI)**		**454**		**501**	**100.0%**	**1.31 [0.62, 2.76]**
Total events	16		13			

Heterogeneity: Chi² = 0.17, df = 1 (P = 0.68); I² = 0%
Test for overall effect: Z = 0.71 (P = 0.48)

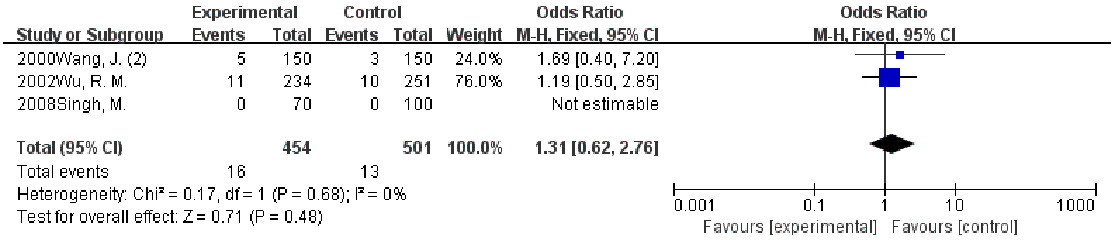

（60）rs705379, C-108T(MAF-T)

Study or Subgroup	Experimental Events	Experimental Total	Control Events	Control Total	Weight	Odds Ratio M-H, Fixed, 95% CI
2003Kelada, S. N.	119	272	212	456	17.3%	0.90 [0.66, 1.21]
2012Belin, A. C.	499	1006	551	1086	51.9%	0.96 [0.80, 1.13]
2013Lee, P. C.	263	538	379	774	30.8%	1.00 [0.80, 1.24]
**Total (95% CI)**		**1816**		**2316**	**100.0%**	**0.96 [0.85, 1.08]**
Total events	881		1142			

Heterogeneity: Chi² = 0.32, df = 2 (P = 0.85); I² = 0%
Test for overall effect: Z = 0.68 (P = 0.49)

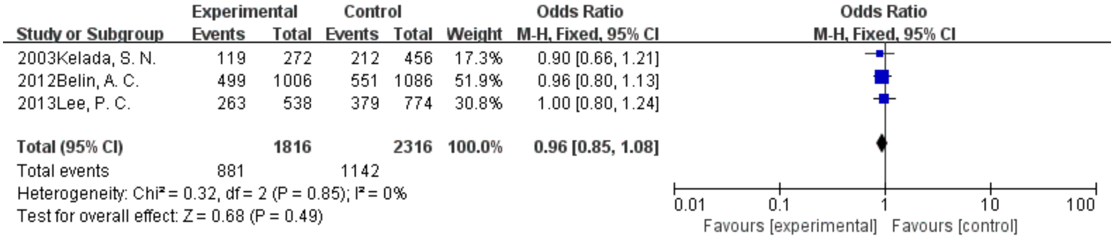

（61）rs705379, C-108T(CT+TT)

Study or Subgroup	Experimental Events	Experimental Total	Control Events	Control Total	Weight	Odds Ratio M-H, Fixed, 95% CI
2003Kelada, S. N.	93	136	157	228	18.2%	0.98 [0.62, 1.55]
2012Belin, A. C.	373	503	402	543	49.1%	1.01 [0.76, 1.33]
2013Lee, P. C.	192	269	283	387	32.7%	0.92 [0.65, 1.30]
**Total (95% CI)**		**908**		**1158**	**100.0%**	**0.97 [0.80, 1.18]**
Total events	658		842			

Heterogeneity: Chi² = 0.17, df = 2 (P = 0.92); I² = 0%
Test for overall effect: Z = 0.29 (P = 0.77)

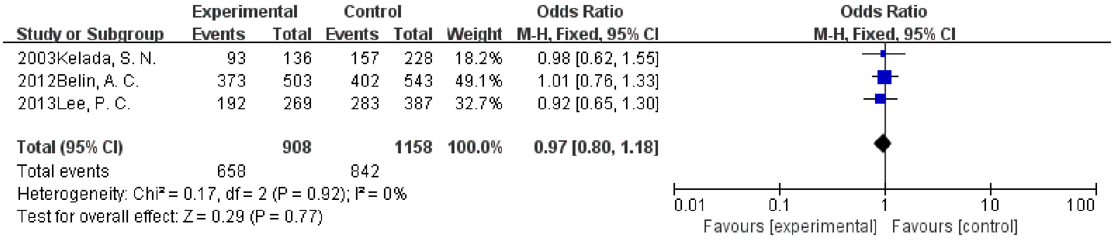

（62）rs705379, C-108T(TT)

Study or Subgroup	Experimental Events	Experimental Total	Control Events	Control Total	Weight	Odds Ratio M-H, Fixed, 95% CI
2003Kelada, S. N.	26	136	55	228	16.7%	0.74 [0.44, 1.26]
2012Belin, A. C.	126	503	149	543	54.1%	0.88 [0.67, 1.16]
2013Lee, P. C.	71	269	96	387	29.2%	1.09 [0.76, 1.55]
**Total (95% CI)**		**908**		**1158**	**100.0%**	**0.92 [0.75, 1.12]**
Total events	223		300			

Heterogeneity: Chi² = 1.56, df = 2 (P = 0.46); I² = 0%
Test for overall effect: Z = 0.82 (P = 0.41)

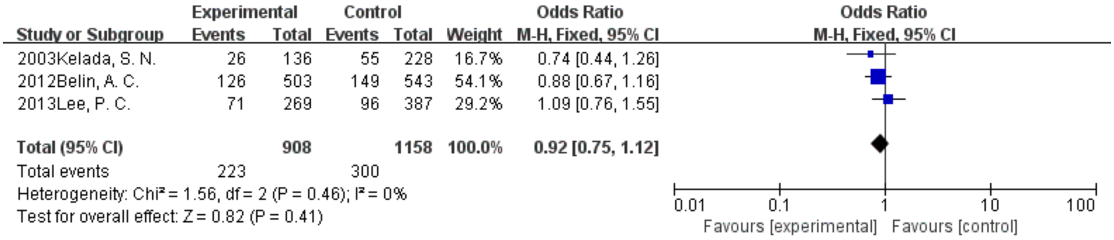

（63）rs854560(MAF-A)

Study or Subgroup	Experimental Events	Experimental Total	Control Events	Control Total	Weight	Odds Ratio M-H, Fixed, 95% CI
2000Wang, J. (1)	16	360	15	360	1.4%	1.07 [0.52, 2.20]
2001Akhmedova, S. N.	101	234	130	414	5.1%	1.66 [1.19, 2.31]
2002Carmine, A.	86	200	63	200	3.4%	1.64 [1.09, 2.47]
2003Kelada, S. N.	110	300	172	486	7.9%	1.06 [0.78, 1.43]
2004Clarimon, J.	92	276	85	260	5.6%	1.03 [0.72, 1.48]
2005Fong, C. S.	6	250	12	324	1.0%	0.64 [0.24, 1.73]
2010Manthripragada, A. D.(i)	206	564	186	580	11.1%	1.22 [0.95, 1.56]
2010Manthripragada, A. D.(ii)	240	702	218	726	13.4%	1.21 [0.97, 1.51]
2011Punia, S.	196	974	173	948	13.3%	1.13 [0.90, 1.42]
2012Belin, A. C.	394	988	385	1024	21.6%	1.10 [0.92, 1.32]
2013Lee, P. C.	209	574	294	880	14.1%	1.14 [0.92, 1.42]
2019Mota, A.	71	140	47	150	2.1%	2.26 [1.40, 3.64]
**Total (95% CI)**		**5562**		**6352**	**100.0%**	**1.20 [1.10, 1.30]**
Total events	1727		1780			

Heterogeneity: Chi² = 17.00, df = 11 (P = 0.11); I² = 35%
Test for overall effect: Z = 4.32 (P < 0.0001)

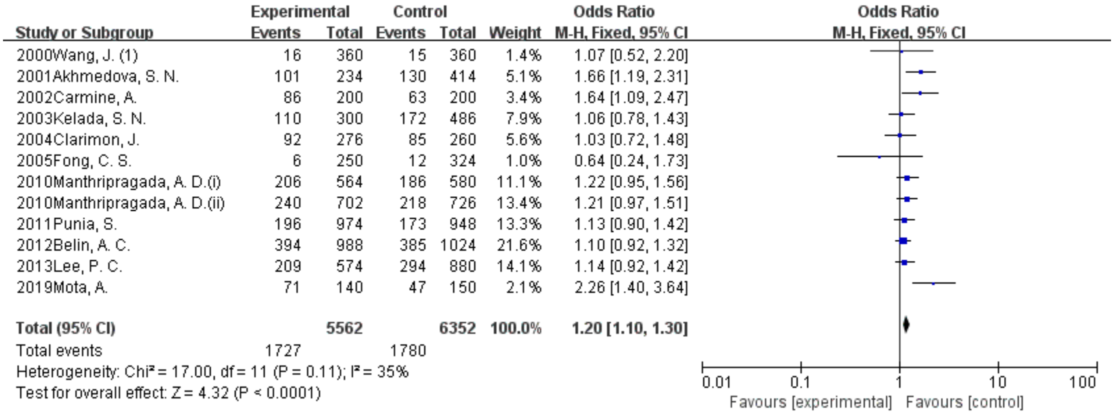

（64）rs854560(TA+AA)

Study or Subgroup	Experimental Events	Experimental Total	Control Events	Control Total	Weight	Odds Ratio M-H, Fixed, 95% CI
2000Wang, J. (1)	15	180	14	180	2.2%	1.08 [0.50, 2.30]
2001Akhmedova, S. N.	81	117	105	207	4.0%	2.19 [1.36, 3.52]
2002Carmine, A.	69	100	51	100	2.7%	2.14 [1.20, 3.81]
2003Kelada, S. N.	90	150	138	243	7.2%	1.14 [0.75, 1.73]
2004Clarimon, J.	80	138	72	130	5.3%	1.11 [0.69, 1.80]
2005Fong, C. S.	5	125	11	162	1.6%	0.57 [0.19, 1.69]
2010Manthripragada, A. D.(i)	164	282	157	290	11.0%	1.18 [0.85, 1.64]
2010Manthripragada, A. D.(ii)	192	351	183	363	13.9%	1.19 [0.89, 1.59]
2011Punia, S.	170	487	155	474	17.4%	1.10 [0.84, 1.44]
2012Belin, A. C.	316	494	313	512	18.9%	1.13 [0.87, 1.46]
2013Lee, P. C.	167	287	245	440	13.8%	1.11 [0.82, 1.50]
2019Mota, A.	46	70	39	75	2.2%	1.77 [0.91, 3.46]
**Total (95% CI)**		**2781**		**3176**	**100.0%**	**1.21 [1.08, 1.35]**
Total events	1395		1483			

Heterogeneity: Chi² = 14.09, df = 11 (P = 0.23); I² = 22%
Test for overall effect: Z = 3.40 (P = 0.0007)

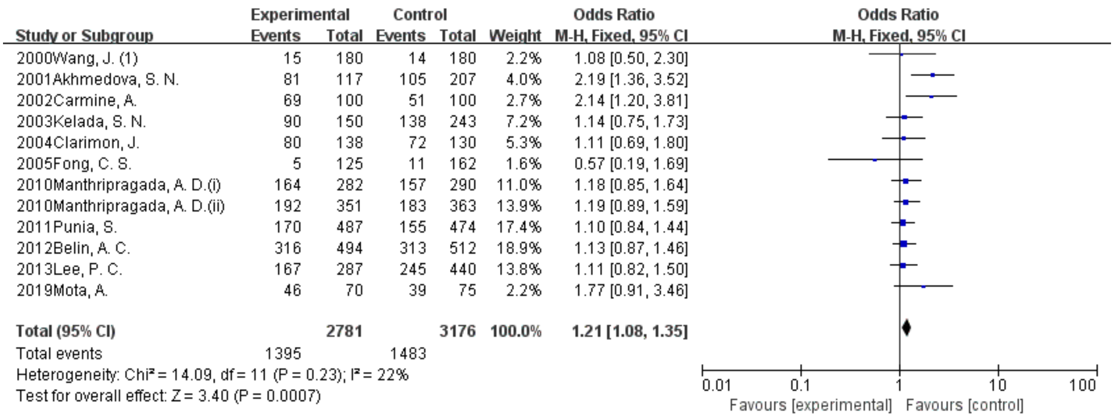

（65）rs854560(AA)

Study or Subgroup	Experimental Events	Experimental Total	Control Events	Control Total	Weight	Odds Ratio M-H, Fixed, 95% CI
2000Wang, J. (1)	1	180	1	180	0.4%	1.00 [0.06, 16.11]
2001Akhmedova, S. N.	20	117	25	207	6.5%	1.50 [0.79, 2.84]
2002Carmine, A.	17	100	12	100	4.3%	1.50 [0.68, 3.33]
2003Kelada, S. N.	20	150	34	243	9.8%	0.95 [0.52, 1.71]
2004Clarimon, J.	12	138	13	130	5.3%	0.86 [0.38, 1.95]
2005Fong, C. S.	1	125	1	162	0.4%	1.30 [0.08, 20.96]
2010Manthripragada, A. D.(i)	42	282	29	290	10.6%	1.57 [0.95, 2.61]
2010Manthripragada, A. D.(ii)	48	351	35	363	12.9%	1.48 [0.93, 2.36]
2011Punia, S.	26	487	18	474	7.5%	1.43 [0.77, 2.64]
2012Belin, A. C.	78	494	72	512	25.9%	1.15 [0.81, 1.62]
2013Lee, P. C.	42	287	49	440	14.3%	1.37 [0.88, 2.13]
2019Mota, A.	25	70	8	75	2.2%	4.65 [1.93, 11.23]
**Total (95% CI)**		**2781**		**3176**	**100.0%**	**1.37 [1.15, 1.62]**
Total events	332		297			

Heterogeneity: Chi² = 11.75, df = 11 (P = 0.38); I² = 6%
Test for overall effect: Z = 3.63 (P = 0.0003)

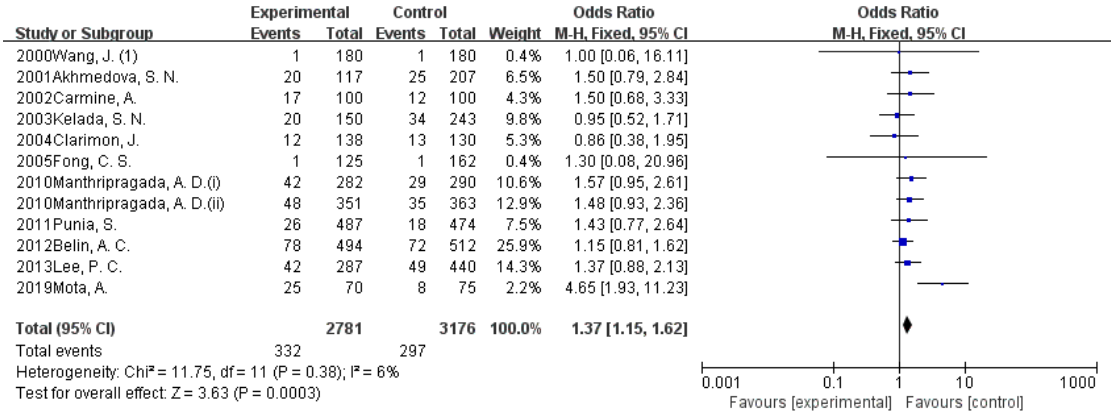

（66）rs662, Q192R(MAF-G)

Study or Subgroup	Experimental Events	Experimental Total	Control Events	Control Total	Weight	Odds Ratio M-H, Fixed, 95% CI
1998Kondo, I.	242	338	312	504	7.1%	1.55 [1.15, 2.09]
1999Akhmedova, S.	61	242	60	234	4.6%	0.98 [0.65, 1.48]
2000Taylor, M. C.	55	184	72	244	4.4%	1.02 [0.67, 1.55]
2000Wang, J. (1)	225	360	220	360	8.3%	1.06 [0.79, 1.43]
2003Kelada, S. N.	81	300	145	484	8.1%	0.86 [0.63, 1.19]
2004Clarimon, J.	95	288	85	270	5.9%	1.07 [0.75, 1.53]
2011Punia, S.	372	974	371	948	23.3%	0.96 [0.80, 1.15]
2012Belin, A. C.	261	1022	263	1088	19.0%	1.08 [0.88, 1.31]
2013Lee, P. C.	164	562	271	794	16.0%	0.80 [0.63, 1.00]
2019Mota, A.	42	140	48	150	3.3%	0.91 [0.55, 1.50]
**Total (95% CI)**		**4410**		**5076**	**100.0%**	**1.01 [0.92, 1.10]**
Total events	1598		1847			

Heterogeneity: Chi² = 14.01, df = 9 (P = 0.12); I² = 36%
Test for overall effect: Z = 0.16 (P = 0.87)

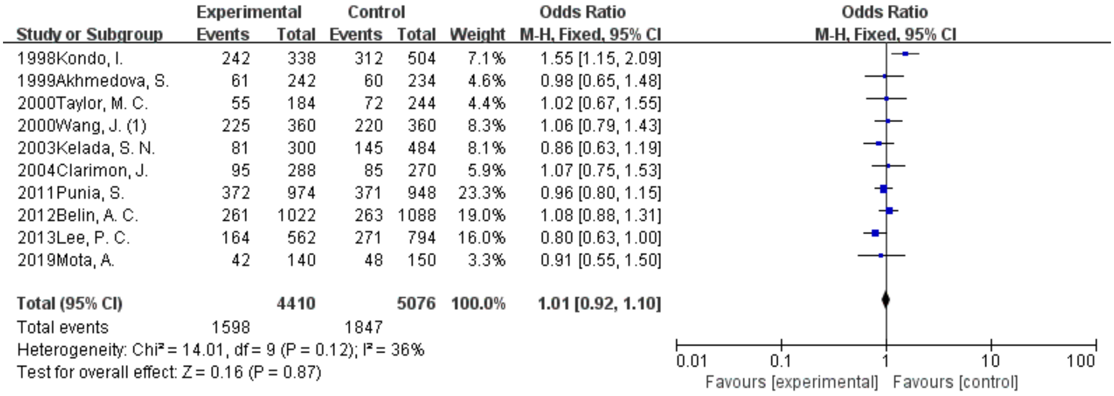

（67）rs662, Q192R(AG+GG)

Study or Subgroup	Experimental Events	Experimental Total	Control Events	Control Total	Weight	Odds Ratio M-H, Fixed, 95% CI
1998Kondo, I.	156	169	218	252	2.6%	1.87 [0.96, 3.66]
1999Akhmedova, S.	56	121	54	117	5.6%	1.01 [0.60, 1.67]
2000Taylor, M. C.	50	92	59	122	4.4%	1.27 [0.74, 2.19]
2000Wang, J. (1)	155	180	154	180	4.1%	1.05 [0.58, 1.89]
2003Kelada, S. N.	69	150	121	242	9.5%	0.85 [0.57, 1.28]
2004Clarimon, J.	77	144	70	135	6.4%	1.07 [0.67, 1.71]
2011Punia, S.	299	487	298	474	22.2%	0.94 [0.72, 1.22]
2012Belin, A. C.	229	511	236	544	24.1%	1.06 [0.83, 1.35]
2013Lee, P. C.	137	281	217	397	17.6%	0.79 [0.58, 1.07]
2019Mota, A.	37	70	40	75	3.5%	0.98 [0.51, 1.88]
**Total (95% CI)**		**2205**		**2538**	**100.0%**	**0.99 [0.88, 1.12]**
Total events	1265		1467			

Heterogeneity: Chi² = 7.49, df = 9 (P = 0.59); I² = 0%
Test for overall effect: Z = 0.16 (P = 0.87)

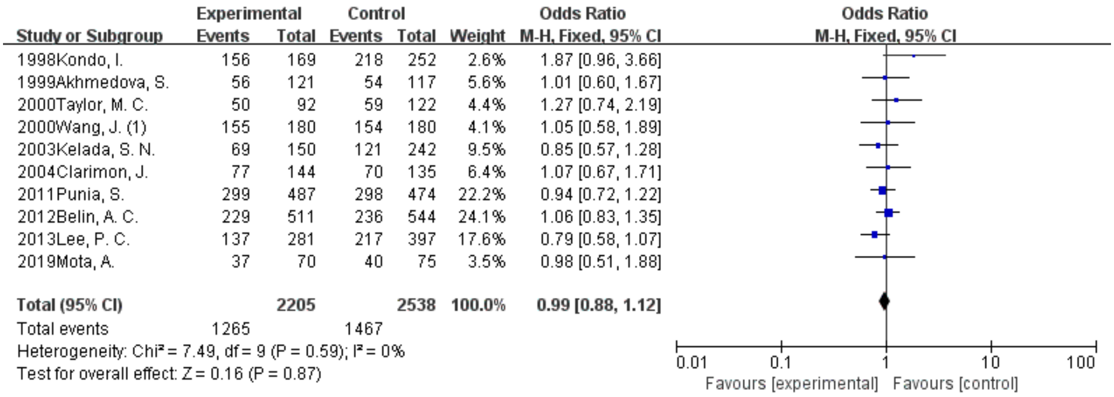

（68）rs662, Q192R(GG)

Study or Subgroup	Experimental Events	Experimental Total	Control Events	Control Total	Weight	Odds Ratio M-H, Fixed, 95% CI
1998Kondo, I.	86	169	94	252	14.3%	1.74 [1.17, 2.59]
1999Akhmedova, S.	5	121	6	117	2.3%	0.80 [0.24, 2.69]
2000Taylor, M. C.	5	92	13	122	4.1%	0.48 [0.17, 1.40]
2000Wang, J. (1)	70	180	66	180	15.6%	1.10 [0.72, 1.68]
2003Kelada, S. N.	12	150	24	242	6.5%	0.79 [0.38, 1.63]
2004Clarimon, J.	18	144	15	135	5.2%	1.14 [0.55, 2.37]
2011Punia, S.	73	487	73	474	24.3%	0.97 [0.68, 1.38]
2012Belin, A. C.	32	511	27	544	9.5%	1.28 [0.76, 2.17]
2013Lee, P. C.	27	281	54	397	15.6%	0.68 [0.41, 1.10]
2019Mota, A.	5	70	8	75	2.8%	0.64 [0.20, 2.07]
**Total (95% CI)**		**2205**		**2538**	**100.0%**	**1.05 [0.88, 1.24]**
Total events	333		380			

Heterogeneity: Chi² = 13.76, df = 9 (P = 0.13); I² = 35%
Test for overall effect: Z = 0.54 (P = 0.59)

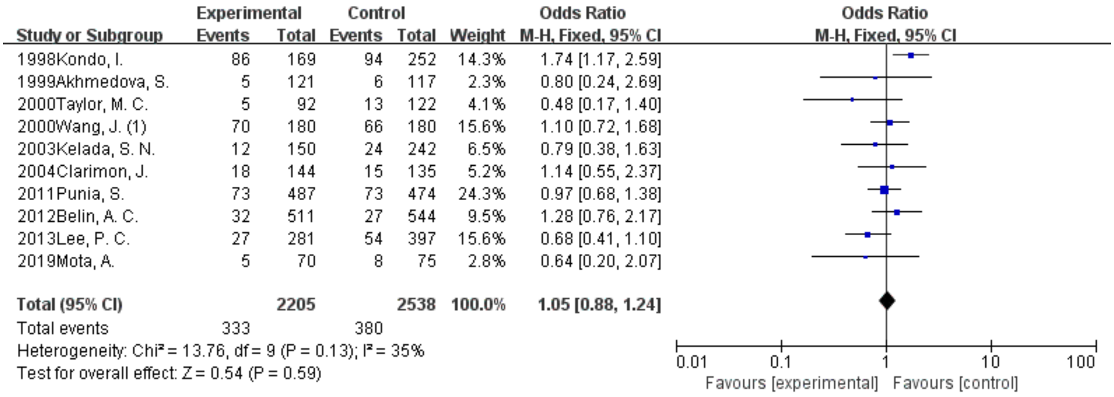

（69）rs1799929(MAF-T)

Study or Subgroup	Experimental Events	Experimental Total	Control Events	Control Total	Weight	Odds Ratio M-H, Fixed, 95% CI
1999Nicholl, D. J.	185	410	185	410	45.3%	1.00 [0.76, 1.32]
2006Borlak, J.	105	248	228	486	39.7%	0.83 [0.61, 1.13]
2010Singh, M.	43	154	61	250	15.0%	1.20 [0.76, 1.89]
**Total (95% CI)**		**812**		**1146**	**100.0%**	**0.96 [0.80, 1.16]**
Total events	333		474			

Heterogeneity: Chi² = 1.85, df = 2 (P = 0.40); I² = 0%
Test for overall effect: Z = 0.40 (P = 0.69)

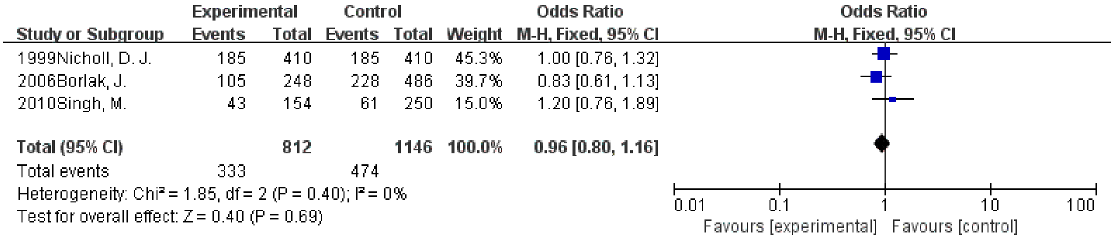

（70）rs1799929(CT+TT)

Study or Subgroup	Experimental Events	Experimental Total	Control Events	Control Total	Weight	Odds Ratio M-H, Fixed, 95% CI
1999Nicholl, D. J.	146	205	138	205	39.0%	1.20 [0.79, 1.83]
2006Borlak, J.	80	124	174	243	41.0%	0.72 [0.45, 1.14]
2010Singh, M.	39	77	54	125	20.0%	1.35 [0.76, 2.39]
**Total (95% CI)**		**406**		**573**	**100.0%**	**1.03 [0.79, 1.36]**
Total events	265		366			

Heterogeneity: Chi² = 3.67, df = 2 (P = 0.16); I² = 46%
Test for overall effect: Z = 0.24 (P = 0.81)

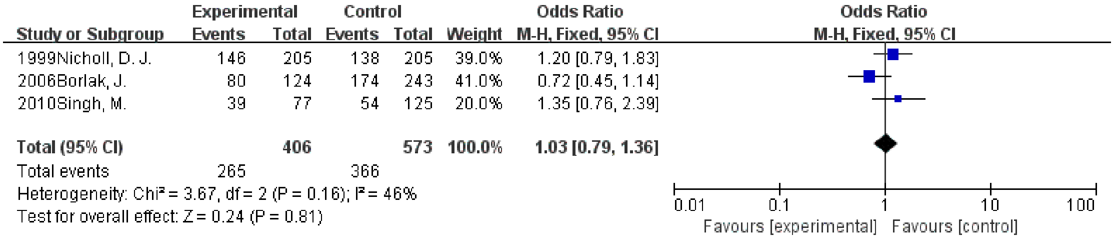

（71）rs1799929(TT)

Study or Subgroup	Experimental Events	Experimental Total	Control Events	Control Total	Weight	Odds Ratio M-H, Fixed, 95% CI
1999Nicholl, D. J.	39	205	47	205	52.7%	0.79 [0.49, 1.27]
2006Borlak, J.	25	124	54	243	40.3%	0.88 [0.52, 1.51]
2010Singh, M.	4	77	7	125	7.0%	0.92 [0.26, 3.27]
**Total (95% CI)**		**406**		**573**	**100.0%**	**0.84 [0.59, 1.18]**
Total events	68		108			

Heterogeneity: Chi² = 0.12, df = 2 (P = 0.94); I² = 0%
Test for overall effect: Z = 1.02 (P = 0.31)

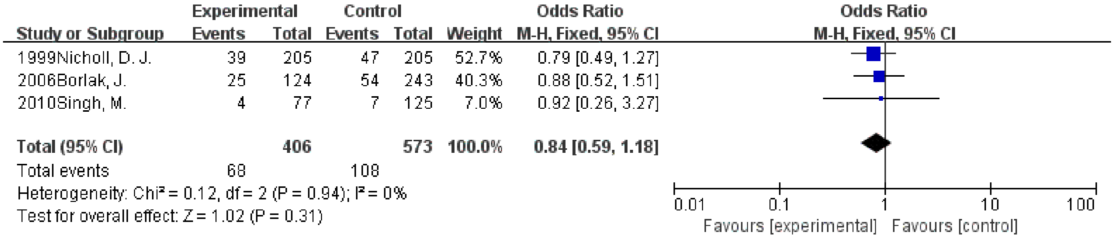

（72）rs1799929(MAF-A)

Study or Subgroup	Experimental Events	Experimental Total	Control Events	Control Total	Weight	Odds Ratio M-H, Fixed, 95% CI
1999Nicholl, D. J.	110	408	113	410	61.5%	0.97 [0.71, 1.32]
2006Borlak, J.	10	248	18	486	8.7%	1.09 [0.50, 2.40]
2010Singh, M.	52	154	79	250	29.8%	1.10 [0.72, 1.69]
**Total (95% CI)**		**810**		**1146**	**100.0%**	**1.02 [0.80, 1.30]**
Total events	172		210			

Heterogeneity: Chi² = 0.26, df = 2 (P = 0.88); I² = 0%
Test for overall effect: Z = 0.17 (P = 0.87)

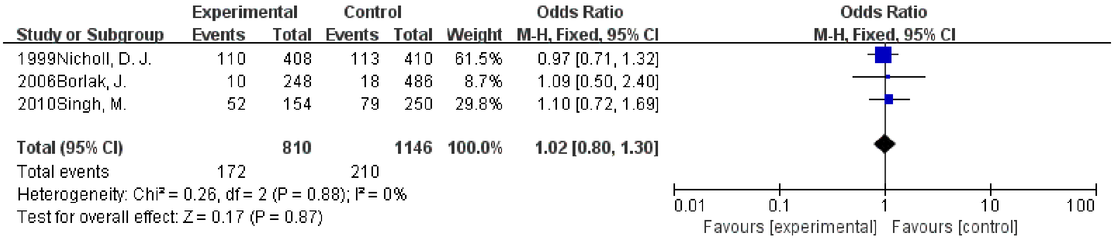

（73）rs1799929(GA+AA)

Study or Subgroup	Experimental Events	Experimental Total	Control Events	Control Total	Weight	Odds Ratio M-H, Fixed, 95% CI
1999Nicholl, D. J.	93	204	95	205	60.9%	0.97 [0.66, 1.43]
2006Borlak, J.	9	124	18	243	13.3%	0.98 [0.43, 2.25]
2010Singh, M.	44	77	67	125	25.8%	1.15 [0.65, 2.05]
**Total (95% CI)**		**405**		**573**	**100.0%**	**1.02 [0.75, 1.37]**
Total events	146		180			

Heterogeneity: Chi² = 0.25, df = 2 (P = 0.88); I² = 0%
Test for overall effect: Z = 0.12 (P = 0.90)

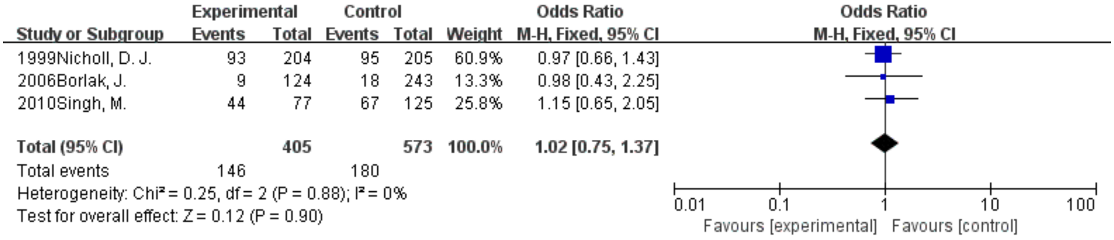

（74）rs1799929(AA)

Study or Subgroup	Experimental Events	Experimental Total	Control Events	Control Total	Weight	Odds Ratio M-H, Fixed, 95% CI
1999Nicholl, D. J.	17	204	18	205	65.9%	0.94 [0.47, 1.89]
2006Borlak, J.	1	124	0	243	1.3%	5.91 [0.24, 146.26]
2010Singh, M.	8	77	12	125	32.8%	1.09 [0.43, 2.80]
**Total (95% CI)**		**405**		**573**	**100.0%**	**1.06 [0.61, 1.83]**
Total events	26		30			

Heterogeneity: Chi² = 1.21, df = 2 (P = 0.55); I² = 0%
Test for overall effect: Z = 0.21 (P = 0.84)

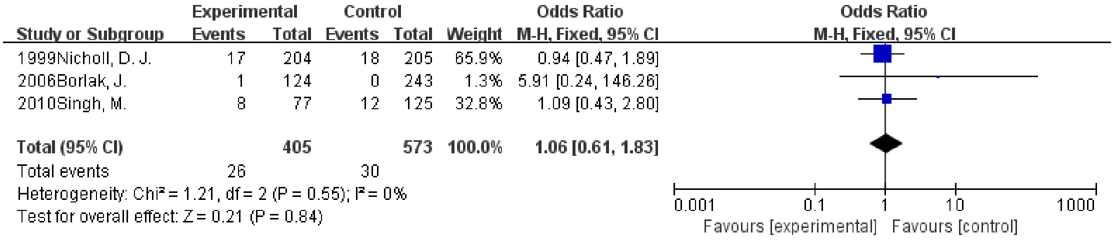

（75）rs1128503（MAF-T）

Study or Subgroup	Experimental Events	Experimental Total	Control Events	Control Total	Weight	Odds Ratio M-H, Fixed, 95% CI
2004Tan, E. K.	127	316	115	278	16.4%	0.95 [0.69, 1.32]
2005Tan, E. K.	226	370	263	412	21.7%	0.89 [0.67, 1.19]
2009Funke, C.	263	600	237	604	29.7%	1.21 [0.96, 1.52]
2009Westerlund, M.	228	550	245	542	32.3%	0.86 [0.68, 1.09]
**Total (95% CI)**		**1836**		**1836**	**100.0%**	**0.98 [0.86, 1.12]**
Total events	844		860			

Heterogeneity: Chi² = 4.84, df = 3 (P = 0.18); I² = 38%
Test for overall effect: Z = 0.24 (P = 0.81)

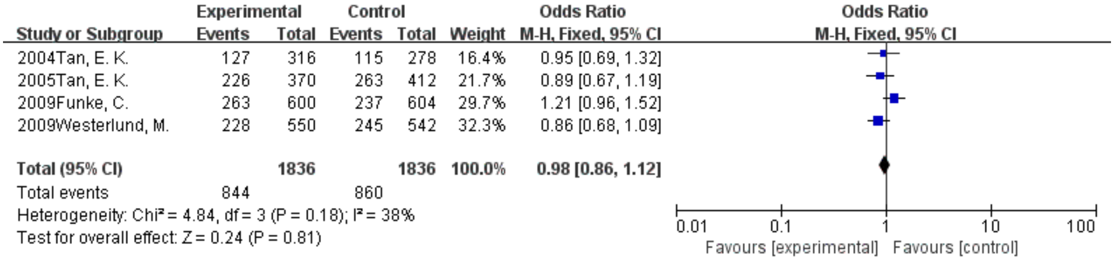

（76）rs1128503（CT+TT）

Study or Subgroup	Experimental Events	Experimental Total	Control Events	Control Total	Weight	Odds Ratio M-H, Fixed, 95% CI
2004Tan, E. K.	102	158	90	139	19.2%	0.99 [0.62, 1.60]
2005Tan, E. K.	156	185	174	206	14.6%	0.99 [0.57, 1.71]
2009Funke, C.	208	300	192	302	33.3%	1.30 [0.92, 1.82]
2009Westerlund, M.	188	275	182	271	32.9%	1.06 [0.74, 1.51]
**Total (95% CI)**		**918**		**918**	**100.0%**	**1.11 [0.91, 1.36]**
Total events	654		638			

Heterogeneity: Chi² = 1.25, df = 3 (P = 0.74); I² = 0%
Test for overall effect: Z = 1.04 (P = 0.30)

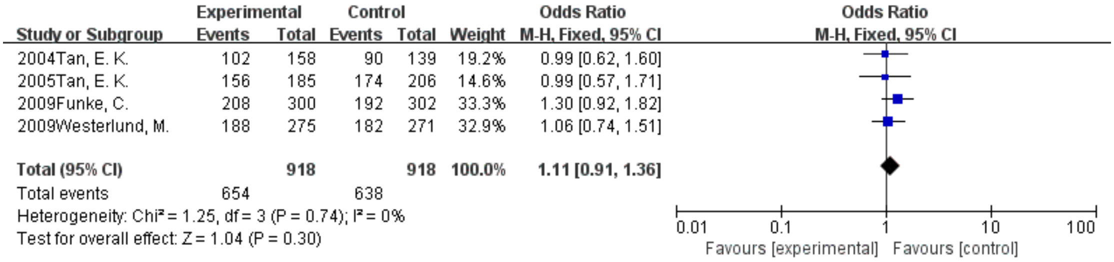

（77）rs1128503（TT）

Study or Subgroup	Experimental Events	Experimental Total	Control Events	Control Total	Weight	Odds Ratio M-H, Random, 95% CI
2004Tan, E. K.	25	158	25	139	19.1%	0.86 [0.47, 1.57]
2005Tan, E. K.	70	185	89	206	27.9%	0.80 [0.53, 1.20]
2009Funke, C.	55	300	45	302	26.6%	1.28 [0.83, 1.97]
2009Westerlund, M.	40	275	63	271	26.3%	0.56 [0.36, 0.87]
**Total (95% CI)**		**918**		**918**	**100.0%**	**0.84 [0.59, 1.19]**
Total events	190		222			

Heterogeneity: Tau² = 0.07; Chi² = 6.99, df = 3 (P = 0.07); I² = 57%
Test for overall effect: Z = 0.98 (P = 0.32)

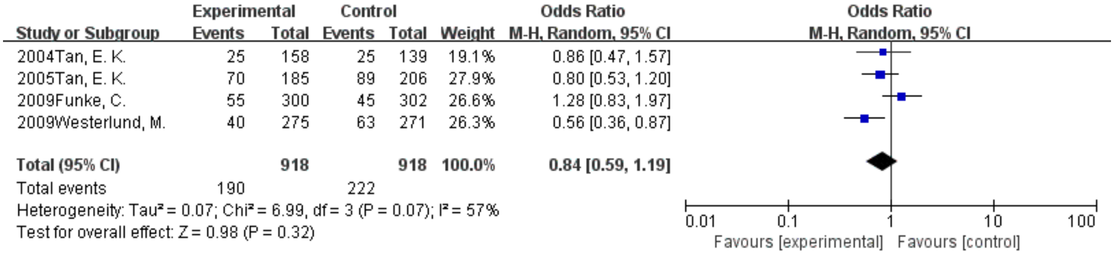

（78）rs1045642(MAF-T)

Study or Subgroup	Experimental Events	Experimental Total	Control Events	Control Total	Weight	Odds Ratio M-H, Fixed, 95% CI
2002Furuno, T.	100	190	98	212	4.0%	1.29 [0.87, 1.91]
2003Dekker, M. C.	96	214	100	206	5.1%	0.86 [0.59, 1.27]
2004Tan, E. K.	168	316	145	278	6.5%	1.04 [0.75, 1.44]
2005Tan, E. K.	131	370	143	412	7.9%	1.03 [0.77, 1.38]
2009Funke, C.	319	600	305	604	12.9%	1.11 [0.89, 1.40]
2009Westerlund, M.	307	554	332	584	13.0%	0.94 [0.75, 1.19]
2009Zschiedrich, K. (i)	266	530	131	246	8.0%	0.88 [0.65, 1.20]
2009Zschiedrich, K. (ii)	42	84	64	122	2.4%	0.91 [0.52, 1.58]
2010Dutheil, F.	212	414	493	964	13.0%	1.00 [0.80, 1.26]
2013Kiyohara, C.	212	476	294	736	11.6%	1.21 [0.96, 1.52]
2015Narayan, S.	318	570	590	1142	15.7%	1.18 [0.96, 1.44]
**Total (95% CI)**		**4318**		**5506**	**100.0%**	**1.06 [0.97, 1.15]**
Total events	2171		2695			

Heterogeneity: Chi² = 7.45, df = 10 (P = 0.68); I² = 0%
Test for overall effect: Z = 1.35 (P = 0.18)

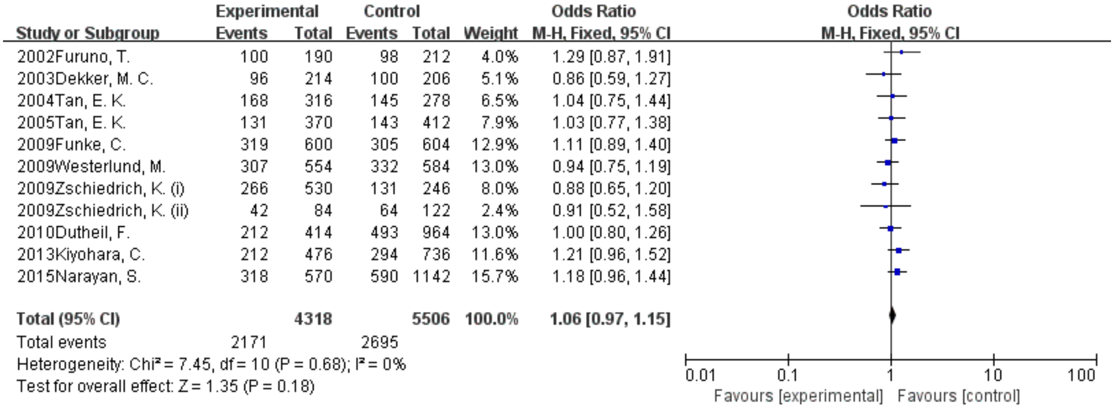

（79）rs1045642(CT+TT)

Study or Subgroup	Experimental Events	Experimental Total	Control Events	Control Total	Weight	Odds Ratio M-H, Fixed, 95% CI
2002Furuno, T.	75	95	78	106	3.7%	1.35 [0.70, 2.59]
2003Dekker, M. C.	81	107	79	103	4.7%	0.95 [0.50, 1.79]
2004Tan, E. K.	123	158	109	139	6.1%	0.97 [0.56, 1.68]
2005Tan, E. K.	104	185	119	206	11.7%	0.94 [0.63, 1.40]
2009Funke, C.	233	300	226	302	12.0%	1.17 [0.80, 1.70]
2009Westerlund, M.	224	277	236	292	10.5%	1.00 [0.66, 1.52]
2009Zschiedrich, K. (i)	196	265	102	123	8.6%	0.58 [0.34, 1.01]
2009Zschiedrich, K. (ii)	30	42	46	61	2.5%	0.82 [0.34, 1.98]
2010Dutheil, F.	162	207	362	482	11.2%	1.19 [0.81, 1.76]
2013Kiyohara, C.	163	238	230	368	13.5%	1.30 [0.92, 1.84]
2015Narayan, S.	223	285	449	571	15.5%	0.98 [0.69, 1.38]
**Total (95% CI)**		**2159**		**2753**	**100.0%**	**1.04 [0.91, 1.19]**
Total events	1614		2036			

Heterogeneity: Chi² = 8.24, df = 10 (P = 0.61); I² = 0%
Test for overall effect: Z = 0.58 (P = 0.56)

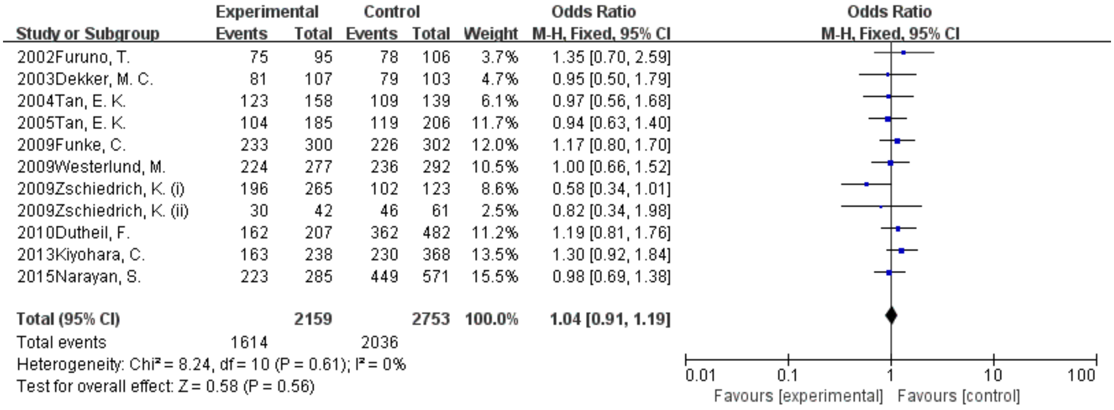

（80）rs1045642(TT)

Study or Subgroup	Experimental Events	Experimental Total	Control Events	Control Total	Weight	Odds Ratio M-H, Fixed, 95% CI
2002Furuno, T.	25	95	20	106	3.5%	1.54 [0.79, 2.99]
2003Dekker, M. C.	15	107	21	103	4.6%	0.64 [0.31, 1.32]
2004Tan, E. K.	45	158	36	139	6.8%	1.14 [0.68, 1.90]
2005Tan, E. K.	27	185	24	206	4.8%	1.30 [0.72, 2.34]
2009Funke, C.	86	300	79	302	14.0%	1.13 [0.79, 1.62]
2009Westerlund, M.	83	277	96	292	16.3%	0.87 [0.61, 1.25]
2009Zschiedrich, K. (i)	70	265	29	123	7.2%	1.16 [0.71, 1.91]
2009Zschiedrich, K. (ii)	12	42	18	61	2.6%	0.96 [0.40, 2.27]
2010Dutheil, F.	50	207	131	482	14.8%	0.85 [0.59, 1.24]
2013Kiyohara, C.	49	238	64	368	9.9%	1.23 [0.81, 1.86]
2015Narayan, S.	95	285	141	571	15.5%	1.52 [1.12, 2.08]
**Total (95% CI)**		**2159**		**2753**	**100.0%**	**1.12 [0.98, 1.28]**
Total events	557		659			

Heterogeneity: Chi² = 11.45, df = 10 (P = 0.32); I² = 13%
Test for overall effect: Z = 1.62 (P = 0.11)

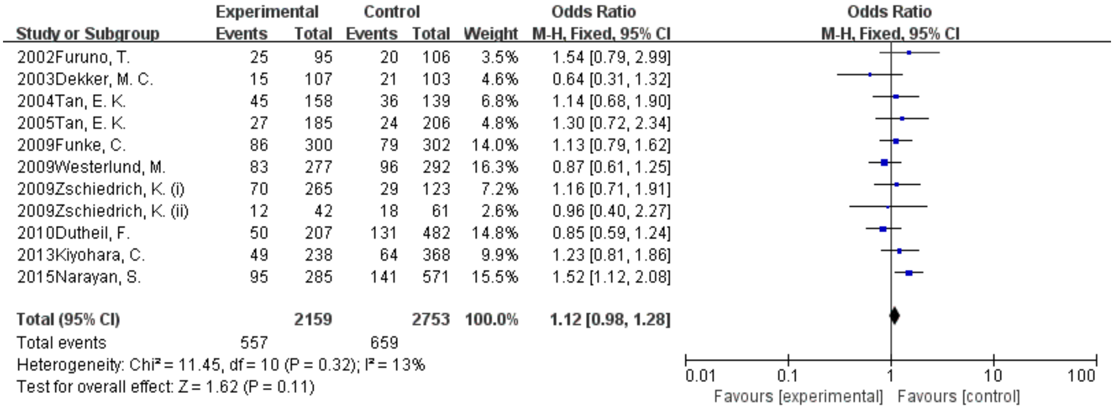

（81）rs2032582(MAF-G, A)

Study or Subgroup	Experimental Events	Experimental Total	Control Events	Control Total	Weight	Odds Ratio M-H, Fixed, 95% CI
2002Furuno, T.	105	190	125	212	6.3%	0.86 [0.58, 1.28]
2004Tan, E. K.	187	316	163	278	8.4%	1.02 [0.74, 1.42]
2005Tan, E. K.	227	370	262	412	11.4%	0.91 [0.68, 1.21]
2009Funke, C.	336	600	351	598	18.4%	0.90 [0.71, 1.13]
2009Westerlund, M.	323	546	326	582	15.3%	1.14 [0.90, 1.44]
2010Dutheil, F.	236	414	552	964	17.0%	0.99 [0.78, 1.25]
2015Narayan, S.	308	562	650	1136	23.1%	0.91 [0.74, 1.11]
**Total (95% CI)**		**2998**		**4182**	**100.0%**	**0.96 [0.87, 1.06]**
Total events	1722		2429			

Heterogeneity: Chi² = 3.28, df = 6 (P = 0.77); I² = 0%
Test for overall effect: Z = 0.80 (P = 0.42)

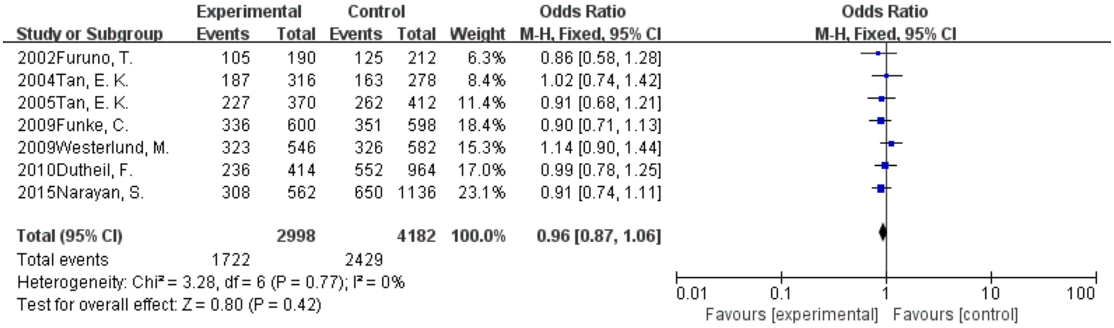

（82）rs2032582(TG,TA)

Study or Subgroup	Experimental Events	Experimental Total	Control Events	Control Total	Weight	Odds Ratio M-H, Fixed, 95% CI
2002Furuno, T.	74	95	85	106	6.9%	0.87 [0.44, 1.72]
2004Tan, E. K.	132	158	115	139	7.8%	1.06 [0.58, 1.95]
2005Tan, E. K.	160	185	179	206	8.9%	0.97 [0.54, 1.73]
2009Funke, C.	243	300	239	299	17.7%	1.07 [0.71, 1.60]
2009Westerlund, M.	233	273	229	291	12.6%	1.58 [1.02, 2.44]
2010Dutheil, F.	168	207	394	482	17.3%	0.96 [0.63, 1.46]
2015Narayan, S.	213	281	464	568	28.8%	0.70 [0.50, 0.99]
**Total (95% CI)**		**1499**		**2091**	**100.0%**	**0.99 [0.83, 1.17]**
Total events	1223		1705			

Heterogeneity: Chi² = 8.49, df = 6 (P = 0.20); I² = 29%
Test for overall effect: Z = 0.17 (P = 0.87)

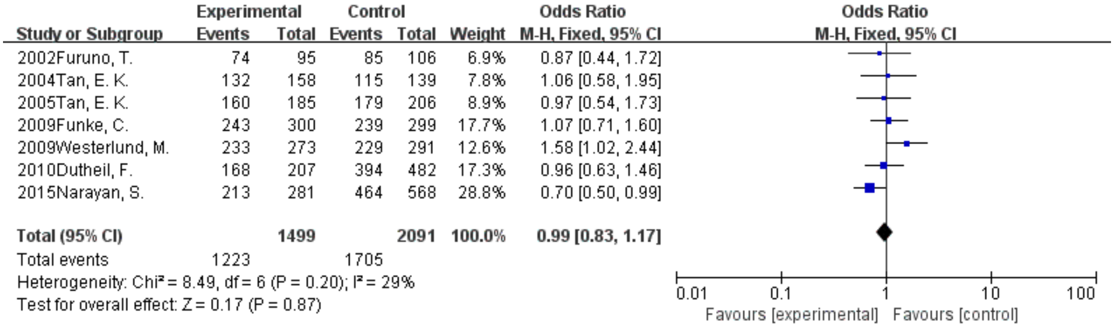

（83）rs2032582(MAF-GG,GA,AA)

Study or Subgroup	Experimental Events	Experimental Total	Control Events	Control Total	Weight	Odds Ratio M-H, Fixed, 95% CI
2002Furuno, T.	31	95	40	106	6.5%	0.80 [0.45, 1.43]
2004Tan, E. K.	55	158	48	139	8.4%	1.01 [0.63, 1.63]
2005Tan, E. K.	67	185	83	206	12.7%	0.84 [0.56, 1.27]
2009Funke, C.	93	300	112	299	19.6%	0.75 [0.53, 1.05]
2009Westerlund, M.	90	273	97	291	16.0%	0.98 [0.69, 1.40]
2010Dutheil, F.	68	207	158	482	16.2%	1.00 [0.71, 1.42]
2015Narayan, S.	95	281	186	568	20.7%	1.05 [0.77, 1.42]
**Total (95% CI)**		**1499**		**2091**	**100.0%**	**0.93 [0.80, 1.07]**
Total events	499		724			

Heterogeneity: Chi² = 3.04, df = 6 (P = 0.80); I² = 0%
Test for overall effect: Z = 1.04 (P = 0.30)

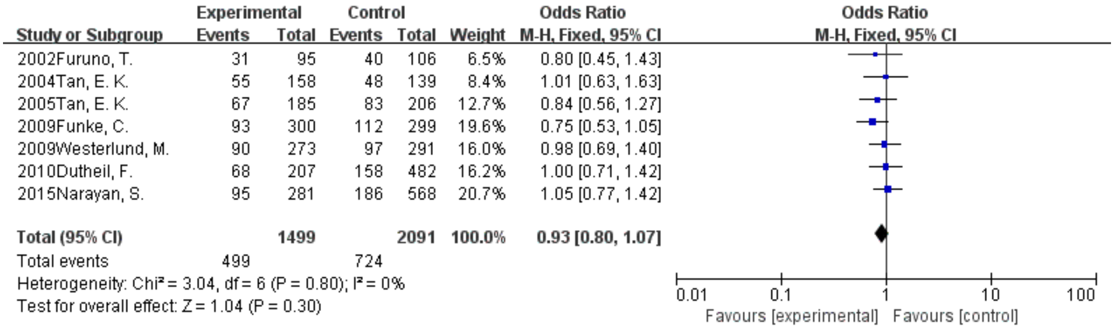

（84）rs11724635(MAF-A)

Study or Subgroup	Experimental Events	Experimental Total	Control Events	Control Total	Weight	Odds Ratio M-H, Fixed, 95% CI
2012Miyake, Y.	188	458	260	714	19.0%	1.22 [0.96, 1.55]
2014Chen, M. L.	371	936	392	974	36.7%	0.97 [0.81, 1.17]
2015Chang, K. H.	530	1192	504	1194	44.3%	1.10 [0.93, 1.29]
**Total (95% CI)**		**2586**		**2882**	**100.0%**	**1.07 [0.96, 1.20]**
Total events	1089		1156			

Heterogeneity: Chi² = 2.15, df = 2 (P = 0.34); I² = 7%
Test for overall effect: Z = 1.30 (P = 0.19)

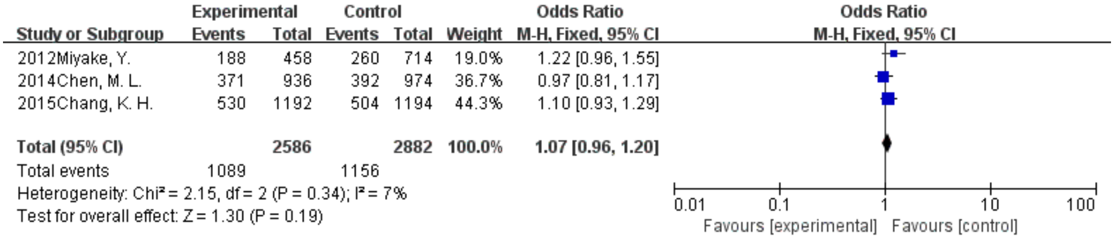

（85）rs11724635(CA+AA)

Study or Subgroup	Experimental Events	Experimental Total	Control Events	Control Total	Weight	Odds Ratio M-H, Fixed, 95% CI
2012Miyake, Y.	145	229	209	357	20.3%	1.22 [0.87, 1.72]
2014Chen, M. L.	294	468	309	487	38.1%	0.97 [0.75, 1.27]
2015Chang, K. H.	413	596	400	597	41.6%	1.11 [0.87, 1.42]
**Total (95% CI)**		**1293**		**1441**	**100.0%**	**1.08 [0.92, 1.27]**
Total events	852		918			

Heterogeneity: Chi² = 1.16, df = 2 (P = 0.56); I² = 0%
Test for overall effect: Z = 0.97 (P = 0.33)

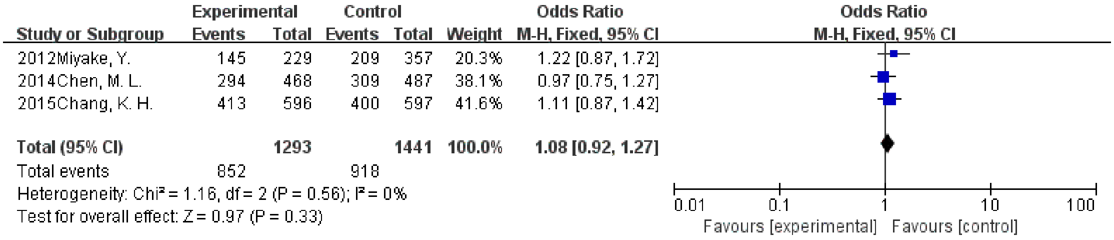

（86）rs11724635(AA)

Study or Subgroup	Experimental Events	Experimental Total	Control Events	Control Total	Weight	Odds Ratio M-H, Fixed, 95% CI
2012Miyake, Y.	43	229	51	357	17.6%	1.39 [0.89, 2.16]
2014Chen, M. L.	77	468	83	487	37.0%	0.96 [0.68, 1.35]
2015Chang, K. H.	117	596	104	597	45.4%	1.16 [0.86, 1.55]
**Total (95% CI)**		**1293**		**1441**	**100.0%**	**1.12 [0.92, 1.37]**
Total events	237		238			

Heterogeneity: Chi^2 = 1.74, df = 2 (P = 0.42); I^2 = 0%
Test for overall effect: Z = 1.16 (P = 0.25)

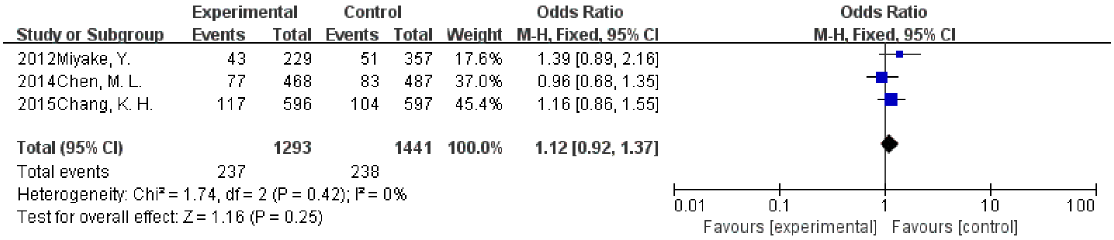

（87）rs11931532(MAF-C)

Study or Subgroup	Experimental Events	Experimental Total	Control Events	Control Total	Weight	Odds Ratio M-H, Fixed, 95% CI
2010Chang, X. L.	588	1240	517	1008	30.7%	0.86 [0.73, 1.01]
2012Miyake, Y.	249	458	412	714	15.0%	0.87 [0.69, 1.11]
2015Guo, J. F.	978	2038	1024	2060	54.2%	0.93 [0.83, 1.06]
**Total (95% CI)**		**3736**		**3782**	**100.0%**	**0.90 [0.82, 0.99]**
Total events	1815		1953			

Heterogeneity: Chi^2 = 0.74, df = 2 (P = 0.69); I^2 = 0%
Test for overall effect: Z = 2.25 (P = 0.02)

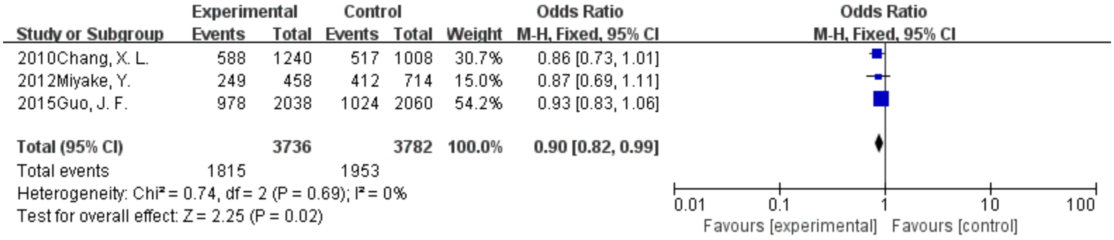

（88）rs11931532(TC+CC)

Study or Subgroup	Experimental Events	Experimental Total	Control Events	Control Total	Weight	Odds Ratio M-H, Fixed, 95% CI
2010Chang, X. L.	462	620	394	504	31.7%	0.82 [0.62, 1.08]
2012Miyake, Y.	175	229	285	357	15.0%	0.82 [0.55, 1.22]
2015Guo, J. F.	777	1019	787	1030	53.2%	0.99 [0.81, 1.22]
**Total (95% CI)**		**1868**		**1891**	**100.0%**	**0.91 [0.78, 1.06]**
Total events	1414		1466			

Heterogeneity: Chi^2 = 1.53, df = 2 (P = 0.46); I^2 = 0%
Test for overall effect: Z = 1.22 (P = 0.22)

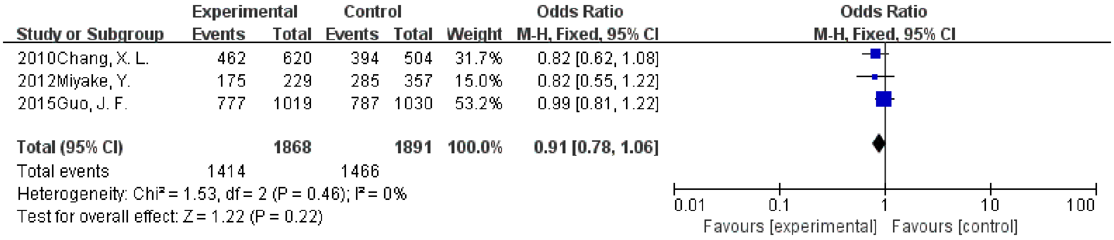

（89）rs11931532(CC)

Study or Subgroup	Experimental Events	Experimental Total	Control Events	Control Total	Weight	Odds Ratio M-H, Fixed, 95% CI
2010Chang, X. L.	126	620	123	504	29.7%	0.79 [0.60, 1.05]
2012Miyake, Y.	74	229	127	357	18.4%	0.86 [0.61, 1.23]
2015Guo, J. F.	201	1019	237	1030	51.9%	0.82 [0.67, 1.02]
**Total (95% CI)**		**1868**		**1891**	**100.0%**	**0.82 [0.70, 0.96]**
Total events	401		487			

Heterogeneity: Chi^2 = 0.15, df = 2 (P = 0.93); I^2 = 0%
Test for overall effect: Z = 2.54 (P = 0.01)

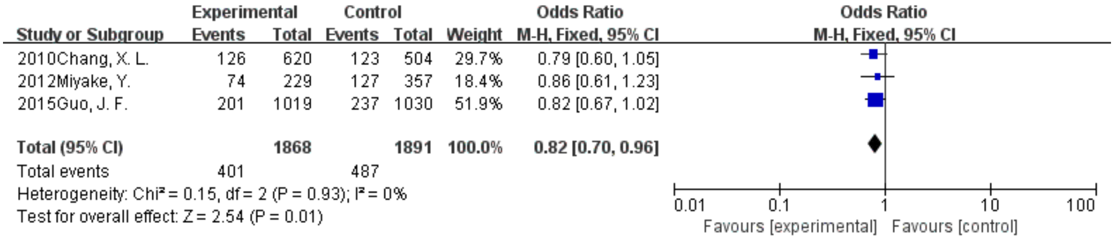

（90）rs660895(MAF-G)

Study or Subgroup	Experimental Events	Experimental Total	Control Events	Control Total	Weight	Odds Ratio M-H, Fixed, 95% CI
2012Ahmed, I.	135	998	408	2244	17.8%	0.70 [0.57, 0.87]
2017Chuang, Y. H. (i)	636	3094	743	3190	47.7%	0.85 [0.76, 0.96]
2017Chuang, Y. H. (ii)	138	1018	411	2256	18.1%	0.70 [0.57, 0.87]
2020Chang, K. H.	245	972	262	946	16.3%	0.88 [0.72, 1.08]
**Total (95% CI)**		**6082**		**8636**	**100.0%**	**0.80 [0.74, 0.87]**
Total events	1154		1824			

Heterogeneity: Chi^2 = 4.75, df = 3 (P = 0.19); I^2 = 37%
Test for overall effect: Z = 5.08 (P < 0.00001)

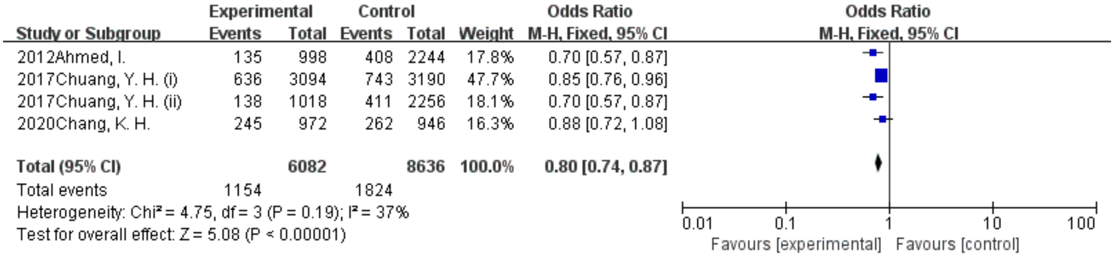

（91）rs660895(AG+GG)

Study or Subgroup	Experimental Events	Experimental Total	Control Events	Control Total	Weight	Odds Ratio M-H, Fixed, 95% CI
2012Ahmed, I.	129	499	371	1122	19.2%	0.71 [0.56, 0.89]
2017Chuang, Y. H. (i)	573	1547	666	1595	46.9%	0.82 [0.71, 0.95]
2017Chuang, Y. H. (ii)	132	509	375	1128	19.6%	0.70 [0.56, 0.89]
2020Chang, K. H.	215	486	222	473	14.3%	0.90 [0.70, 1.16]
**Total (95% CI)**		**3041**		**4318**	**100.0%**	**0.79 [0.71, 0.87]**
Total events	1049		1634			

Heterogeneity: Chi^2 = 3.06, df = 3 (P = 0.38); I^2 = 2%
Test for overall effect: Z = 4.73 (P < 0.00001)

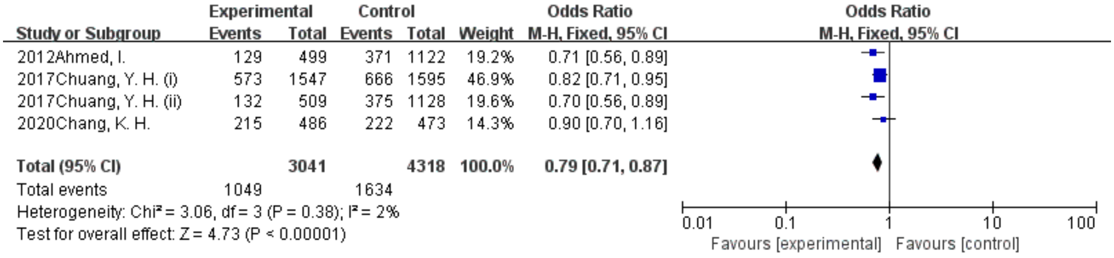

（92）rs660895(GG)

Study or Subgroup	Experimental Events	Experimental Total	Control Events	Control Total	Weight	Odds Ratio M-H, Fixed, 95% CI
2012Ahmed, I.	6	499	37	1122	14.5%	0.36 [0.15, 0.85]
2017Chuang, Y. H. (i)	63	1547	77	1595	46.8%	0.84 [0.60, 1.18]
2017Chuang, Y. H. (ii)	6	509	36	1128	14.2%	0.36 [0.15, 0.86]
2020Chang, K. H.	30	486	40	473	24.5%	0.71 [0.44, 1.16]
**Total (95% CI)**		**3041**		**4318**	**100.0%**	**0.67 [0.52, 0.86]**
Total events	105		190			

Heterogeneity: Chi² = 5.64, df = 3 (P = 0.13); I² = 47%
Test for overall effect: Z = 3.15 (P = 0.002)

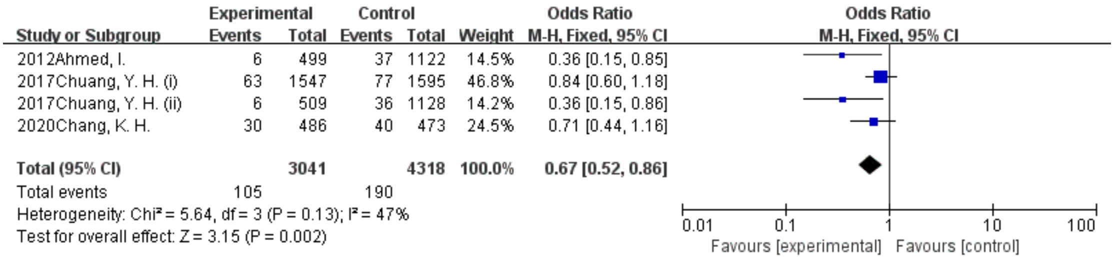

（93）rs12817488(MAF-G)

Study or Subgroup	Experimental Events	Experimental Total	Control Events	Control Total	Weight	Odds Ratio M-H, Fixed, 95% CI
2013Li, N. N.	686	1520	686	1416	43.4%	0.88 [0.76, 1.01]
2014Liu, R. R.	286	682	414	846	23.9%	0.75 [0.62, 0.92]
2015Yu, R. L.	437	1014	523	1036	32.8%	0.74 [0.62, 0.88]
**Total (95% CI)**		**3216**		**3298**	**100.0%**	**0.80 [0.73, 0.89]**
Total events	1409		1623			

Heterogeneity: Chi² = 2.50, df = 2 (P = 0.29); I² = 20%
Test for overall effect: Z = 4.41 (P < 0.0001)

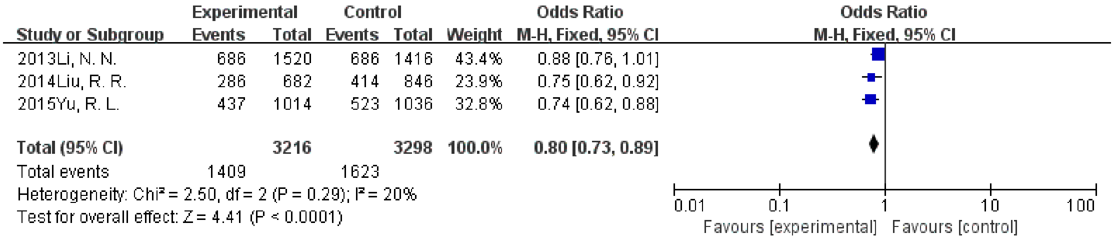

（94）rs12817488(AG+GG)

Study or Subgroup	Experimental Events	Experimental Total	Control Events	Control Total	Weight	Odds Ratio M-H, Fixed, 95% CI
2013Li, N. N.	526	760	508	708	42.1%	0.88 [0.71, 1.11]
2014Liu, R. R.	220	341	315	423	25.9%	0.62 [0.46, 0.85]
2015Yu, R. L.	343	507	385	518	32.0%	0.72 [0.55, 0.95]
**Total (95% CI)**		**1608**		**1649**	**100.0%**	**0.77 [0.66, 0.89]**
Total events	1089		1208			

Heterogeneity: Chi² = 3.45, df = 2 (P = 0.18); I² = 42%
Test for overall effect: Z = 3.47 (P = 0.0005)

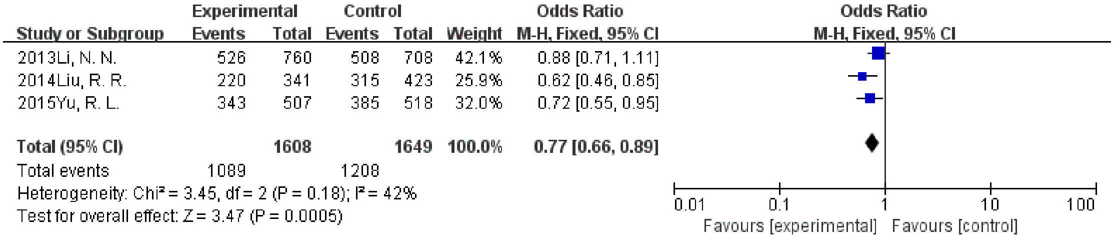

（95）rs12817488(GG)

Study or Subgroup	Experimental Events	Experimental Total	Control Events	Control Total	Weight	Odds Ratio M-H, Fixed, 95% CI
2013Li, N. N.	160	760	178	708	44.4%	0.79 [0.62, 1.01]
2014Liu, R. R.	66	341	99	423	21.7%	0.79 [0.55, 1.12]
2015Yu, R. L.	94	507	138	518	33.9%	0.63 [0.47, 0.84]
**Total (95% CI)**		**1608**		**1649**	**100.0%**	**0.74 [0.62, 0.87]**
Total events	320		415			

Heterogeneity: Chi² = 1.63, df = 2 (P = 0.44); I² = 0%
Test for overall effect: Z = 3.64 (P = 0.0003)

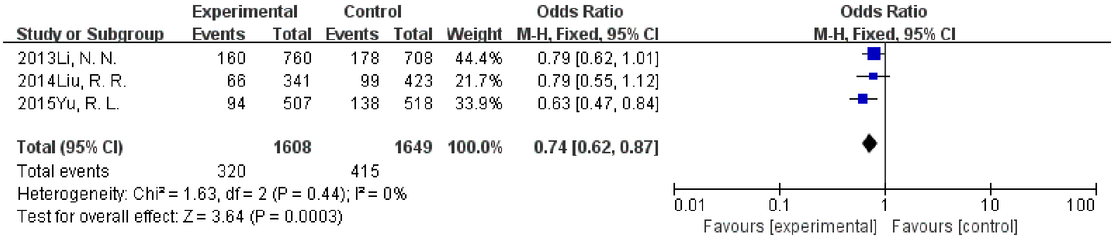

（96）rs1800562, C282Y(MAF-A)

Study or Subgroup	Experimental Events	Experimental Total	Control Events	Control Total	Weight	Odds Ratio M-H, Fixed, 95% CI
2002Borie, C.	5	142	5	114	2.4%	0.80 [0.22, 2.82]
2002Buchanan, D. D.	48	876	84	970	34.4%	0.61 [0.42, 0.88]
2003Dekker, M. C.	23	394	355	5828	19.3%	0.96 [0.62, 1.48]
2006Guerreiro, R. J.	18	264	5	230	2.3%	3.29 [1.20, 9.01]
2007Aamodt, A. H.	60	776	79	1010	28.9%	0.99 [0.70, 1.40]
2008Halling, J.	12	158	30	306	8.6%	0.76 [0.38, 1.52]
2011Greco, V.	3	362	3	360	1.4%	0.99 [0.20, 4.96]
2013Mariani, S.	3	146	2	278	0.6%	2.90 [0.48, 17.52]
2016Mariani, S.	3	170	5	212	2.0%	0.74 [0.18, 3.16]
**Total (95% CI)**		**3288**		**9308**	**100.0%**	**0.89 [0.73, 1.08]**
Total events	175		568			

Heterogeneity: Chi² = 12.90, df = 8 (P = 0.12); I² = 38%
Test for overall effect: Z = 1.21 (P = 0.23)

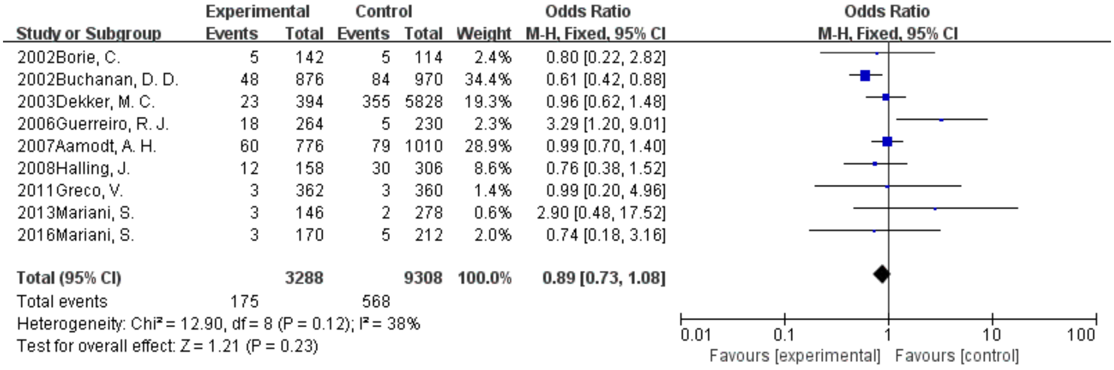

（97）rs1800562, C282Y(GA+AA)

Study or Subgroup	Experimental Events	Experimental Total	Control Events	Control Total	Weight	Odds Ratio M-H, Fixed, 95% CI
2002Borie, C.	5	71	5	57	2.6%	0.79 [0.22, 2.87]
2002Buchanan, D. D.	47	438	80	485	34.2%	0.61 [0.41, 0.90]
2003Dekker, M. C.	21	197	347	2914	19.8%	0.88 [0.55, 1.41]
2006Guerreiro, R. J.	18	132	5	115	2.3%	3.47 [1.25, 9.68]
2007Aamodt, A. H.	58	388	77	505	28.7%	0.98 [0.67, 1.41]
2008Halling, J.	12	79	28	153	8.1%	0.80 [0.38, 1.67]
2011Greco, V.	3	181	3	180	1.5%	0.99 [0.20, 4.99]
2013Mariani, S.	3	73	2	139	0.7%	2.94 [0.48, 17.98]
2016Mariani, S.	3	85	5	106	2.2%	0.74 [0.17, 3.18]
**Total (95% CI)**		**1644**		**4654**	**100.0%**	**0.88 [0.72, 1.08]**
Total events	170		552			

Heterogeneity: Chi² = 12.57, df = 8 (P = 0.13); I² = 36%
Test for overall effect: Z = 1.23 (P = 0.22)

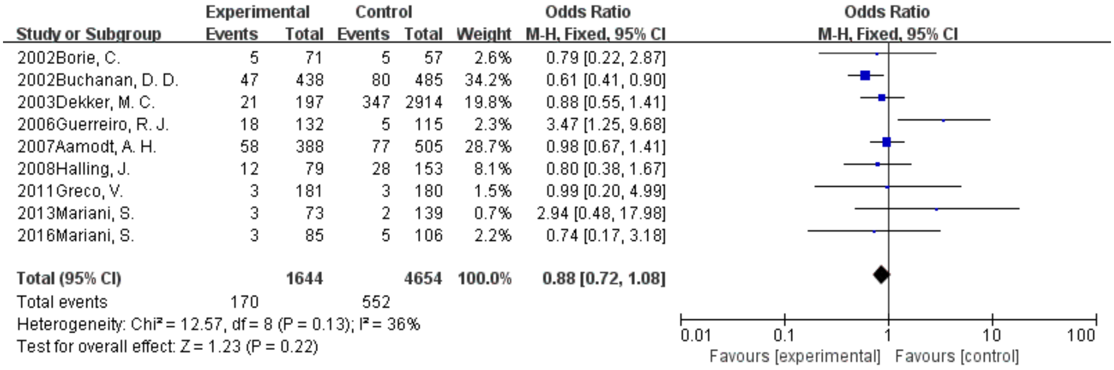

（98）rs1800562, C282Y(AA)

Study or Subgroup	Experimental Events	Experimental Total	Control Events	Control Total	Weight	Odds Ratio M-H, Fixed, 95% CI
2002Borie, C.	0	71	0	57		Not estimable
2002Buchanan, D. D.	1	438	4	485	46.1%	0.28 [0.03, 2.47]
2003Dekker, M. C.	2	197	8	2914	12.2%	3.73 [0.79, 17.66]
2006Guerreiro, R. J.	0	132	0	115		Not estimable
2007Aamodt, A. H.	2	388	2	505	21.0%	1.30 [0.18, 9.29]
2008Halling, J.	0	79	2	153	20.7%	0.38 [0.02, 8.04]
2011Greco, V.	0	181	0	180		Not estimable
2013Mariani, S.	0	73	0	139		Not estimable
2016Mariani, S.	0	85	0	106		Not estimable
**Total (95% CI)**		**1644**		**4654**	**100.0%**	**0.93 [0.35, 2.51]**
Total events	5		16			

Heterogeneity: Chi² = 4.67, df = 3 (P = 0.20); I² = 36%
Test for overall effect: Z = 0.13 (P = 0.89)

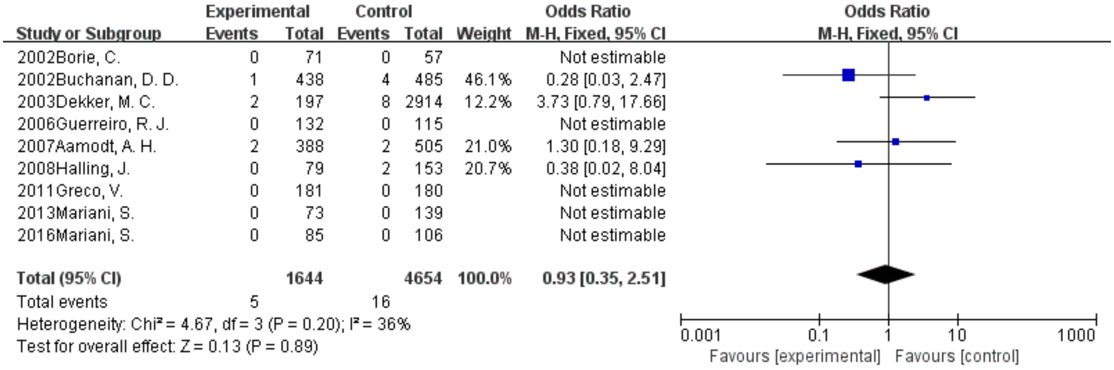

（99）rs1799945, H63D(MAF-G)

Study or Subgroup	Experimental Events	Experimental Total	Control Events	Control Total	Weight	Odds Ratio M-H, Fixed, 95% CI
2002Borie, C.	25	132	20	118	4.9%	1.14 [0.60, 2.19]
2003Dekker, M. C.	54	394	846	5828	26.4%	0.94 [0.70, 1.26]
2006Guerreiro, R. J.	48	264	43	230	10.7%	0.97 [0.61, 1.52]
2007Aamodt, A. H.	80	776	116	1010	25.8%	0.89 [0.66, 1.20]
2008Halling, J.	28	158	48	306	7.7%	1.16 [0.69, 1.93]
2011Greco, V.	63	362	42	360	9.9%	1.60 [1.05, 2.43]
2013Mariani, S.	24	154	42	278	7.2%	1.04 [0.60, 1.79]
2016Mariani, S.	34	194	30	172	7.5%	1.01 [0.59, 1.73]
**Total (95% CI)**		**2434**		**8302**	**100.0%**	**1.03 [0.89, 1.19]**
Total events	356		1187			

Heterogeneity: Chi² = 5.90, df = 7 (P = 0.55); I² = 0%
Test for overall effect: Z = 0.41 (P = 0.68)

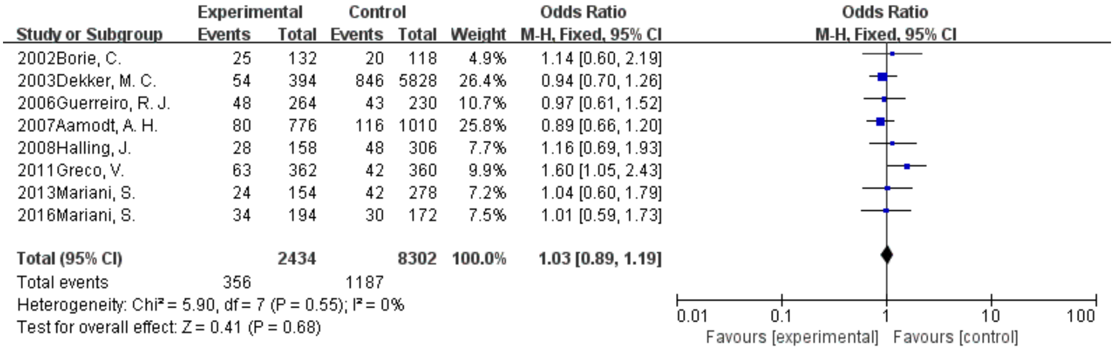

（100）rs1799945, H63D(CG+GG)

Study or Subgroup	Experimental Events	Experimental Total	Control Events	Control Total	Weight	Odds Ratio M-H, Fixed, 95% CI
2002Borie, C.	24	66	20	59	4.8%	1.11 [0.53, 2.33]
2003Dekker, M. C.	52	197	778	2914	25.9%	0.98 [0.71, 1.37]
2006Guerreiro, R. J.	43	132	41	115	10.6%	0.87 [0.51, 1.48]
2007Aamodt, A. H.	73	388	109	505	27.5%	0.84 [0.60, 1.17]
2008Halling, J.	25	79	43	153	7.1%	1.18 [0.66, 2.14]
2011Greco, V.	56	181	39	180	9.6%	1.62 [1.01, 2.60]
2013Mariani, S.	24	77	40	139	7.0%	1.12 [0.61, 2.05]
2016Mariani, S.	33	97	30	86	7.5%	0.96 [0.52, 1.77]
**Total (95% CI)**		**1217**		**4151**	**100.0%**	**1.02 [0.87, 1.21]**
Total events	330		1100			

Heterogeneity: Chi² = 5.75, df = 7 (P = 0.57); I² = 0%
Test for overall effect: Z = 0.27 (P = 0.78)

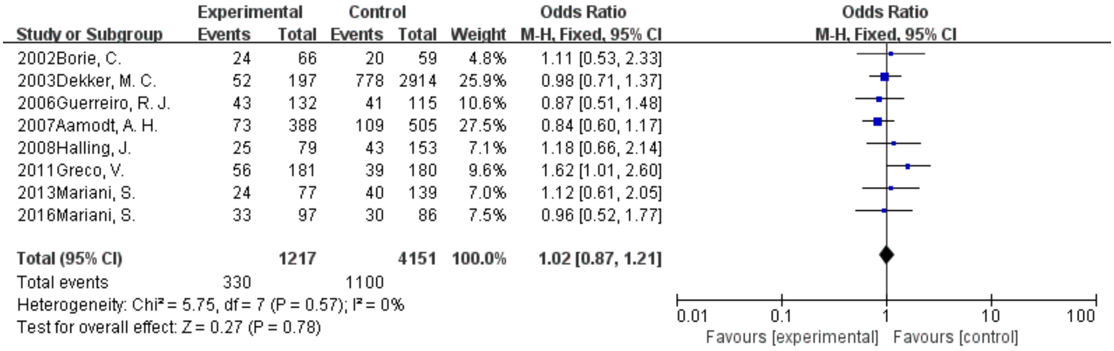

（101）rs1799945, H63D(GG)

Study or Subgroup	Experimental Events	Experimental Total	Control Events	Control Total	Weight	Odds Ratio M-H, Fixed, 95% CI
2002Borie, C.	1	66	0	59	2.0%	2.73 [0.11, 68.19]
2003Dekker, M. C.	2	197	68	2914	33.4%	0.43 [0.10, 1.76]
2006Guerreiro, R. J.	5	132	2	115	8.1%	2.22 [0.42, 11.69]
2007Aamodt, A. H.	7	388	7	505	23.4%	1.31 [0.45, 3.76]
2008Halling, J.	3	79	5	153	12.8%	1.17 [0.27, 5.02]
2011Greco, V.	7	181	3	180	11.3%	2.37 [0.60, 9.33]
2013Mariani, S.	0	77	2	139	7.0%	0.35 [0.02, 7.49]
2016Mariani, S.	1	97	0	86	2.0%	2.69 [0.11, 66.88]
**Total (95% CI)**		**1217**		**4151**	**100.0%**	**1.18 [0.70, 1.99]**
Total events	26		87			

Heterogeneity: Chi² = 4.67, df = 7 (P = 0.70); I² = 0%
Test for overall effect: Z = 0.63 (P = 0.53)

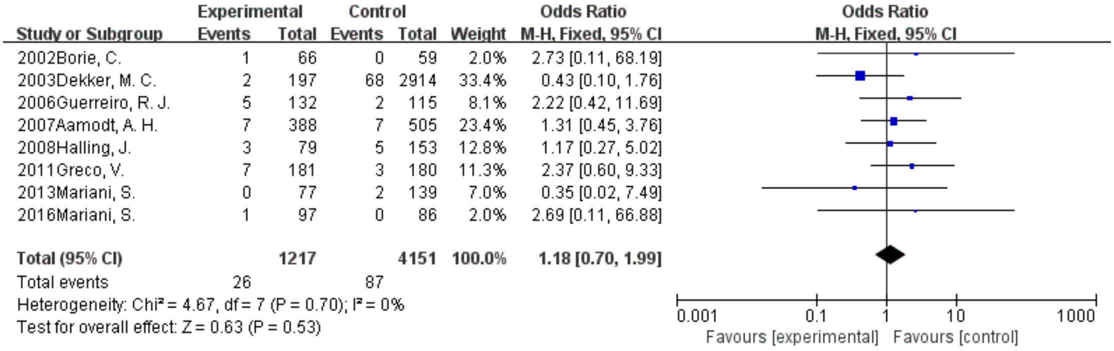

（102）rs1801133(MAF-T)117

Study or Subgroup	Experimental Events	Experimental Total	Control Events	Control Total	Weight	Odds Ratio M-H, Random, 95% CI
2000Yasui, K.	82	180	43	106	6.7%	1.23 [0.75, 1.99]
2005Wullner, U.	230	684	218	684	10.5%	1.08 [0.86, 1.36]
2006Religa, D.	76	228	52	200	7.6%	1.42 [0.94, 2.16]
2006Todorovic, Z.	81	226	46	106	6.9%	0.73 [0.46, 1.17]
2007Caccamo, D.	53	98	67	172	6.5%	1.85 [1.12, 3.05]
2009Camicioli, R. M.	36	102	41	98	5.6%	0.76 [0.43, 1.34]
2009Rodriguez-Oroz, M. C.	47	154	25	56	5.1%	0.54 [0.29, 1.02]
2009Yuan, R. Y.	53	152	52	220	7.0%	1.73 [1.10, 2.73]
2011Fong, C. S.	112	422	107	436	9.3%	1.11 [0.82, 1.51]
2012Gorgone, G.	67	120	66	164	6.8%	1.88 [1.17, 3.02]
2014Kumudini, N.	31	302	62	832	7.1%	1.42 [0.90, 2.23]
2014Liao, Q.	541	1530	582	1434	11.6%	0.80 [0.69, 0.93]
2016Zahra, C.	102	302	216	622	9.5%	0.96 [0.72, 1.28]
**Total (95% CI)**		**4500**		**5130**	**100.0%**	**1.11 [0.93, 1.34]**
Total events	1511		1577			

Heterogeneity: Tau² = 0.07; Chi² = 39.49, df = 12 (P < 0.0001); I² = 70%
Test for overall effect: Z = 1.14 (P = 0.26)

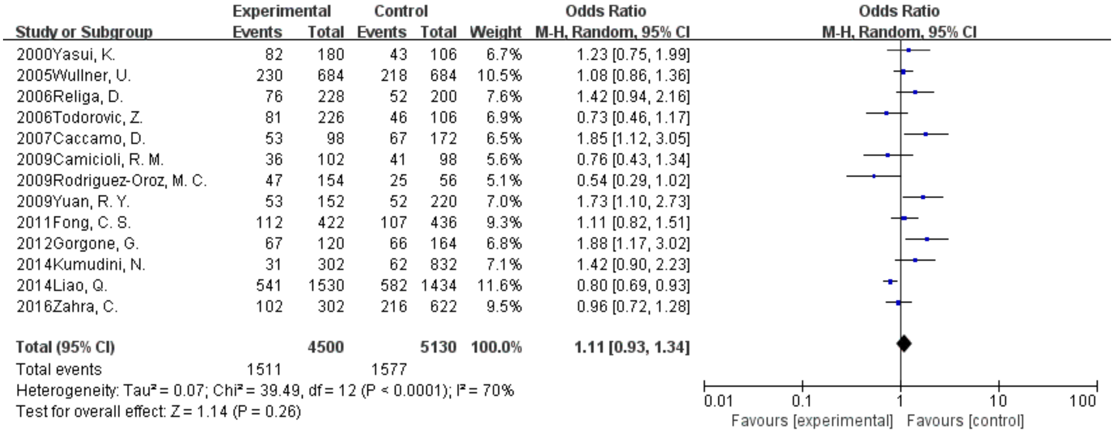

（103）rs1801133(CT+TT)

Study or Subgroup	Experimental Events	Experimental Total	Control Events	Control Total	Weight	Odds Ratio M-H, Random, 95% CI
2000Yasui, K.	61	90	32	53	6.2%	1.38 [0.68, 2.80]
2005Wullner, U.	192	342	174	342	10.8%	1.24 [0.91, 1.67]
2006Religa, D.	66	114	45	100	7.9%	1.68 [0.98, 2.89]
2006Todorovic, Z.	66	113	37	53	6.3%	0.61 [0.30, 1.22]
2007Caccamo, D.	37	49	52	86	5.6%	2.02 [0.92, 4.40]
2009Camicioli, R. M.	31	51	32	49	5.3%	0.82 [0.36, 1.86]
2009Rodriguez-Oroz, M. C.	44	77	19	28	4.6%	0.63 [0.25, 1.57]
2009Yuan, R. Y.	45	76	45	110	7.3%	2.10 [1.16, 3.80]
2011Fong, C. S.	95	211	92	218	9.8%	1.12 [0.77, 1.64]
2012Gorgone, G.	45	60	51	82	6.0%	1.82 [0.87, 3.80]
2014Kumudini, N.	29	151	61	416	8.5%	1.38 [0.85, 2.25]
2014Liao, Q.	444	765	486	717	11.8%	0.66 [0.53, 0.81]
2016Zahra, C.	85	151	175	311	9.7%	1.00 [0.68, 1.48]
**Total (95% CI)**		**2250**		**2565**	**100.0%**	**1.15 [0.90, 1.47]**
Total events	1240		1301			

Heterogeneity: Tau² = 0.12; Chi² = 38.02, df = 12 (P = 0.0002); I² = 68%
Test for overall effect: Z = 1.12 (P = 0.26)

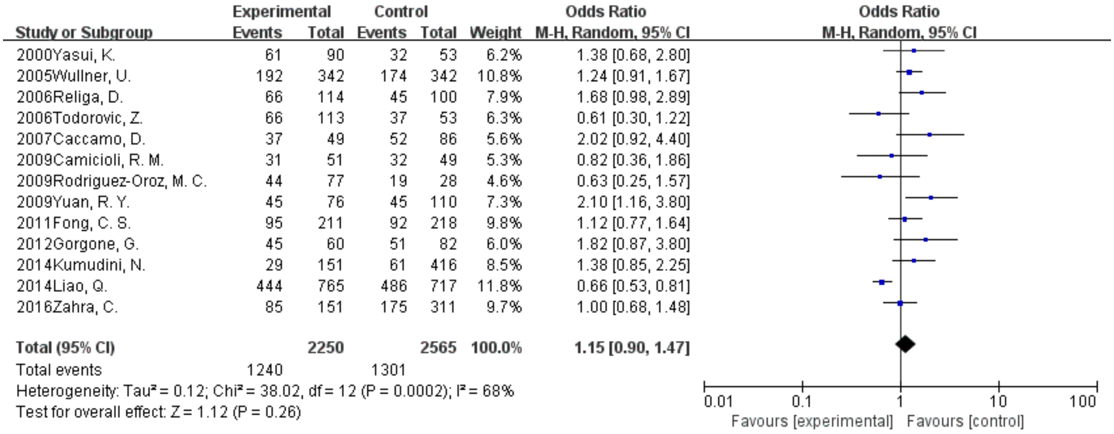

（104）rs1801133(TT)

Study or Subgroup	Experimental Events	Experimental Total	Control Events	Control Total	Weight	Odds Ratio M-H, Fixed, 95% CI
2000Yasui, K.	21	90	11	53	4.6%	1.16 [0.51, 2.65]
2005Wullner, U.	38	342	44	342	17.1%	0.85 [0.53, 1.34]
2006Religa, D.	10	114	7	100	3.0%	1.28 [0.47, 3.49]
2006Todorovic, Z.	15	113	9	53	4.6%	0.75 [0.30, 1.84]
2007Caccamo, D.	16	49	15	86	3.2%	2.29 [1.01, 5.19]
2009Camicioli, R. M.	5	51	9	49	3.6%	0.48 [0.15, 1.56]
2009Rodriguez-Oroz, M. C.	3	77	6	28	3.7%	0.15 [0.03, 0.64]
2009Yuan, R. Y.	8	76	7	110	2.2%	1.73 [0.60, 4.99]
2011Fong, C. S.	17	211	15	218	5.9%	1.19 [0.58, 2.44]
2012Gorgone, G.	22	60	15	82	3.5%	2.59 [1.20, 5.57]
2014Kumudini, N.	2	151	1	416	0.2%	5.57 [0.50, 61.88]
2014Liao, Q.	97	765	96	717	37.8%	0.94 [0.69, 1.27]
2016Zahra, C.	17	151	41	311	10.4%	0.84 [0.46, 1.53]
**Total (95% CI)**		**2250**		**2565**	**100.0%**	**1.02 [0.85, 1.23]**
Total events	271		276			

Heterogeneity: Chi² = 22.74, df = 12 (P = 0.03); I² = 47%
Test for overall effect: Z = 0.24 (P = 0.81)

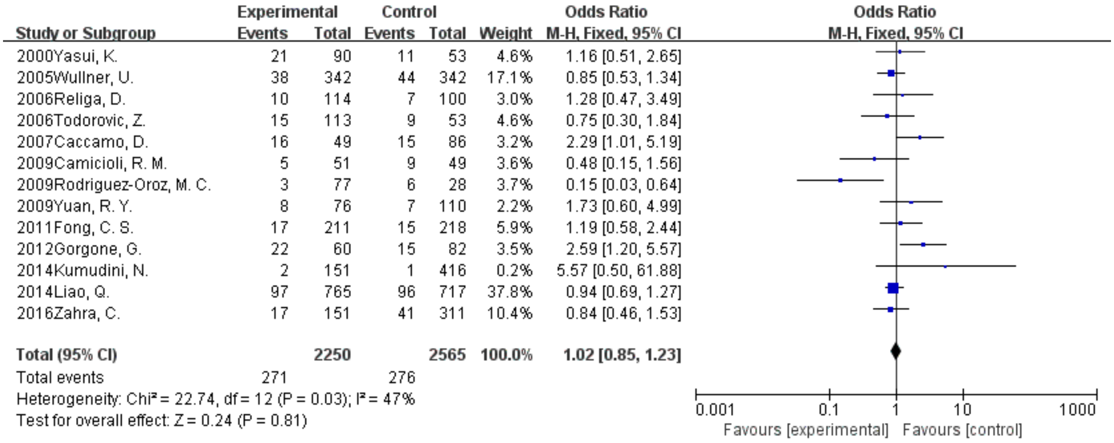

（105）rs1801131(MAF-C)

Study or Subgroup	Experimental Events	Experimental Total	Control Events	Control Total	Weight	Odds Ratio M-H, Random, 95% CI
2005Wullner, U.	202	684	233	684	41.3%	0.81 [0.65, 1.02]
2009Camicioli, R. M.	32	100	24	98	18.8%	1.45 [0.78, 2.71]
2009Rodriguez-Oroz, M. C.	59	164	13	52	16.0%	1.69 [0.83, 3.41]
2009Yuan, R. Y.	31	152	48	220	23.8%	0.92 [0.55, 1.53]
**Total (95% CI)**		**1100**		**1054**	**100.0%**	**1.05 [0.75, 1.47]**
Total events	324		318			

Heterogeneity: Tau² = 0.06; Chi² = 6.07, df = 3 (P = 0.11); I² = 51%
Test for overall effect: Z = 0.27 (P = 0.79)

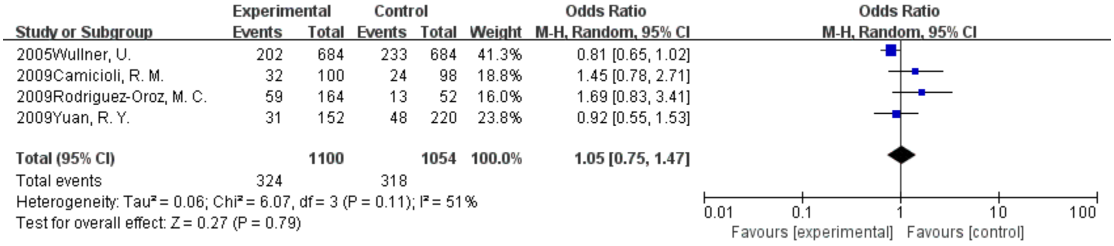

（106）rs1801131(AC+CC)

Study or Subgroup	Experimental Events	Experimental Total	Control Events	Control Total	Weight	Odds Ratio M-H, Fixed, 95% CI
2005Wullner, U.	171	342	185	342	70.7%	0.85 [0.63, 1.15]
2009Camicioli, R. M.	26	50	21	49	7.8%	1.44 [0.65, 3.19]
2009Rodriguez-Oroz, M. C.	49	82	11	26	5.1%	2.02 [0.83, 4.95]
2009Yuan, R. Y.	26	76	40	110	16.4%	0.91 [0.49, 1.68]
**Total (95% CI)**		**550**		**527**	**100.0%**	**0.97 [0.76, 1.23]**
Total events	272		257			

Heterogeneity: Chi² = 4.37, df = 3 (P = 0.22); I² = 31%
Test for overall effect: Z = 0.28 (P = 0.78)

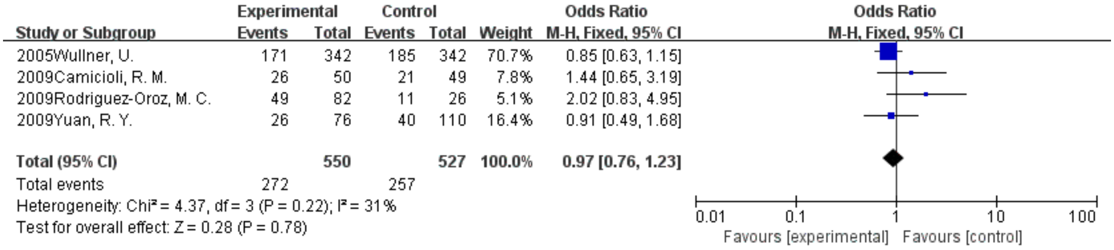

(107) rs1801131(CC)

Study or Subgroup	Experimental Events	Experimental Total	Control Events	Control Total	Weight	Odds Ratio M-H, Fixed, 95% CI
2005Wullner, U.	31	342	48	342	79.2%	0.61 [0.38, 0.99]
2009Camicioli, R. M.	6	50	3	49	4.8%	2.09 [0.49, 8.88]
2009Rodriguez-Oroz, M. C.	10	82	2	26	4.8%	1.67 [0.34, 8.15]
2009Yuan, R. Y.	5	76	8	110	11.1%	0.90 [0.28, 2.86]
**Total (95% CI)**		**550**		**527**	**100.0%**	**0.77 [0.51, 1.14]**
Total events	52		61			

Heterogeneity: Chi² = 3.71, df = 3 (P = 0.29); I² = 19%
Test for overall effect: Z = 1.31 (P = 0.19)

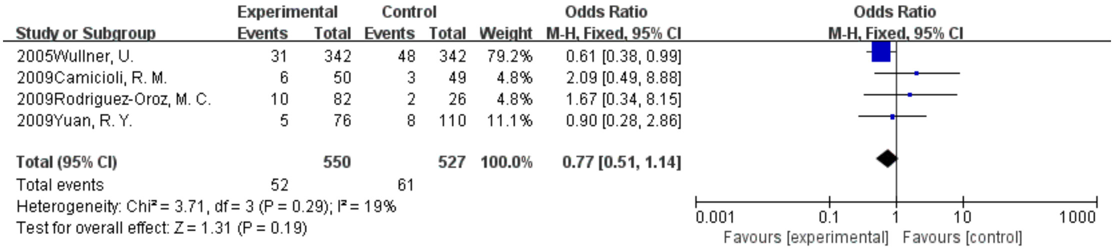

Supplementary Figure 3. Funnel plots of the association between each candidate variant included in quantitative analysis and risk of PD.

(1) rs1800629, G-308A(MAF-A)

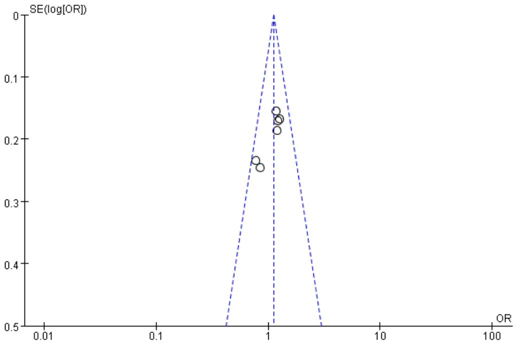

(2) rs1800629, G-308A(GA+AA)

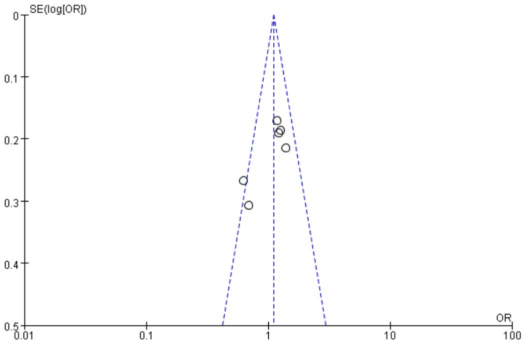

(3) rs1800629, G-308A(AA)

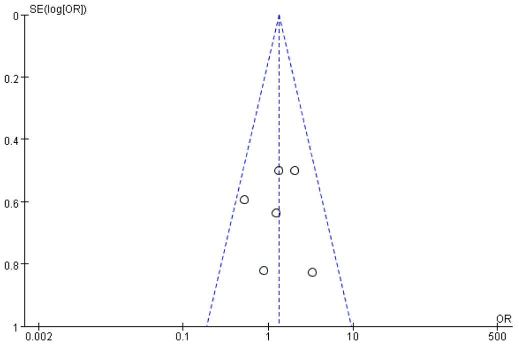

(4) rs1799964, T-1031C(MAF-C)

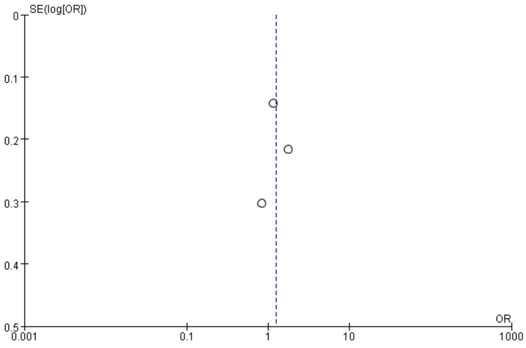

(5) rs1799964, T-1031C(TC+CC)

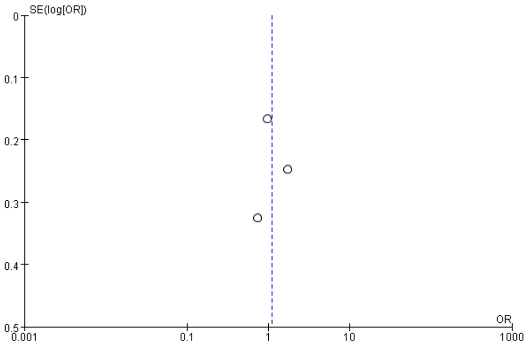

(6) rs1799964, T-1031C(CC)

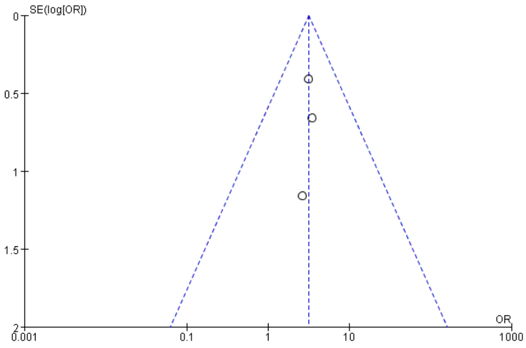

(7) rs1800587, C-889T（MAF-T）

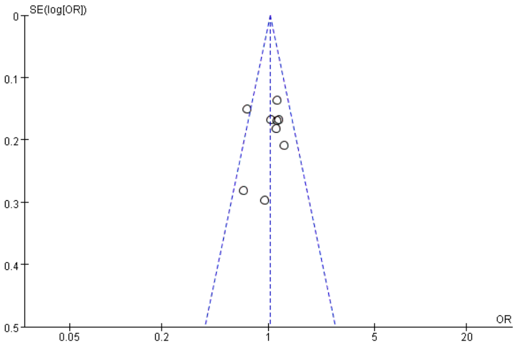

(8) rs1800587, C-889T（CT+TT）

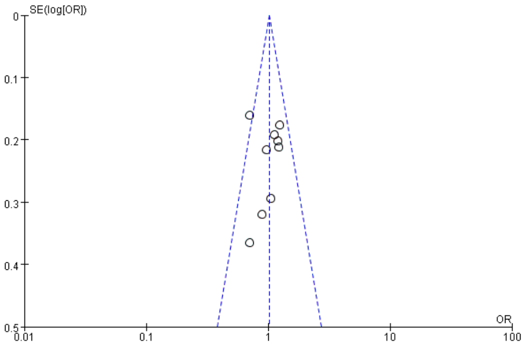

(9) rs1800587, C-889T（TT）

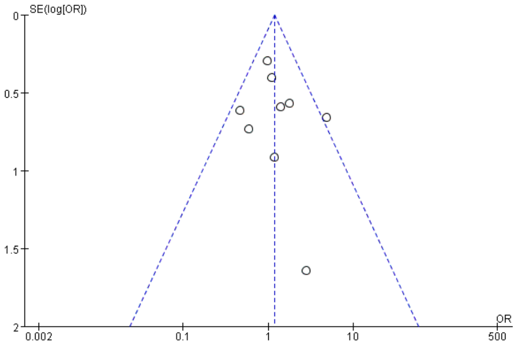

(10) rs16944, C-511T(MAF-T)

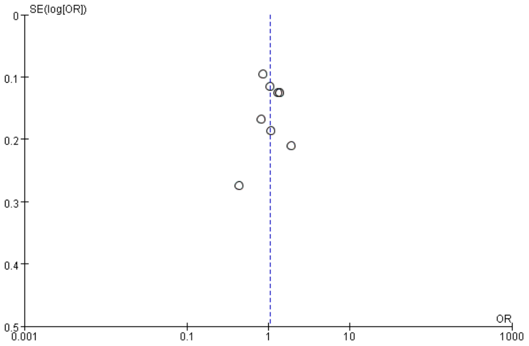

(11) rs16944, C-511T(CT+TT)

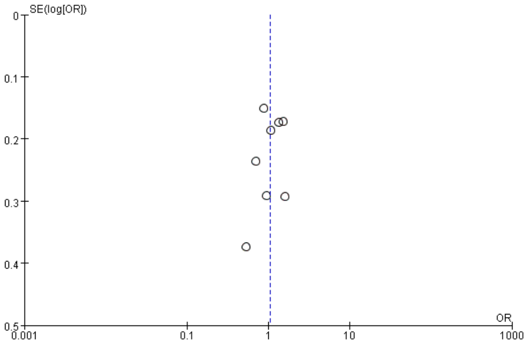

(12) rs16944, C-511T(TT)

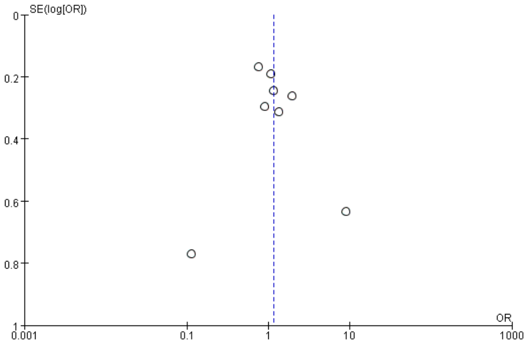

(13) rs1800795, G-174C(MAF-C)

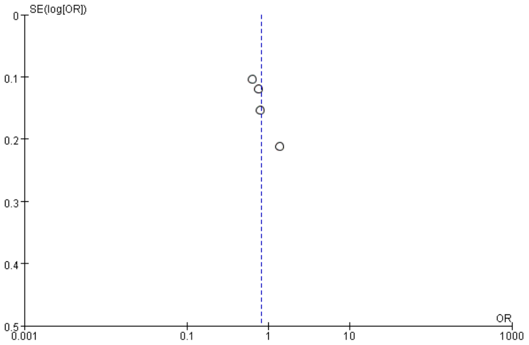

(14) rs1800795, G-174C(GC+CC)

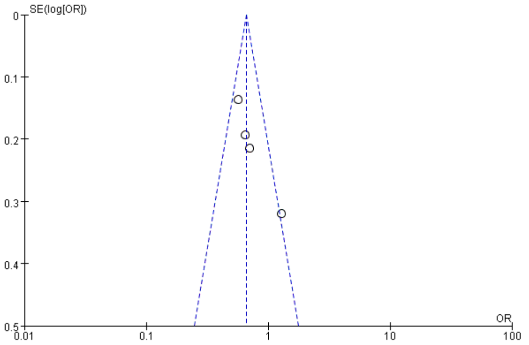

(15) rs1800795, G-174C(CC)

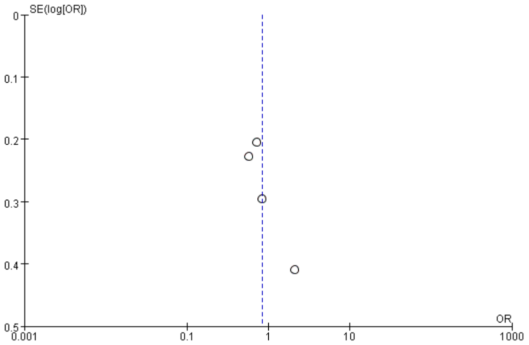

(16) rs1800896, -1082GA（MAF-G）

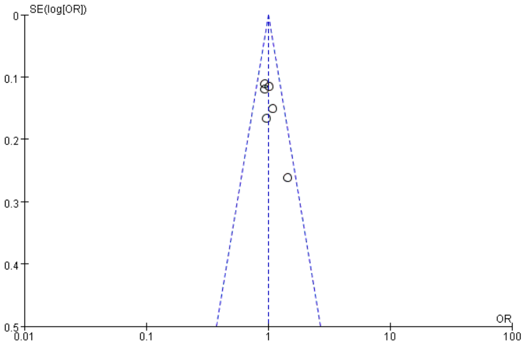

(17) rs1800896, -1082GA（AG+GG）

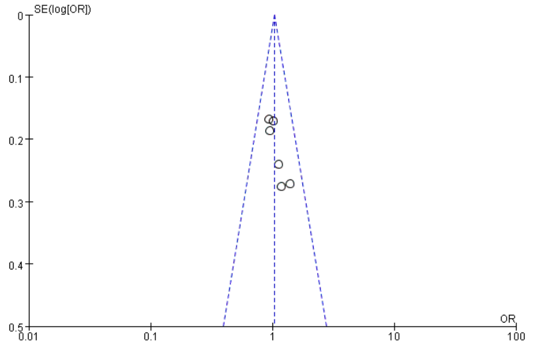

(18) rs1800896, -1082GA（GG）

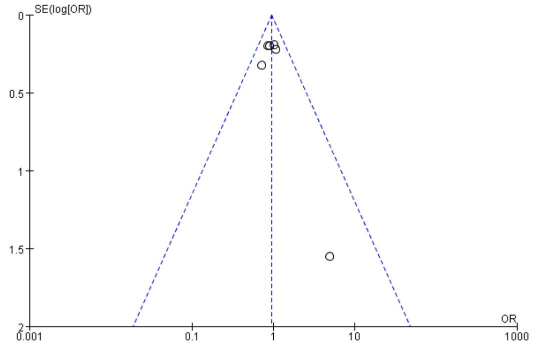

(19) rs1800871, -592CA(MAF-A)

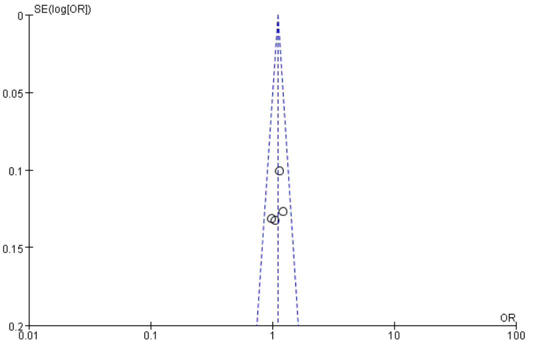

(20) rs1800871, -592CA(CA+AA)

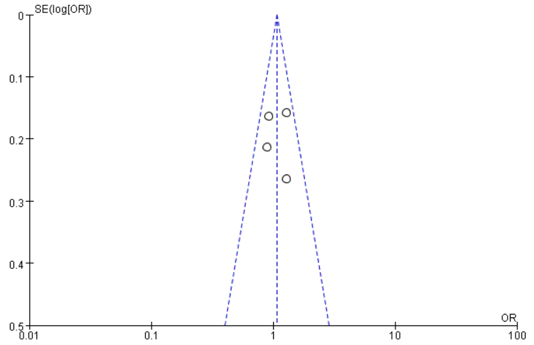

(21) rs1800871, -592CA(AA)

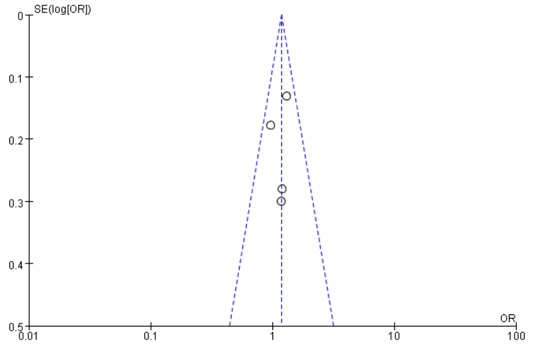

(22) rs2682826(MAF-T)

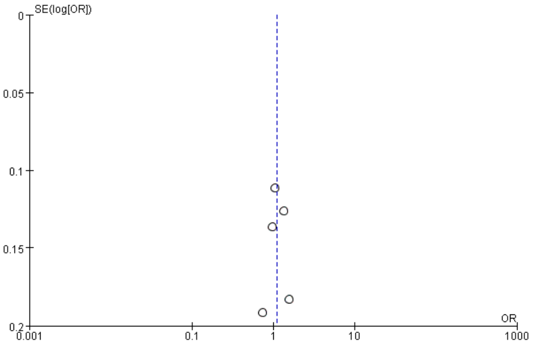

(23) rs2682826(CT+TT)

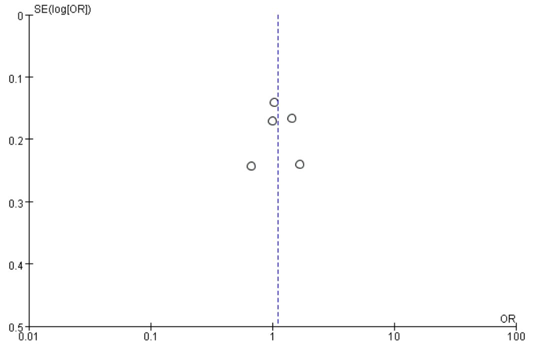

(24) rs2682826(TT)

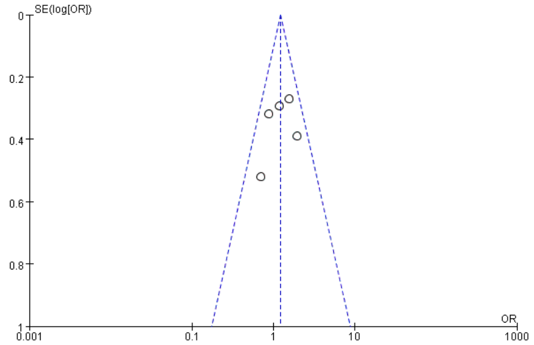

(25) rs1060826(MAF-A)

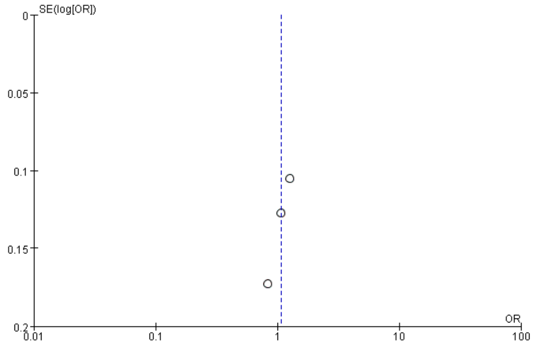

(26) rs1060826(GA+AA)

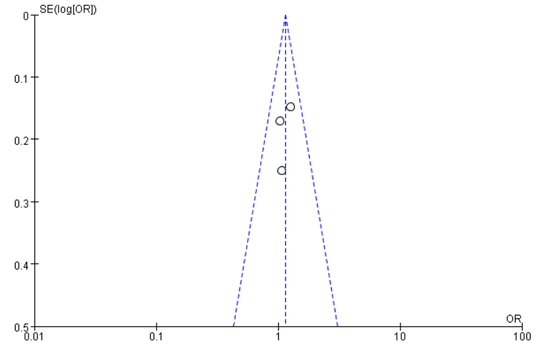

(27) rs1060826(AA)

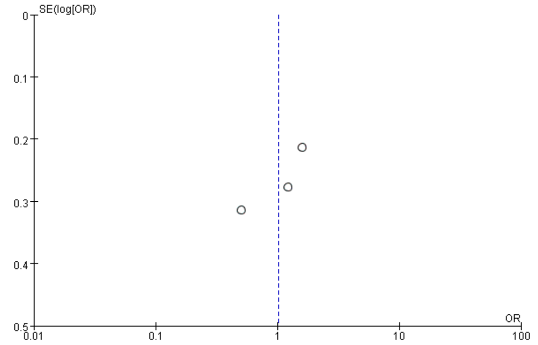

(28) rs4880(MAF-C)

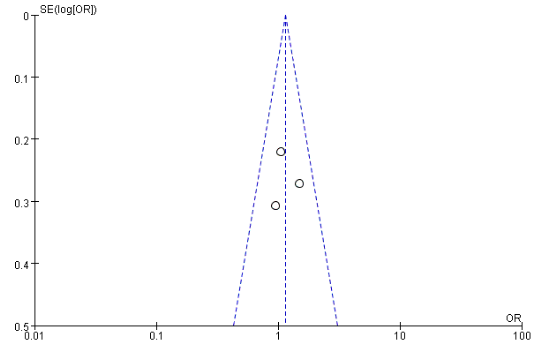

(29) rs4880(TC+CC)

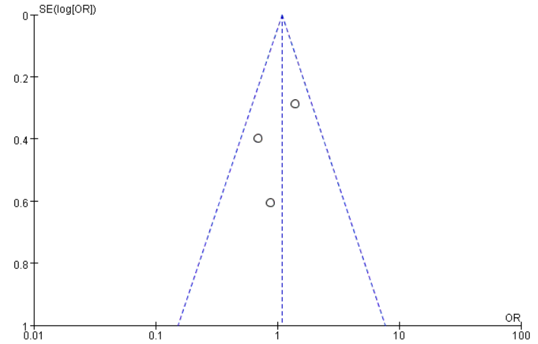

(30) rs4880(CC)

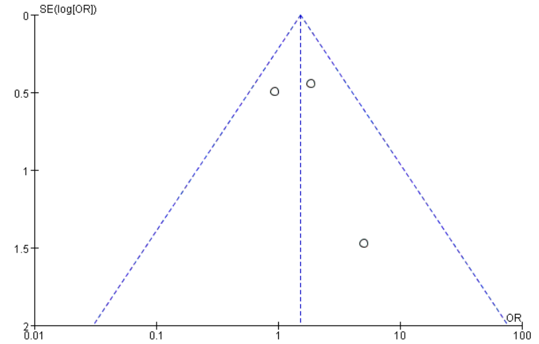

(31) rs6706649（MAF-A）

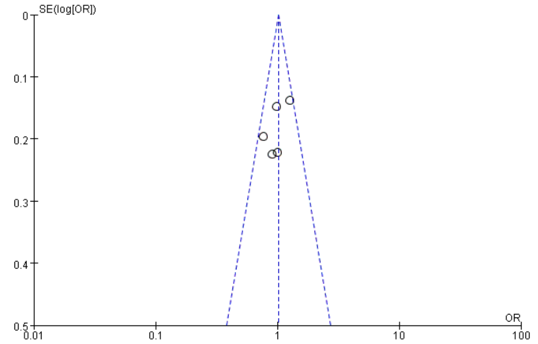

(32) rs6706649（GA+AA）

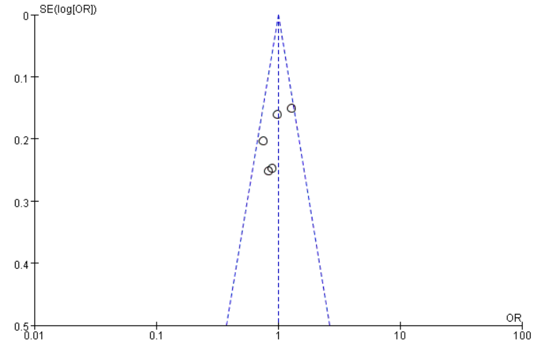

(33) rs6706649（AA）

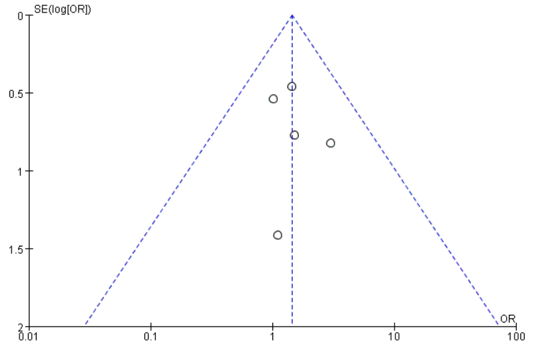

(34) rs6721961(MAF-A)

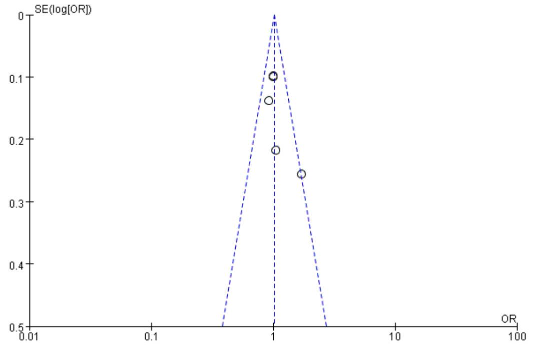

(35) rs6721961(AC+AA)

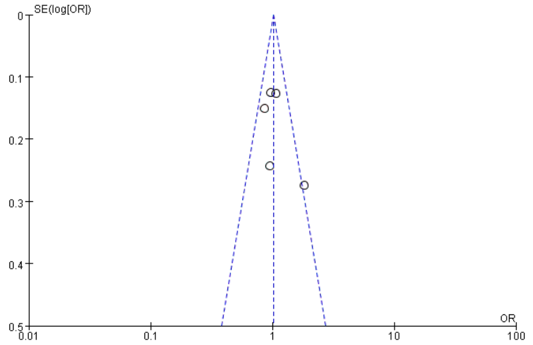

(36) rs6721961(AA)

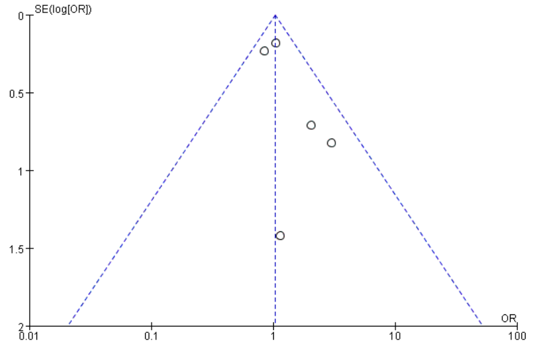

(37) rs35652124 (MAF-G)

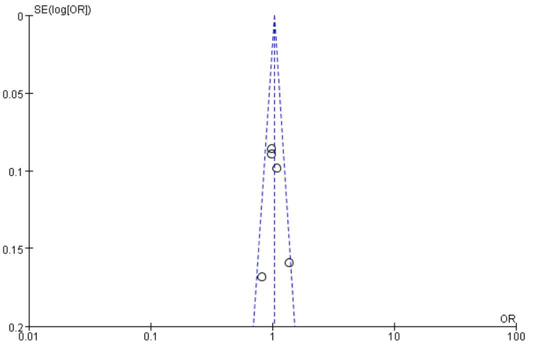

(38) rs35652124 (AG+GG)

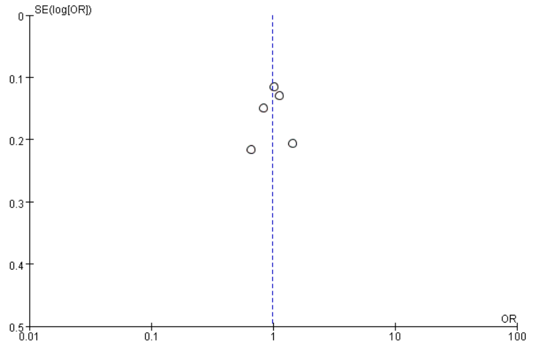

(39) rs35652124 (GG)

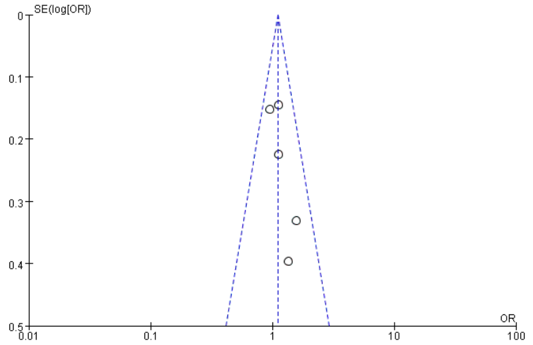

(40) rs2706110(MAF-A)

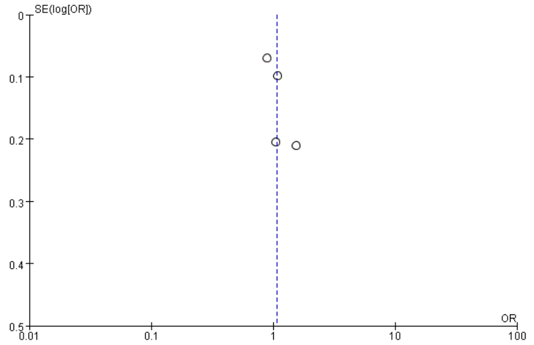

(41) rs2706110(GA+AA)

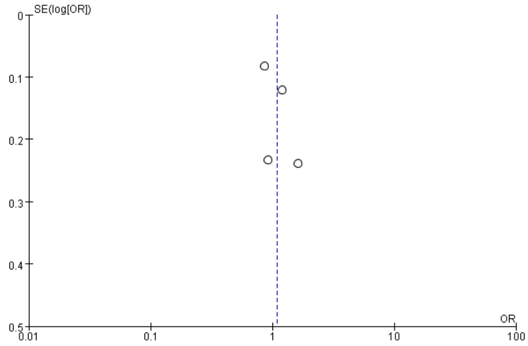

(42) rs2706110(AA)

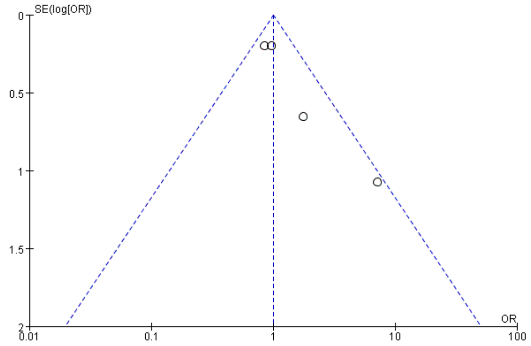

(43) rs10183914 (MAF-A)

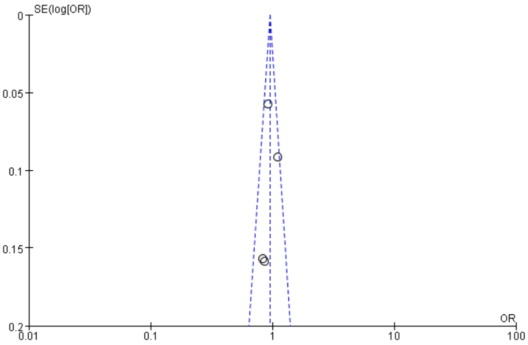

(44) rs10183914 (GA+AA)

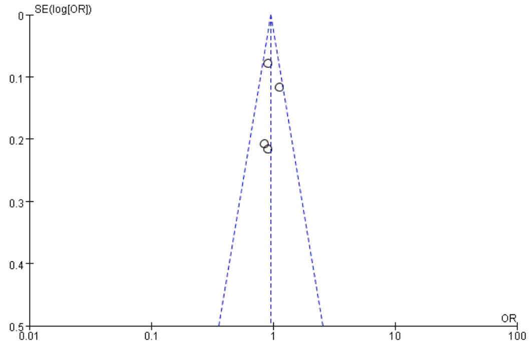

(45) rs10183914 (AA)

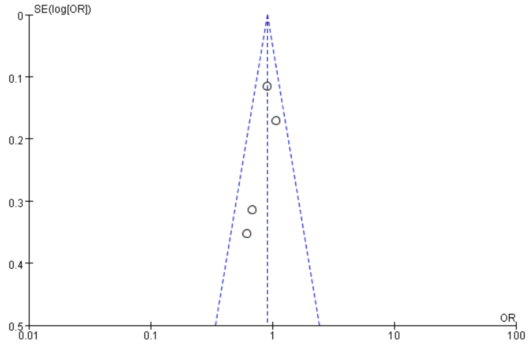

(46) rs1806649 (MAF-A)

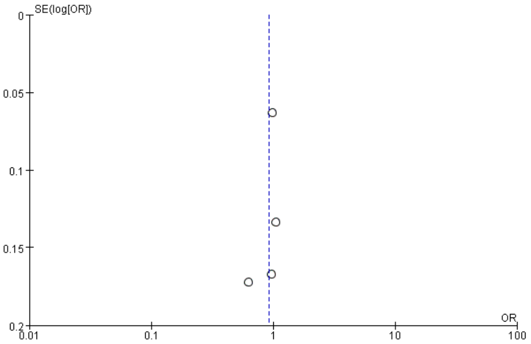

(47) rs1806649 (GA+AA)

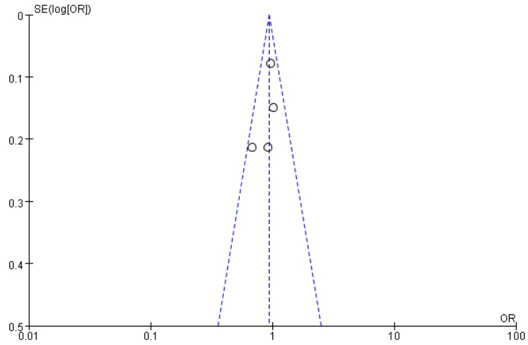

(48) rs1806649 (AA)

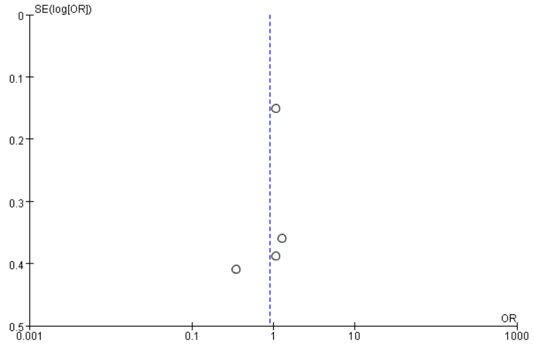

(49) rs2001350(MAF-G)

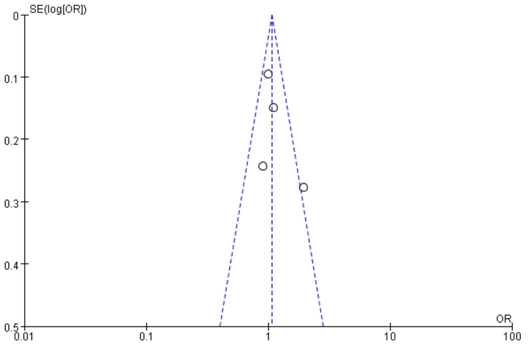

(50) rs2001350(AG+GG)

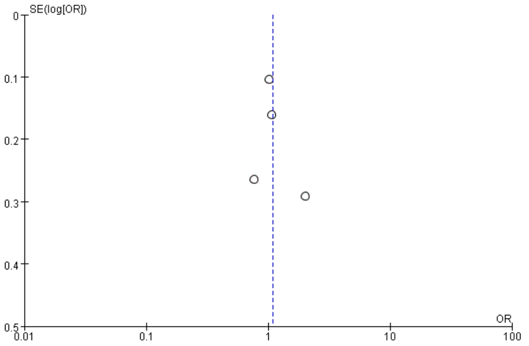

(51) rs2001350(GG)

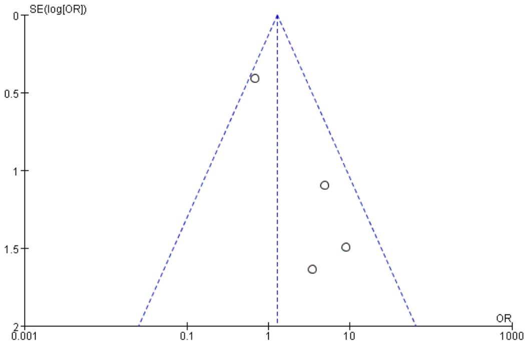

(52) rs3892097 (MAF-A)

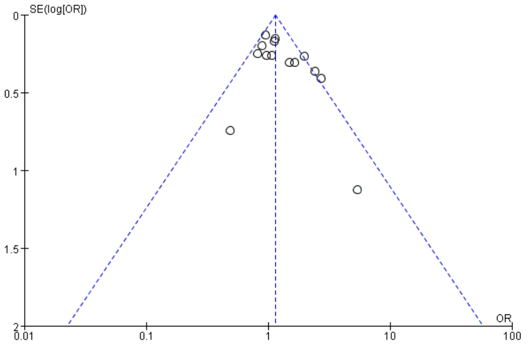

(53) rs3892097(GA+AA)

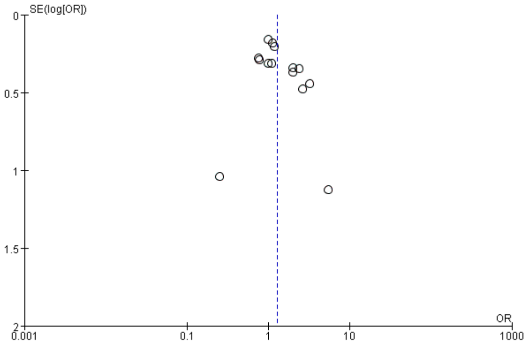

(54) rs3892097(AA)

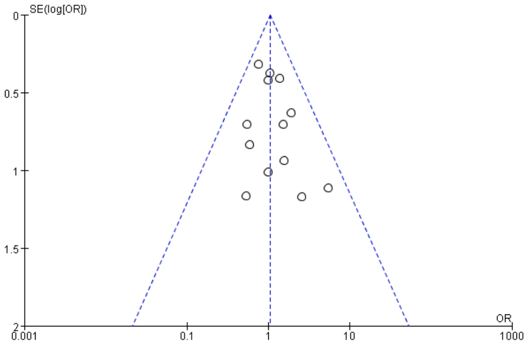

(55) A2637(MAF- -)

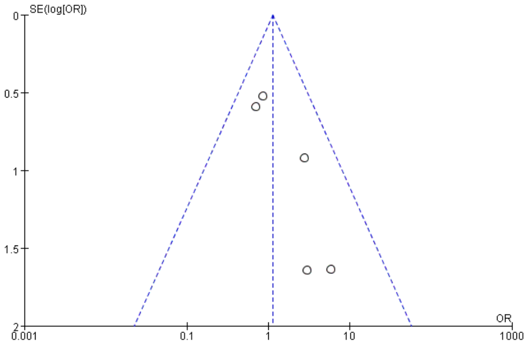

(56) A2637(A-+ --)

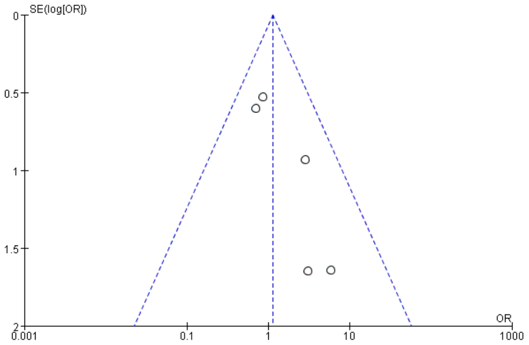

(57) rs2031920 (MAF-T)

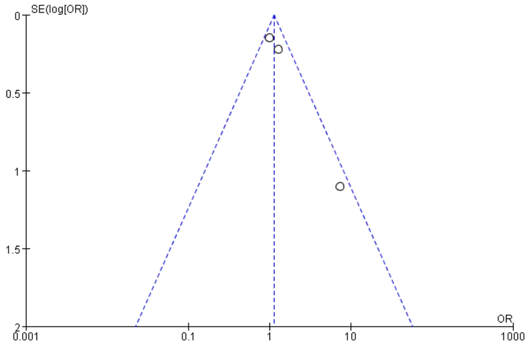

(58) rs2031920 (CT+TT)

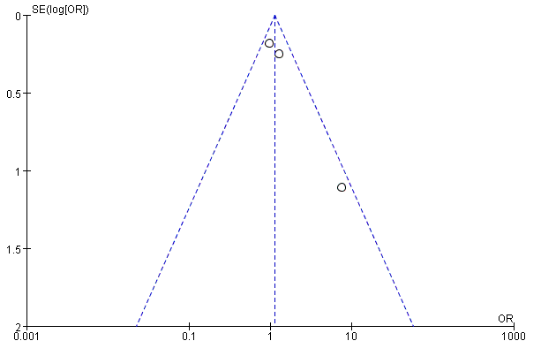

(59) rs2031920 (TT)

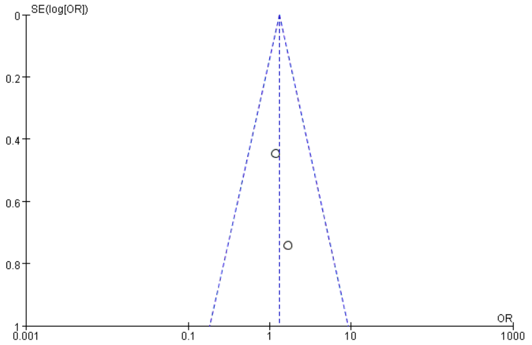

(60) rs705379, C-108T(MAF-T)

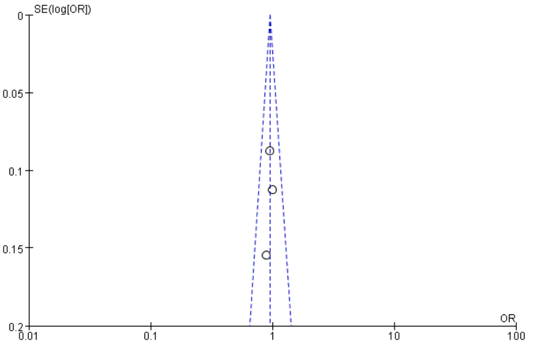

(61) rs705379, C-108T(CT+TT)

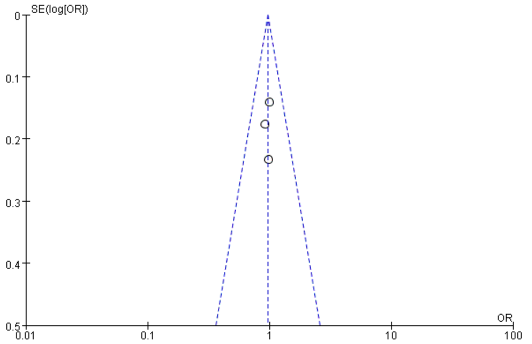

(62) rs705379, C-108T(TT)

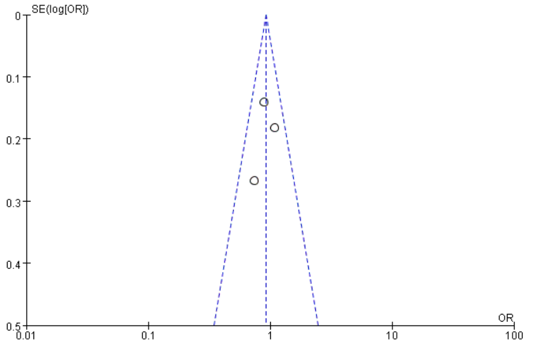

(63) rs854560(MAF-A)

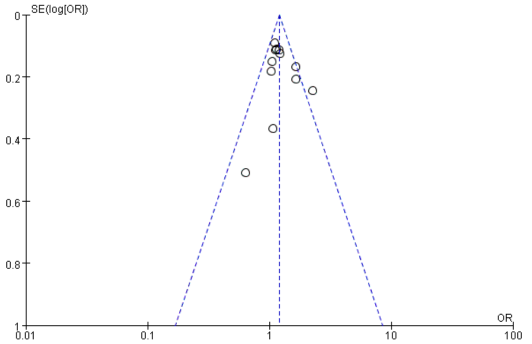

(64) rs854560(TA+AA)

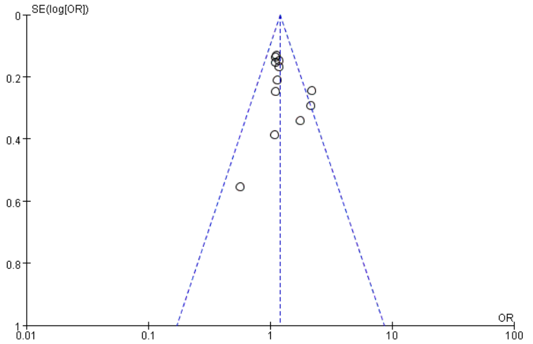

(65) rs854560(AA)

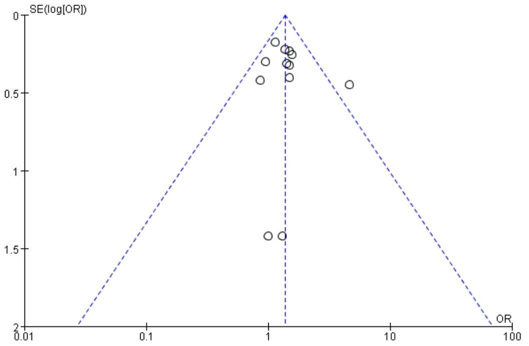

(66) rs662, Q192R(MAF-G)

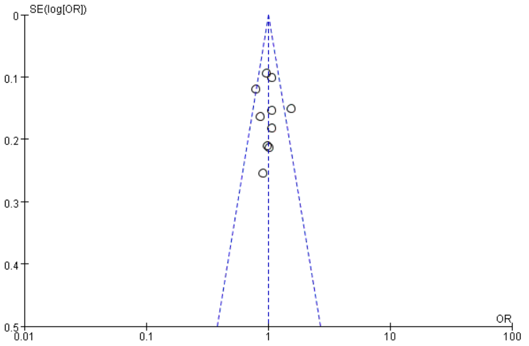

(67) rs662, Q192R(AG+GG)

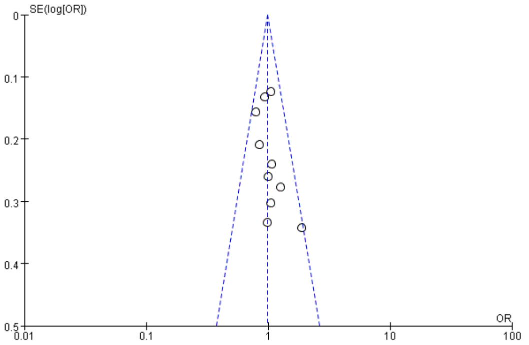

(68) rs662, Q192R(GG)

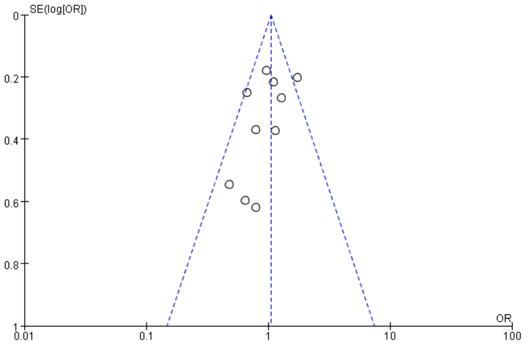

（69）rs1799929(MAF-T)

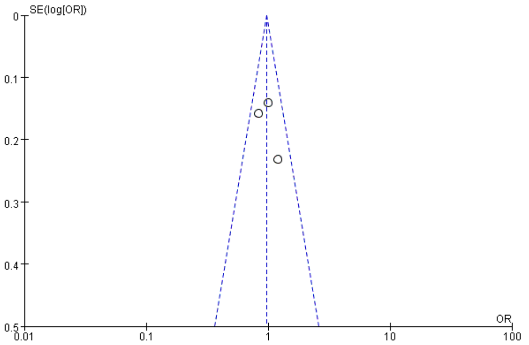

（70）rs1799929(CT+TT)

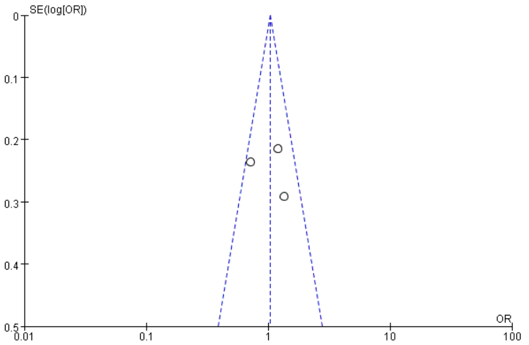

（71）rs1799929(TT)

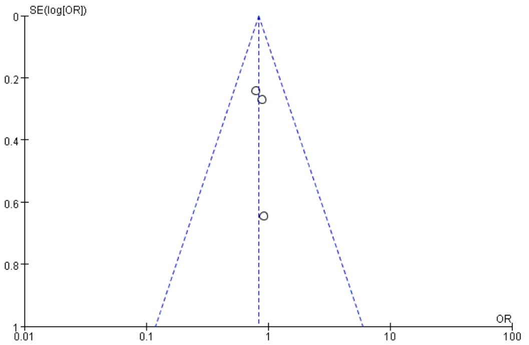

（72）rs1799929(MAF-A)

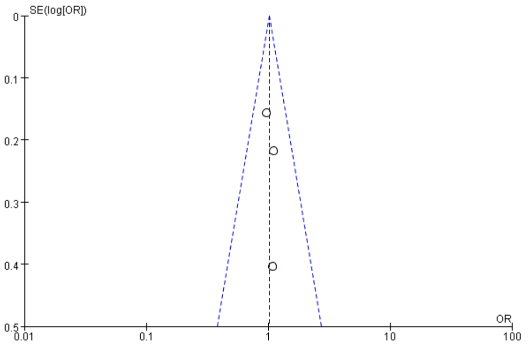

（73）rs1799929(GA+AA)

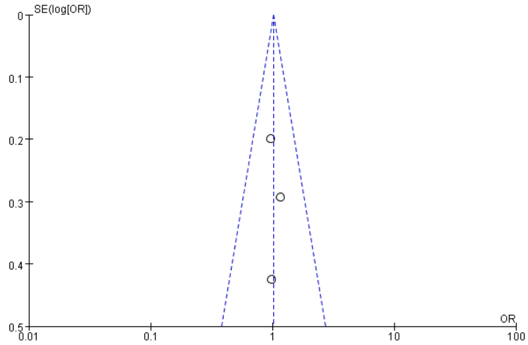

（74）rs1799929(AA)

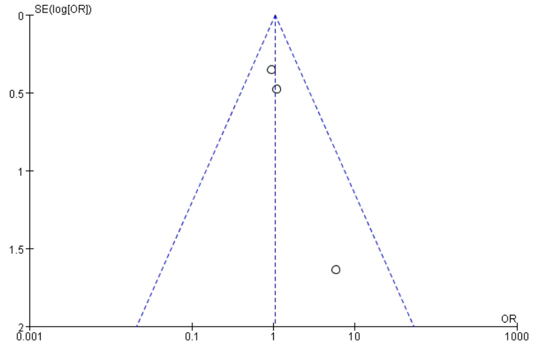

（75）rs1128503（MAF-T）

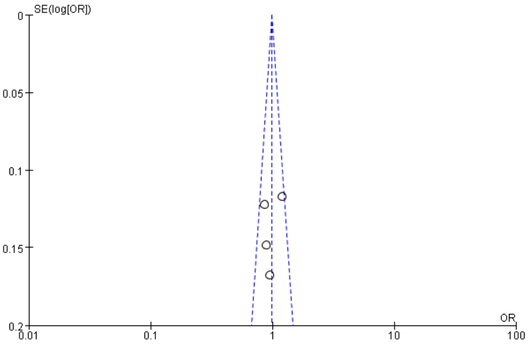

（76）rs1128503（CT+TT）

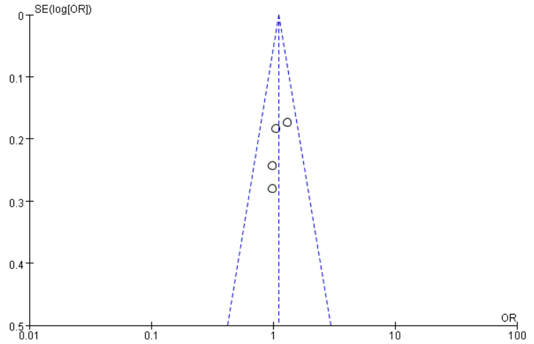

（77）rs1128503（TT）

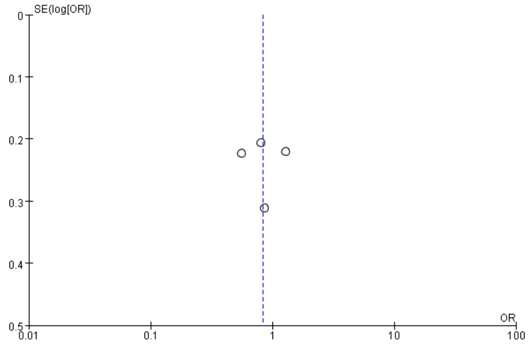

（78）rs1045642(MAF-T)

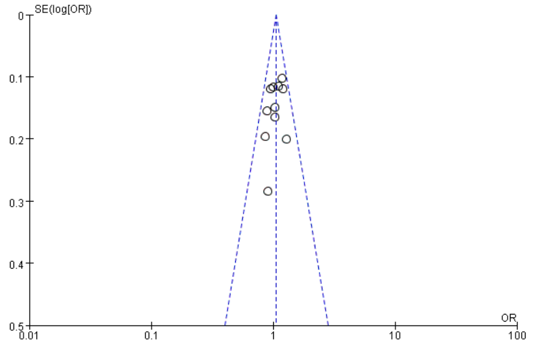

(79) rs1045642(CT+TT)

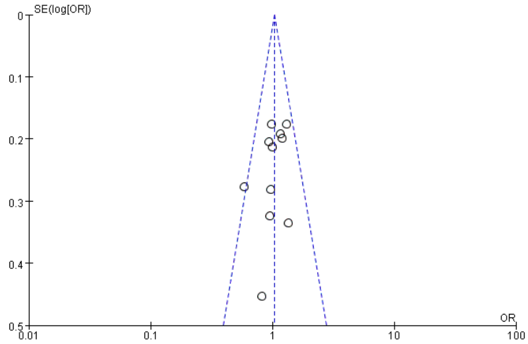

(80) rs1045642(TT)

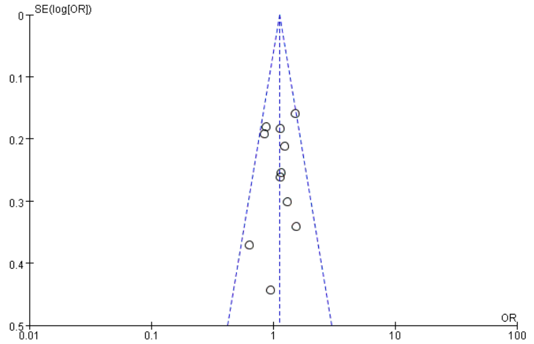

(81) rs2032582(MAF-G, A)

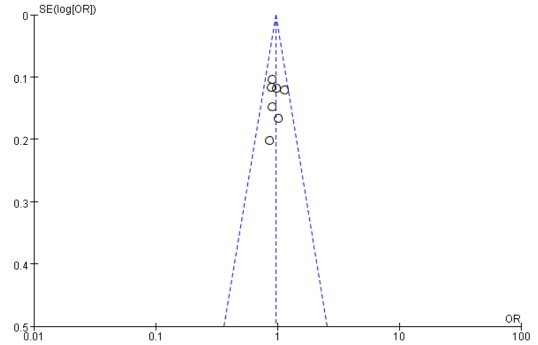

(82) rs2032582(TG,TA)

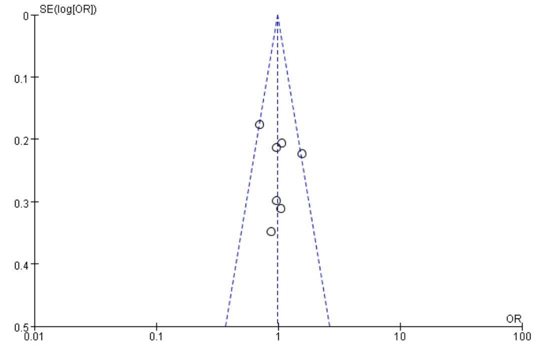

(83) rs2032582(MAF-GG,GA,AA)

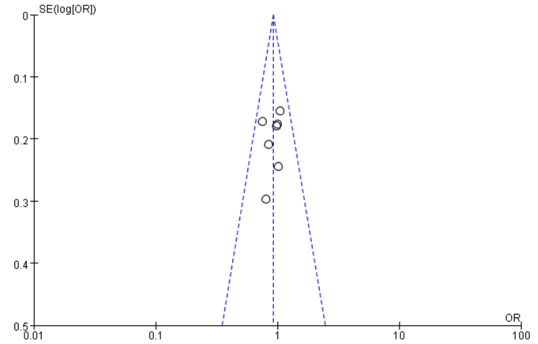

(84) rs11724635(MAF-A)

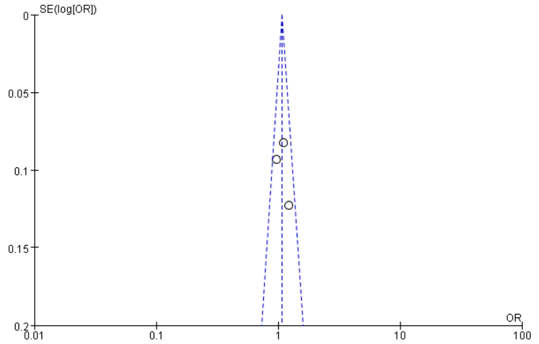

(85) rs11724635(CA+AA)

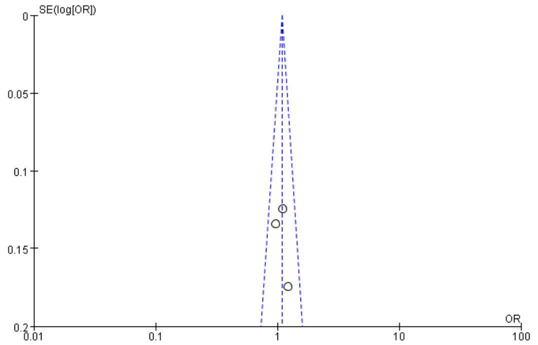

(86) rs11724635(AA)

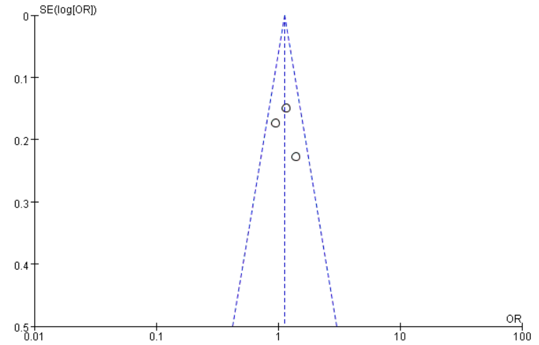

(87) rs11931532(MAF-C)

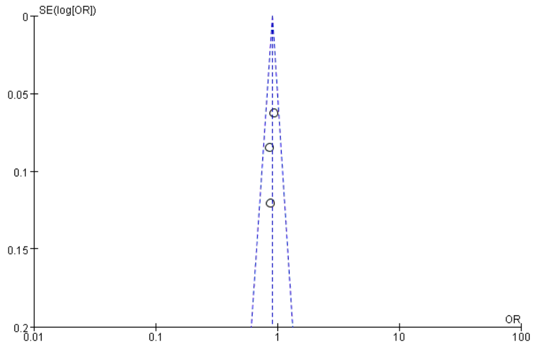

(88) rs11931532(TC+CC)

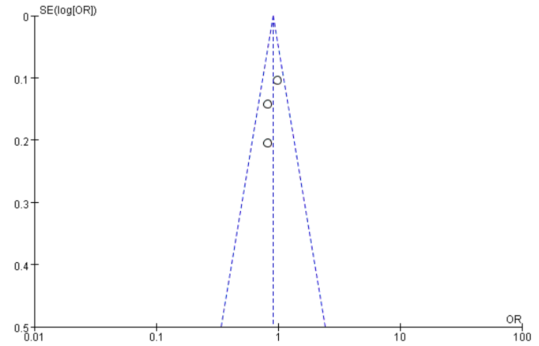

(89) rs11931532(CC)

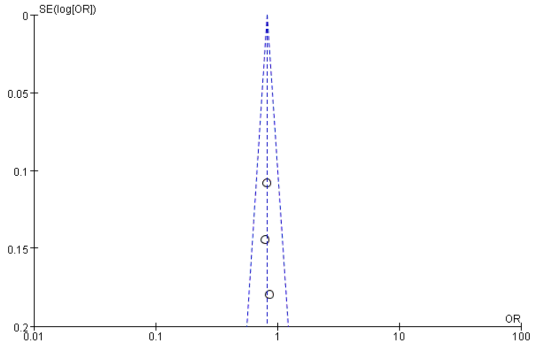

(90) rs660895(MAF-G)

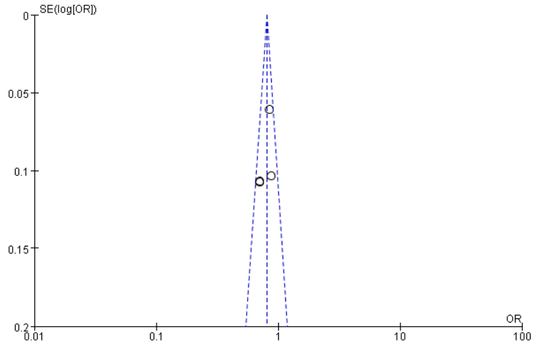

(91) rs660895(AG+GG)

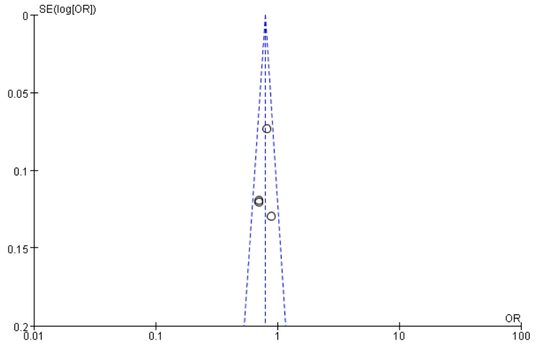

(92) rs660895(GG)

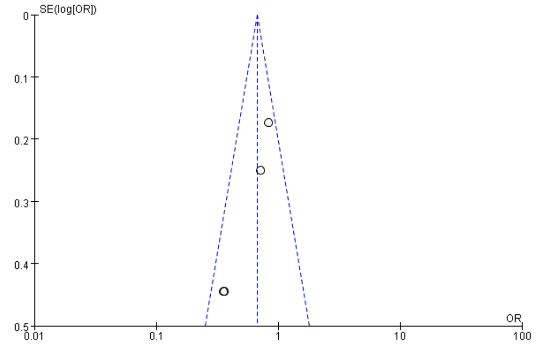

(93) rs12817488(MAF-G)

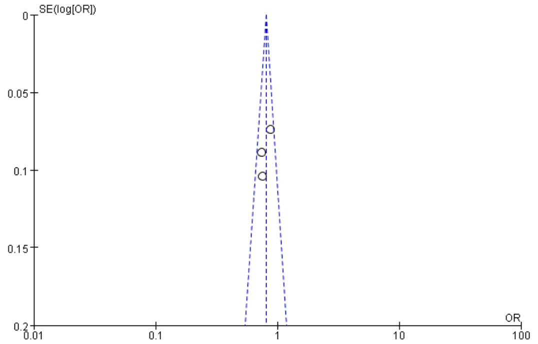

(94) rs12817488(AG+GG)

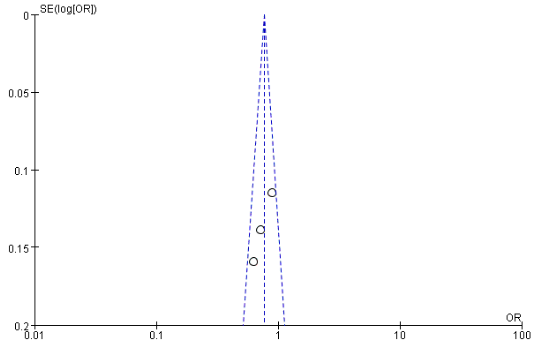

(95) rs12817488(GG)

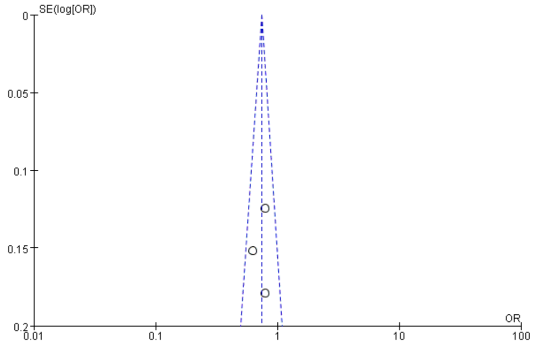

(96) rs1800562, C282Y(MAF-A)

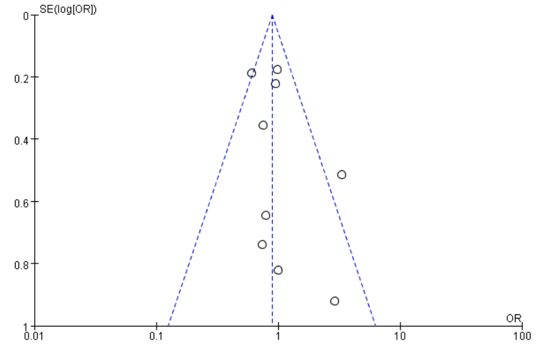

(97) rs1800562, C282Y(GA+AA)

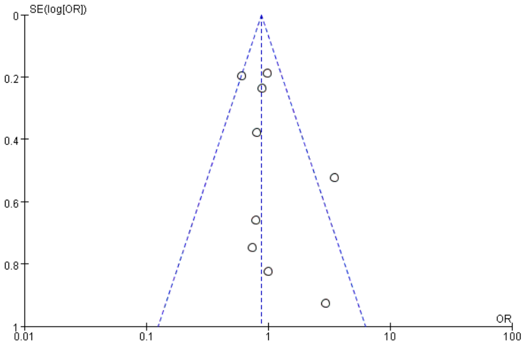

(98) rs1800562, C282Y(AA)

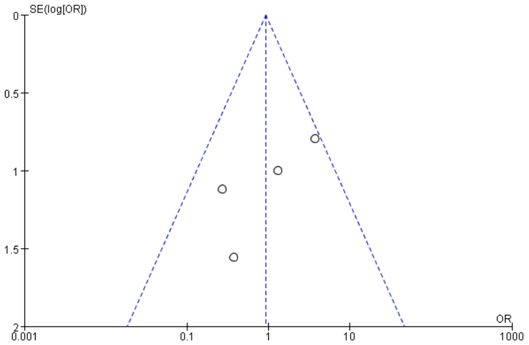

(99) rs1799945, H63D(MAF-G)

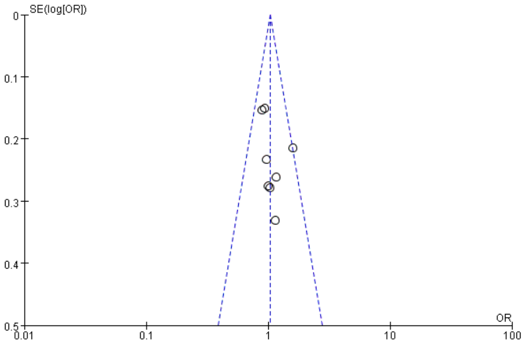

(100) rs1799945, H63D(CG+GG)

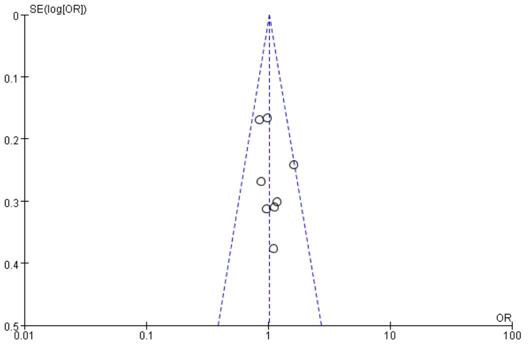

(101) rs1799945, H63D(GG)

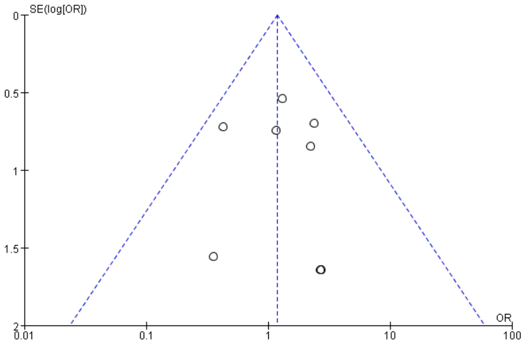

(102) rs1801133(MAF-T)

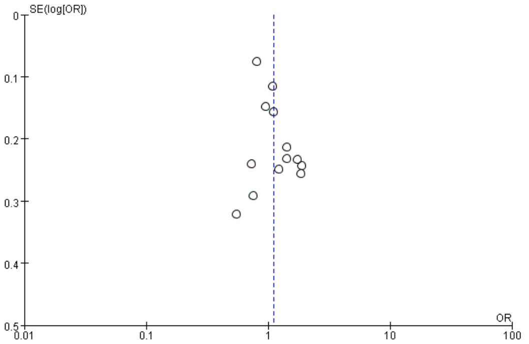

(103) rs1801133(CT+TT)

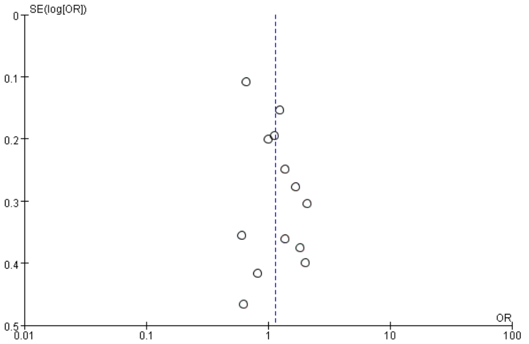

(104) rs1801133(TT)

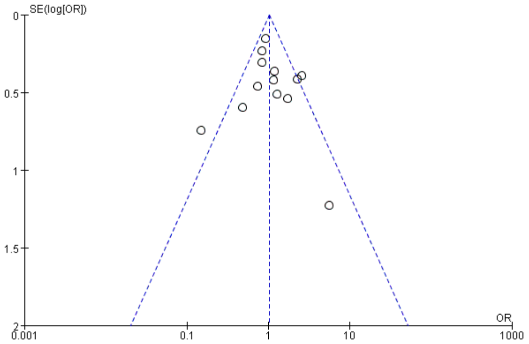

(105) rs1801131(MAF-C)

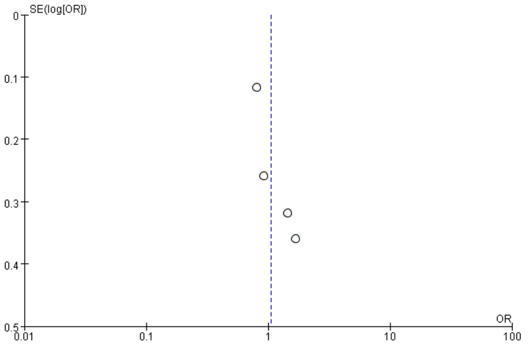

(106) rs1801131(AC+CC)

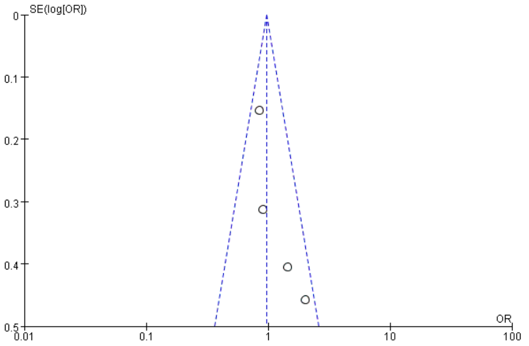

(107) rs1801131(CC)

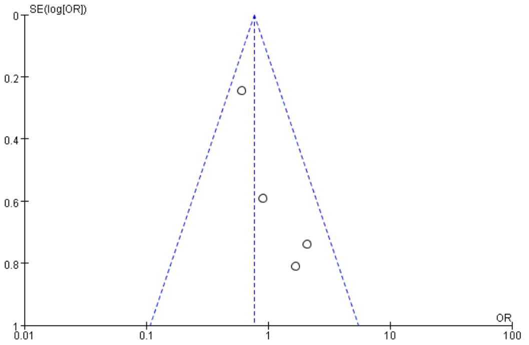

Supplement: Supplementary file 2 [file DataSheet_2.pdf]
